# Towards Enantiomerically Pure Unnatural α-Amino Acids via Photoredox Catalytic 1,4-Additions to a Chiral Dehydroalanine

**DOI:** 10.1021/acs.joc.2c01774

**Published:** 2022-09-30

**Authors:** Paula Oroz, Claudio D. Navo, Alberto Avenoza, Jesús H. Busto, Francisco Corzana, Gonzalo Jiménez-Osés, Jesús M. Peregrina

**Affiliations:** † Departamento de Química, Centro de Investigación en Síntesis Química, Universidad de La Rioja, 26006 Logroño, La Rioja, Spain; ‡ Center for Cooperative Research in Biosciences (CIC bioGUNE), Basque Research and Technology Alliance (BRTA), Bizkaia Technology Park, Building 800, 48160 Derio, Spain; § Ikerbasque, Basque Foundation for Science, 48013 Bilbao, Spain

## Abstract

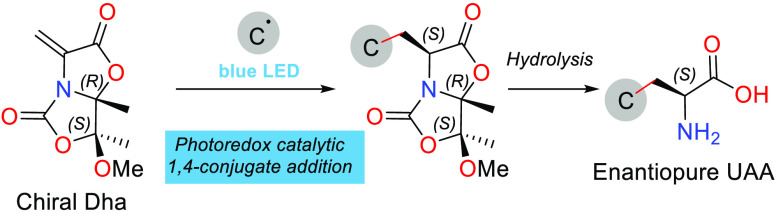

Chemo- and diastereoselective 1,4-conjugate additions of anionic and radical *C*-nucleophiles to a chiral bicyclic dehydroalanine (Dha) are described. Of particular importance, radical carbon photolysis by a catalytic photoredox process using a simple method with a metal-free photocatalyst provides exceptional yields and selectivities at room temperature. Moreover, these 1,4-conjugate additions offer an excellent starting point for synthesizing enantiomerically pure carbon-β-substituted unnatural α-amino acids (UAAs), which could have a high potential for applications in chemical biology.

## Introduction

**Scheme 1:**
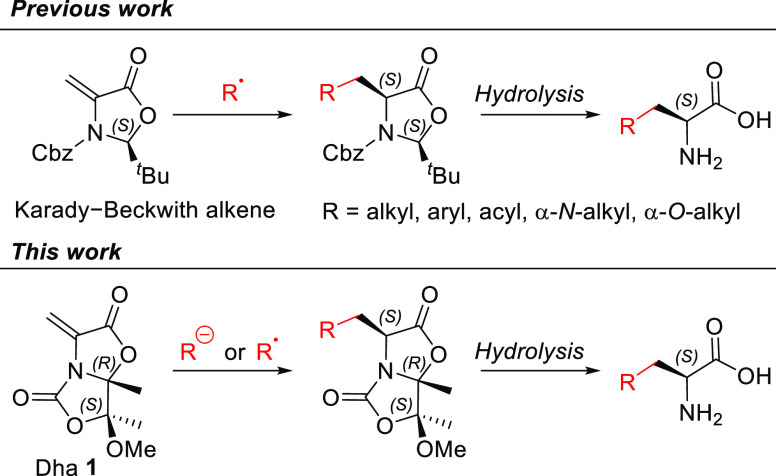
Synthesis of Enantiopure Carbon-β-Substituted UAAs via 1,4-Conjugate Addition to Chiral Dehydroalanines

Access to enantiopure unnatural α-amino acids (UAAs) remains a challenge in organic chemistry, especially those bearing side-chain diversity in their structure because they are common components of pharmaceuticals or medicinal chemistry targets.^1^ Beyond applications to modulate the conformational space of peptides and then their biological functions, UAAs are particularly relevant in stereoselective synthesis as chiral ligands and auxiliaries. Thus, synthetic methodologies for generating libraries of diverse enantiomerically pure UAAs are valuable to access relevant molecules in the field of drug design and peptide chemistry.^2^ In this regard, various methods have been described to generate enantiopure carbon-β-substituted UAA. However, until recent years, less attention has been paid to carbon nucleophilic 1,4-conjugate addition reactions to dehydroamino acids due to the difficult stereochemical control, especially at the α-carbon.^3^ Moreover, the use of highly reactive organometallic reagents such as organomagnesiun derivatives usually leads to mixtures of 1,4- and 1,2-adducts^4^ as we have seen in this article (see Supporting Information, SI, Table S1). A few examples are reported in which the treatment of dehydroalanine derivatives (Dha) with anionic carbon species afforded carbon-β-substituted derivatives through asymmetric Michael addition reactions.^5^ On the other hand, current synthetic methodologies have focused on visible-light-mediated catalytic methods that offer the advantages of using mild conditions, which allow for selective and controlled reactions.^6^ Thus, very recently and employing a variety of precursors, several radical 1,4-conjugate additions to Dha derivatives by photoredox catalytic reactions have been deeply explored to synthesize racemic UAAs.^7^ However, only very few cases have been reported to obtain enantiopure UAAs or their precursors,^8^ allowing the incorporation of different alkyl or aryl radicals at β-carbon, and most of them involved the use of a modified chiral Dha termed Karady–Beckwith alkene.^9^ Hence, inspired by these works and following the methodology established by our group,^10^ we envisioned the synthesis of enantiopure UAAs by using our 2nd-generation chiral Dha **1** as the starting material in both anionic carbon nucleophilic 1,4-attack and radical carbon photoredox catalytic 1,4-conjugate addition (Scheme 1).

## Results and Discussion

**Scheme 2:**
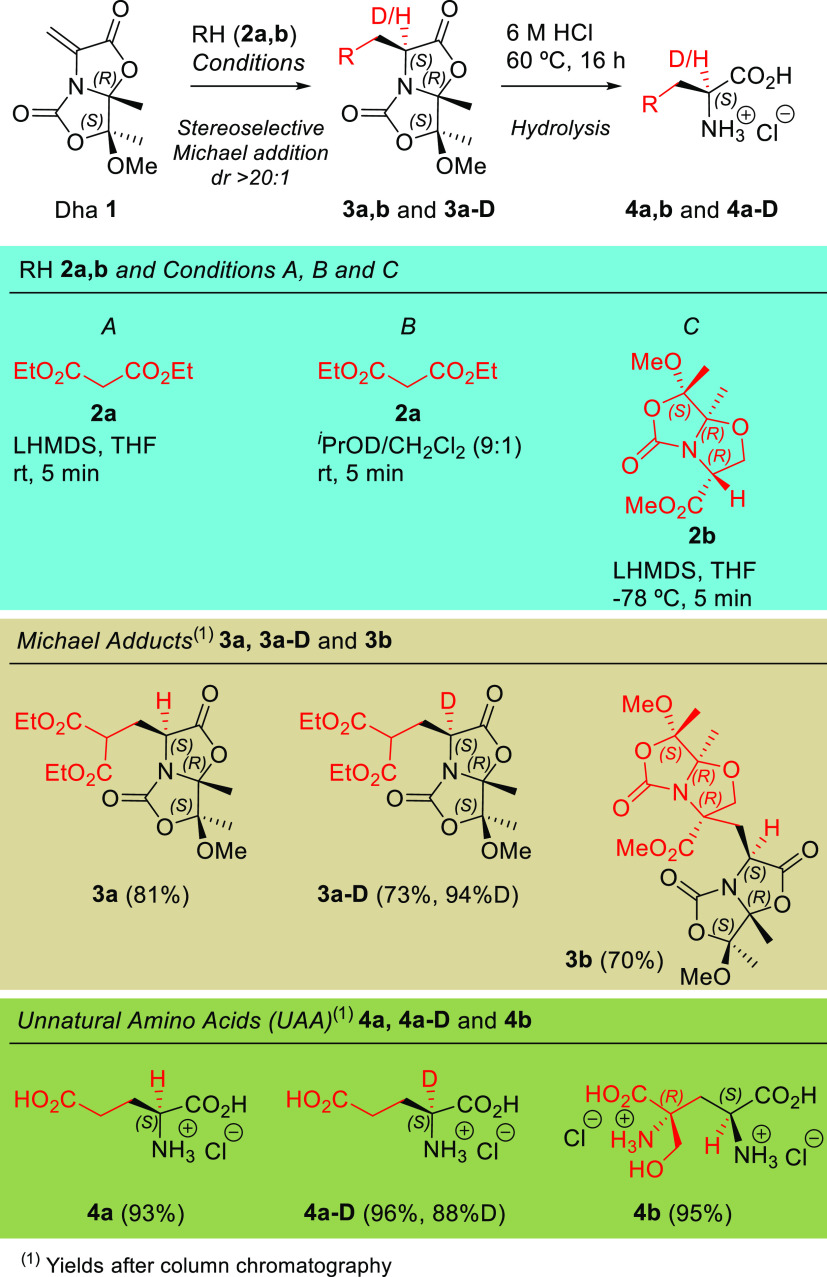
Synthesis of Enantiopure Glutamic Acid Derivatives via Stereoselective Michael Additions to Chiral Dha **1**

First, we assayed the 1,4-conjugated addition of some carbanions, generated *in situ* from their corresponding precursors, to Dha **1** as a Michael acceptor, following our protocol described for *S*-, *N*-, or *Se*-nucleophiles.^10^ However, the scope was very limited (SI) because we only achieved good results with diethyl malonate **2a** and a chiral bicyclic serine derivative **2b**. In the first case, diethyl malonate **2a** and Dha **1** were dissolved in dry tetrahydrofuran (THF) and lithium hexamethyldisilazide (LHMDS) was added at room temperature as a base (Conditions *A*, Scheme 2). The reaction was completed in 5 min and adduct **3a** was obtained in 81% yield after purification by column chromatography. In the case of chiral bicyclic serine derivative **2b**, the reaction conditions were similar, but the temperature had to be lowered to −78 °C to preserve the configuration of the substrate in the generated carbanion (Conditions *C*, Scheme 2), as we demonstrated previously.^11^ Once the reaction was completed (5 min), the corresponding adduct **3b** was obtained in a 70% yield after purification. Particularly relevant was the latter reaction since the 1,4-adduct **3b** displays 6 stereogenic centers and, most importantly, the chirality of the two ones generated in the global process is totally stereocontrolled. Both reactions took place with excellent yields. Acid hydrolysis and decarboxylation of adduct **3a** yielded enantiomerically pure glutamic acid **4a**. Given the importance of deuterium amino acids in medicinal chemistry,^8a,8b,12^ enantiomerically pure α-deuterated glutamic acid **4a**–**D** was synthesized by using a 9:1 mixture of 2-propanol-OD (^*i*^PrOD) and anhydrous CH_2_Cl_2_ as a solvent in the Michael addition (Conditions *B*, Scheme 2) followed by hydrolysis of adduct **3a**–**D** (Scheme 2).

On the other hand, hydrolysis of adduct **3b** led to bis-α-amino acid **4b** (Scheme 2), which is a 2,4-diaminoglutaric acid (Dag)^13^ featuring a chiral quaternary stereocenter. Chimeric amino acids are important scaffolds often used to stabilize 3D structures of peptides; therefore, this new amino acid can be regarded as a chimera that combines the steric and conformational properties of a α-alkyl-substituted Ser and Dag.^14^ In all adducts **3a**, **3a**–**D**, and **3b**, the absolute configurations of the new stereogenic centers created in the 1,4-conjugate additions were assessed by two-dimensional nuclear Overhauser effect spectroscopy (2D-NOESY) experiments and confirmed, in the case of **3b**, by X-ray crystallography (SI). In view of these results, the reaction mechanism is hypothesized to be similar to that proposed for *S*- or *Se*-nucleophilic 1,4-additions^10^ to Dha **1**. The stereochemical outcome of these Michael reactions to Dha **1** suggests a conserved stereoinduction mechanism for the protonation of the enolate adduct formed after conjugate addition (SI).

To increase the scope of these 1,4-additions, we focused on the photoredox catalytic 1,4-additions to chiral Dha **1**. Following the methodology used by Wang^8a^ and Schubert,^8b^ we envisioned the addition of a decarboxylated radical, starting from readily available and inexpensive alkylcarboxylic acids to Dha **1**, including the α-deuterating version of the reaction. We first assayed the reaction between Boc-Gly (**2c**) and methyl 2-acetamidoacrylate to test different conditions under blue light-emitting diode (LED) irradiation (SI, Table S2), and the better ones were transferred to chiral Dha **1**.

**Scheme 3:**
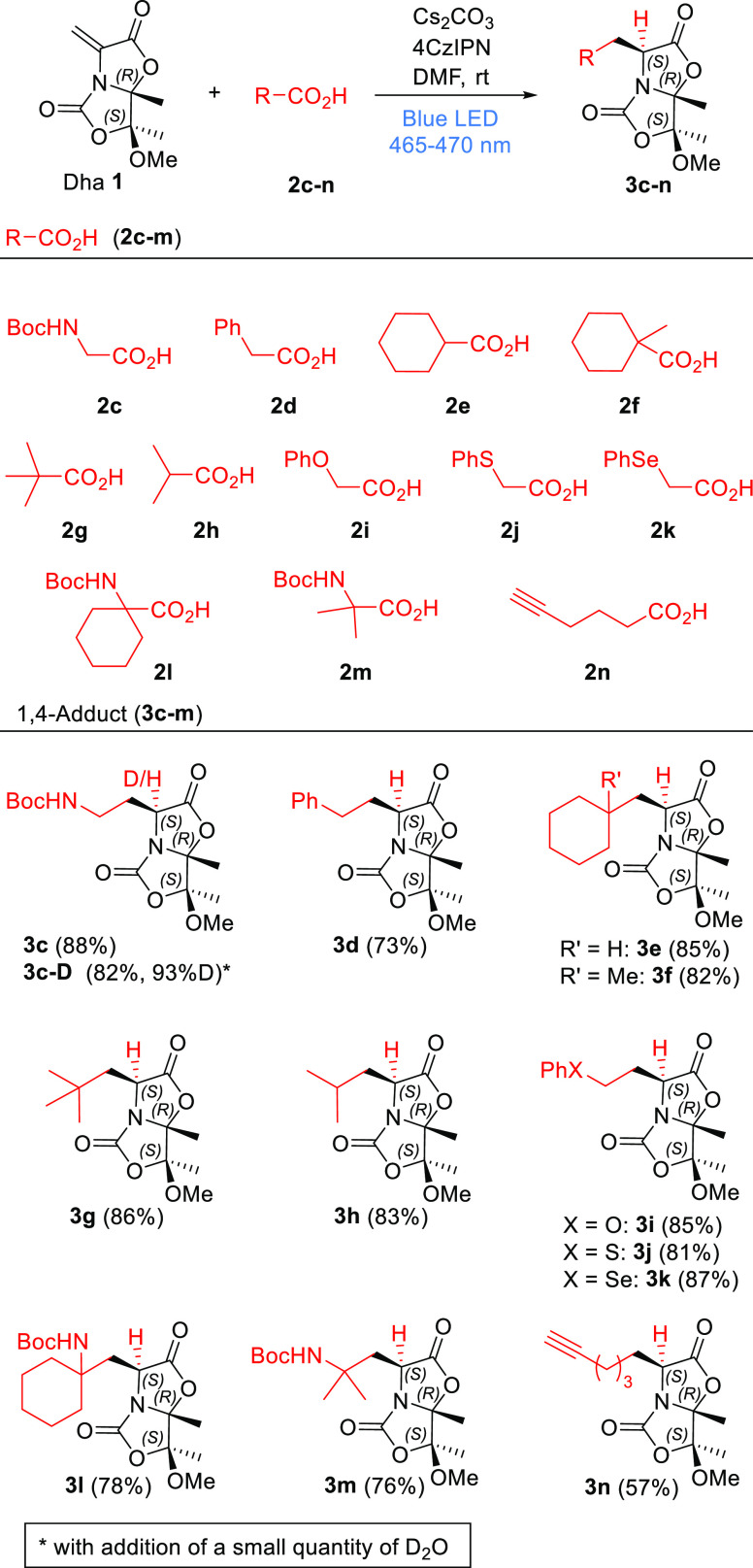
Photoredox Catalytic 1,4-Additions to Chiral Dha **1**

After testing several conditions using different solvents and catalysts (SI), the optimum conditions involved the use of Dha **1** (1.0 equiv), carboxylic acid **2c** (1.2 equiv), Cs_2_CO_3_ (1.5 equiv) as a base, and 4CzIPN (0.05 equiv) as a catalyst in *N*,*N*-dimethylformamide (DMF) as a solvent at room temperature. Once the reaction was completed after 16 h of irradiation, we observed the clean formation of a single diastereomer, corresponding to adduct **3c** (88% yield, Scheme 3).

**Scheme 4:**
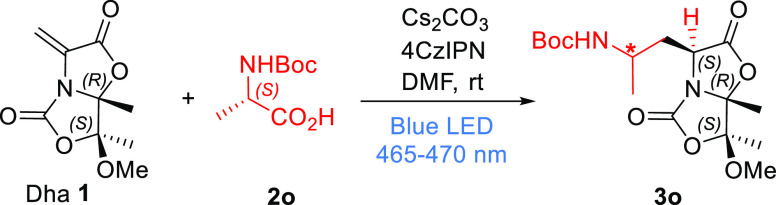
Photoredox Catalytic 1,4-Addition of Boc-l-Ala to Chiral Dha **1**

The scope of the reaction was examined by reacting carboxylic acids **2c**–**n** with Dha **1**, which generated adducts **3c**–**n** in good yields (Scheme 3). In the case of α-deuterated derivative **3c**–**D** (82% yield and 93% deuteration, Scheme 3), it was necessary to add a small quantity of D_2_O (50 μL) to the reaction. Besides carbamate-protected amine **2c**, we explored several functionalities in the structure of carboxylic acids such as ether (**2i**), thioether (**2j**), and selenoether (**2k**), which were well-tolerated and afforded desired products **3i**–**k** in excellent yields. The reaction of Dha **1** with benzylic and secondary alkylcarboxylic acids (**2d**, **2e**, and **2h**, respectively), as well as with other highly hindered tertiary alkylcarboxylic acids (**2f** and **2g**), also gave excellent yields of adducts **3d**–**h** (Scheme 3). In addition, the reaction with Boc-protected α,α-disubstituted α-amino acids **2l** and **2m** gave excellent yields of adducts **3l** and **3m**, respectively. Finally, we assayed the photoredox reaction of Dha **1** with the important carboxylic acid **2n**, which bears a reactive alkyne group in 1,3-dipolar cycloadditions. Therefore, the corresponding UAA derived from adduct **3n** would be of application in bioconjugation chemistry. As expected, the reaction proceeds with an adequate yield (57%) of adduct **3n**. However, and unfortunately, although this reaction works with 100% conversions with amino acids that present chirality at the α-carbon (Boc-l-Ala, Boc-l-Leu, or Boc-l-Pro), it is impossible to control the chirality of the generated radical, resulting in a mixture of adducts in similar ratios (SI). As an example, Scheme 4 shows the reaction of Dha **1** with Boc-l-Ala **2o** to give the mixture of diastereomers **3o**.

**Scheme 5:**
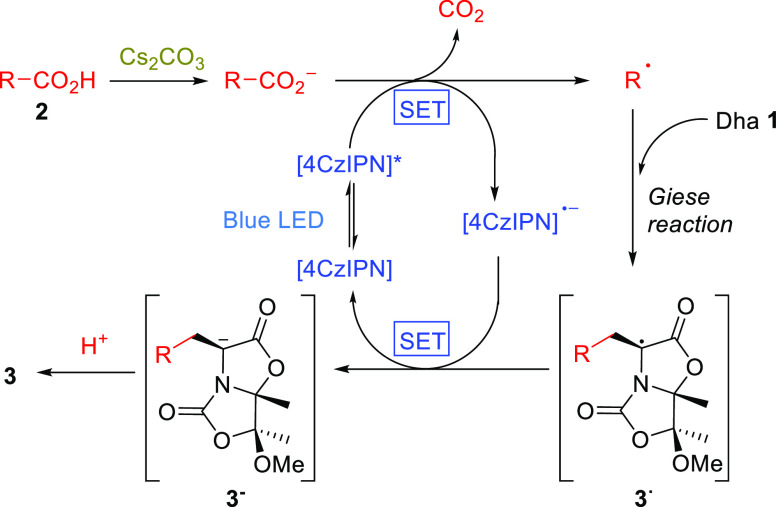
Mechanism for the Giese Reactions on Dha **1**

Based on the above-described results, we propose that these photoredox catalytic Giese reactions proceed via the mechanism shown in Scheme 5. Initially, Cs_2_CO_3_ deprotonates the carboxylic acid **2**, and once the photocatalyst [4CzIPN] is excited by irradiation at 465–470 nm, the excited-state catalyst [4CzIPN]* led to decarboxylation to generate an alkyl radical (R^•^), which is added to the Dha **1** to afford radical intermediate **3**^•^. This carbon radical is reduced to enolate **3**^**–**^, which is trapped by a proton/deuterium to give the 1,4-adduct **3**. Simultaneously, conversion of [4CzIPN]^•**–**^ to the [4CzIPN] catalyst completes the catalytic cycle.

**Figure 1:**
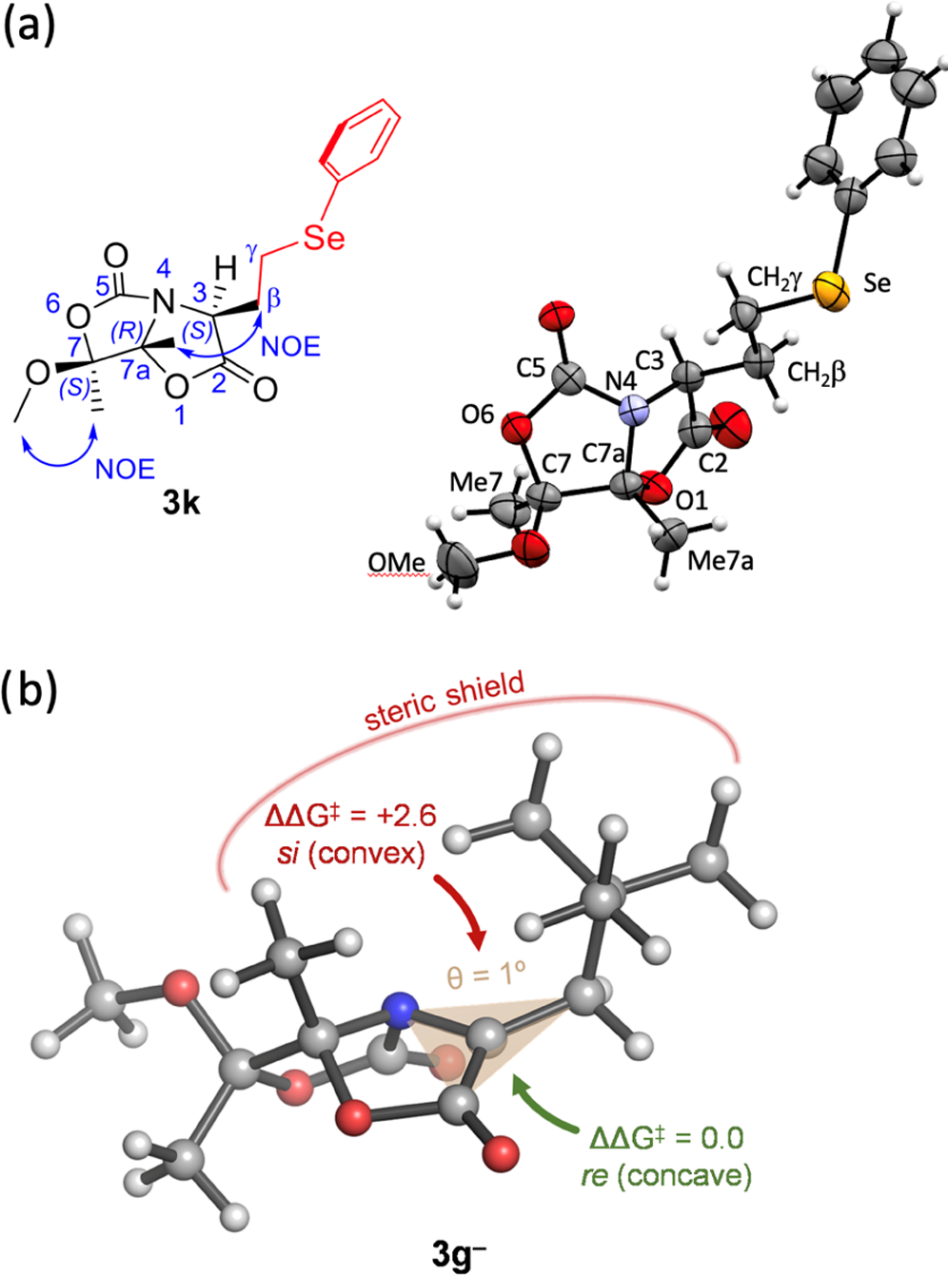
(a) ORTEP diagram of compound **3k**, showing thermal ellipsoids at the 75% probability level. (b) Lowest-energy structure for enolate intermediate **3g**– calculated with PCM(DMF)/M06-2X/6-31+G(d,p). θ represents the out-of-plane angle of Cβ with respect to the plane defined by C2, C3, and N4. Angles close to 0° correspond to planarity (i.e., negligible pyramidalization at Cα). Relative activation barriers (ΔΔ*G*^‡^ in kcal mol^–1^) for protonation with HCO_3_^–^ by the *re* and *si* faces are indicated with green and red arrows, respectively (see the SI, Figure S7, for further details).

In all adducts **3c**–**m**, the absolute configurations of the new stereogenic centers created in the Giese reactions were assessed by 2D-NOESY experiments (SI). Alternatively, this structural feature was also determined by X-ray analysis of monocrystals of compound **3k** (Figure 1a). As described above for the anionic 1,4-additions, the stereochemical outcome of these Giese reactions on Dha **1** indicates a highly conserved stereoinduction mechanism.

The proposed mechanism for the addition of *tert*-butyl (**g**^**•**^), iso-propyl (**h**^**•**^), and model ethyl (**Et**^**•**^) radicals to Dha **1** was studied computationally using the PCM(DMF)/M06-2X/6-31+G(d,p) method (SI, Figures S4–S6). The computed activation barriers for the Giese reaction were relatively low (Δ*G*^‡^ ≈ 11 kcal mol^–1^) leading to stable radical intermediates **3**^**•**^ (Δ*G* ≈ −22 kcal mol^–1^) and enolate intermediates **3**^–^ (Δ*G* ≈ −36 kcal mol^–1^) upon single-electron transfer (SET). In contrast to what was observed for β-thioenolates,^10a^ the calculated lowest-energy structures of β-alkylenolate intermediates (ultimately responsible for stereoselection) displayed low (θ = 6° for less hindered **3Et**^–^) to negligible (θ < 1° for **3g**^–^ and **3h**^–^) pyramidalization at the α-carbon (C3). A similar effect was observed for enolate **3a**^–^ (θ = 4°). The steric hindrance between the bridgehead methyl group and the β-substituent makes the latter tilt away, overcoming the native tendency of the bicyclic scaffold to yield pyramidalized enolates and resulting in an almost planar α-carbon. As a consequence, the usually more accessible convex face is completely shielded, favoring protonation by the concave face (Figure 1b and SI, Figure S6).

In fact, protonation of enolate **3g**^–^ with hydrogencarbonate (**HCO**_**3**_^–^) as a proton source by the concave (*re*) face (**3g**^–^**_TSprot_***re*) induced a conformational change of the bicyclic scaffold to avoid steric clashes with the substituent at Cβ upon sp^2^ → sp^3^ rehybridization of Cα (SI, Figure S7). Nevertheless, and in excellent agreement with the observed stereoselectivity, this reaction pathway is 2.6 kcal mol^–1^ more favorable than protonation by the convex (*si*) face (**3g**^–^**_TSprot_***si*) due to significant repulsion between the hydrogencarbonate anion and both the bridgehead methyl group and the substituent at Cβ (Figures 1b and S7 in the SI). A similar trend is observed for the least stable rotamer of the enolate (**3g**^–^_*conf2*), which already ca. 3 kcal mol^–1^ higher in energy than **3g**^–^ (SI, Figure S5), resulting hence in even higher activation energies for the protonation by either face (ΔΔ*G*^‡^ = 3–4 kcal mol^–1^) (SI, Figure S7).

**Scheme 6:**
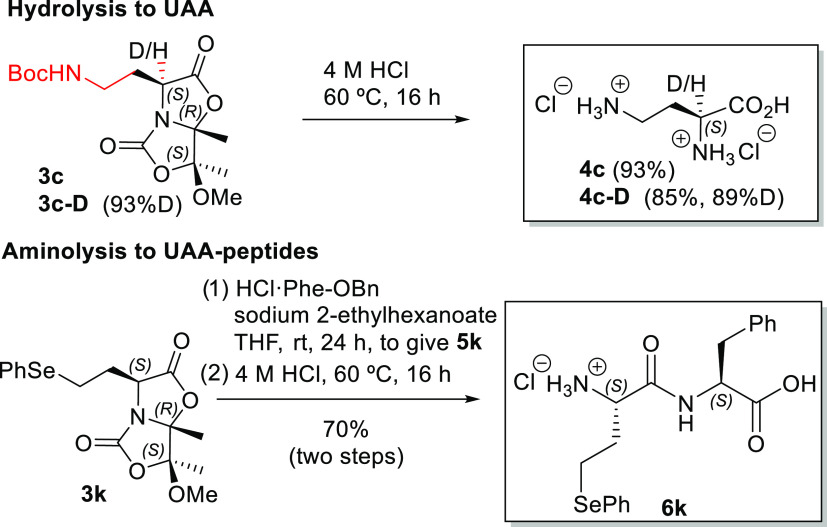
Synthetic Applications of 1,4-Adducts **3**

Hydrolysis of adduct **3c** led to 2,4-diaminobutyric acid (Dab, **4c**) in a good yield (93%). Dab is an important UAA that appears in the structure of several polymyxin antibiotics. Besides, Dab is a neurotoxin with antitumor effects.^15,16^ Using the same deuteration methodology, α-deuterated Dab **4c**–**D** was synthesized (89% yield and 89% deuteration, Scheme 6). Adducts **3d**, **3e**, **3g**, **3i**, and **3j** are precursors of l-homophenylalanine (Hph), 3-cyclohexylalanine (Cha), 3-*tert*-butylalanine (Tba), *O*-phenylhomoserine [Hse(*O*Ph)], and *S*-phenylhomocysteine [Hcy(*S*Ph)], respectively, which are relevant UAAs involved in different biological studies.^17,18^ Particularly significant is the adduct **3k** as the precursor of *Se*-phenylhomoselenocysteine [Hsc(*Se*Ph)], which is a selenoamino acid with potential use in native chemical ligation.^19^

The methodology reported herein deals with a new chiral Giese acceptor (Dha, **1**), different from Karady–Beckwith alkene, highly stereoselective at room temperature, providing clean reactions and high yields of 1,4-adducts **3c**–**n**. The subsequent deprotection of these derivatives allows for the synthesis of a variety of UAA **4**. Apart from these synthetic advantages, Dha **1** offers the attractive feature that its carboxylic acid group is efficiently protected and activated in the form of oxazolidine-5-one, which allows coupling with amino acids to obtain peptides. Thus, as a synthetic application of 1,4-aducts **3**, we coupled **3k** with the α-amino ester hydrochloride derived from Phe in the presence of sodium 2-ethylhexanoate as a base to give dipeptide **5k** in a good yield (74%, Scheme 6). The acidic hydrolysis of **5k** with 4 M HCl at 60 °C and subsequent purification by semipreparative HPLC gave enantiopure L-Hsc(*Se*Ph)-l-Phe (**6k**) in high yields (95 and 70% global yield for two steps, Scheme 5).

**Scheme 7:**
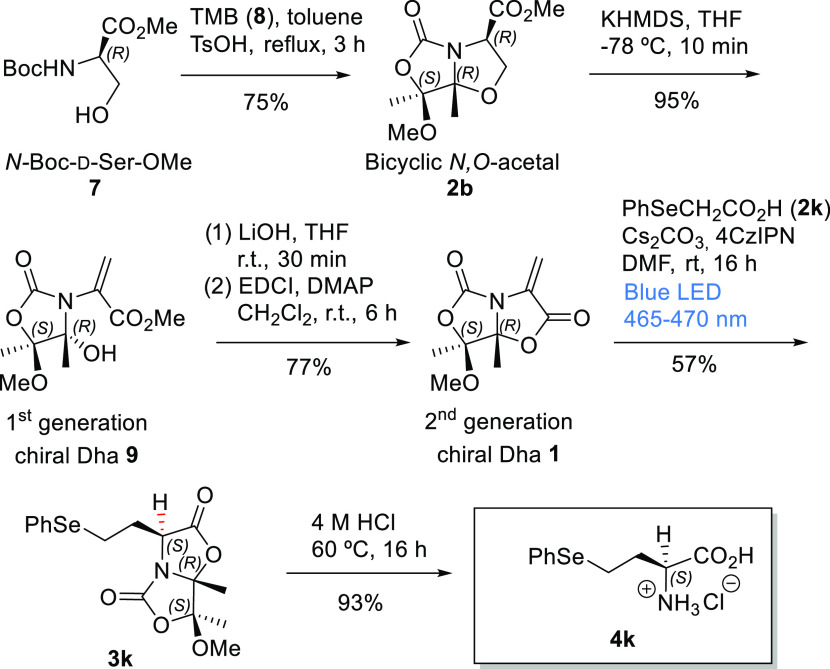
Synthetic Procedure to Obtain the UAA Hsc(*Se*Ph) **4k** from Ser Derivative **7**

In addition, to demonstrate the feasibility of this synthetic process to afford unnatural amino acids, we have scaled up the synthesis of the UAA *Se*-phenylhomoselenocysteine **4k**, including the synthetic procedure to obtain the chiral substrate Dha **1** (on a gram scale) from readily available raw materials (Scheme 7 and SI).

Thus, following our procedure previously published,^11a^ starting from 11.4 g of (*R*)-*N*-Boc-serine methyl ester (*N*-Boc-d-Ser-OMe, **7**) and 2,2,3,3-tetramethoxybutane (TMB, **8**), the corresponding bicyclic *N*,*O*-acetal **2b** was obtained in a 75% yield (9.4 g). This compound **2b** (2.8 g) was transformed with a 95% yield into the first-generation chiral Dha **9** (2.6 g). The second-generation Dha **1** (1.4 g) was obtained from Dha **9** (2.1 g) through basic hydrolysis followed by an internal coupling (lactonization).^10a^ The photoredox catalytic 1,4-conjugate addition of PhSeCH_2_CO_2_H (**2k**) to Dha **1** (200 mg) gave the adduct **3k** (205 mg, 57%) with somewhat less yield than that obtained with smaller amounts. Next, an amount of **3k** (45 mg) was readily hydrolyzed to the UAA Hsc(*Se*Ph) **4k** (27 mg, 93%). Finally, our methodology allows to obtain Hsc(*Se*Ph) from a Ser derivative using six steps with an overall yield of 69%. This method competes with several published methods to obtain *Se*-substituted homoselenocysteine derivatives.^19^ For instance, from *tert*-butyl 2-((diphenylmethylene)amino)acetate, a glycine derivative, and using five steps, *N*-Boc-*Se*-(*ortho*-nitrophenyl)selenohomocysteine *tert*-butyl ester was obtained in an overall yield of 50%. In the same way, free *Se*-(methyl)selenohomocysteine (also known as selenomethionine) was obtained using eight steps in an overall yield of 32%. In both cases, the stereoselectivity was introduced via a chiral alkylation of a glycine derivative using a chiral cinchona-derived phase-transfer catalyst.^19a,19b^ Moreover, selenomethionine has been used to synthesize *Se*-(*para*-methoxtbenzyl)homoselenocysteine via homoselenocystine in an overall yield of 62% (20% overall yield from the above-cited glycine derivative and using 10 steps).^19c^ In addition, (*S*)-*Se*-(phenyl)selenohomocysteine was synthesized from (*S*)-methionine using seven steps in an overall yield of 34% and all of them involved interconversion of functional groups.^19d^

In conclusion, this work describes the totally chemo- and stereoselective 1,4-conjugate additions of different anionic and radical *C*-nucleophiles to chiral bicyclic dehydroalanine Dha **1**. This methodology allows the synthesis of enantiopure unnatural amino acids (UAAs) with structural diversity. Besides, both the diastereoselective incorporation of β-carbon-sidechain and the selective α-deuteration occur concomitantly giving access to enantioenriched α-deuterated UAAs. This procedure not only utilizes the photoredox methodology based on visible light to generate enantiopure UAA but offers additional synthetic advantages. Thus, the oxazolidine-5-one skeleton of these 1,4-adducts gives access to peptides incorporating different UAAs. In summary, readily available starting materials, mild conditions, metal-free photocatalysts, functional group tolerance, and high yields and stereoselectivities make this strategy an appealing method for the synthesis of enantiomerically pure UAAs.

## Experimental Section

### General and Experimental Methods

Commercial reagents were used without further purification. Analytical thin-layer chromatography (TLC) was performed on Macherey-Nagel precoated aluminum sheets with a 0.20 mm thickness of silica gel 60 with the fluorescent indicator UV254. TLC plates were visualized with UV light and by staining with a potassium permanganate solution (0.75 g of KMnO_4_, 5 g of K_2_CO_3_, and 0.63 mL of 10% NaOH in 100 mL of water) or a ninhydrin solution (1.5 g of ninhydrin in 100 mL of *n*-butanol and 3.0 mL of acetic acid). Column chromatography was performed on silica gel (230–400 mesh). ^1^H and ^13^C{^1^H} NMR spectra were measured with a 300 or 400 MHz spectrometer with TMS as the internal standard. Multiplicities are quoted as singlet (s), broad singlet (br s), doublet (d), doublet of doublets (dd), triplet (t), or multiplet (m). Spectra were assigned using COSY and HSQC experiments. The results of these experiments were processed with MestreNova software. High-resolution electrospray mass (ESI) spectra were recorded on a microTOF spectrometer; accurate mass measurements were achieved by using sodium formate as an external reference.

### *C*-Michael Addition on Dha 1 Followed by Hydrolysis to Obtain Glutamic Acid Derivatives

Dha **1** was obtained in a gram scale from *N*-Boc-d-Ser-OMe **7** following the published procedure.^10a^ Compound **2a**, carboxylic acids **2c**–**j**, and **2l**–**o** are commercially available. Bicyclic compound **2b**,^11a^ carboxylic acid **2k**,^20^ and Boc-d-Ser-OMe^21^**7** were synthesized following the procedures described in the literature. The NMR spectra of these compounds were included in the SI.

#### Diethyl-2-(((3*S*,7*S*,7a*R*)-7-methoxy-7,7a-dimethyl-2,5-dioxotetrahydro-5*H*-oxazolo[4,3-*b*]oxazol-3-yl)methyl)malonate (**3a**)

Chiral bicyclic Dha **1** (21 mg, 0.1 mmol, 1.0 equiv) and diethyl malonate **2a** (18 μL, 0.11 mmol, 1.1 equiv) were dissolved, at room temperature, in anhydrous THF (final concentration 0.1 M) in a Schlenk under an Ar atmosphere. Then, a 1 M solution of LHMDS in THF (0.2 mL, 2.0 equiv) was added with a syringe. The reaction was monitored by TLC (7:3, hexanes/ethyl acetate. *R*_f_ (**Dha**) = 0.75) and, once completed (5 min), the solution was dried under vacuum. The crude mixture was purified by column chromatography on silica gel (hexanes/ethyl acetate, 7:3) to afford compound **3a** as a sticky foam (30 mg, 0.08 mmol, 81% yield). [α]_D_^20^ +83.7 (c 1.0, CHCl_3_). HRMS (ESI) *m*/*z*: [M + Na]^+^ calcd for C_16_H_23_NO_9_Na 396.1265; Found 396.1268. ^1^H NMR (CDCl_3_, 400 MHz): δ 4.43 (dd, 1H, *J* = 11.6, 5.2 Hz, H^3α^), 4.16–4.28 (m, 4H, 2CH_2_^b^), 3.66 (dd, 1H, *J* = 8.3, 6.0 Hz, H^a^), 3.48 (s, 3H, OMe^7^), 2.66 (ddd, 1H, *J* = 14.0, 8.3, 5.2 Hz, H^β^), 2.26 (ddd, 1H, *J* = 14.0, 11.6, 6.0 Hz, H^β^), 1.65 (s, 3H, CH_3_^7a^), 1.61 (s, 3H, CH_3_^7^), 1.24–1.32 (m, 6H, 2CH_2_^c^). ^13^C{^1^H} NMR (CDCl_3_, 100 MHz): δ 171.1, 168.6, 168.0, 159.2 (4CO), 108.3 (C^7^), 101.7 (C^7a^), 62.2 (C^b^), 62.1 (C^b^), 59.0 (C^3α^), 51.7 (OMe^7^), 48.9 (C^a^), 30.2 (C^β^), 22.0 (CH_3_^7a^), 16.6 (CH_3_^7^), 14.1 (C^c^), 14.1 (C^c^).

#### Methyl(3*R*,3′*S*,7*S*,7a*R*,7′*S*,7′a*R*)-7,7′-dimethoxy-7,7a,7′,7′a-tetramethyl-2′,5,5′ trioxohe-xahydro-5*H*,5′*H*-[3,3′-bioxazolo[4,3-*b*]oxazole]-3(2*H*)-carboxylate (**3b**)

Chiral bicyclic Dha **1** (21 mg, 0.1 mmol, 1.0 equiv) and chiral bicyclic serine derivative **2b** (24 mg, 0.1 mmol, 1.0 equiv) were dissolved in anhydrous THF (final concentration 0.1 M) in a Schlenk under Ar atmosphere. Then, the mixture was cooled down to −78 °C and a 1 M solution of LHMDS in THF (0.2 mL, 2.0 equiv) was added with a syringe. The reaction was monitored by TLC (7:3, hexanes/ethyl acetate. *R*_f_ (**Dha**) = 0.75) and,, once completed (5 min), the solution was dried under vacuum. The crude mixture was purified by column chromatography on silica gel (hexanes/ethyl acetate, 7:3) to afford compound **3b** as a sticky foam, but with time and solvents, monocrystals were formed. (32 mg, 0.07 mmol, 70% yield). [α]_D_^20^ +49.4 (c 1.0, CHCl_3_). HRMS (ESI) *m*/*z*: [M + Na]^+^ calcd for C_19_H_26_N_2_O_11_Na 481.1429; Found 481.1411. ^1^H NMR (CDCl_3_, 400 MHz): δ 5.00 (d, 1H, *J* = 9.2 Hz, H^2′^), 4.24 (dd, 1H, *J* = 11.0, 3.7 Hz, H^3α^), 4.16 (d, 1H, *J* = 9.2 Hz, H^2′^), 3.79 (s, 3H, CO_2_CH_3_), 3.48 (s, 3H, OMe^7^), 3.45 (s, 3H, OMe^7′^), 3.33 (dd, 1H, *J* = 14.7, 11.0 Hz, H^β^), 2.49 (dd, 1H, *J* = 14.7, 3.7 Hz, H^β^), 1.66 (s, 3H, CH_3_^7a^), 1.62 (s, 3H, CH_3_^7^), 1.56 (s, 3H, CH_3_^7′^), 1.36 (s, 3H, CH_3_^7′a^). ^13^C{^1^H} NMR (CDCl_3_, 100 MHz): δ 170.9, 170.5, 159.0, 154.7 (4CO), 107.8, 107.7, 102.5, 102.3 (4C^7,7′,7a,7′a^), 72.8 (C^2′^), 67.2 (C^3′^), 57.8 (C^3α^), 53.4 (CO_2_CH_3_), 51.7 (OMe^7^), 51.7 (OMe^7′^), 34.2 (C^β^), 21.5 (CH_3_^7a^), 18.6 (C^7′a^), 16.6 (CH_3_^7′^), 16.2 (CH_3_^7^).

#### Diethyl-2-(((3*S*,7*S*,7a*R*)-7-methoxy-7,7a-dimethyl-2,5-dioxotetrahydro-5*H*-oxazolo[4,3-*b*]oxazol-3-yl-3-*d*)methyl)malonate (**3a**–**D**)

Chiral bicyclic Dha **1** (21 mg, 0.1 mmol, 1.0 equiv) and diethyl malonate **2a** (18 μL, 0.11 mmol, 1.1 equiv) were dissolved, at room temperature, in a 9:1 mixture of 2-propanol-OD (^*i*^PrOD) and anhydrous CH_2_Cl_2_ (final concentration 0.1 M) in a Schlenk under an Ar atmosphere. Then, a 1 M solution of LHMDS in THF (0.2 mL, 2.0 equiv) was added with a syringe. The reaction was monitored by TLC (7:3, hexanes/ethyl acetate. *R*_f_ (**Dha**) = 0.75) and, once completed (5 min), the solution was dried under vacuum. The crude mixture was purified by column chromatography on silica gel (hexanes/ethyl acetate, 7:3) to afford compound **3a**–**D** as a sticky foam (27 mg, 0.07 mmol, 73% yield, 94% D). [α]_D_^20^ +85.4 (c 1.0, CHCl_3_). HRMS (ESI) *m*/*z*: [M + H]^+^ calcd for C_16_H_23_DNO_9_ 375.1508; Found 375.1504. ^1^H NMR (CDCl_3_, 400 MHz): δ 4.43 (dd, 0.06H, *J* = 11.6, 5.2 Hz, H^3α^), 4.16–4.28 (m, 4H, 2CH_2_^b^), 3.66 (dd, 1H, *J* = 8.3, 5.9 Hz, H^a^), 3.48 (s, 3H, OMe^7^), 2.66 (dd, 1H, *J* = 14.4, 8.3 Hz, H^β^), 2.25 (dd, 1H, *J* = 14.4, 5.9 Hz, H^β^), 1.65 (s, 3H, CH_3_^7a^), 1.61 (s, 3H, CH_3_^7^), 1.24–1.31 (m, 6H, 2CH_2_^c^). ^13^C{^1^H} NMR (CDCl_3_, 100 MHz): δ 171.1, 168.6, 168.0, 159.2 (4CO), 108.3 (C^7^), 101.7 (C^7a^), 62.2 (C^b^), 62.1 (C^b^), 58.8 (t, J= 21.6 Hz, C^3α^), 51.7 (OMe^7^), 48.8 (C^a^), 30.1 (C^β^), 22.0 (CH_3_^7a^), 16.6 (CH_3_^7^), 14.1 (C^c^), 14.1 (C^c^).

### General Procedure for Hydrolysis of Michael Adducts

Compound **3a** (15 mg, 0.04 mmol), **3a**–**D** (15 mg, 0.04 mmol), or **3b** (15 mg, 0.03 mmol) was suspended in a 6 M HCl aqueous solution (3.0 mL), and the reaction mixture was stirred at 60 °C in an oil bath for 16 h. The solvent was then removed under vacuum, the crude mixture was dissolved in water (5 mL), washed with ethyl acetate (5 mL), and purified by solid phase extraction in a C18 cartridge to afford **4a** (5.5 mg, 0.04 mmol, 93% yield), **4a**–**D** (5.7 mg, 0.04 mmol, 96% yield, 88% D), or **4b** (6 mg, 0.03 mmol, 95% yield).

#### l-Glutamic Acid Hydrochloride (**4a**)

Following the general procedure for hydrolysis. Yield after SPE cartridge: 5.5 mg, 93%. White solid. [α]_D_^20^ +32.7 (c 1.0, 6 M HCl). HRMS (ESI) *m*/*z*: [M + H]^+^ calcd for C_5_H_10_NO_4_ 148.0604; Found 148.0606. ^1^H NMR (D_2_O, 400 MHz): δ 4.01 (t, 1H, *J* = 6.5 Hz, H^α^), 2.57–2.62 (m, 2H, H^γ^), 2.11–2.25 (m, 2H, H^β^). ^13^C{^1^H} NMR (D_2_O, 100 MHz): δ 176.4, 172.2 (2CO), 52.6 (H^α^), 29.5 (C^γ^), 25.0 (C^β^).

#### l-Glutamic-2-*d* Acid Hydrochloride (**4a**–**D**)

Following the general procedure for hydrolysis. Yield after SPE cartridge: 5.7 mg, 96%. White solid. [α]_D_^20^ +30.2 (c 1.0, 6 M HCl). HRMS (ESI) *m*/*z*: [M – H]^−^ calcd for C_5_H_7_DNO_4_ 147.0522; Found 147.0519. ^1^H NMR (D_2_O, 400 MHz): δ 4.08 (t, 1H, *J* = 6.6 Hz, 0.12H^α^), 2.60–2.66 (m, 2H, H^γ^), 2.15–2.28 (m, 2H, H^β^). ^13^C{^1^H} NMR (D_2_O, 100 MHz): δ 176.4, 172.0 (2CO), 52.1 (t, *J* = 22.5 Hz, H^α^), 29.5 (C^γ^), 24.9 (C^β^).

#### (2*R*,4*S*)-2,4-Diamino-2-(hydroxymethyl)pentanedioic Acid Dihydrochloride (**4b**)

Following the general procedure for hydrolysis. Yield after SPE cartridge: 6.0 mg, 95%. White solid. [α]_D_^20^ +10.6 (c 1.0, 6 M HCl). HRMS (ESI) *m*/*z*: [M + H]^+^ calcd for C_6_H_13_N_2_O_5_ 193.0819; Found 193.0822. ^1^H NMR (D_2_O, 400 MHz): δ 4.31 (t, 1H, *J* = 9.4 Hz, H^α^), 3.87 (d, 1H, *J* = 11.6 Hz, H^δ^), 3.82 (d, 1H, *J* = 11.6 Hz, H^δ^), 2.81 (dd, 1H, *J* = 13.8, 9.4 Hz, H^β^), 2.39 (dd, 1H, *J* = 13.8, 9.4 Hz, H^β^). ^13^C{^1^H} NMR (D_2_O, 100 MHz): δ 174.5, 172.4 (2CO), 65.5 (C^γ^), 62.7 (C^δ^), 50.0 (C^α^), 32.0 (C^β^).

### Photoredox Catalytic 1,4-Additions to Chiral Dha **1** Followed by Hydrolysis to Obtain Carbon-β-Substituted UAAs

#### General Procedure for Photoredox Catalytic 1,4-Additions

Chiral bicyclic Dha **1** (21 mg, 0.1 mmol, 1.0 equiv), the corresponding carboxylic acid **2c**–**n** (0.12 mmol, 1.2 equiv), Cs_2_CO_3_ (39 mg, 0.15 mmol, 1.5 equiv), and 4CzIPN (4 mg, 0.005 mmol, 0.05 equiv) were added in sample vials. The tube was evacuated and back-filled with N_2_ (three times). Then, anhydrous DMF (1 mL, final concentration 0.1 M) was added using a syringe. The solution was then stirred at room temperature under the irradiation of Blue LEDs for 2–16 h. Once completed, 1 mL of water was added and extracted with ethyl acetate. The combined organic layer was dried over anhydrous Na_2_SO_4_, filtered, and evaporated under vacuum. The crude mixture was purified by column chromatography (hexanes/ethyl acetate) on silica gel to afford desired products. Light-promoted reactions have been carried out in a SynLED Parallel Photoreactor (available from Sigma-Aldrich). Bottom-lit LEDs (465–470 nm) across a 4 × 4 reaction block array provide consistent light intensity (130–140 lm) and an angle (45°). A built-in cooling fan provides consistent temperature to each parallel reaction. Uses 1–2 dram scintillation vials or microwave vials (O.D. of 1.7 cm or less). A power supply is a wall plug power supply 700 mA 12 W. Wheaton sample vials (clear borosilicate glass vial).

#### Procedure to Scale Up the Photoredox Catalytic 1,4-Additions

Chiral bicyclic Dha **1** (200 mg, 0.93 mmol, 1.0 equiv), 2-(phenylselanyl)acetic acid **2k** (240 mg, 1.12 mmol, 1.2 equiv), Cs_2_CO_3_ (371 mg, 1.40 mmol, 1.5 equiv), and 4CzIPN (38 mg, 0.046 mmol, 0.05 equiv) were added in a 50 mL flask. The vessel was evacuated and refilled with N_2_ (×3). Then, anhydrous DMF (10 mL, final concentration 0.1 M) was added using a syringe. The solution was then stirred at room temperature under the irradiation of Blue LEDs for 16 h. Once completed, 10 mL of water was added and extracted with ethyl acetate. The combined organic layer was dried over anhydrous Na_2_SO_4_ and dried under vacuum. The crude mixture was purified by column chromatography (hexanes/ethyl acetate, 7:3) on silica gel to afford **3k** (205 mg, 0.53 mmol, 57%). Light-promoted reactions on a larger scale have been carried out by irradiating with the blue light of an RGB LED of 50 W. Ce RoHS EMC IP65 50 W at 15 cm from the flask on the stirring plate in a photochemical cabinet.

### General Procedure for Deuterated Compounds

Chiral bicyclic Dha **1** (21 mg, 0.1 mmol, 1.0 equiv), deuterated carboxylic acids (0.12 mmol, 1.2 equiv), Cs_2_CO_3_ (39 mg, 0.15 mmol, 1.5 equiv), and 4CzIPN (4 mg, 0.005 mmol, 0.05 equiv) were added in sample vials. The tube was evacuated and back-filled with N_2_ (three times). Then, anhydrous DMF (1 mL, final concentration 0.1 M) and D_2_O (50 μL) were added using a syringe. The solution was then stirred at room temperature under the irradiation of Blue LEDs (SynLED parallel photoreactor) for 2–16 h. Once completed, 1 mL of D_2_O was added and extracted with ethyl acetate. The combined organic layer was dried over anhydrous Na_2_SO_4_, filtered, and evaporated under vacuum. The crude mixture was purified by column chromatography (hexanes/ethyl acetate) on silica gel to afford desired products.

### General Procedure for Hydrolysis of Adducts

Compounds were suspended in a 4 M HCl aqueous solution and the reaction mixture was stirred at 60 °C in an oil bath for 16 h. The solvent was then removed under vacuum, the crude mixture was dissolved in water (5 mL), washed with ethyl acetate (5 mL), and purified by solid phase extraction in a C18 cartridge to afford desired products.

### Aminolysis of Adduct **3k** with HCl·Phe-OBn

Compound **3k** (64 mg, 0.16 mmol), the corresponding amino ester hydrochloride (H-Phe-OBn·HCl, 73 mg, 0.25 mmol, 1.5 equiv), and sodium 2-ethylhexanoate (69 mg, 0.42 mmol, 2.5 equiv) were charged in an oven-dried Schlenk flask and subjected to a vacuum/N_2_ cycle (×3) to remove possible moisture. Under a N_2_ atmosphere, dry THF (8 mL, 50 mL mmol^–1^) was added to the flask by a syringe. The solution was stirred at room temperature for 24 h. After that time, brine and ethyl acetate were added to the solution. Layers were separated and the aqueous layer was back-extracted with more ethyl acetate. The crude mixture was purified by column chromatography (hexanes/ethyl acetate 7:3) on silica gel to afford the desired product **5k**.

#### *tert*-Butyl-(2-((3*S*,7*S*,7a*R*)-7-methoxy-7,7a-dimethyl-2,5-dioxotetrahydro-5*H*-oxazolo[4,3-*b*]oxazol-3-yl)ethyl)carbamate (**3c**)

Following the general procedure. Yield after column chromatography (hexanes/ethyl acetate, 8:2): 30 mg, 88%. White solid. Mp: 116–119. [α]_D_^20^ +34.5 (c 1.0, CHCl_3_). HRMS (ESI) *m*/*z*: [M + H]^+^ calcd for C_15_H_24_N_2_O_7_ 345.1656; Found 345.1652. ^1^H NMR (CDCl_3_, 400 MHz): δ 5.13 (br s, 1H, H^NH^), 4.37 (dd, 1H, *J* = 12.0, 4.2 Hz, H^3α^), 3.56–3.60 (m, 1H, CH_2_^γ^), 3.51 (s, 3H, OMe^7^), 3.15–3.23 (m, 1H, CH_2_^γ^), 2.22–2.29 (m, 1H, H^β^), 1.76–1.84 (m, 1H, H^β^), 1.63 (s, 6H, CH_3_^7a^, CH_3_^7^), 1.44 (s, 9H, NHBoc). ^13^C{^1^H} NMR (CDCl_3_, 100 MHz): δ 172.0, 159.6, 156.0 (3CO), 108.9 (C^7^), 101.3 (C^7a^), 79.7 (C(CH_3_)_3_), 58.7 (C^3α^), 51.9 (OMe^7^), 37.6 (C^γ^), 31.4 (C^β^), 28.5 (C(CH_3_)_3_), 22.4 (CH_3_^7a^), 16.8 (CH_3_^7^).

#### *tert*-Butyl-(2-((3*S*,7*S*,7a*R*)-7-methoxy-7,7a-dimethyl-2,5-dioxotetrahydro-5*H*-oxazolo[4,3-*b*]oxazol-3-yl-3-*d*)ethyl)carbamate (**3c**–**D**)

Following the general procedure for deuterated compounds. Yield after column chromatography (hexanes/ethyl acetate, 8:2): 28 mg, 82% [93% deuterated]. White solid. Mp: 118–118. [α]_D_^20^ +36.4 (c 1.0, CHCl_3_). HRMS (ESI) *m*/*z*: [M + H]^+^ calcd for C_15_H_23_DN_2_O_7_Na 368.1544; Found 368.1549. ^1^H NMR (CDCl_3_, 400 MHz): δ 5.12 (br s, 1H, H^NH^), 4.37 (dd, 0.07H, *J* = 11.4, 4.2 Hz, H^3α^), 3.56–3.60 (m, 1H, CH_2_^γ^), 3.51 (s, 3H, OMe^7^), 3.13–3.25 (m, 1H, CH_2_^γ^), 2.20–2.29 (m, 1H, H^β^), 1.76–1.84 (m, 1H, H^β^), 1.63 (s, 6H, CH_3_^7a^, CH_3_^7^), 1.44 (s, 9H, NHBoc). ^13^C{^1^H} NMR (CDCl_3_, 100 MHz): δ 172.0, 159.6, 155.9 (3CO), 108.9 (C^7^), 101.3 (C^7a^), 79.7 (C(CH_3_)_3_), 51.9 (OMe^7^), 37.5 (C^γ^), 31.2 (C^β^), 28.5 (C(CH_3_)_3_), 22.4 (CH_3_^7a^), 16.8 (CH_3_^7^). *C^3α^ is not observed.

#### (3*S*,7*S*,7a*R*)-7-Methoxy-7,7a-dimethyl-3-phenethyldihydro-5*H*-oxazolo[4,3-*b*]oxazole-2,5(3*H*)-dione (**3d**)

Following the general procedure. Yield after column chromatography (hexanes/ethyl acetate, 8:2): 22 mg, 73%. Light yellow solid. Mp: 52–55. [α]_D_^20^ +52.2 (c 1.0, CHCl_3_). HRMS (ESI) *m*/*z*: [M + Na]^+^ calcd for C_16_H_19_NNaO_5_ 328.1155; Found 328.1150. ^1^H NMR (CDCl_3_, 400 MHz): δ 7.20–7.34 (m, 5H, H^Ar^), 4.32 (dd, 1H, *J* = 10.9, 4.8 Hz, H^3α^), 3.51 (s, 3H, OMe^7^), 2.82–2.97 (m, 2H, CH_2_^γ^), 2.27–2.36 (m, 1H, H^β^), 1.95–2.06 (m, 1H, H^β^), 1.66 (s, 3H, CH_3_^7a^), 1.63 (s, 3H, CH_3_^7^). ^13^C{^1^H} NMR (CDCl_3_, 100 MHz): δ 172.1, 159.5 (2CO), 140.0 (C^*Ar^), 128.8 (4C^Ar^), 126.6 (C^Ar^), 108.2 (C^7^), 101.6 (C^7a^), 60.5 (C^3α^), 51.7 (OMe^7^), 33.7 (C^β^), 32.6 (C ^γ^), 22.2 (CH_3_^7a^), 16.7 (CH_3_^7^).

#### (3*S*,7*S*,7a*R*)-3-(Cyclohexylmethyl)-7-methoxy-7,7a-dimethyldihydro-5*H*-oxazolo[4,3-*b*]oxazole-2,5(3*H*)-dione (**3e**)

Following the general procedure. Yield after column chromatography (hexanes/ethyl acetate, 8:2): 25 mg, 85%. Light yellow solid. Mp: 60–63. [α]_D_^20^ +68.5 (c 1.0, CHCl_3_). HRMS (ESI) *m*/*z*: [M + Na]^+^ calcd for C_15_H_23_NNaO_5_ 320.1468; Found 320.1474. ^1^H NMR (CDCl_3_, 400 MHz): δ 4.41 (dd, 1H, *J* = 11.1, 4.3 Hz, H^3α^), 3.50 (s, 3H, OMe^7^), 1.96–2.03 (m, 1H, 1CH_2_^cyclo^), 1.68–1.86 (m, 5H, 4CH_2_^cyclo^, 1H^β^), 1.64 (s, 3H, CH_3_^7a^), 1.62 (s, 3H, CH_3_^7^), 1.54–1.65 (m, 2H, CH^cyclo^, 1H^β^), 1.14–1.30 (m, 3H, 3CH_2_^cyclo^), 0.56–1.09 (m, 2H, 3CH_2_^cyclo^). ^13^C{^1^H} NMR (CDCl_3_, 100 MHz): δ 173.0, 159.6 (2CO), 107.9 (C^7^), 101.6 (C^7a^), 59.2 (C^3α^), 51.6 (OMe^7^), 38.9 (C^β^), 34.9 (CH^cyclo^), 33.6 (CH_2_^cyclo^), 31.8 (CH_2_^cyclo^), 26.5 (CH_2_^cyclo^), 26.3 (CH_2_^cyclo^), 26.0 (CH_2_^cyclo^), 22.1 (CH_3_^7a^), 16.6 (CH_3_^7^).

#### (3*S*,7*S*,7a*R*)-7-Methoxy-7,7a-dimethyl-3-((1-methylcyclohexyl)methyl)dihydro-5*H*-oxazolo[4,3-*b*]oxazole-2,5(3*H*)-dione (**3f**)

Following the general procedure. Yield after column chromatography (hexanes/ethyl acetate, 8:2): 25 mg, 82%. Light yellow solid. Mp: 76–79. [α]_D_^20^ +67.1 (c 1.0, CHCl_3_). HRMS (ESI) *m*/*z*: [M + Na]^+^ calcd for C_16_H_25_NNaO_5_ 334.1625; Found 334.1620. ^1^H NMR (CDCl_3_, 400 MHz): δ 4.45 (dd, 1H, *J* = 10.9, 2.5 Hz, H^3α^), 3.49 (s, 3H, OMe^7^), 1.88 (dd, 1H, *J* = 14.5, 2.5 Hz, H^β^), 1.72 (dd, 1H, *J* = 14.5, 10.9 Hz, H^β^), 1.66 (s, 3H, CH_3_^7a^), 1.62 (s, 3H, CH_3_^7^), 1.38–1.52 (m, 10H, H^cyclo^), 1.07 (s, 3H, CH_3_^cyclo^). ^13^C{^1^H} NMR (CDCl_3_, 100 MHz): δ 173.7, 159.4 (2CO), 107.6 (C^7^), 101.9 (C^7a^), 58.1 (C^3α^), 51.6 (OMe^7^), 43.2 (C^β^), 37.7 (CH_2_^cyclo^), 37.6 (CH_2_^cyclo^), 33.6 (C*^cyclo^), 26.3 (CH_2_^cyclo^), 24.4 (CH_3_^cyclo^), 22.0 (2CH_2_^cyclo^), 22.0 (CH_3_^7a^), 16.6 (CH_3_^7^).

#### (3*S*,7*S*,7a*R*)-7-Methoxy-7,7a-dimethyl-3-neopentyldihydro-5*H*-oxazolo[4,3-*b*]oxazole-2,5(3*H*)-dione (**3g**)

Following the general procedure. Yield after column chromatography (hexanes/ethyl acetate, 8:2): 23 mg, 86%. Yellow solid. Mp: 91–93. [α]_D_^20^ +79.5 (c 1.0, CHCl_3_). HRMS (ESI) *m*/*z*: [M + Na]^+^ calcd for C_13_H_21_NNaO_5_ 294.1312; Found 294.1304. ^1^H NMR (CDCl_3_, 400 MHz): δ 4.42 (dd, 1H, *J* = 11.1, 2.6 Hz, H^3α^), 3.49 (s, 3H, OMe^7^), 1.87 (dd, 1H, *J* = 14.5, 2.6 Hz, H^β^), 1.63–1.68 (m, 1H, H^β^), 1.65 (s, 3H, CH_3_^7a^), 1.62 (s, 3H, CH_3_^7^), 1.06 (s, 9H, (CH_3_)_3_). ^13^C{^1^H} NMR (CDCl_3_, 100 MHz): δ 173.5, 159.4 (2CO), 107.6 (C^7^), 101.8 (C^7a^), 58.8 (C^3α^), 51.6 (OMe^7^), 44.7 (C^β^), 31.2 (C(CH_3_)_3_), 29.3 (3C(CH_3_)_3_), 22.0 (CH_3_^7a^), 16.6 (CH_3_^7^).

#### (3*S*,7*S*,7a*R*)-3-*iso-*Butyl-7-methoxy-7,7a-dimethyldihydro-5*H*-oxazolo[4,3-*b*]oxazole-2,5(3*H*)-dione (**3h**)

Following the general procedure. Yield after column chromatography (hexanes/ethyl acetate, 8:2): 20 mg, 83%. Sticky foam. [α]_D_^20^ +52.5 (c 1.0, CHCl_3_). HRMS (ESI) *m*/*z*: [M +Na]^+^ calcd for C_12_H_19_NNaO_5_ 280.1155; Found 280.1146. ^1^H NMR (CDCl_3_, 400 MHz): δ 4.37 (dd, 1H, *J* = 11.5, 4.5 Hz, H^3α^), 3.50 (s, 3H, OMe^7^), 1.88–1.98 (m, 1H, CH(CH_3_)_2_), 1.79 (ddd, 1H, *J* = 13.4, 8.8, 4.5 Hz, H^β^), 1.64–1.69 (m, 1H, H^β^), 1.64 (s, 3H, CH_3_^7a^), 1.62 (s, 3H, CH_3_^7^), 1.06 (d, 3H, *J* = 6.5, 1CH(CH_3_)_2_), 1.03 (d, 3H, *J* = 6.7, 1CH(CH_3_)_2_). ^13^C{^1^H} NMR (CDCl_3_, 100 MHz): δ 172.8, 159.6 (2CO), 107.9 (C^7^), 101.6 (C^7a^), 59.7 (C^3α^), 51.7 (OMe^7^), 40.2 (C^β^), 25.8 (CH(CH_3_)_2_), 22.9 (1CH(CH_3_)_2_), 22.1 (CH_3_^7a^), 21.3 (1CH(CH_3_)_2_), 16.6 (CH_3_^7^).

#### (3*S*,7*S*,7a*R*)-7-Methoxy-7,7a-dimethyl-3-(2-phenoxyethyl)dihydro-5*H*-oxazolo[4,3-*b*]oxazole-2,5(3*H*)-dione (**3i**)

Following the general procedure. Yield after column chromatography (hexanes/ethyl acetate, 8:2): 27 mg, 85%. Sticky foam. [α]_D_^20^ +32.2 (c 1.0, CHCl_3_). HRMS (ESI) *m*/*z*: [M + Na]^+^ calcd for C_16_H_19_NNaO_6_ 344.1105; Found 344.1098. ^1^H NMR (CDCl_3_, 400 MHz): δ 7.28–7.31 (m, 2H, CH^Ar^), 6.92–7.00 (m, 3H, CH^Ar^), 4.61 (dd, 1H, *J* = 10.4, 5.0 Hz, H^3α^), 4.15–4.26 (m, 2H, CH_2_^γ^), 3.50 (s, 3H, OMe^7^), 2.44–2.54 (m, 1H, H^β^), 2.12–2.21 (m, 1H, H^β^), 1.66 (s, 3H, CH_3_^7a^), 1.66 (s, 3H, CH_3_^7^). ^13^C{^1^H} NMR (CDCl_3_, 100 MHz): δ 171.9, 159.2 (2CO), 129.7, 129.7, 121.4, 115.0, 115.0 (5C^Ar^), 108.2 (C^7^), 101.6 (C^7a^), 63.9 (C^γ^), 57.9 (C^3α^), 51.8 (OMe^7^), 31.7 (C^β^), 29.9 (C*^Ar^), 22.2 (CH_3_^7a^), 16.7 (CH_3_^7^).

#### (3*S*,7*S*,7a*R*)-7-Methoxy-7,7a-dimethyl-3-(2-(phenylthio)ethyl)dihydro-5*H*-oxazolo[4,3-*b*]oxazole-2,5(3*H*)-dione (**3j**)

Following the general procedure. Yield after column chromatography (hexanes/ethyl acetate, 8:2): 27 mg, 81%. White solid. Mp: 108–110. [α]_D_^20^ +48.2 (c 1.0, CHCl_3_). HRMS (ESI) *m*/*z*: [M + Na]^+^ calcd for C_16_H_19_NNaO_5_S 360.0876; Found 360.0871. ^1^H NMR (CDCl_3_, 400 MHz): δ 7.13–7.3 (m, 5H, CH^Ar^), 4.42 (dd, 1H, *J* = 10.8, 4.8 Hz, H^3α^), 3.44 (s, 3H, OMe^7^), 3.05–3.14 (m, 1H, CH_2_^γ^), 2.95–3.04 (m, 1H, CH_2_^γ^), 2.13–2.23 (m, 1H, H^β^), 1.83–1.93 (m, 1H, H^β^), 1.55 (s, 3H, CH_3_^7^), 1.50 (s, 3H, CH_3_^7a^).^13^C{^1^H} NMR (CDCl_3_, 100 MHz): δ 171.6, 159.3 (2CO), 134.9 (C*^Ar^), 130.6, 130.6, 129.3, 129.3, 127.0 (5C^Ar^), 108.3 (C^7^), 101.6 (C^7a^), 59.8 (C^3α^), 51.8 (OMe^7^), 31.5 (C^β^), 30.8 (C^γ^), 22.1 (CH_3_^7a^), 16.7 (CH_3_^7^).

#### (3*S*,7*S*,7a*R*)-7-Methoxy-7,7a-dimethyl-3-(2-(phenylselanyl)ethyl)dihydro-5*H*-oxazolo[4,3-*b*]oxazole-2,5(3*H*)-dione (**3k**)

Following the general procedure. Yield after column chromatography (hexanes/ethyl acetate, 8:2): 33 mg, 87%. Sticky foam, but with time and solvents we got monocrystals. [α]_D_^20^ +41.2 (c 1.0, CHCl_3_). HRMS (ESI) *m*/*z*: [M + Na]^+^ calcd for C_16_H_19_NNaO_5_Se 408.0321; Found 408.0320. ^1^H NMR (CDCl_3_, 400 MHz): δ 7.43–7.50 (m, 2H, CH^Ar^), 7.16–7.22 (m, 3H, CH^Ar^), 4.41 (dd, 1H, *J* = 10.9, 4.9 Hz, H^3α^), 3.43 (s, 3H, OMe^7^), 3.01–3.08 (m, 1H, CH_2_^γ^), 2.89–2.97 (m, 1H, CH_2_^γ^), 2.17–2.28 (m, 1H, H^β^), 1.88–1.99 (m, 1H, H^β^), 1.54 (s, 3H, CH_3_^7^), 1.47 (s, 3H, CH_3_^7a^). ^13^C{^1^H} NMR (CDCl_3_, 100 MHz): δ 171.6, 159.4 (2CO), 128.9 (C*^Ar^), 133.7, 133.7, 129.4, 129.4, 127.7, 115.0 (5C^Ar^), 108.3 (C^7^), 101.5 (C^7a^), 60.7 (C^3α^), 51.8 (OMe^7^), 32.4 (C^β^), 23.7 (C^γ^), 22.1 (CH_3_^7a^), 16.7 (CH_3_^7^).

#### *tert*-Butyl-(1-(((3*S*,7*S*,7a*R*)-7-methoxy-7,7a-dimethyl-2,5-dioxotetrahydro-5*H*-oxazolo[4,3-*b*]oxazol-3-yl)methyl)cyclohexyl)carbamate (**3l**)

Following the general procedure. Yield after column chromatography (hexanes/ethyl acetate, 8:2): 32 mg, 78%. Light yellow solid. Mp: 84–87. [α]_D_^20^ +145.7 (c 1.0, CHCl_3_). HRMS (ESI) *m*/*z*: [M +Na]^+^ calcd for C_20_H_32_N_2_NaO_7_ 435.2101; Found 435.2102. ^1^H NMR (CDCl_3_, 400 MHz): δ 4.56 (bs, 1H, NHBoc), 4.41 (d, 1H, *J* = 11.2 Hz, H^3α^), 3.48 (s, 3H, OMe^7^), 2.41–2.51 (m, 1H, H^β^), 2.24–2.36 (m, 1H, H^cycle^), 1.97–2.09 (m, 2H, H^β^, H^cycle^), 1.64 (s, 3H, CH_3_^7a^), 1.61 (s, 3H, CH_3_^7^), 1.53–1.64 (m, 2H, H^cycle^), 1.34–1.50 (m, 5H, H^cycle^), 1.40 (s, 9H, 1C(CH_3_)_3_), 1.22–1.29 (m, 1H, H^cycle^). ^13^C{^1^H} NMR (CDCl_3_, 100 MHz): δ 173.2, 159.4 (2CO), 154.5 (C(CH_3_)_3_), 108.0 (C^7^), 102.0 (C^7a^), 57.2 (C^3α^), 53.5 (CNHBoc), 51.5 (OMe^7^), 38.1 (C^β^), 34.9 (2C^cycle^), 28.1, 28.4, 28.5 (C(CH_3_)_3_), 25.8 (C^cycle^), 21.9 (CH_3_^7a^), 21.5 (C^cycle^), 21.3 (C^cycle^), 16.5 (CH_3_^7^).

#### *tert*-Butyl-(1-((3*S*,7*S*,7a*R*)-7-methoxy-7,7a-dimethyl-2,5-dioxotetrahydro-5*H*-oxazolo[4,3-*b*]oxazol-3-yl)-2-methylpropan-2-yl)carbamate (**3m**)

Following the general procedure. Yield after column chromatography (hexanes/ethyl acetate, 8:2): 28 mg, 76%. Light yellow solid. Mp: 78–81. [α]_D_^20^ +43.8 (c 1.0, CHCl_3_). HRMS (ESI) *m*/*z*: [M +Na]^+^ calcd for C_17_H_28_N_2_NaO_7_ 395.1789; Found 395.1793. ^1^H NMR (CDCl_3_, 400 MHz): δ 4.71 (br s, 1H, NHBoc), 4.37 (d, 1H, *J* = 11.5 Hz, H^3α^), 3.48 (s, 3H, OMe^7^), 2.45–2.57 (m, 1H, H^β^), 2.01–2.10 (m, 1H, H^β^), 1.64 (s, 3H, CH_3_^7a^), 1.62 (s, 3H, CH_3_^7^), 1.43 (s, 3H, 1C(CH_3_)_2_), 1.40 (s, 9H, 1C(CH_3_)_3_), 1.35 (s, 3H, 1C(CH_3_)_2_). ^13^C{^1^H} NMR (CDCl_3_, 100 MHz): δ 172.6, 159.1 (2CO), 154.2 (C(CH_3_)_3_), 107.7 (C^7^), 101.7 (C^7a^), 57.8 (C^3α^), 51.2 (OMe^7^), 38.1 (C^β^), 28.2 (4C, 3C(CH_3_)_3_, 1CH(CH_3_)_2_), 27.6 (1CH(CH_3_)_2_), 21.6 (CH_3_^7a^), 16.2 (CH_3_^7^).

#### (3*S*,7*S*,7a*R*)-3-(hex-5-yn-1-yl)-7-Methoxy-7,7a-dimethyldihydro-5*H*-oxazolo[4,3-*b*]oxazole-2,5(3*H*)-dione (**3n**)

Following the general procedure. Yield after column chromatography (hexanes/ethyl acetate, 8:2): 16 mg, 57%. Light yellow oil. [α]_D_^20^ +67.2 (c 1.0, CHCl_3_). HRMS (ESI) *m*/*z*: [M + H]^+^ calcd for C_14_H_20_NO_5_ 282.13360; Found 282.13428. ^1^H NMR (CDCl_3_, 400 MHz): δ 4.27 (dd, 1H, *J* = 9.7, 4.9 Hz, H^3α^), 3.50 (s, 3H, OMe^7^), 2.20–2.26 (m, 2H, CH_2_), 2.00–2.08 (m, 1H, 1H^β^), 1.95–1.98 (m, 1H, CH^alkyne^), 2.61–1.78 (m, 5H, 2CH_2_, 1H^β^), 1.64 (s, 3H, CH_3_^7a^), 1.62 (s, 3H, CH_3_^7^). ^13^C{^1^H} NMR (CDCl_3_, 100 MHz): δ 172.3, 159.5 (2CO), 108.2 (C^7^), 101.6 (C^7a^), 83.9 (C^alkyne^), 68.9 (CH^alkyne^), 60.9 (C^3α^), 51.7 (OMe^7^), 31.1 (C^β^), 27.5 (CH_2_), 25.6 (CH_2_), 22.2 (CH_3_^7a^), 18.3 (CH_2_), 16.7 (CH_3_^7^).

#### (*S*)-2,4-Diaminobutanoic Acid Dihydrochloride (**4c**)

Following the general procedure for hydrolysis. Yield: 93%. [α]_D_^20^ +14.2 (c 1.0, 6 M HCl). HRMS (ESI) *m*/*z*: [M – H]^−^ calcd for C_4_H_9_N_2_O_2_ 117.0670; Found 117.0668. ^1^H NMR (D_2_O, 400 MHz): δ 3.99 (t, 1H, *J* = 6.5 Hz, H^α^), 3.14–3.30 (m, 2H, H^γ^), 2.18–2.31 (m, 2H, H^β^). ^13^C{^1^H} NMR (D_2_O, 100 MHz): δ 172.0 (CO), 51.4 (C^α^), 36.2 (C^γ^), 27.8 (C^β^).

#### (*S*)-2,4-Diaminobutanoic-2-*d* Acid Dihydrochloride (**4c**–**D**)

Following the general procedure for hydrolysis. Yield: 85% [89% deuterated]. [α]_D_^20^ +13.8 (c 1.0, 6 M HCl). HRMS (ESI) *m*/*z*: [M – H]^+^ calcd for C_4_H_10_DN_2_O_2_ 120.0883; Found 120.0877. ^1^H NMR (D_2_O, 400 MHz): δ 3.90–3.93 (m, 0.11H, H^α^), 3.14–3.22 (m, 2H, H^γ^), 2.17–2.20 (m, 2H, H^β^). ^13^C{^1^H} NMR (D_2_O, 100 MHz): δ 173.0 (CO), 36.4 (C^γ^), 27.9 (C^β^). C^α^ is not observed.

#### (*S*)-2-Amino-4-(phenylselanyl)butanoic Acid Hydrochloride (**4k**)

Following the general procedure for hydrolysis. Yield: 93% (27 mg). [α]_D_^20^ −2.3 (c 1.0, 6 M HCl). HRMS (ESI) *m*/*z*: [M + H]^+^ calcd for C_10_H_14_NO_2_Se 260.01843; Found 260.01715. ^1^H NMR (D_2_O, 400 MHz): δ 7.55–7.60 (m, H, CH^Ar^), 7.32–7.38 (m, H, CH^Ar^), 3.68 (t, 1H, *J* = 6.3 Hz, H^α^), 2.99 (t, 2H, *J* = 7.9 Hz, H ^γ^), 2.03–2.19 (m, 2H, H^β^). ^13^C{^1^H} NMR (D_2_O, 100 MHz): δ 170.1 (CO), 132.4 (2C^Ar^), 129.4 (3C^Ar^), 129.1(C^*Ar^), 54.9 (C^α^), 32.1 (C^β^), 22.0 (C^γ^).

#### Benzyl-((*S*)-2-((4*R*,5*S*)-4-hydroxy-5-methoxy-4,5-dimethyl-2-oxooxazolidin-3-yl)-4-(phenylselanyl)butanoyl)-l-phenylalaninate (**5k**)

Following the general procedure for aminolysis with amino ester hydrochlorides. Yield after column chromatography (hexanes/ethyl acetate, 7:3): 78 mg, 74%. Sticky foam. [α]_D_^20^ +18.4 (c 1.0, CHCl_3_). HRMS (ESI) *m*/*z*: [M + Na]^+^ calcd for C_32_H_36_N_2_NaO_7_Se 663.1585; Found 663.1609. ^1^H NMR (CDCl_3_, 400 MHz): δ 7.38–7.42 (m, 2H, CH^Ar^), 7.27–7.30 (m, 2H, CH^Ar^), 7.20–7.24 (m, 2H, CH^Ar^), 7.16–7.19 (m, 3H, CH^Ar^), 7.11–7.16 (m, 3H, CH^Ar^), 6.93–6.97 (m, 2H, CH^Ar^), 6.82 (d, 1H, *J* = 7.9 Hz, NH), 5.08 (d, 1H, *J* = 12.0 Hz, 1CH_2_^OBn^), 5.01 (d, 1H, *J* = 12.0 Hz, 1CH_2_^OBn^), 4.81 (ddd, 1H, *J* = 7.9, 5.5, 5.5 Hz H^αPhe^), 4.34 (dd, 1H, *J* = 9.5, 5.9 Hz, H^3α^), 4.28 (br s, 1H, OH), 3.12 (s, 3H, OMe^7^), 3.01 (t, 2H, *J* = 5.5 Hz, CH_2_^βPhe^), 2.88–2.97 (m, 1H, CH_2_^γ^), 2.75–2.83 (m, 1H, CH_2_^γ^), 2.36–2.48 (m, 1H, H^β^), 2.20–2.31 (m, 1H, H^β^), 1.44 (s, 3H, CH_3_^7^), 1.36 (s, 3H, CH_3_^7a^). ^13^C{^1^H} NMR (CDCl_3_, 100 MHz): δ 170.8, 170.8, 155.9 (2CO), 135.3, 135.1, 129.9 (3C*^Ar^), 133.2–127.3 (15C^Ar^), 107.9 (C^7^), 91.0 (C^7a^), 67.5 (CH_2_^OBn^), 56.5 (C^3α^), 53.8 (C^αPhe^), 50.6 (OMe^7^), 38.0 (C^βPhe^), 29.6 (C^β^), 24.9 (C^γ^), 20.4 (CH_3_^7a^), 14.1 (CH_3_^7^).

#### ((*S*)-2-Amino-4-(phenylselanyl)butanoyl)-l-phenylalanine (**6k**)

Following the general procedure for hydrolysis. Yield: 95%. [α]_D_^20^ −12.3 (c 1.0, 6 M HCl). HRMS (ESI) *m*/*z*: [M – H]^+^ calcd for C_19_H_23_N_2_O_3_Se 407.0874; Found 407.0878. ^1^H NMR (D_2_O, 400 MHz): δ 7.20–7.60 (m, 10H, CH^Ar^), 3.86 (dd, 1H, *J* = 7.9, 5.2 Hz, H^αPhe^), 3.69 (t, 1H, *J* = 6.7 Hz, H^α^), 3.16 (dd, 1H, *J* = 14.5, 5.2 Hz, 1H^βPhe^), 3.00 (dd, 1H, *J* = 14.5, 7.9 Hz, 1H ^βPhe^), 2.90 (t, 2H, *J* = 7.8 Hz, H ^γ^), 1.80–2.15 (m, 2H, H^β^). ^13^C{^1^H} NMR (D_2_O, 100 MHz): δ 174.2, 174.2 (2CO), 135.1 (C^*Phe^), 132.5 (2C^Ar^), 132.2 (C^*Se^), 129.4, 129.3, 129.2, 129.0 128.6, 128.4, 127.6, 127.5 (8C^Ar^), 56.0 (C^αPhe^), 54.6 (C^α^), 36.4 (C^βPhe^), 31.2 (C^β^), 21.8 (C^γ^).

### 2D NMR Experiments

Spectra were assigned using COSY and edited-HSQC experiments (blue color for CH_2_ and red color for CH and CH_3_ groups). NOESY experiments were recorded on a 400 MHz spectrometer at 298 K. The experiments were conducted using phase-sensitive ge-2D-NOESY spectra. The number of scans used was 16, and the mixing time was 800 ms.

### X-ray Diffraction Analysis

CIF file for compounds **3k** and **3b** is presented in the Supporting Information. The SHELXL97 program^22^ was used for the refinement of crystal structures, and hydrogen atoms were fitted at theoretical positions.

### Quantum Mechanical calculations

Full geometry optimizations and transition structure (TS) searches were carried out with Gaussian 16^23^ using the M06-2X hybrid functional,^24^ 6-31+G(d,p) basis set with ultrafine integration grids. Bulk solvent effects in either *N*,*N*-dimethylformamide (DMF) or tetrahydrofuran (THF) were considered implicitly through the IEF-PCM polarizable continuum model.^25^ The possibility of different conformations was considered for all structures. All stationary points were characterized by a frequency analysis performed at the same level used in the geometry optimizations from which thermal corrections were obtained at 298.15 K. The quasiharmonic approximation reported by Truhlar et al. was used to replace the harmonic oscillator approximation for the calculation of the vibrational contribution to entropy.^26^ Scaled frequencies were not considered. Mass-weighted intrinsic reaction coordinate (IRC) calculations were carried out by using the Hratchian and Schlegel algorithm^27^ to ensure that the TSs indeed connected the appropriate reactants and products. Gibbs free energies (Δ*G*) were used for the discussion on the relative stabilities of the considered structures. The lowest-energy conformer for each calculated stationary point was considered in the discussion

## Supporting Information Available

Experimental procedures, characterization data, computational details, and copies of the NMR spectra (PDF) (PDF)

## Dedication

Dedicated to Prof. Joan Bosch on the occasion of his 75th birthday.
