# Supplementary material for: Towards Enantiomerically Pure Unnatural α-Amino Acids via Photoredox Catalytic 1,4-Additions to a Chiral Dehydroalanine
Source: J Org Chem. 2022 Sep 30;87(21):14308–18. doi: 10.1021/acs.joc.2c01774 (PMC9639051; doi:10.1021/acs.joc.2c01774)
Supplement: Supplementary file 1 — jo2c01774_si_001.pdf [file jo2c01774_si_001.pdf]

Supporting Information

Towards Enantiomerically Pure Unnatural α-Amino Acids via Photoredox Catalytic 1,4-Additions to a Chiral Dehydroalanine

Paula Oroz, Claudio D. Navo, Alberto Avenoza, Jesús H. Busto, Francisco Corzana, Gonzalo Jiménez-Osés and Jesús M. Peregrina*

Contents

1. Additional information to follow the text of the manuscript.................................. S02-S06
2. Experimental procedures.. S07-S17
3. X-Ray data for compounds **3b** and **3k** S18-S20
4. NOESY spectra to determine the absolute configurations..................................... S21-S29
5. NMR spectra S30-S82
6. Quantum Mechanical calculations S83-S99

1. Additional information to follow the text of the manuscript.

Table S1. Study of other *C*-nucleophiles used in the Michael additions to Dha **1** in dry THF

Entry	*C*-nucleophile	Results
**1**	Ph-C≡C-Cu	No reaction, starting product is recovered.
**2**	^{i}PrMgBr + CuI	Low yield of a mixture of 1,2- and 1,4 adducts along with other unknown products

Table S2. Study of conditions to carry out the photoredox reaction between Boc-glycine (Boc-Gly) and methyl 2-acetamidoacrylate (MAA) to give 2,4-diaminobutyric acid derivative (2,4-Dab adduct).

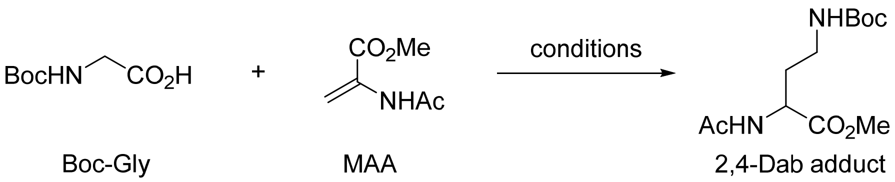

Entry	Deviation from standard condition[a]	Conversion to 2,4-Dab adduct[b]
**1**	None	100%
**2**	Without light	0%
**3**	Without base	0%
**4**	Without catalyst	0%
**5**	[Mes-Acr]ClO_4 as catalyst	50%
**6**	[Ir[dF(CF_3)ppy]$_2$(dtbbpy)]PF_6 as catalyst	100%
**7**	Ir(ppy)$_3$ as catalyst	0%
**8**	No degassed DMF as solvent	100%
**9**	DMSO as solvent	100%
**10**	CH_2Cl_2 as solvent	18%

[a] Conditions: dehydroalanine MAA (20 mg, 0.12 mmol), Boc-Gly (29 mg, 0.17 mmol), Cs_2CO_3 (58 mg, 0.18 mmol), 1,2,3,5-tetrakis(carbazol-9-yl)-4,6-dicyanobenzene (4CzIPN, 5 mg, 5 mol%), anhydrous DMF (1 mL), Blue LED, r.t, 3 h. [b] Conversion determined by NMR.

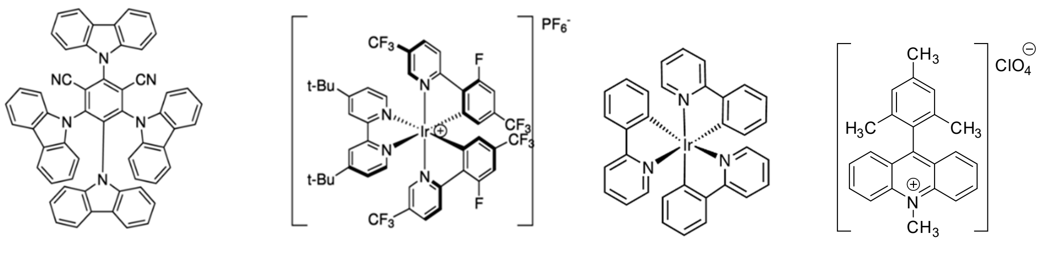

4CzIPN [Ir[dF(CF_3)ppy]2(dtbbpy)]PF_6 Ir(ppy)$_3$ [Mes-Acr]ClO_4

Figure S1. Determination of the yield of reactions between AAM and Boc-Gly by ^{1}H NMR 300 MHz

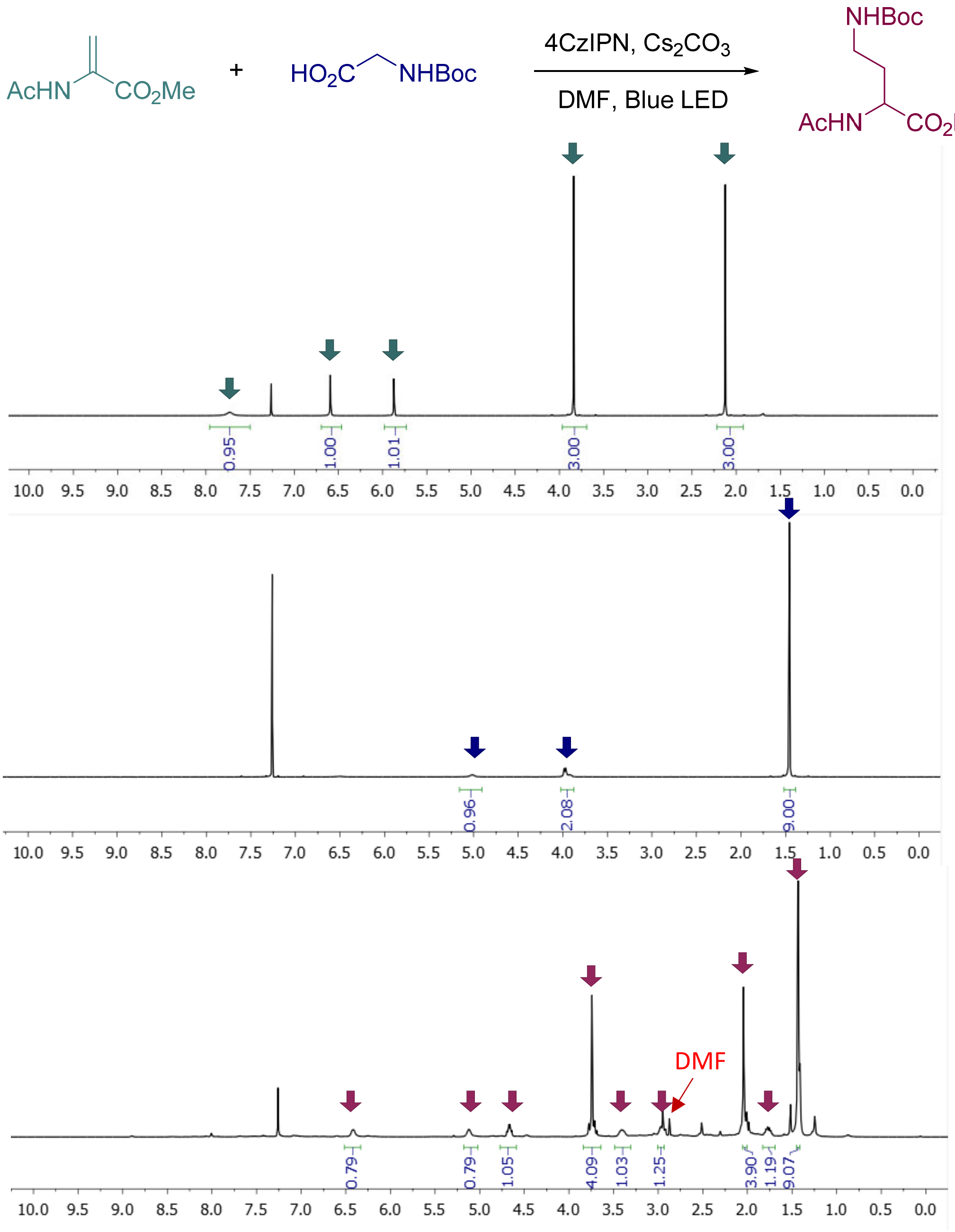

Figure S1. Continued

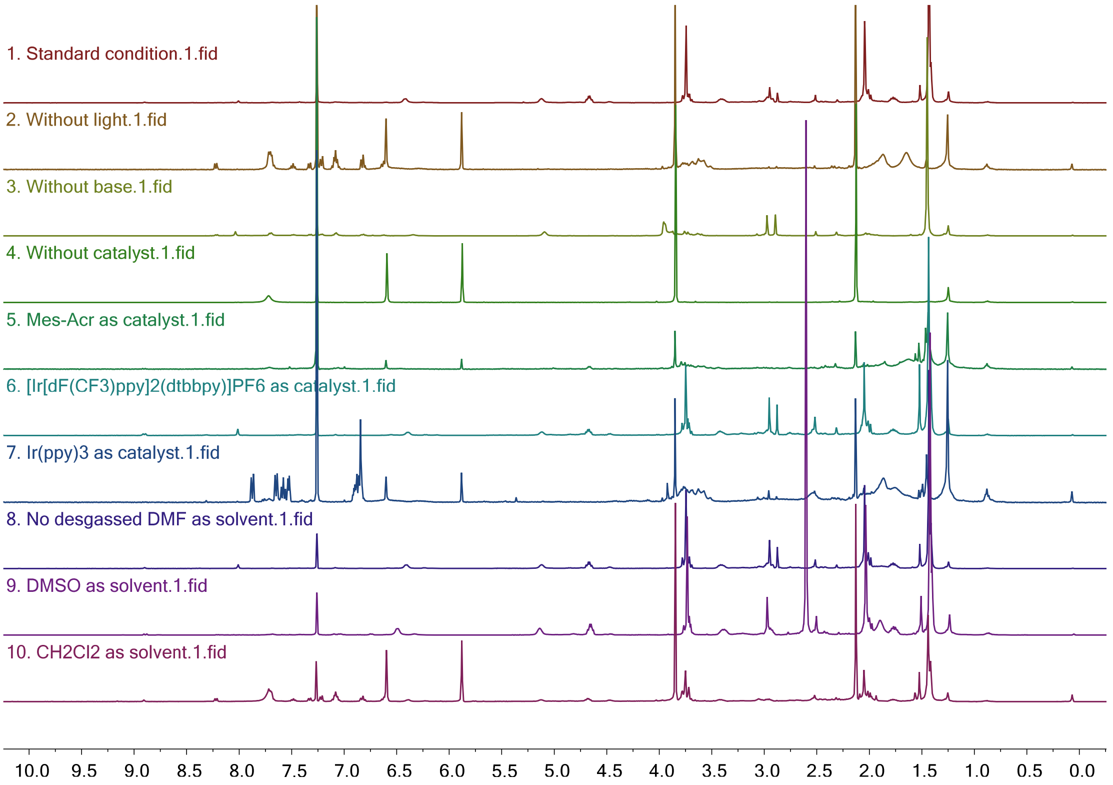

Scheme S1. We tested this reaction with several chiral amino acids (Boc-L-Ala, Boc-L-Leu, and Boc-L-Pro). In all cases the conversion was complete, but we obtained a mixture of two adducts in similar ratios because it was not possible to control the chirality of the radical generated from the amino acid. (**A**) Tested photoredox reactions of Dha **1** with Boc-Ala, Boc-Leu, and Boc-Pro. ^{1}H NMR corresponding to reaction of Dha **1** with Boc-L-Ala (**B**), Boc-L-Leu (**C**) Boc-L-Pro (**D**)

(**A**)

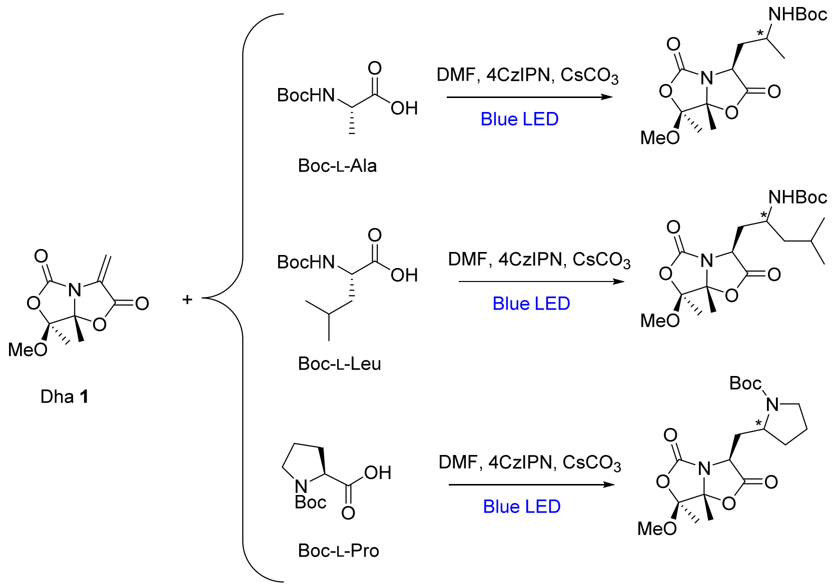

(**B**)

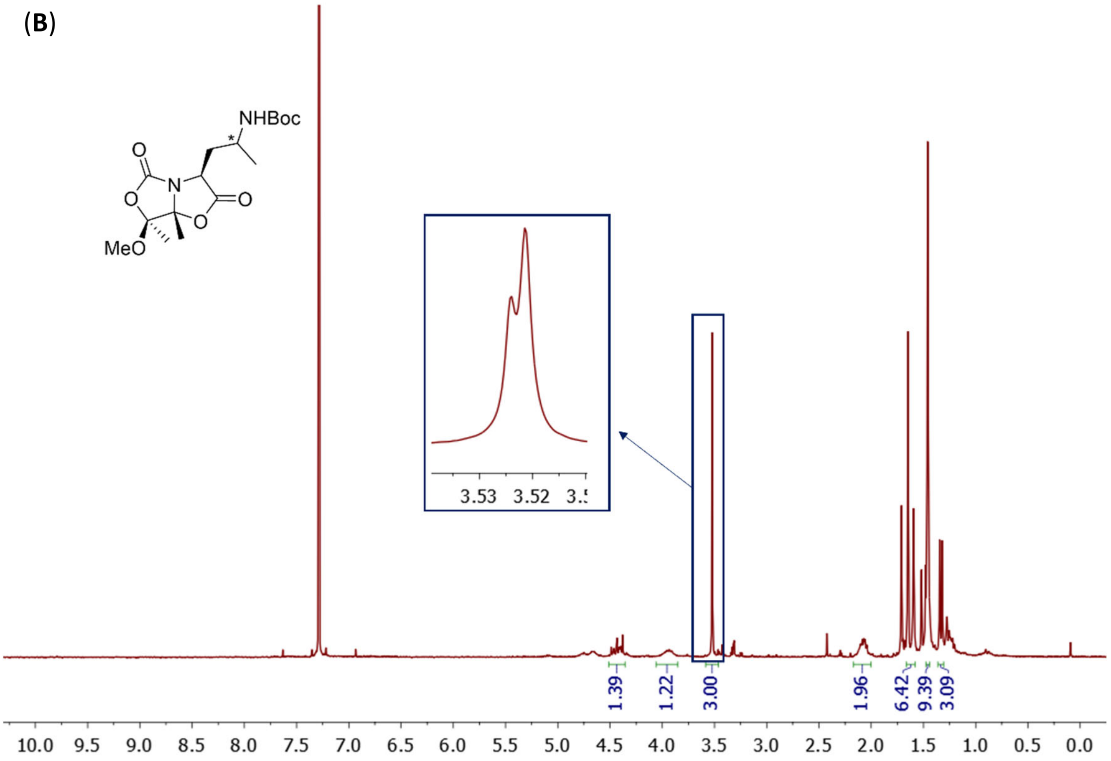
NHBoc
MeO
3.53 3.52
1.39
1.22
3.00
1.96
6.42
9.39
3.09
10.0 9.5 9.0 8.5 8.0 7.5 7.0 6.5 6.0 5.5 5.0 4.5 4.0 3.5 3.0 2.5 2.0 1.5 1.0 0.5 0.0

(**C**)

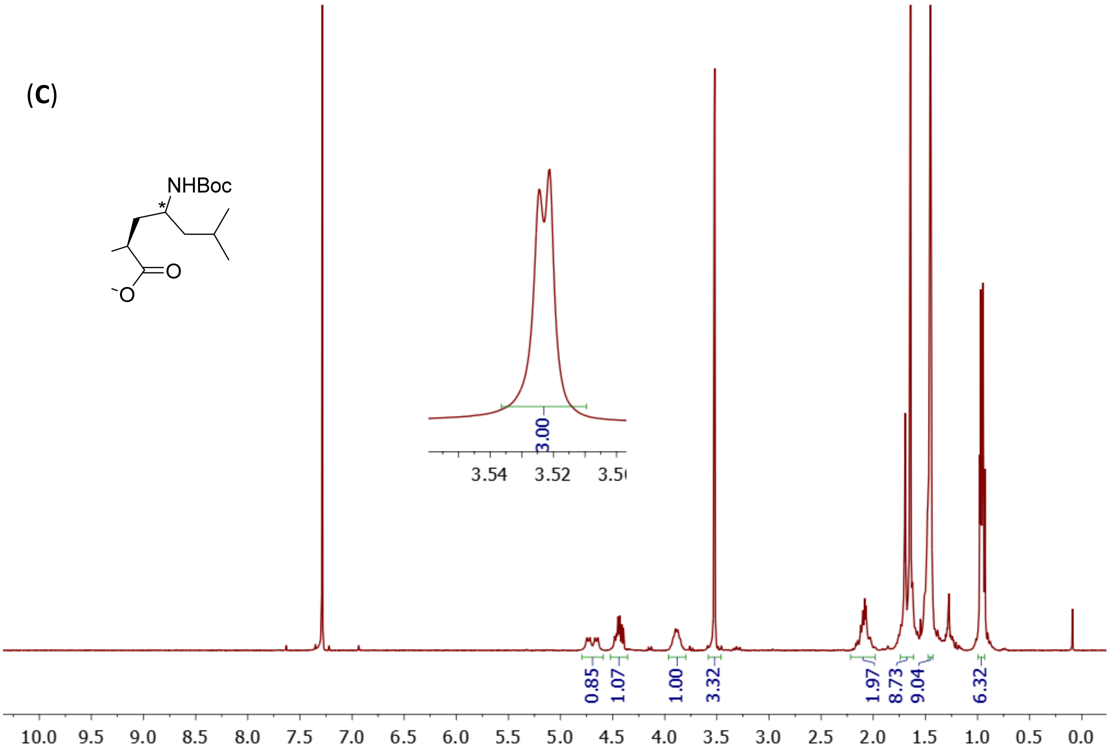
NHBoc
3.00
3.54 3.52
0.85
1.07
1.00
3.32
1.97
8.73
9.04
6.32
10.0 9.5 9.0 8.5 8.0 7.5 7.0 6.5 6.0 5.5 5.0 4.5 4.0 3.5 3.0 2.5 2.0 1.5 1.0 0.5 0.0

(**D**)

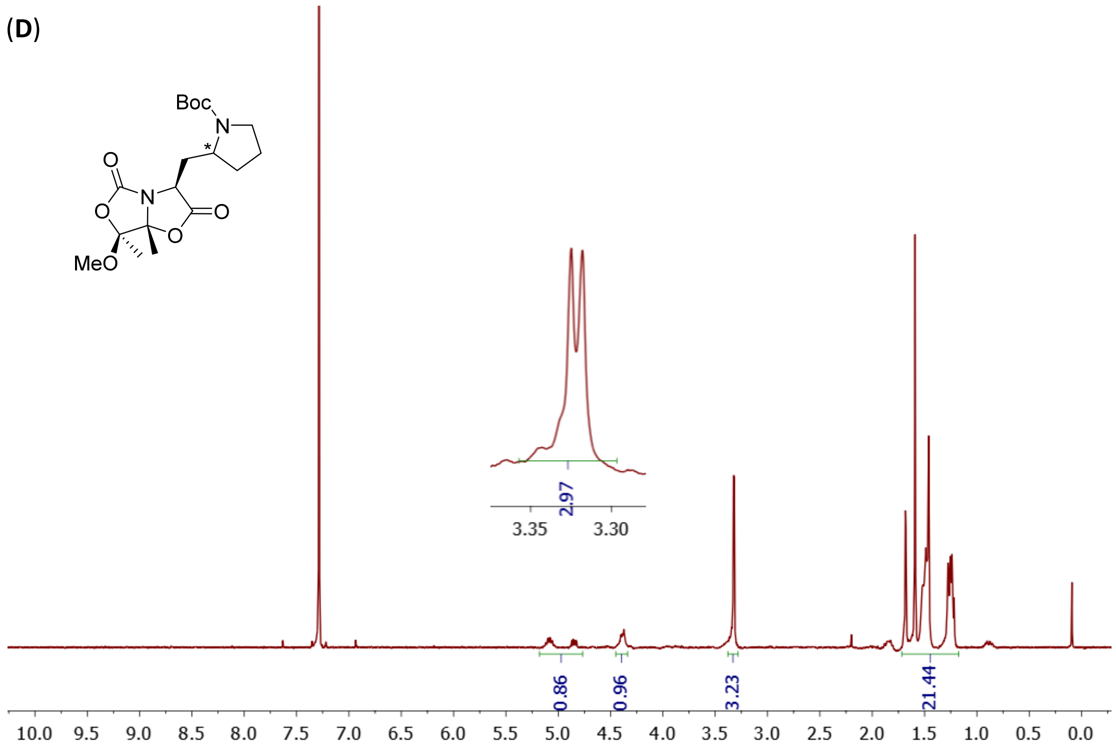

Scheme S2. Proposed mechanism of the 1,4-conjugated additions of *X*-nucleophiles to Dha **1**.

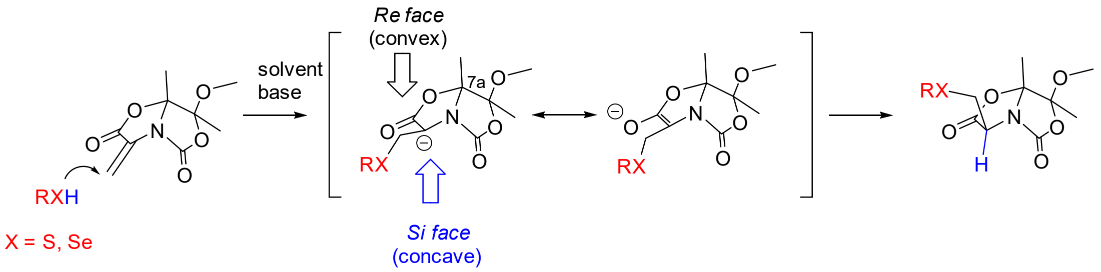

We assume that the mechanism is similar to that previously described for *S*- and *Se*-Michael additions to Dha **1**.[S1] In those works, the calculated structure of a model enolate using MeS^- as a nucleophile revealed a slight pyramidalization of the enolic carbon, suggesting a more favorable protonation by the concave (*Si*) face. The steric hindrance exerted by the bridgehead methyl group at position 7a and the appearance of torsional strain in the five-membered oxazolidin-5-one upon protonation of the enolate by the convex (*Re*) face also favor protonation by the, in principle, less accessible concave face.

[S1] Gutiérrez-Jiménez, M. I.; Aydillo, C.; Navo, C. D.; Avenoza, A.; Corzana, F.; Jiménez-Osés, G.; Zurbano, M. M.; Busto, J. H.; Peregrina, J. M. *Org. Lett.* **2016**, *18*, 2796–2799.

2. Experimental Procedures

2.1. Reagents and general procedures. Commercial reagents were used without further purification. Solvents were dried and redistilled prior to use in the usual way. All reactions were performed in oven-dried glassware with magnetic stirring under an inert atmosphere unless noted otherwise. Analytical thin layer chromatography (TLC) was performed on glass plates precoated with a 0.25 mm thickness of silica gel. The TLC plates were visualized with UV light and by staining with Hanessian solution (ceric sulfate and ammonium molybdate in aqueous sulfuric acid) or sulfuric acid-ethanol solution. Column chromatography was performed on silicagel (230−400 mesh). Optical rotations (OR) were measured with a polarimeter at a concentration (*c*) expressed in g/100 mL. ^{1}H and ^{13}C NMR spectra were measured with a 400 MHz spectrometer with TMS as the internal standard and in D_2O with TMS as external standard in a coaxial microtube. Multiplicities are quoted as singlet (s), broad singlet (br s), doublet (d), doublet of doublets (dd), triplet (t), or multiplet (m). Spectra were assigned using COSY and HSQC. The results of these experiments were processed with MestreNova software. Melting points were determined on a Büchi melting-point apparatus and are uncorrected. High resolution electrospray mass (ESI) spectra were recorded on a microTOF spectrometer; accurate mass measurements were achieved by using sodium formate as an external reference.

2.2. NMR experiments. NMR experiments were performed on a 400 spectrometer at 298 K. Magnitude-mode ge-2D COSY spectra were acquired with gradients by using the *cosygpqf* pulse program with a pulse width of 90º. Phase-sensitive ge-2D HSQC spectra were acquired by using z-filter and selection before t1 removing the decoupling during acquisition by use of the *invigpndph* pulse program with CNST2 (*J*HC) = 145. Phase-sensitive ge-2D NOESY experiments were performed. NOE intensities were normalized with respect to the diagonal peak at zero mixing time.

2.3. *C*-Michael addition on Dha 1 followed by hydrolysis to obtain glutamic acid derivatives

2.3.1. Diethyl 2-(((3*S*,7*S*,7a*R*)-7-methoxy-7,7a-dimethyl-2,5-dioxotetrahydro-5H-oxazolo[4,3-*b*]oxazol-3-yl)methyl)malonate 3a)

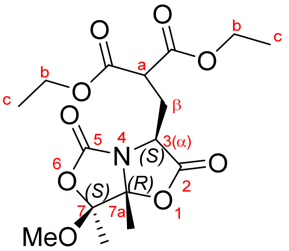

Chiral bicyclic Dha **1** (21 mg, 0.1 mmol, 1.0 equiv.) and diethyl malonate **2a** (18 μL, 0.11 mmol, 1.1 equiv.) were dissolved, at room temperature, in anhydrous THF (final concentration 0.1 M) in a Schlenk under Ar atmosphere. Then, a 1 M solution of LHMDS in THF (0.2 mL, 2.0 equiv.) was added with a syringe. The reaction was monitored by TLC (7:3, hexanes/ethyl acetate, R_f (**Dha**) = 0.75) and, once completed (5 min), the solution was dried under vacuum. The crude mixture was purified by column chromatography on silica gel (hexanes/ethyl acetate, 7:3) to afford compound **3a** as a sticky foam (30 mg, 0.08 mmol, 81% yield). $[\alpha]_D^{20}$ +83.7 (c 1.0, $CHCl_3$). HRMS (ESI) m/z: $[M + Na]^+$ Calcd. for $C_{16}H_{23}NO_9Na$ 396.1265; Found 396.1268. ^{1}H NMR ($CDCl_3$, 400 MHz): δ 4.43 (dd, 1H, *J* = 11.6, 5.2 Hz, $H^{3\alpha}$), 4.16-4.28 (m, 4H, $2CH_2^{b}$), 3.66 (dd, 1H, *J* = 8.3, 6.0 Hz, H^{a}), 3.48 (s, 3H, OMe^{7}), 2.66 (ddd, 1H, *J* = 14.0, 8.3, 5.2 Hz, H^{β}), 2.26 (ddd, 1H, *J* = 14.0, 11.6, 6.0 Hz, H^{β}), 1.65 (s, 3H, CH_3^{7a}), 1.61 (s, 3H, CH_3^{7}), 1.24-1.32 (m, 6H, $2CH_2^{c}$). $^{13}C\{^{1}H\}$ NMR ($CDCl_3$, 100 MHz): δ 171.1, 168.6, 168.0, 159.2 (4CO), 108.3 (C^{7}), 101.7 (C^{7a}), 62.2 (C^{b}), 62.1 (C^{b}), 59.0 ($C^{3\alpha}$), 51.7 (OMe^{7}), 48.9 (C^{a}), 30.2 (C^{β}), 22.0 (CH_3^{7a}), 16.6 (CH_3^{7}), 14.1 (C^{c}), 14.1 (C^{c}).

2.3.2. Methyl(3*R*,3'*S*,7*S*,7a*R*,7'*S*,7'a*R*)-7,7'-dimethoxy-7,7a,7',7'a-tetramethyl-2',5,5' trioxohexahydro-5*H*,5'*H*-[3,3'-bioxazolo[4,3-*b*]oxazole]-3(2*H*)-carboxylate (3b)

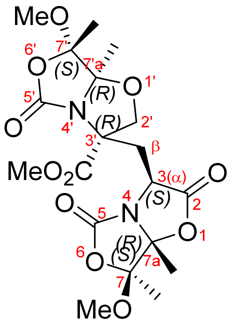

Chiral bicyclic Dha **1** (21 mg, 0.1 mmol, 1.0 equiv.) and chiral bicyclic serine derivative **2b** (24 mg, 0.1 mmol, 1.0 equiv.) were dissolved in anhydrous THF (final concentration 0.1 M) in a Schlenk under Ar atmosphere. Then, the mixture was cooled down to -78 ºC and a 1 M solution of LHMDS in THF (0.2 mL, 2.0 equiv.) was added with a syringe. The reaction was monitored by TLC (7:3, hexanes/ethyl acetate, R_f (**Dha**) = 0.75) and, once completed (5 min), the solution was dried under vacuum. The crude mixture was purified by column chromatography on silica gel (hexanes/ethyl acetate, 7:3) to afford compound **3b** as a sticky foam, but with time and solvents we got monocrystals. (32 mg, 0.07 mmol, 70% yield). $[\alpha]_D^{20}$ +49.4 (c 1.0, $CHCl_3$). HRMS (ESI) m/z: $[M + Na]^+$ Calcd. for $C_{19}H_{26}N_2O_{11}Na$ 481.1429; Found 481.1411. ^{1}H NMR ($CDCl_3$, 400 MHz): δ 5.00 (d, 1H, J = 9.2 Hz, $H^{2'}$), 4.24 (dd, 1H, J = 11.0, 3.7 Hz, $H^{3\alpha}$), 4.16 (d, 1H, J = 9.2 Hz, $H^{2'}$), 3.79 (s, 3H, CO_2CH_3), 3.48 (s, 3H, OMe^7), 3.45 (s, 3H, $OMe^{7'}$), 3.33 (dd, 1H, J = 14.7, 11.0 Hz, H^β), 2.49 (dd, 1H, J = 14.7, 3.7 Hz, H^β), 1.66 (s, 3H, CH_3^{7a}), 1.62 (s, 3H, CH_3^7), 1.56 (s, 3H, $CH_3^{7'}$), 1.36 (s, 3H, $CH_3^{7'a}$). $^{13}C\{^1H\}$ NMR ($CDCl_3$, 100 MHz): δ 170.9, 170.5, 159.0, 154.7 (4CO), 107.8, 107.7, 102.5, 102.3 ($4C^{7,7',7a,7'a}$), 72.8 ($C^{2'}$), 67.2 ($C^{3'}$), 57.8 ($C^{3\alpha}$), 53.4 ($CO_2\underline{C}H_3$), 51.7 (OMe^7), 51.7 ($OMe^{7'}$), 34.2 (C^β), 21.5 (CH_3^{7a}), 18.6 ($C^{7'a}$), 16.6 ($CH_3^{7'}$), 16.2 (CH_3^7).

2.3.3. Diethyl 2-(((3*S*,7*S*,7a*R*)-7-methoxy-7,7a-dimethyl-2,5-dioxotetrahydro-5H-oxazolo[4,3-*b*]oxazol-3-yl-3-*d*)methyl)malonate (3a-D)

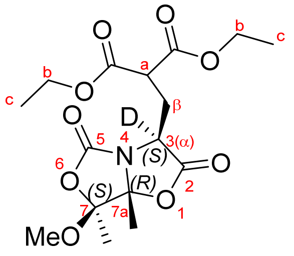

Chiral bicyclic Dha **1** (21 mg, 0.1 mmol, 1.0 equiv.) and diethyl malonate **2a** (18 µL, 0.11 mmol, 1.1 equiv.) were dissolved, at room temperature, in a 9:1 mixture of 2-propanol-OD (^{i}PrOD) and anhydrous CH_2Cl_2 (final concentration 0.1 M) in a Schlenk under Ar atmosphere. Then, a 1 M solution of LHMDS in THF (0.2 mL, 2.0 equiv.) was added with a syringe. The reaction was monitored by TLC (7:3, hexanes/ethyl acetate, R_f (**Dha**) = 0.75) and, once completed (5 min), the solution was dried under vacuum. The crude mixture was purified by column chromatography on silica gel (hexanes/ethyl acetate, 7:3) to afford compound **3a-D** as a sticky foam (27 mg, 0.07 mmol, 73% yield, 94%D). $[\alpha]_D^{20}$ +85.4 (c 1.0, $CHCl_3$). HRMS (ESI) m/z: $[M + H]^+$ Calcd. for $C_{16}H_{23}DNO_9$ 375.1508; Found 375.1504. ^{1}H NMR ($CDCl_3$, 400 MHz): δ 4.43 (dd, 0.06H, J = 11.6, 5.2 Hz, $H^{3\alpha}$), 4.16-4.28 (m, 4H, $2CH_2^b$), 3.66 (dd, 1H, J = 8.3, 5.9 Hz, H^a), 3.48 (s, 3H, OMe^7), 2.66 (dd, 1H, J = 14.4, 8.3 Hz, H^β), 2.25 (dd, 1H, J = 14.4, 5.9 Hz, H^β), 1.65 (s, 3H, CH_3^{7a}), 1.61 (s, 3H, CH_3^7), 1.24-1.31 (m, 6H, $2CH_2^c$). $^{13}C\{^1H\}$ NMR ($CDCl_3$, 100 MHz): δ 171.1, 168.6, 168.0, 159.2 (4CO), 108.3 (C^7), 101.7 (C^{7a}), 62.2 (C^b), 62.1 (C^b), 58.8 (t, J= 21.6 Hz, $C^{3\alpha}$), 51.7 (OMe^7), 48.8 (C^a), 30.1 (C^β), 22.0 (CH_3^{7a}), 16.6 (CH_3^7), 14.1 (C^c), 14.1 (C^c).

2.3.4. General procedure for hydrolysis of Michael adducts. Compound **3a** (15 mg, 0.04 mmol), **3a-D** (15 mg, 0.04 mmol) or **3b** (15 mg, 0.03 mmol) was suspended in a 6 M HCl aqueous solution (3.0 mL) and the reaction mixture was stirred at 60 ºC in an oil bath for 16 h. The solvent was then removed under vacuum, the crude mixture was dissolved in water (5 mL), washed with ethyl acetate (5 mL) and purified by solid phase extraction in a C18 cartridge to afford **4a** (5.5 mg, 0.04 mmol, 93% yield), **4a-D** (5.7 mg, 0.04 mmol, 96% yield, 88%D) or **4b** (6 mg, 0.03 mmol, 95% yield).

2.3.5. L-Glutamic acid hydrochloride (4a)

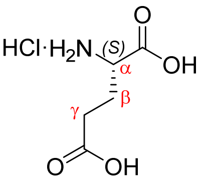

Following the general procedure for hydrolysis. Yield after SPE cartridge: 5.5 mg, 93%. White solid. $[\alpha]_D^{20}$ +32.7 (c 1.0, 6M HCl). HRMS (ESI) m/z: $[M + H]^+$ Calcd. for $C_5H_{10}NO_4$ 148.0604; Found 148.0606. ^1H NMR (D_2O, 400 MHz): δ 4.01 (t, 1H, J = 6.5 Hz, H^α), 2.57-2.62 (m, 2H, H^γ), 2.11-2.25 (m, 2H, H^β). $^{13}C\{^1H\}$ NMR (D_2O, 100 MHz): δ 176.4, 172.2 (2CO), 52.6 (H^α), 29.5 (C^γ), 25.0 (C^β).

2.3.6. L-Glutamic-2-*d* acid hydrochloride (4a-D)

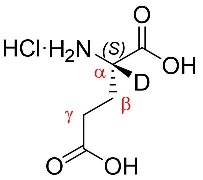

Following the general procedure for hydrolysis. Yield after SPE cartridge: 5.7 mg, 96%. White solid. $[\alpha]_D^{20}$ +30.2 (c 1.0, 6M HCl). HRMS (ESI) m/z: $[M - H]^-$ Calcd. for $C_5H_7DNO_4$ 147.0522; Found 147.0519. ^1H NMR (D_2O, 400 MHz): δ 4.08 (t, 1H, J = 6.6 Hz, 0.12H^α), 2.60-2.66 (m, 2H, H^γ), 2.15-2.28 (m, 2H, H^β). $^{13}C\{^1H\}$ NMR (D_2O, 100 MHz): δ 176.4, 172.0 (2CO), 52.1 (t, J = 22.5 Hz, H^α), 29.5 (C^γ), 24.9 (C^β).

2.3.7. (2*R*,4*S*)-2,4-Diamino-2-(hydroxymethyl)pentanedioic acid dihydrochloride (4b)

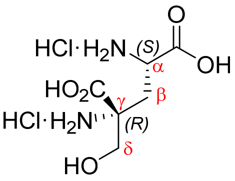

Following the general procedure for hydrolysis. Yield after SPE cartridge: 6.0 mg, 95%. White solid. $[\alpha]_D^{20}$ +10.6 (c 1.0, 6M HCl). HRMS (ESI) m/z: $[M + H]^+$ Calcd. for $C_6H_{13}N_2O_5$ 193.0819; Found 193.0822. ^1H NMR (D_2O, 400 MHz): δ 4.31 (t, 1H, J = 9.4 Hz, H^α), 3.87 (d, 1H, J = 11.6 Hz, H^δ), 3.82 (d, 1H, J = 11.6 Hz, H^δ), 2.81 (dd, 1H, J = 13.8, 9.4 Hz, H^β), 2.39 (dd, 1H, J = 13.8, 9.4 Hz, H^β). $^{13}C\{^1H\}$ NMR (D_2O, 100 MHz): δ 174.5, 172.4 (2CO), 65.5 (C^γ), 62.7 (C^δ), 50.0 (C^α), 32.0 (C^β).

2.4. Photoredox catalytic 1,4-additions to chiral Dha 1 followed by hydrolysis to obtain carbon-β-substituted UAA.

Compound **2a**, carboxylic acids **2c-j** and **2l-o** are commercially available. Bicyclic compound **2b**, carboxylic acid **2k** and Boc-D-Ser-OMe **7** were synthesized following the procedures described in the references *Chem. Eur. J.* **2007**, *13*, 4840, *Org. Lett.* **2010**, *12*, 612, and *Helv. Chem. Acta* **2015**, *98*, 260, respectively. The NMR spectra of these synthesized known compounds were included in this SI.

2.4.1. General procedure for photoredox catalytic 1,4-additions. Chiral bicyclic Dha **1** (21 mg, 0.1 mmol, 1.0 equiv.), the corresponding carboxylic acid **2c-n** (0.12 mmol, 1.2 equiv.), Cs_2CO_3 (39 mg, 0.15 mmol, 1.5 equiv.) and 4CzIPN (4 mg, 0.005 mmol, 0.05 equiv.) were added in sample vials. The tube was evacuated and back-filled with N_2 (three times). Then, anhydrous DMF (1 mL, final concentration 0.1 M) was added using a syringe. The solution was then stirred at room temperature under the irradiation of Blue LEDs for 2-16 h. Once completed, 1 mL of water was added and extracted with ethyl acetate. The combined organic layer was dried over anhydrous Na_2SO_4, filtered and evaporated under vacuum. The crude mixture was purified by column chromatography (hexanes/ethyl acetate) on silica gel to afford desired products.

Light-promoted reactions have been carried out in a SynLED Parallel Photoreactor (available from Sigma Aldrich). Bottom-lit LEDs (465-470 nm) across a 4x4 reaction block array provides consistent light intensity (130-140 lm) and angle (45°). Built-in cooling fan provides consistent temperature to each parallel reaction. Uses 1-2 dram scintillation vials or microwave vials (O.D. of 1.7 cm or less). Power supply is wall plug power supply 700 mA 12 W. Wheaton sample vials (clear borosilicate glass vial).

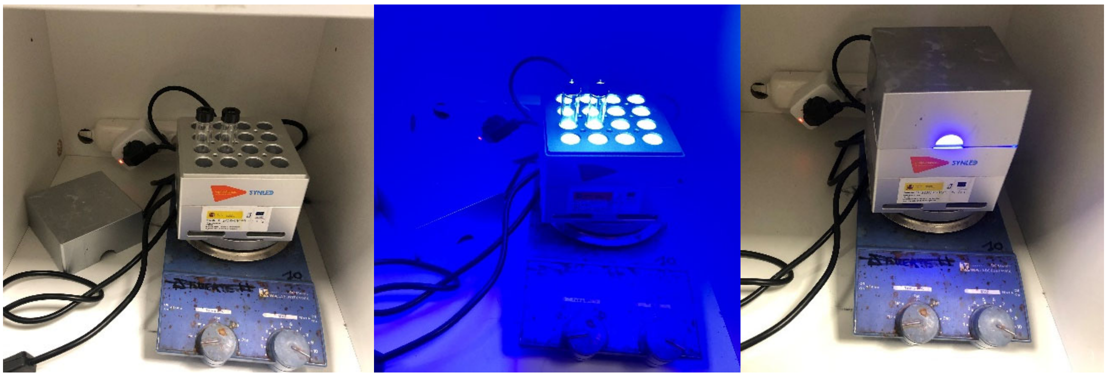

2.4.2. Procedure to scale up the photoredox catalytic 1,4-additions. Chiral bicyclic Dha **1** (200 mg, 0.93 mmol, 1.0 equiv.), 2-(phenylselanyl)acetic acid **2k** (240 mg, 1.12 mmol, 1.2 equiv.), Cs_2CO_3 (371 mg, 1.40 mmol, 1.5 equiv.) and 4CzIPN (38 mg, 0.046 mmol, 0.05 equiv.) were added in a 50 mL flask. The vessel was evacuated and refilled with N_2 (× 3). Then, anhydrous DMF (10 mL, final concentration 0.1 M) was added using a syringe. The solution was then stirred at room temperature under the irradiation of Blue LEDs for 16 h. Once completed, 10 mL water was added and extracted with ethyl acetate. The combined organic layer was dried over anhydrous Na_2SO_4 and dried under vacuum. The crude mixture was purified by column chromatography (hexanes/ethyl acetate, 7:3) on silica gel to afford **3k** (205 mg, 0.53 mmol, 57%).

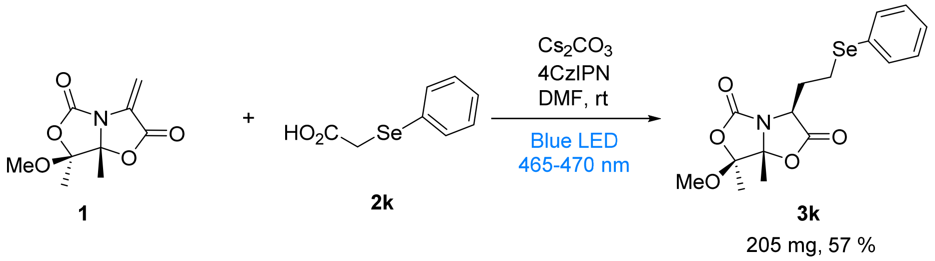

Light-promoted reactions on a larger scale have been carried out irradiating with blue light of a RGB LED of 50 W. Ce RoHS EMC IP65 50W at 15 cm from the flask on the stirring plate in a photochemical cabinet.

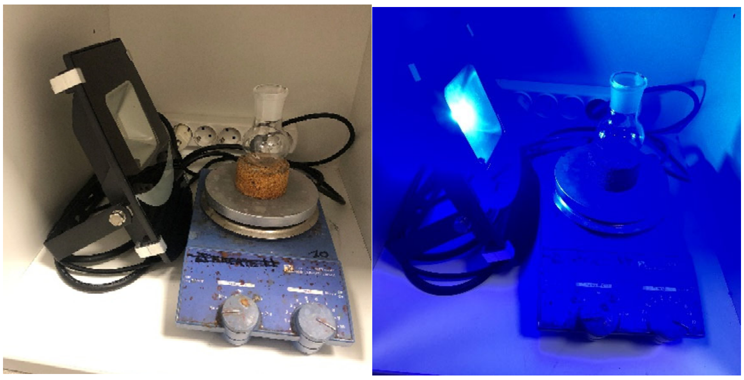

2.4.3. General procedure for deuterated compounds. Chiral bicyclic Dha **1** (21 mg, 0.1 mmol, 1.0 equiv.), deuterated carboxylic acids (0.12 mmol, 1.2 equiv.), Cs_2CO_3 (39 mg, 0.15 mmol, 1.5 equiv.) and 4CzIPN (4 mg, 0.005 mmol, 0.05 equiv.) were added in sample vials. The tube was evacuated and back-filled with N_2 (three times). Then, anhydrous DMF (1 mL, final concentration 0.1 M) and D_2O (50 µL) were added using a syringe. The solution was then stirred at room temperature under the irradiation of Blue LEDs (SynLED parallel photoreactor) for 2-16 h. Once completed, 1 mL of D_2O was added and extracted with ethyl acetate. The combined organic layer was dried over anhydrous Na_2SO_4, filtered and evaporated under vacuum. The crude mixture was purified by column chromatography (hexanes/ethyl acetate) on silica gel to afford desired products.

2.4.4. General procedure for hydrolysis of adducts. Compounds were suspended in a 4 M HCl aqueous solution and the reaction mixture was stirred at 60 ºC in an oil bath for 16 h. The solvent was then removed under vacuum, the crude mixture was dissolved in water (5 mL), washed with ethyl acetate (5 mL) and purified by solid phase extraction in a C18 cartridge to afford desired products.

2.4.5. Aminolysis of adduct 3k with HCl·Phe-OBn. Compound **3k** (64 mg, 0.16 mmol), the corresponding amino ester hydrochloride (H-Phe-OBn·HCl, 73 mg, 0.25 mmol, 1.5 equiv.) and sodium 2-ethylhexanoate (69 mg, 0.42 mmol, 2.5 equiv.) were charged in an oven dried Schlenk flask and subjected to vacuum/N_2 cycle (× 3) to remove possible moisture. Under N_2 atmosphere, dry THF (8 mL, 50 mL/mmol) was added into the flask by a syringe. The solution was stirred at room temperature for 24 h. After that time, brine and ethyl acetate were added to the solution. Layers were separated and aqueous layer was back extracted with more ethyl acetate. The crude mixture was purified by column chromatography (hexanes/ethyl acetate 7:3) on silica gel to afford the desired product **5k**.

2.4.6. *tert*-Butyl (2-((3*S*,7*S*,7a*R*)-7-methoxy-7,7a-dimethyl-2,5-dioxotetrahydro-5*H*-oxazolo[4,3-*b*]oxazol-3-yl)ethyl)carbamate (3c)

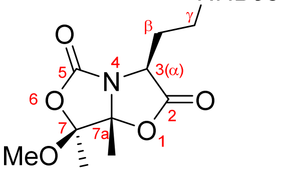

Following the general procedure. Yield after column chromatography (hexanes/ethyl acetate, 8:2): 30 mg, 88%. White solid. Mp:116-119. $[\alpha]_D^{20}$ +34.5 (c 1.0, $CHCl_3$). HRMS (ESI) m/z: $[M + H]^+$ Calcd. for $C_{15}H_{24}N_2O_7$ 345.1656; Found 345.1652. ^1H NMR ($CDCl_3$, 400 MHz): δ 5.13 (br s, 1H, H^{NH}), 4.37 (dd, 1H, J = 12.0, 4.2 Hz, $H^{3\alpha}$), 3.56-3.60 (m, 1H, CH_2^{γ}), 3.51 (s, 3H, OMe^7), 3.15-3.23 (m, 1H, CH_2^{γ}), 2.22-2.29 (m, 1H, H^{β}), 1.76-1.84 (m, 1H, H^{β}), 1.63 (s, 6H, CH_3^{7a}, CH_3^{7}), 1.44 (s, 9H, NHBoc). $^{13}C\{^1H\}$ NMR ($CDCl_3$, 100 MHz): δ 172.0, 159.6, 156.0 (3CO), 108.9 (C^7), 101.3 (C^{7a}), 79.7 ($\underline{C}(CH_3)_3$), 58.7 ($C^{3\alpha}$), 51.9 (OMe^7), 37.6 (C^{γ}), 31.4 (C^{β}), 28.5 ($C(\underline{C}H_3)_3$), 22.4 (CH_3^{7a}), 16.8 (CH_3^{7}).

2.4.7. *tert*-Butyl (2-((3*S*,7*S*,7a*R*)-7-methoxy-7,7a-dimethyl-2,5-dioxotetrahydro-5*H*-oxazolo[4,3-*b*]oxazol-3-yl-3-*d*)ethyl)carbamate (3c-D)

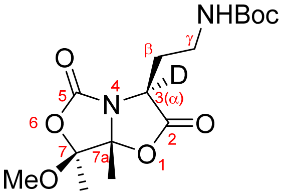

Following the general procedure for deuterated compounds. Yield after column chromatography (hexanes/ethyl acetate, 8:2): 28 mg, 82% [93% deuterated]. White solid. Mp: 118-118. $[\alpha]_D^{20}$ +36.4 (c 1.0, $CHCl_3$). HRMS (ESI) m/z: $[M + H]^+$ Calcd. for $C_{15}H_{23}DN_2O_7Na$ 368.1544; Found 368.1549. ^1H NMR ($CDCl_3$, 400 MHz): δ 5.12 (br s, 1H, H^{NH}), 4.37 (dd, 0.07H, J = 11.4, 4.2 Hz, $H^{3\alpha}$), 3.56-3.60 (m, 1H, CH_2^{γ}), 3.51 (s, 3H, OMe^7), 3.13-3.25 (m, 1H, CH_2^{γ}), 2.20-2.29 (m, 1H, H^{β}), 1.76-1.84 (m, 1H, H^{β}), 1.63 (s, 6H, CH_3^{7a}, CH_3^7), 1.44 (s, 9H, NHBoc). $^{13}C\{^1H\}$ NMR ($CDCl_3$, 100 MHz): δ 172.0, 159.6, 155.9 (3CO), 108.9 (C^7), 101.3 (C^{7a}), 79.7 ($\underline{C}(CH_3)_3$), 51.9 (OMe^7), 37.5 (C^{γ}), 31.2 (C^{β}), 28.5 ($C(\underline{C}H_3)_3$), 22.4 (CH_3^{7a}), 16.8 (CH_3^7). *$C^{3\alpha}$ is not observed.

2.4.8. (3*S*,7*S*,7a*R*)-7-Methoxy-7,7a-dimethyl-3-phenethyldihydro-5*H*-oxazolo[4,3-*b*]oxazole-2,5(3*H*)-dione (3d)

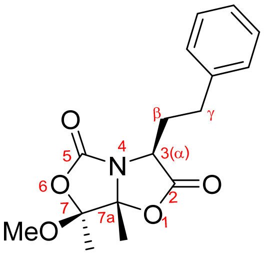

Following the general procedure. Yield after column chromatography (hexanes/ethyl acetate, 8:2): 22 mg, 73%. Light yellow solid. Mp:52-55. $[\alpha]_D^{20}$ +52.2 (c 1.0, $CHCl_3$). HRMS (ESI) m/z: $[M + Na]^+$ Calcd. for $C_{16}H_{19}NNaO_5$ 328.1155; Found 328.1150. ^1H NMR ($CDCl_3$, 400 MHz): δ 7.20-7.34 (m, 5H, H^{Ar}), 4.32 (dd, 1H, J = 10.9, 4.8 Hz, $H^{3\alpha}$), 3.51 (s, 3H, OMe^7), 2.82-2.97 (m, 2H, CH_2^{γ}), 2.27-2.36 (m, 1H, H^{β}), 1.95-2.06 (m, 1H, H^{β}), 1.66 (s, 3H, CH_3^{7a}), 1.63 (s, 3H, CH_3^7). $^{13}C\{^1H\}$ NMR ($CDCl_3$, 100 MHz): δ 172.1, 159.5 (2CO), 140.0 (C^{*Ar}), 128.8 ($4C^{Ar}$), 126.6 (C^{Ar}), 108.2 (C^7), 101.6 (C^{7a}), 60.5 ($C^{3\alpha}$), 51.7 (OMe^7), 33.7 (C^{β}), 32.6 (C^{γ}), 22.2 (CH_3^{7a}), 16.7 (CH_3^7).

2.4.9. (3*S*,7*S*,7a*R*)-3-(Cyclohexylmethyl)-7-methoxy-7,7a-dimethyldihydro-5*H*-oxazolo[4,3-*b*]oxazole-2,5(3*H*)-dione (3e)

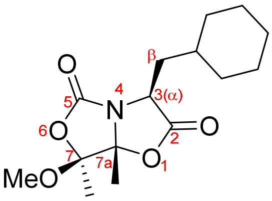

Following the general procedure. Yield after column chromatography (hexanes/ethyl acetate, 8:2): 25 mg, 85%. Light yellow solid. Mp: 60-63. $[\alpha]_D^{20}$ +68.5 (c 1.0, $CHCl_3$). HRMS (ESI) m/z: $[M + Na]^+$ Calcd. for $C_{15}H_{23}NNaO_5$ 320.1468; Found 320.1474. ^1H NMR ($CDCl_3$, 400 MHz): δ 4.41 (dd, 1H, J = 11.1, 4.3 Hz, $H^{3\alpha}$), 3.50 (s, 3H, OMe^7), 1.96-2.03 (m, 1H, $1CH_2^{cyclo}$), 1.68-1.86 (m, 5H, $4CH_2^{cyclo}$, $1H^{\beta}$), 1.64 (s, 3H, CH_3^{7a}), 1.62 (s, 3H, CH_3^7), 1.54-1.65 (m, 2H, CH^{cyclo}, $1H^{\beta}$), 1.14-1.30 (m, 3H, $3CH_2^{cyclo}$), 0.56-1.09 (m, 2H, $3CH_2^{cyclo}$). $^{13}C\{^1H\}$ NMR ($CDCl_3$, 100 MHz): δ 173.0, 159.6 (2CO), 107.9 (C^7), 101.6 (C^{7a}), 59.2 ($C^{3\alpha}$), 51.6 (OMe^7), 38.9 (C^{β}), 34.9 (CH^{cyclo}), 33.6 (CH_2^{cyclo}), 31.8 (CH_2^{cyclo}), 26.5 (CH_2^{cyclo}), 26.3 (CH_2^{cyclo}), 26.0 (CH_2^{cyclo}), 22.1 (CH_3^{7a}), 16.6 (CH_3^7).

2.4.10. (3*S*,7*S*,7a*R*)-7-Methoxy-7,7a-dimethyl-3-((1-methylcyclohexyl)methyl)dihydro-5*H*-oxazolo[4,3-*b*]oxazole-2,5(3*H*)-dione (3f)

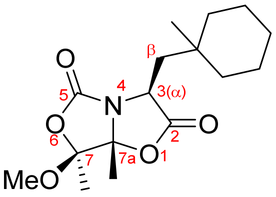

Following the general procedure. Yield after column chromatography (hexanes/ethyl acetate, 8:2): 25 mg, 82%. Light yellow solid. Mp: 76-79. $[\alpha]_D^{20}$ +67.1 (c 1.0, $CHCl_3$). HRMS (ESI) m/z: $[M + Na]^+$ Calcd. for $C_{16}H_{25}NNaO_5$ 334.1625; Found 334.1620. ^1H NMR ($CDCl_3$, 400 MHz): δ 4.45 (dd, 1H, J = 10.9, 2.5 Hz, $H^{3\alpha}$), 3.49 (s, 3H, OMe^7), 1.88 (dd, 1H, J = 14.5, 2.5 Hz, H^{β}), 1.72 (dd, 1H, J = 14.5, 10.9 Hz, H^{β}), 1.66 (s, 3H, CH_3^{7a}), 1.62 (s, 3H, CH_3^7), 1.38-1.52 (m, 10H, H^{cyclo}), 1.07 (s, 3H, CH_3^{cyclo}). $^{13}C\{^1H\}$ NMR ($CDCl_3$, 100

MHz): δ 173.7, 159.4 (2CO), 107.6 (C^{7}), 101.9 (C^{7a}), 58.1 ($C^{3\alpha}$), 51.6 (OMe^{7}), 43.2 (C^{β}), 37.7 (CH_2^{cyclo}), 37.6 (CH_2^{cyclo}), 33.6 (C^{*cyclo}), 26.3 (CH_2^{cyclo}), 24.4 (CH_3^{cyclo}), 22.0 ($2CH_2^{cyclo}$), 22.0 (CH_3^{7a}), 16.6 (CH_3^{7}).

2.4.11. (3*S*,7*S*,7a*R*)-7-Methoxy-7,7a-dimethyl-3-neopentyldihydro-5*H*-oxazolo[4,3-*b*]oxazole-2,5(3*H*)-dione (3g)

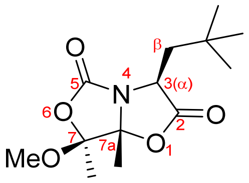

Following the general procedure. Yield after column chromatography (hexanes/ethyl acetate, 8:2): 23 mg, 86%. Yellow solid. Mp: 91-93. $[\alpha]_D^{20}$ +79.5 (c 1.0, $CHCl_3$). HRMS (ESI) m/z: $[M + Na]^+$ Calcd. for $C_{13}H_{21}NNaO_5$ 294.1312; Found 294.1304. ^1H NMR ($CDCl_3$, 400 MHz): δ 4.42 (dd, 1H, J = 11.1, 2.6 Hz, $H^{3\alpha}$), 3.49 (s, 3H, OMe^{7}), 1.87 (dd, 1H, J = 14.5, 2.6 Hz, H^{β}), 1.63-1.68 (m, 1H, H^{β}), 1.65 (s, 3H, CH_3^{7a}), 1.62 (s, 3H, CH_3^{7}), 1.06 (s, 9H, $(CH_3)_3$). $^{13}C\{^1H\}$ NMR ($CDCl_3$, 100 MHz): δ 173.5, 159.4 (2CO), 107.6 (C^{7}), 101.8 (C^{7a}), 58.8 ($C^{3\alpha}$), 51.6 (OMe^{7}), 44.7 (C^{β}), 31.2 ($\underline{C}(CH_3)_3$), 29.3 ($3C(\underline{C}H_3)_3$), 22.0 (CH_3^{7a}), 16.6 (CH_3^{7}).

2.4.12. (3*S*,7*S*,7a*R*)-3-*iso*-Butyl-7-methoxy-7,7a-dimethyldihydro-5*H*-oxazolo[4,3-*b*]oxazole-2,5(3*H*)-dione (3h)

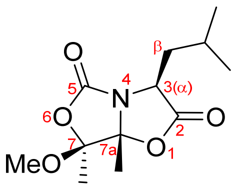

Following the general procedure. Yield after column chromatography (hexanes/ethyl acetate, 8:2): 20 mg, 83%. Sticky foam. $[\alpha]_D^{20}$ +52.5 (c 1.0, $CHCl_3$). HRMS (ESI) m/z: $[M +Na]^+$ Calcd. for $C_{12}H_{19}NNaO_5$ 280.1155; Found 280.1146. ^1H NMR ($CDCl_3$, 400 MHz): δ 4.37 (dd, 1H, J = 11.5, 4.5 Hz, $H^{3\alpha}$), 3.50 (s, 3H, OMe^{7}), 1.88-1.98 (m, 1H, $C\underline{H}(CH_3)_2$), 1.79 (ddd, 1H, J = 13.4, 8.8, 4.5 Hz, H^{β}), 1.64-1.69 (m, 1H, H^{β}), 1.64 (s, 3H, CH_3^{7a}), 1.62 (s, 3H, CH_3^{7}), 1.06 (d, 3H, J = 6.5, $1CH(C\underline{H}_3)_2$), 1.03 (d, 3H, J = 6.7, $1CH(C\underline{H}_3)_2$). $^{13}C\{^1H\}$ NMR ($CDCl_3$, 100 MHz): δ 172.8, 159.6 (2CO), 107.9 (C^{7}), 101.6 (C^{7a}), 59.7 ($C^{3\alpha}$), 51.7 (OMe^{7}), 40.2 (C^{β}), 25.8 ($\underline{C}H(CH_3)_2$), 22.9 ($1CH(\underline{C}H_3)_2$), 22.1 (CH_3^{7a}), 21.3 ($1CH(\underline{C}H_3)_2$), 16.6 (CH_3^{7}).

2.4.13. (3*S*,7*S*,7a*R*)-7-Methoxy-7,7a-dimethyl-3-(2-phenoxyethyl)dihydro-5*H*-oxazolo[4,3-*b*]oxazole-2,5(3*H*)-dione (3i)

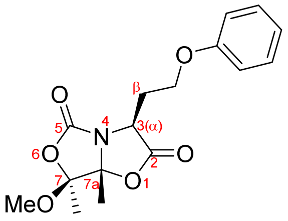

Following the general procedure. Yield after column chromatography (hexanes/ethyl acetate, 8:2): 27 mg, 85%. Sticky foam. $[\alpha]_D^{20}$ +32.2 (c 1.0, $CHCl_3$). HRMS (ESI) m/z: $[M + Na]^+$ Calcd. for $C_{16}H_{19}NNaO_6$ 344.1105; Found 344.1098. ^1H NMR ($CDCl_3$, 400 MHz): δ 7.28-7.31 (m, 2H, CH^{Ar}), 6.92-7.00 (m, 3H, CH^{Ar}), 4.61 (dd, 1H, J = 10.4, 5.0 Hz, $H^{3\alpha}$), 4.15-4.26 (m, 2H, CH_2^{γ}), 3.50 (s, 3H, OMe^{7}), 2.44-2.54 (m, 1H, H^{β}), 2.12-2.21 (m, 1H, H^{β}), 1.66 (s, 3H, CH_3^{7a}), 1.66 (s, 3H, CH_3^{7}). $^{13}C\{^1H\}$ NMR ($CDCl_3$, 100 MHz): δ 171.9, 159.2 (2CO), 129.7, 129.7, 121.4, 115.0, 115.0 ($5C^{Ar}$), 108.2 (C^{7}), 101.6 (C^{7a}), 63.9 (C^{γ}), 57.9 ($C^{3\alpha}$), 51.8 (OMe^{7}), 31.7 (C^{β}), 29.9 (C^{*Ar}), 22.2 (CH_3^{7a}), 16.7 (CH_3^{7}).

2.4.14. (3*S*,7*S*,7a*R*)-7-Methoxy-7,7a-dimethyl-3-(2-(phenylthio)ethyl)dihydro-5*H*-oxazolo[4,3-*b*]oxazole-2,5(3*H*)-dione (3j)

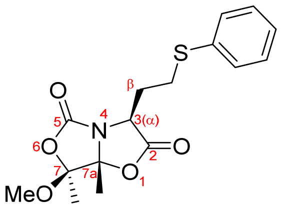

Following the general procedure. Yield after column chromatography (hexanes/ethyl acetate, 8:2): 27 mg, 81%. White solid. Mp: 108-110. $[\alpha]_D^{20}$ +48.2 (c 1.0, $CHCl_3$). HRMS (ESI) m/z: $[M + Na]^+$ Calcd. for $C_{16}H_{19}NNaO_5S$ 360.0876; Found 360.0871. ^1H NMR ($CDCl_3$, 400 MHz): δ 7.13-7.3 (m, 5H, CH^{Ar}), 4.42 (dd, 1H, J = 10.8, 4.8 Hz, $H^{3\alpha}$), 3.44 (s, 3H, OMe^{7}), 3.05-3.14 (m, 1H, CH_2^{γ}), 2.95-3.04 (m, 1H, CH_2^{γ}), 2.13-2.23 (m,

1H, H^{β}), 1.83-1.93 (m, 1H, H^{β}), 1.55 (s, 3H, CH_3^{7}), 1.50 (s, 3H, CH_3^{7a}). $^{13}C\{^1H\}$ NMR ($CDCl_3$, 100 MHz): δ 171.6, 159.3 (2CO), 134.9 (C^{*Ar}), 130.6, 130.6, 129.3, 129.3, 127.0 ($5C^{Ar}$), 108.3 (C^{7}), 101.6 (C^{7a}), 59.8 ($C^{3\alpha}$), 51.8 (OMe^{7}), 31.5 (C^{β}), 30.8 (C^{γ}), 22.1 (CH_3^{7a}), 16.7 (CH_3^{7}).

2.4.15. (3*S*,7*S*,7a*R*)-7-Methoxy-7,7a-dimethyl-3-(2-(phenylselanyl)ethyl)dihydro-5*H*-oxazolo[4,3-*b*]oxazole-2,5(3*H*)-dione (3k)

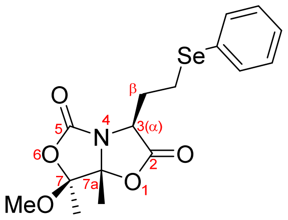

Following the general procedure. Yield after column chromatography (hexanes/ethyl acetate, 8:2): 33 mg, 87%. Sticky foam, but with time and solvents we got monocrystals. $[\alpha]_D^{20}$ +41.2 (c 1.0, $CHCl_3$). HRMS (ESI) m/z: $[M + Na]^+$ Calcd. for $C_{16}H_{19}NNaO_5Se$ 408.0321; Found 408.0320. ^1H NMR ($CDCl_3$, 400 MHz): δ 7.43-7.50 (m, 2H, CH^{Ar}), 7.16-7.22 (m, 3H, CH^{Ar}), 4.41 (dd, 1H, J = 10.9, 4.9 Hz, $H^{3\alpha}$), 3.43 (s, 3H, OMe^{7}), 3.01-3.08 (m, 1H, CH_2^{γ}), 2.89-2.97 (m, 1H, CH_2^{γ}), 2.17-2.28 (m, 1H, H^{β}), 1.88-1.99 (m, 1H, H^{β}), 1.54 (s, 3H, CH_3^{7}), 1.47 (s, 3H, CH_3^{7a}). $^{13}C\{^1H\}$ NMR ($CDCl_3$, 100 MHz): δ 171.6, 159.4 (2CO), 128.9 (C^{*Ar}), 133.7, 133.7, 129.4, 129.4, 127.7, 115.0 ($5C^{Ar}$), 108.3 (C^{7}), 101.5 (C^{7a}), 60.7 ($C^{3\alpha}$), 51.8 (OMe^{7}), 32.4 (C^{β}), 23.7 (C^{γ}), 22.1 (CH_3^{7a}), 16.7 (CH_3^{7}).

2.4.16. *tert*-Butyl (1-(((3*S*,7*S*,7a*R*)-7-methoxy-7,7a-dimethyl-2,5-dioxotetrahydro-5*H*-oxazolo[4,3-*b*]oxazol-3-yl)methyl)cyclohexyl)carbamate (3l)

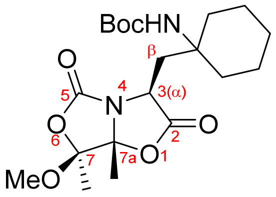

Following the general procedure. Yield after column chromatography (hexanes/ethyl acetate, 8:2): 32 mg, 78%. Light yellow solid. Mp: 84-87. $[\alpha]_D^{20}$ +145.7 (c 1.0, $CHCl_3$). HRMS (ESI) m/z: $[M +Na]^+$ Calcd. for $C_{20}H_{32}N_2NaO_7$ 435.2101; Found 435.2102. ^1H NMR ($CDCl_3$, 400 MHz): δ 4.56 (bs, 1H, N<u>H</u>Boc), 4.41 (d, 1H, J = 11.2 Hz, $H^{3\alpha}$), 3.48 (s, 3H, OMe^{7}), 2.41-2.51 (m, 1H, H^{β}), 2.24-2.36 (m, 1H, H^{cycle}), 1.97-2.09 (m, 2H, H^{β}, H^{cycle}), 1.64 (s, 3H, CH_3^{7a}), 1.61 (s, 3H, CH_3^{7}), 1.53-1.64 (m, 2H, H^{cycle}), 1.34-1.50 (m, 5H, H^{cycle}), 1.40 (s, 9H, 1C(C<u>H</u>$_3$)$_3$), 1.22-1.29 (m, 1H, H^{cycle}). $^{13}C\{^1H\}$ NMR ($CDCl_3$, 100 MHz): δ 173.2, 159.4 (2CO), 154.5 (<u>C</u>(CH$_3$)$_3$), 108.0 (C^{7}), 102.0 (C^{7a}), 57.2 ($C^{3\alpha}$), 53.5 (<u>C</u>NHBoc), 51.5 (OMe^{7}), 38.1 (C^{β}), 34.9 ($2C^{cycle}$), 28.1, 28.4, 28.5 (C(<u>C</u>H$_3$)$_3$), 25.8 (C^{cycle}), 21.9 (CH_3^{7a}), 21.5 (C^{cycle}), 21.3 (C^{cycle}), 16.5 (CH_3^{7}).

2.4.17. *tert*-Butyl (1-((3*S*,7*S*,7a*R*)-7-methoxy-7,7a-dimethyl-2,5-dioxotetrahydro-5*H*-oxazolo[4,3-*b*]oxazol-3-yl)-2-methylpropan-2-yl)carbamate (3m)

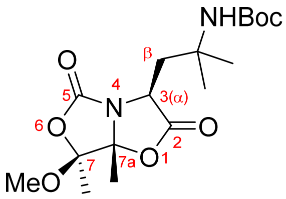

Following the general procedure. Yield after column chromatography (hexanes/ethyl acetate, 8:2): 28 mg, 76%. Light yellow solid. Mp:78-81. $[\alpha]_D^{20}$ +43.8 (c 1.0, $CHCl_3$). HRMS (ESI) m/z: $[M +Na]^+$ Calcd. for $C_{17}H_{28}N_2NaO_7$ 395.1789; Found 395.1793. ^1H NMR ($CDCl_3$, 400 MHz): δ 4.71 (br s, 1H, N<u>H</u>Boc), 4.37 (d, 1H, J = 11.5 Hz, $H^{3\alpha}$), 3.48 (s, 3H, OMe^{7}), 2.45-2.57 (m, 1H, H^{β}), 2.01-2.10 (m, 1H, H^{β}), 1.64 (s, 3H, CH_3^{7a}), 1.62 (s, 3H, CH_3^{7}), 1.43 (s, 3H, 1C(C<u>H</u>$_3$)$_2$), 1.40 (s, 9H, 1C(C<u>H</u>$_3$)$_3$), 1.35 (s, 3H, 1C(C<u>H</u>$_3$)$_2$). $^{13}C\{^1H\}$ NMR ($CDCl_3$, 100 MHz): δ 172.6, 159.1 (2CO), 154.2 (<u>C</u>(CH$_3$)$_3$), 107.7 (C^{7}), 101.7 (C^{7a}), 57.8 ($C^{3\alpha}$), 51.2 (OMe^{7}), 38.1 (C^{β}), 28.2 (4C, 3C(<u>C</u>H$_3$)$_3$, 1CH(<u>C</u>H$_3$)$_2$), 27.6 (1CH(<u>C</u>H$_3$)$_2$), 21.6 (CH_3^{7a}), 16.2 (CH_3^{7}).

2.4.18. (3*S*,7*S*,7a*R*)-3-(hex-5-yn-1-yl)-7-methoxy-7,7a-dimethyldihydro-5*H*-oxazolo[4,3-b]oxazole-2,5(3*H*)-dione (3n)

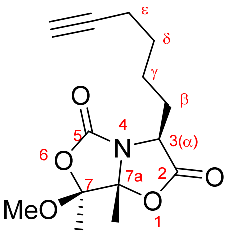

Following the general procedure. Yield after column chromatography (hexanes/ethyl acetate, 8:2): 16 mg, 57%. Light yellow oil. $[\alpha]_D^{20}$ +67.2 (c 1.0, $CHCl_3$). HRMS (ESI) m/z: $[M + H]^+$ Calcd. for $C_{14}H_{20}NO_5$ 282.13360; Found 282.13428. ^1H NMR ($CDCl_3$, 400 MHz): δ 4.27 (dd, 1H, J = 9.7, 4.9 Hz, $H^{3\alpha}$), 3.50 (s, 3H, OMe^7), 2.20-2.26 (m, 2H, CH_2), 2.00-2.08 (m, 1H, $1H^\beta$), 1.95-1.98 (m, 1H, CH^{alkyne}), 2.61-1.78 (m, 5H, $2CH_2$, $1H^\beta$), 1.64 (s, 3H, CH_3^{7a}), 1.62 (s, 3H, CH_3^7). $^{13}C\{^1H\}$ NMR ($CDCl_3$, 100 MHz): δ 172.3, 159.5 (2CO), 108.2 (C^7), 101.6 (C^{7a}), 83.9 (C^{alkyne}), 68.9 (CH^{alkyne}), 60.9 ($C^{3\alpha}$), 51.7 (OMe^7), 31.1 (C^β), 27.5 (CH_2), 25.6 (CH_2), 22.2 (CH_3^{7a}), 18.3 (CH_2), 16.7 (CH_3^7).

2.4.19. (*S*)-2,4-Diaminobutanoic acid dihydrochloride (4c)

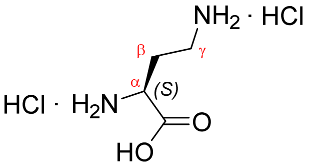

Following the general procedure for hydrolysis. Yield: 93%. $[\alpha]_D^{20}$ +14.2 (c 1.0, 6 M HCl). HRMS (ESI) m/z: $[M - H]^-$ Calcd. for $C_4H_9N_2O_2$ 117.0670; Found 117.0668. ^1H NMR (D_2O, 400 MHz): δ 3.99 (t, 1H, J = 6.5 Hz, H^α), 3.14-3.30 (m, 2H, H^γ), 2.18-2.31 (m, 2H, H^β). $^{13}C\{^1H\}$ NMR (D_2O, 100 MHz): δ 172.0 (CO), 51.4 (C^α), 36.2 (C^γ), 27.8 (C^β).

2.4.20. (*S*)-2,4-Diaminobutanoic-2-*d* acid dihydrochloride (4c-D)

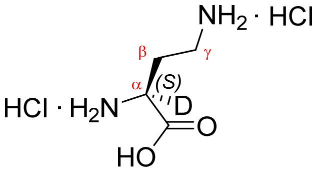

Following the general procedure for hydrolysis. Yield: 85% [89% deuterated]. $[\alpha]_D^{20}$ +13.8 (c 1.0, 6 M HCl). HRMS (ESI) m/z: $[M - H]^+$ Calcd. for $C_4H_{10}DN_2O_2$ 120.0883; Found 120.0877. ^1H NMR (D_2O, 400 MHz): δ 3.90-3.93 (m, 0.11H, H^α), 3.14-3.22 (m, 2H, H^γ), 2.17-2.20 (m, 2H, H^β). $^{13}C\{^1H\}$ NMR (D_2O, 100 MHz): δ 173.0 (CO), 36.4 (C^γ), 27.9 (C^β). C^α is not observed.

2.4.21. (*S*)-2-Amino-4-(phenylselanyl)butanoic acid hydrochloride (4k)

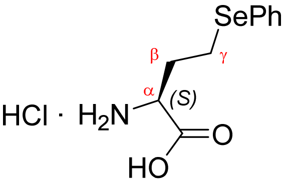

Following the general procedure for hydrolysis. Yield: 93% (27 mg). $[\alpha]_D^{20}$ -2.3 (c 1.0, 6 M HCl). HRMS (ESI) m/z: $[M + H]^+$ Calcd. for $C_{10}H_{14}NO_2Se$ 260.01843; Found 260.01715. ^1H NMR (D_2O, 400 MHz): δ 7.55-7.60 (m, H, CH^{Ar}), 7.32-7.38 (m, H, CH^{Ar}), 3.68 (t, 1H, J = 6.3 Hz, H^α), 2.99 (t, 2H, J = 7.9 Hz, H^γ), 2.03-2.19 (m, 2H, H^β). $^{13}C\{^1H\}$ NMR (D_2O, 100 MHz): δ 170.1 (CO), 132.4 ($2C^{Ar}$), 129.4 ($3C^{Ar}$), 129.1(C^{*Ar}), 54.9 (C^α), 32.1 (C^β), 22.0 (C^γ).

2.4.22. Benzyl ((*S*)-2-((4*R*,5*S*)-4-hydroxy-5-methoxy-4,5-dimethyl-2-oxooxazolidin-3-yl)-4-(phenylselanyl)butanoyl)-L-phenylalaninate (5k)

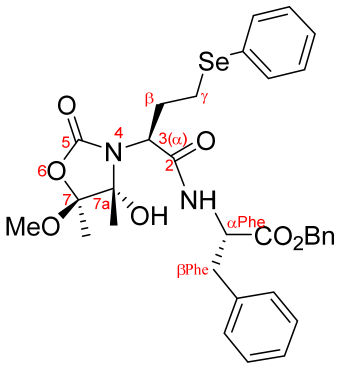

Following the general procedure for aminolysis with amino ester hydrochlorides. Yield after column chromatography (hexanes/ethyl acetate, 7:3): 78 mg, 74%. Sticky foam. $[\alpha]_D^{20}$ +18.4 (c 1.0, $CHCl_3$). HRMS (ESI) m/z: $[M + Na]^+$ Calcd. for $C_{32}H_{36}N_2NaO_7Se$ 663.1585; Found 663.1609. ^{1}H NMR ($CDCl_3$, 400 MHz): δ 7.38-7.42 (m, 2H, CH^{Ar}), 7.27-7.30 (m, 2H, CH^{Ar}), 7.20-7.24 (m, 2H, CH^{Ar}), 7.16-7.19 (m, 3H, CH^{Ar}), 7.11-7.16 (m, 3H, CH^{Ar}), 6.93-6.97 (m, 2H, CH^{Ar}), 6.82 (d, 1H, J = 7.9 Hz, NH), 5.08 (d, 1H, J = 12.0 Hz, $1CH_2^{OBn}$), 5.01 (d, 1H, J = 12.0 Hz, $1CH_2^{OBn}$), 4.81 (ddd, 1H, J = 7.9, 5.5, 5.5 Hz $H^{\alpha Phe}$), 4.34 (dd, 1H, J = 9.5, 5.9 Hz, $H^{3\alpha}$), 4.28 (br s, 1H, OH), 3.12 (s, 3H, OMe^7), 3.01 (t, 2H, J = 5.5 Hz, $CH_2^{\beta Phe}$), 2.88-2.97 (m, 1H, CH_2^{γ}), 2.75-2.83 (m, 1H, CH_2^{γ}), 2.36-2.48 (m, 1H, H^{β}), 2.20-2.31 (m, 1H, H^{β}), 1.44 (s, 3H, CH_3^7), 1.36 (s, 3H, CH_3^{7a}). $^{13}C\{^1H\}$ NMR ($CDCl_3$, 100 MHz): δ 170.8, 170.8, 155.9 (2CO), 135.3, 135.1, 129.9 ($3C^{*Ar}$), 133.2 - 127.3 ($15C^{Ar}$), 107.9 (C^7), 91.0 (C^{7a}), 67.5 (CH_2^{OBn}), 56.5 ($C^{3\alpha}$), 53.8 ($C^{\alpha Phe}$), 50.6 (OMe^7), 38.0 ($C^{\beta Phe}$), 29.6 (C^{β}), 24.9 (C^{γ}), 20.4 (CH_3^{7a}), 14.1 (CH_3^7).

2.4.23. ((*S*)-2-Amino-4-(phenylselanyl)butanoyl)-L-phenylalanine (6k)

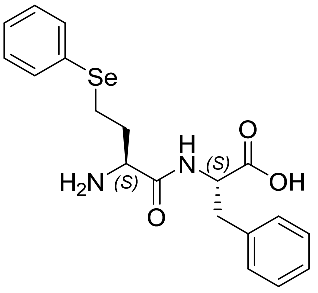

Following the general procedure for hydrolysis. Yield: 95%. $[\alpha]_D^{20}$ -12.3 (c 1.0, 6 M HCl). HRMS (ESI) m/z: $[M - H]^+$ Calcd. for $C_{19}H_{23}N_2O_3Se$ 407.0874; Found 407.0878. ^{1}H NMR (D_2O, 400 MHz): δ 7.20-7.60 (m, 10H, CH^{Ar}), 3.86 (dd, 1H, J = 7.9, 5.2 Hz, $H^{\alpha Phe}$), 3.69 (t, 1H, J = 6.7 Hz, H^{α}), 3.16 (dd, 1H, J = 14.5, 5.2 Hz, $1H^{\beta Phe}$), 3.00 (dd, 1H, J = 14.5, 7.9 Hz, $1H^{\beta Phe}$), 2.90 (t, 2H, J = 7.8 Hz, H^{γ}), 1.80-2.15 (m, 2H, H^{β}). $^{13}C\{^1H\}$ NMR (D_2O, 100 MHz): δ 174.2, 174.2 (2CO), 135.1 (C^{*Phe}), 132.5 ($2C^{Ar}$), 132.2 (C^{*Se}), 129.4, 129.3, 129.2, 129.0 128.6, 128.4, 127.6, 127.5 ($8C^{Ar}$), 56.0 ($C^{\alpha Phe}$), 54.6 (C^{α}), 36.4 ($C^{\beta Phe}$), 31.2 (C^{β}), 21.8 (C^{γ}).

2.5. Synthesis of Dha 1 in a gram scale

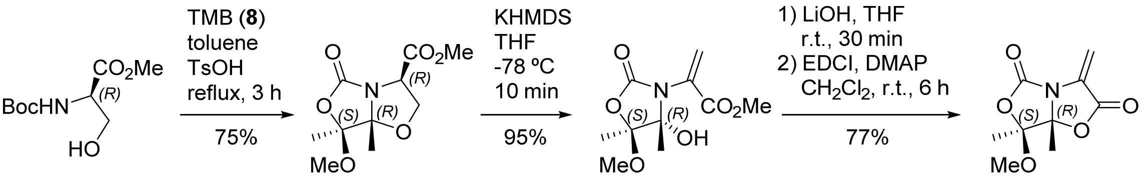

N-Boc-D-Ser-OMe **7** Bicyclic *N,O*-acetal **2b** 1st generation chiral Dha **9** 2nd generation chiral Dha **1**

The first generation chiral Dha **9** was obtained in a gram scale, following the procedure previously published,[S5] starting from (*R*)-*N*-Boc-serine methyl ester (N-Boc-D-Ser-OMe, **7**) and using two steps. A round-bottomed flask was charged with *N*-Boc-D-Ser-OMe **7** (11.18 g, 45.58 mmol), 2,2,3,3-tetramethoxybutane **8** (TMB, 16.26 g, 91.19 mmol), toluene (300 mL) and $TsOH\cdot H_2O$ (0.86 g, 4.57 mmol). The solution was heated under reflux and stirred for 3 h. The reaction mixture was cooled to room temperature, diluted with diethyl ether (100 mL) and then quenched with saturated $NaHCO_3$ (100 mL). The aqueous phase was extracted with diethyl ether (2 × 80 mL). The organic layers were combined, washed with brine, and dried over anhydrous Na_2SO_4. The solvent was evaporated and the

crude product purified by column chromatography (hexane/ethyl acetate, 9:1) to give the corresponding bicyclic *N,O*-acetal **2b** as a yellow oil (9.37 g, 31.91 mmol); yield: 75%. $[\alpha]_D^{25}$ = +120.2 (*c* = 1.27 in $CHCl_3$). (ESI^+) m/z: 246.1. Elemental analysis Calcd. (%) for $C_{10}H_{15}NO_6$: C 48.98, H 6.17, N 5.71; Found: C 48.91, H 6.15, N 5.69. ^{1}H NMR ($CDCl_3$, 400 MHz): δ 4.77 (dd, 1H, *J* = 5.9, 9.0 Hz, CH), 4.30 (t, 1H, *J* = 8.9 Hz, CH_2), 4.15 (dd, 1H, *J* = 5.9, 8.8 Hz, CH_2), 3.82 (s, 3H, CO_2CH_3), 3.47 (s, 3H, OCH_3), 1.58 (s, 3H, CH_3), 1.36 (s, 3H, CH_3). ^{13}C{^{1}H} NMR ($CDCl_3$, 100 MHz): δ 170.5 ($\mathit{C}O_2CH_3$), 160.6 (NCO_2), 107.1 ($\mathit{C}CH_3OCH_3$), 101.5 ($\mathit{C}NCH_3OCH_2$), 66.7 (CH_2), 59.9 (CH), 52.9 ($CO_2\mathit{C}H_3$), 51.0 (OCH_3), 16.2 (CH_3), 15.5 (CH_3).

This bicyclic *N,O*-acetal **2b** (2.8 g, 11 mmol) was dissolved in dry THF (250 mL) under argon atmosphere. The solution was cooled to –78 ºC and stirred. A 0.91 M solution of KHMDS in THF (18 mL, 17 mmol) was added with a syringe (KHMDS was added slowly). After 10 min, the reaction was quenched with a NH_4Cl saturated solution (250 mL). The mixture was heated to room temperature in a warm water bath and vigorously stirred. The crude was diluted with diethyl ether and the aqueous phase was extracted with more diethyl ether. Organic layers were combined, washed with brine, and dried over anhydrous Na_2SO_4. The solvent was evaporated and the crude was purified by a silica gel column chromatography (hexane/ethyl acetate, 6:4) to give the first generation Dha **9**, as a colourless oil (2.6 g, 10 mmol); yield: 95%. $[\alpha]_D^{25}$ = +122.7 (*c* 1.10, $CHCl_3$). ^{1}H NMR ($CDCl_3$, 400 MHz): δ (ppm) 6.57 (s, 1H, C=CH_2), 5.99 (s, 1H, C=CH_2), 4.71 (s, 1H, OH), 3.87 (s, 3H, CO_2CH_3), 3.44 (s, 3H, OCH_3), 1.62 (s, 3H, CH_3), 1.36 (s, 3H, CH_3). ^{13}C{^{1}H} NMR ($CDCl_3$, 100 MHz): δ (ppm) 166.1 ($\mathit{C}O_2CH_3$), 154.9 (NCO_2), 131.8 (*C*=CH_2), 129.1 (C=$\mathit{C}H_2$), 108.7 ($\mathit{C}CH_3OCH_3$), 90.8 ($\mathit{C}NCH_3OH$), 53.2 ($CO_2\mathit{C}H_3$), 50.7 (OCH_3), 19.6 (CH_3), 14.6 (CH_3). (ESI^+) m/z: 246.1. Elemental analysis Calcd. (%) for $C_{10}H_{15}NO_6$: C 48.98, H 6.17, N 5.71; Found: C 49.03, H 6.15, N 5.77.

The second generation Dha **1** was obtained from the first generation Dha **9** (2.08 g, 8.48 mmol) through a basic hydrolysis followed by an internal coupling (lactonization).[S1] This compound (**9**) was dissolved in THF (30 mL) and the solution was cooled to 0 °C. Then, LiOH·H_2O (1.78 mg, 42.41 mmol) was dissolved in H_2O (30 mL) and was introduced in the flask. After the mixture was stirred at this temperature for 30 min, a 2 M aqueous HCl solution (21 mL, 42.47 mmol) was added in order to acidify the mixture. After that, the reaction crude was diluted with diethyl ether and the aqueous layer was extracted with ethyl acetate. The combined organic layers were washed with brine and dried with anhydrous Na_2SO_4. The solvent was filtered and evaporated to give acid derivative of **9** as a white solid. The crude was dissolved in a Schlenk in anhydrous CH_2Cl_2 (70 mL). Then, 1-ethyl-3-(3-dimethylaminopropyl)carbodiimide) (EDCI) (1.78 g, 9.33 mmol) and 4-dimethylaminopyridine (DMAP) (1.24 g, 10.17 mmol), as coupling agents, were added under an inert atmosphere. The mixture was stirred at room temperature for 6 h and then, a 0.5 M aqueous HCl solution (70 mL) was added. The aqueous layer was extracted with CH_2Cl_2. The solution was evaporated and the residue was purified by a silica gel column chromatography (hexane/ethyl acetate, 7:3) to give Dha **1** (1.39 g, 6.52 mmol) as a white crystalline solid with a 77% yield. $[\alpha]_D^{25}$ (1.06, $CHCl_3$) = +107.4. Mp: 103-106 °C. HRMS (ESI) m/z: $[M+Na]^+$ Calcd. for $C_9H_{11}NO_5Na$ 236.0529; Found 236.0531. ^{1}H NMR ($CDCl_3$, 400 MHz): δ (ppm) 5.97 (s, 1H, $C\mathit{H_2}$), 5.84 (s, 1H, $C\mathit{H_2}$), 3.59 (s, 3H, OMe), 1.64 (s, 3H, Me), 1.63 (s, 3H, Me). ^{13}C{^{1}H} NMR ($CDCl_3$, 100 MHz): δ (ppm) 163.1 (*C3*=CH_2), 153.2 (*C5*=O), 130.1 (*C2*=O), 110.1 (*C7*), 109.7 (*C*H_2), 98.5 (*C7a*), 52.4 (OMe), 21.9 (Me), 17.5 (Me).

[S5] Aydillo, C.; Jiménez-Osés, G.; Busto, J. H.; Peregrina, J. M.; Zurbano, M. M.; Avenoza, A. *Chem. Eur. J.* **2007**, *13*, 4840.

3. X-Ray data for compound 3k and 3b

Details of the X-ray analyses are summarized in Table S3. Compound **3k** was dissolved in dichloromethane and *n*-hexane was added carefully creating an interphase. The colorless crystal needle was obtained after 24 h by slow diffusion at 4 °C. Compound **3b** was dissolved in dichloromethane and crystal needle was obtained at 4 °C after three days. The formed crystals were analyzed by X-ray diffraction. The diffraction data were collected using graphite-monochromatic Mo-K_{α} radiation with a Bruker APEX-II diffractometer at a temperature of 110 K using the APEX3 software. The absorption correction was performed using MULTI-SCAN.[S2] The structures were solved with the WINGX program suite[S3] and refined by full-matrix least squares with SHELXL.[S4] Hydrogen atoms were located by mixed methods (electron-density maps and theoretical positions).

[S2] Blessing, R. H. *Acta Crystallogr.* **1995**, *A51*, 33-38.
[S3] Farrugia, L. J. *Appl. Crystallogr.* **2012**, *45*, 849-854.
[S4] Sheldrick, G. *Acta Crystallogr., Sect. C* **2015**, *71*, 3-8.

Table S3. X-ray crystallographic data for compounds **3k** and **3b**

	**3k**	**3b**
**Empirical formula**	$C_{16} H_{19} N O_5 Se$	$C_{19} H_{26} N_2 O_{11}$
**F_w**	384.28	458.42
**T (K)**	298(2)	140 (2)
**Wavelength (Å)**	0.71076	0.71076
**Crystal system**	Orthorhombic	Monoclinic
**Space group**	P 21 21 21	P 21
**Crystal size (mm^3)**	0.23 x 0.06 x 0.06	0.32 x 0.18 x 0.09
**a (Å)**	5.9899(2)	6.7546(4)
**b (Å)**	13.5860(5)	15.3873(7)
**c (Å)**	20.6060(7)	9.8942(6)
**α (°)**	90	90
**β (°)**	90	92.451(2)
**γ (°)**	90	90
**V (Å^3)**	1676.89(10)	1027.41(10)
**Z**	4	2
**D_{calcd} (Mg/m3)**	1.522	1.482
**Absorption coefficient (mm^{-1})**	2.263	0.123
**F(000)**	784	484
**θ range for data collection (deg)**	3.158 to 27.902	2.449 to 27.928
**Index ranges**	-7<=h<=7, -17<=k<=17, -27<=l<=27	-8<=h<=8, -20<=k<=20, -13<=l<=13
**Reflections collected**	69184	63818
**Independent reflections**	3995 [R(int) = 0.0839]	4892 [R(int) = 0.0363]
**Data / restraints/ parameters**	3995 / 0 / 208	4892 / 1 / 289
**Goodness-of-fit on F^2 [a]**	1.126	1.054
**Final R index**	R_1 = 0.0522	R_1 = 0.0278
**[I > 2σ(I)][a]**	wR_2 = 0.1022	wR_2 = 0.0690
**R indexes (all data)[a]**	R_1 = 0.0668, wR_2 = 0.1067	R_1 = 0.0295, wR_2 = 0.0700

**Largest diff. peak and hole (e. Å^{-3})**	0.555 and -0.364	0.227 and -0.182

[a] $R_1 = \sum(|F_o| - |F_c|)/\sum|F_o|$; $wR_2 = [\sum w(F_o^2 - F_c^2)^2/\sum wF_o^2]^{1/2}$; goodness of fit = $\{\sum[w(F_o^2 - F_c^2)^2]/(N_{obs} - N_{param})\}^{1/2}$;$w=[\sigma^2(F_o)+(g_1P)^2 + g_2P]^{-1}$; $P = [\max(F_o^2 ;0 + 2F_c^2)/3$.

Accession Codes

CCDC 2173824 – 2173825 contain the supplementary crystallographic data for this paper. These data can be obtained free of charge via www.ccdc.cam.ac.uk/data_request/cif, or by emailing data_request@ccdc.cam.ac.uk, or by contacting The Cambridge Crystallographic Data Centre, 12 Union Road, Cambridge CB2 1EZ, UK; fax: +44 1223 336033

Figure S2. ORTEP3 diagram of compound **3k** obtained by X-ray diffraction analysis showing thermal ellipsoids at the 75% probability level.

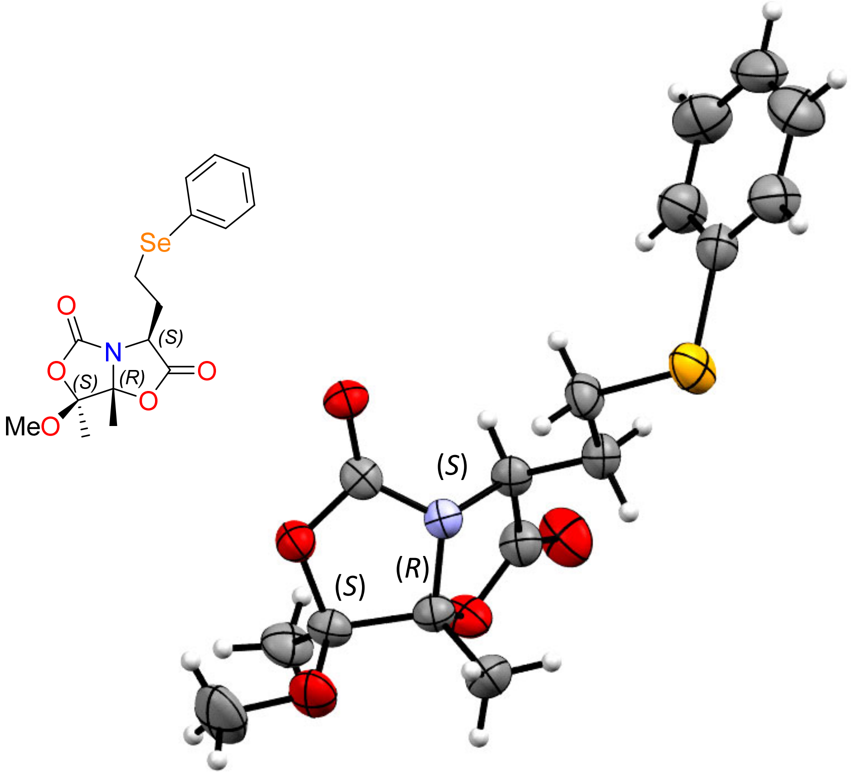

Figure S3. ORTEP3 diagram of compound **3k** obtained by X-ray diffraction analysis showing thermal ellipsoids at the 75% probability level.

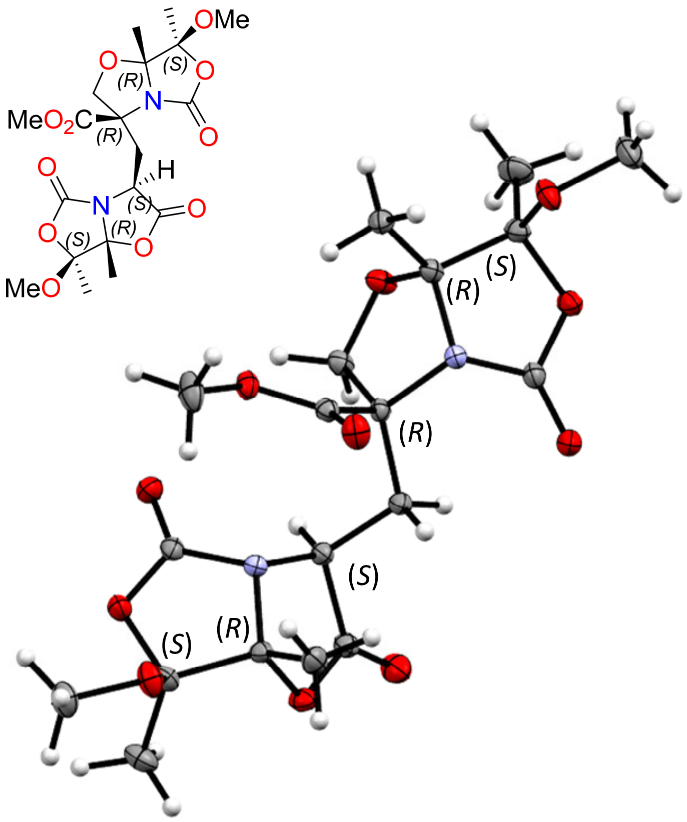

5. NOESY spectra to determine the absolute configurations.

The absolute configuration of the new stereocenter (C3) of compound **3a** formed in the *C*-Michael addition was also determined by a 2D NOESY−NMR experiment on **3a**. The NOE contact between one hydrogen of the –C3-**CH₂**-CH− (Hβ) and the –CH₃-C7a– (Me7a) units in the rigid bicyclic system indicates that these two groups are on the same face of the molecule. Therefore, we can conclude that C3 displays an *S*-configuration in compound **3a**. The same feature occurs in adducts **3a-D** and **3b** obtained in the Michael reactions, as well as in adducts **3c-m** obtained from the Giese reactions. In addition, these structural features were confirmed by X-ray analysis of monocrystals of compounds **3b** and **3k**.

NOESY in $CDCl_3$ (400 MHz) **3a**

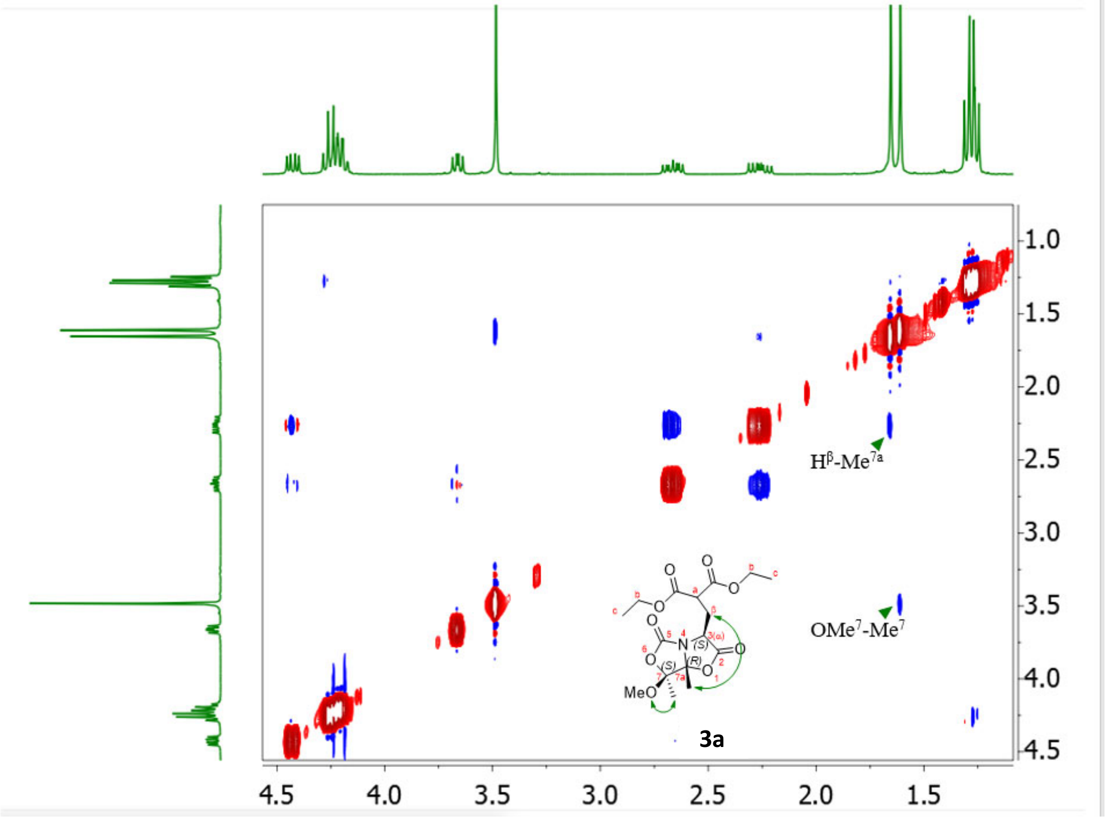

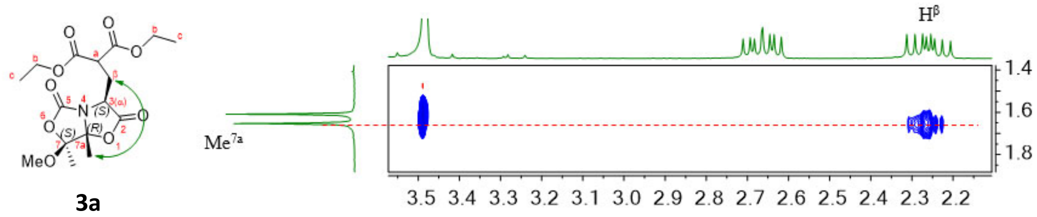

NOESY in $CDCl_3$ (400 MHz) **3a-D**

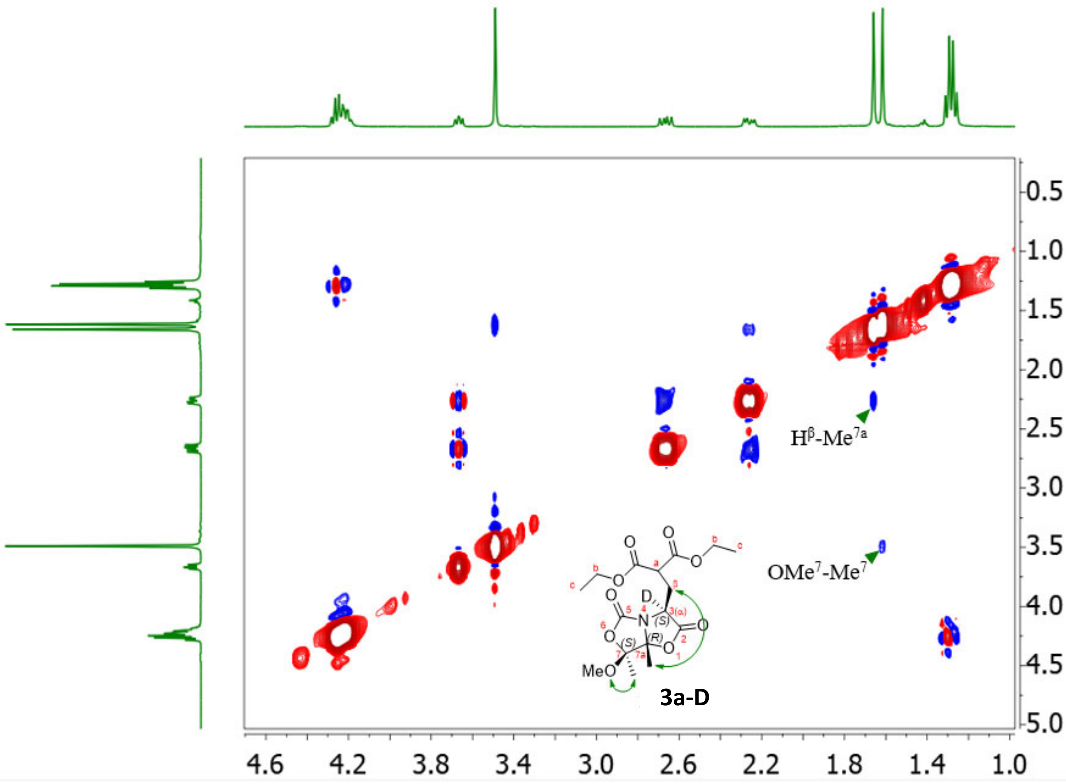

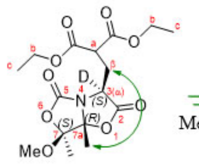

3a-D

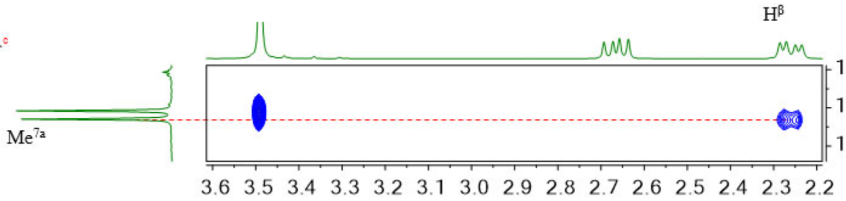

NOESY in $CDCl_3$ (400 MHz) **3b**

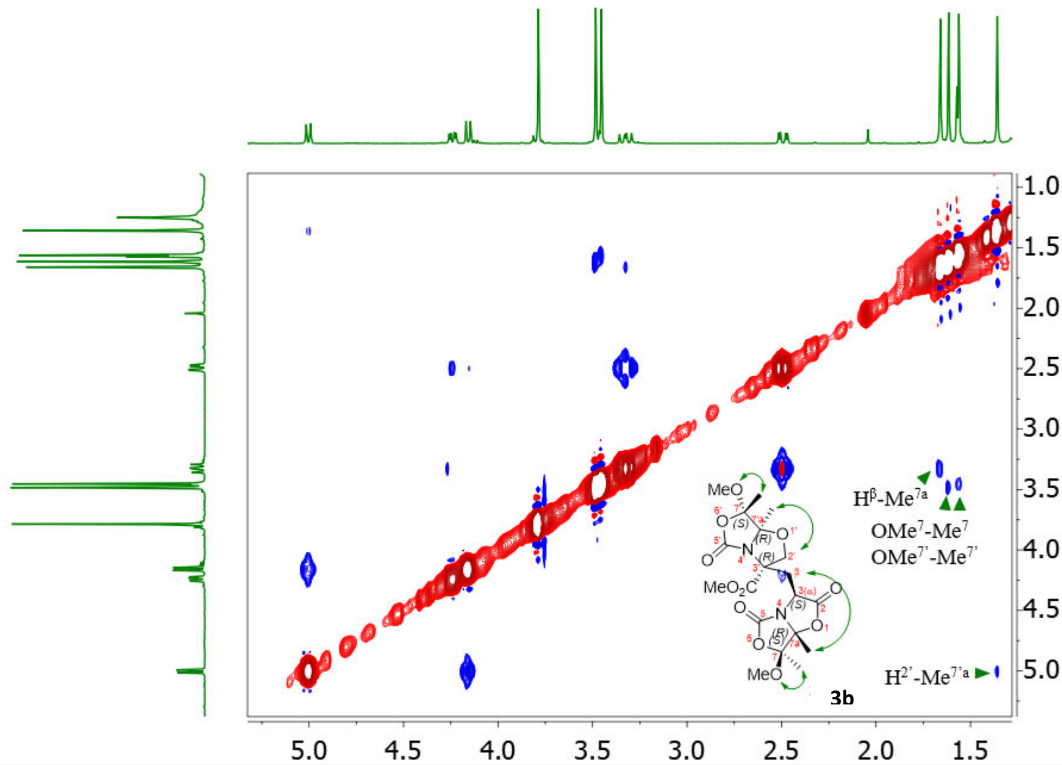

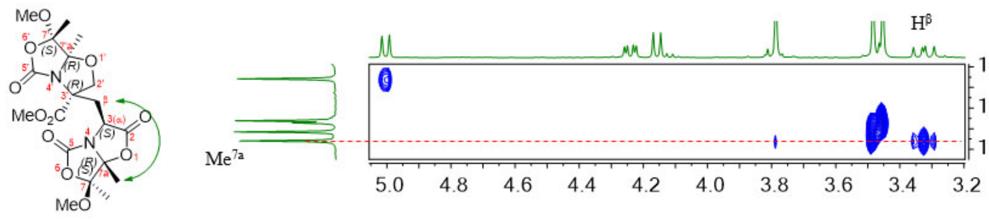

NOESY in $CDCl_3$ (400 MHz) **3c**

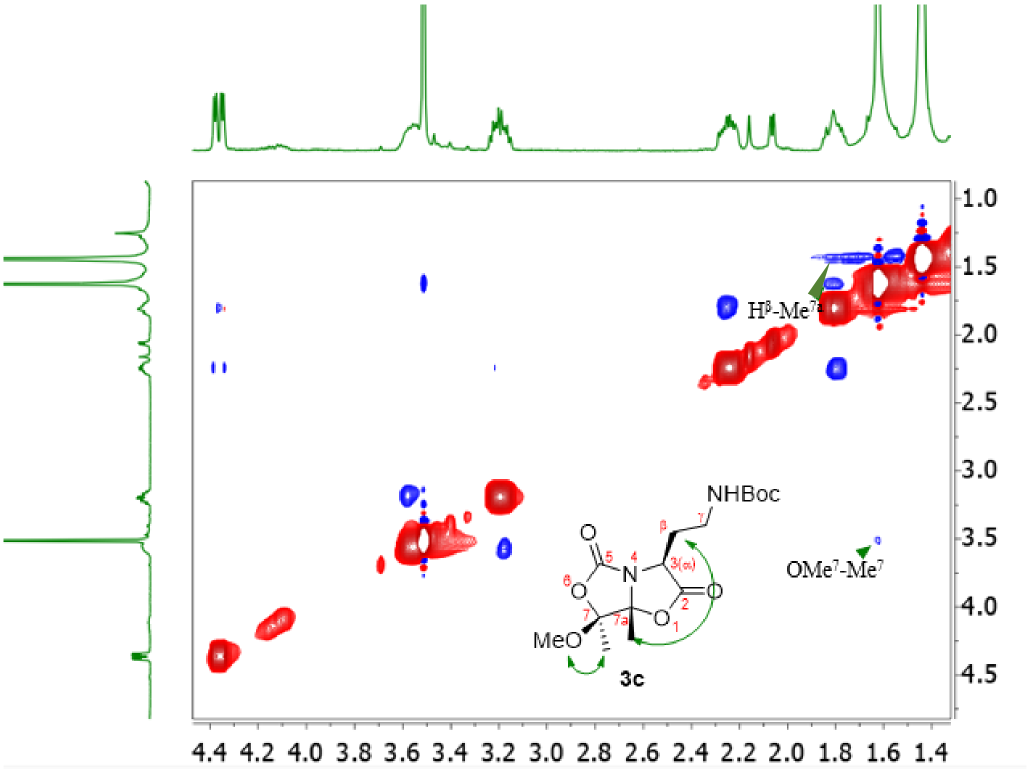

NOESY in $CDCl_3$ (400 MHz) **3d**

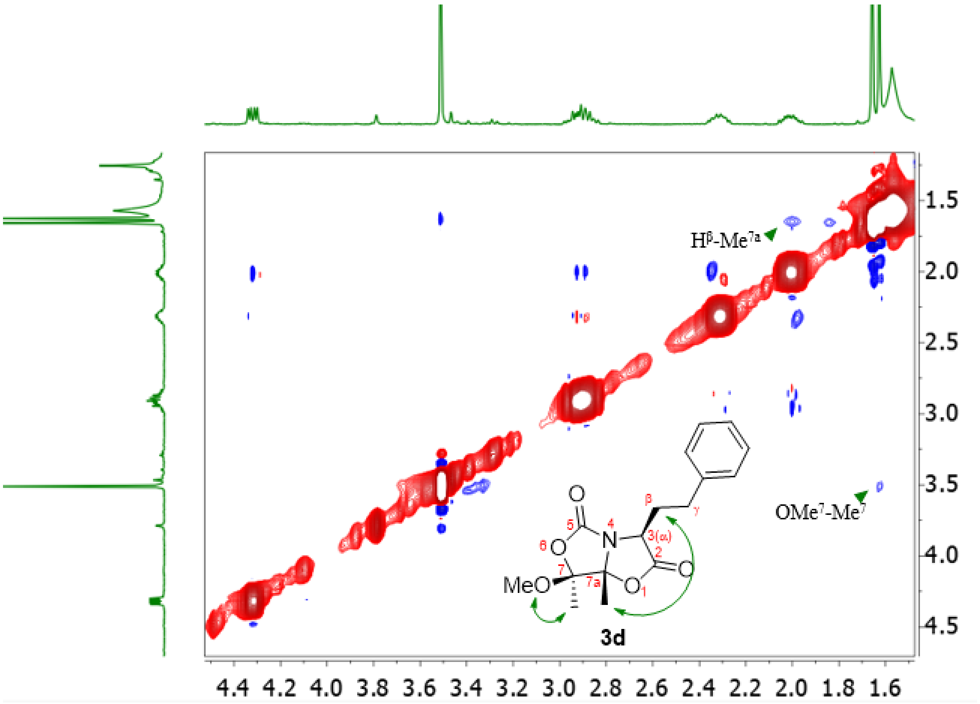

NOESY in $CDCl_3$ (400 MHz) **3e**

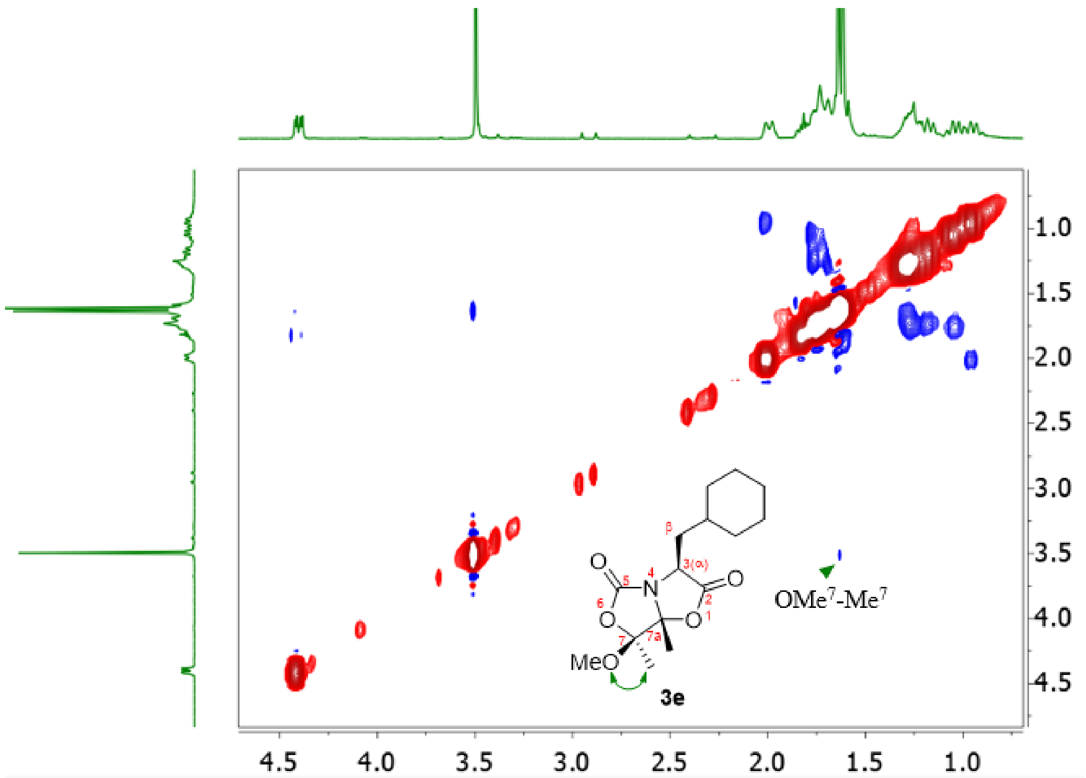

NOESY in $CDCl_3$ (400 MHz) **3f**

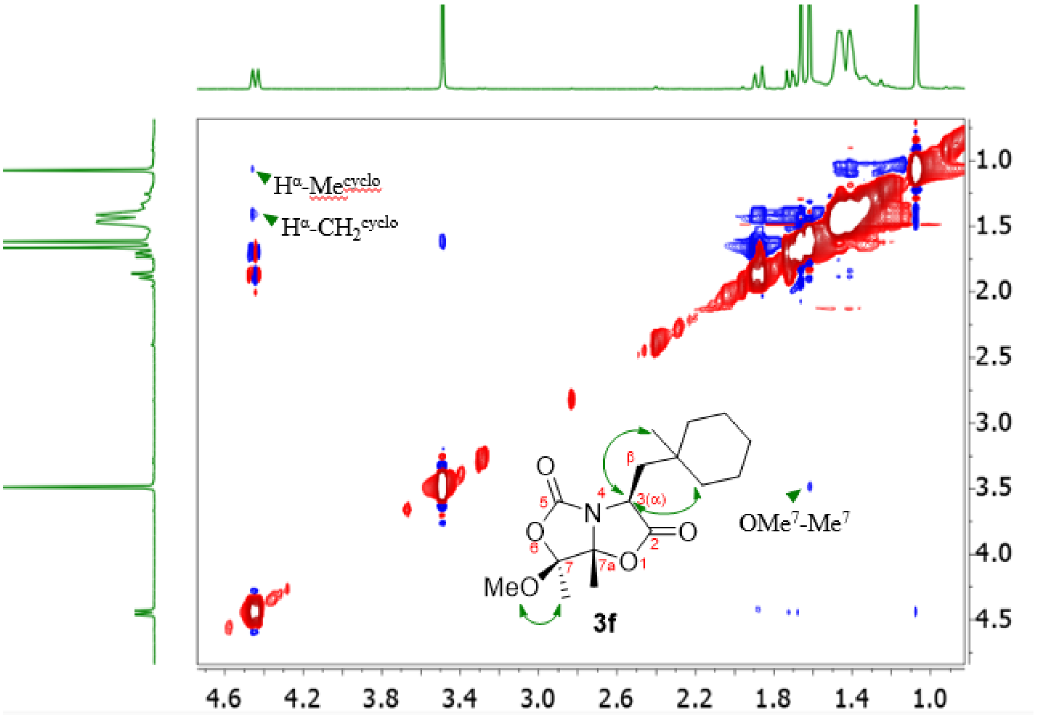

NOESY in $CDCl_3$ (400 MHz) **3g**

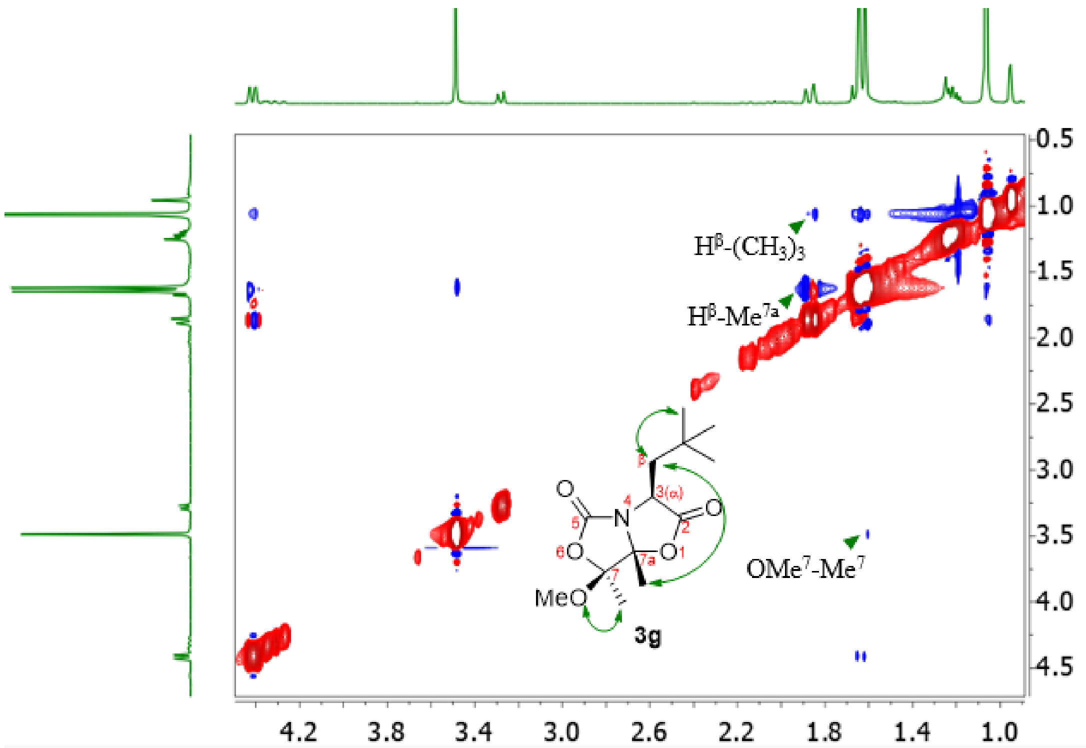

NOESY in $CDCl_3$ (400 MHz) **3h**

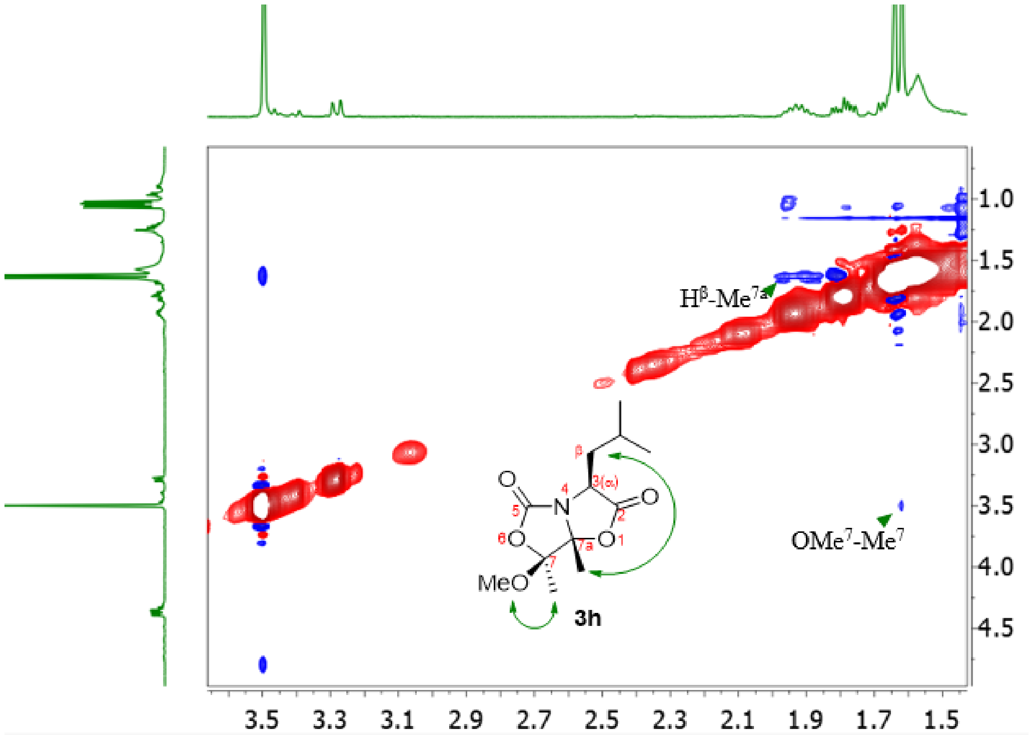

NOESY in $CDCl_3$ (400 MHz) **3i**

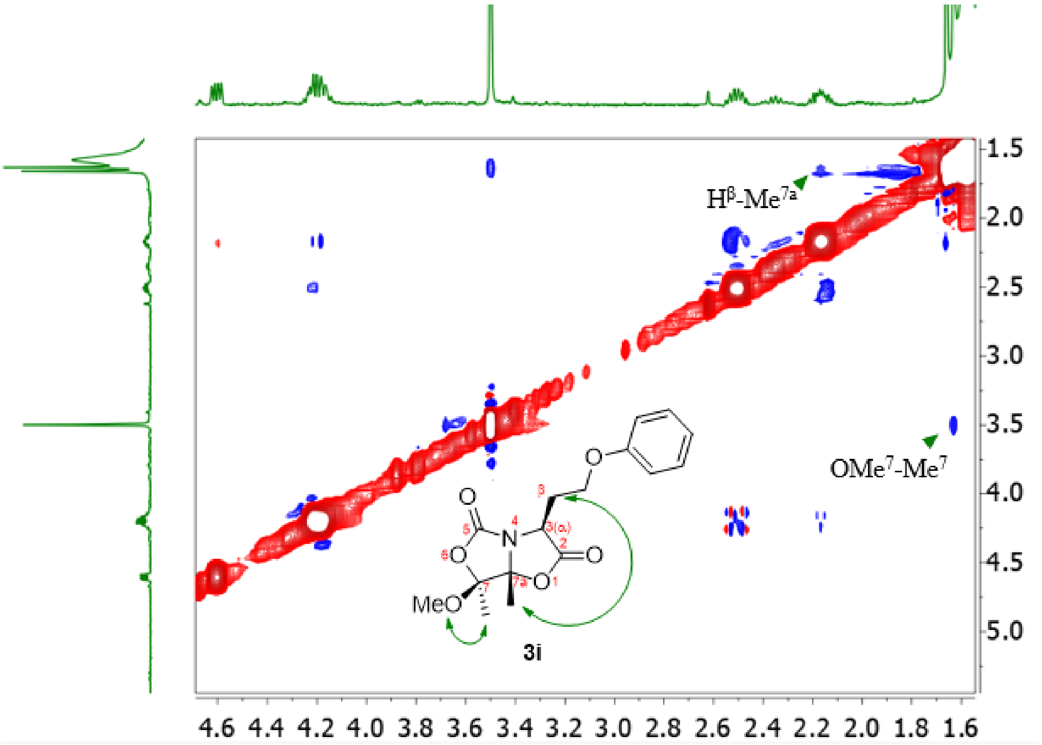

NOESY in $CDCl_3$ (400 MHz) **3j**

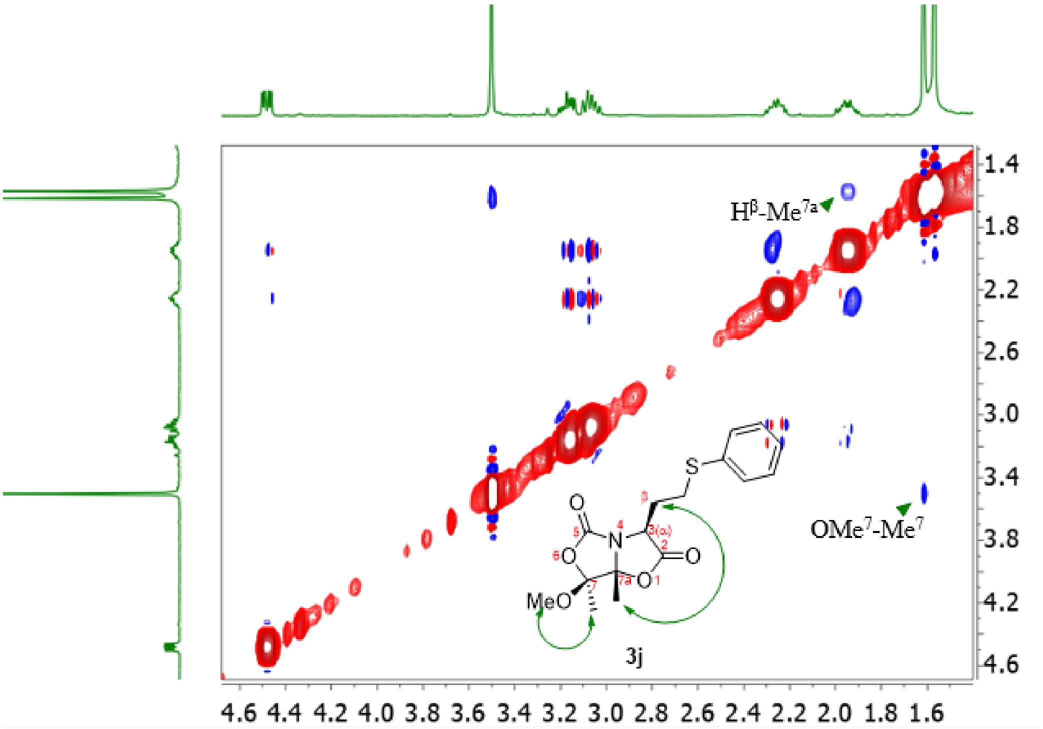

NOESY in $CDCl_3$ (400 MHz) **3k**

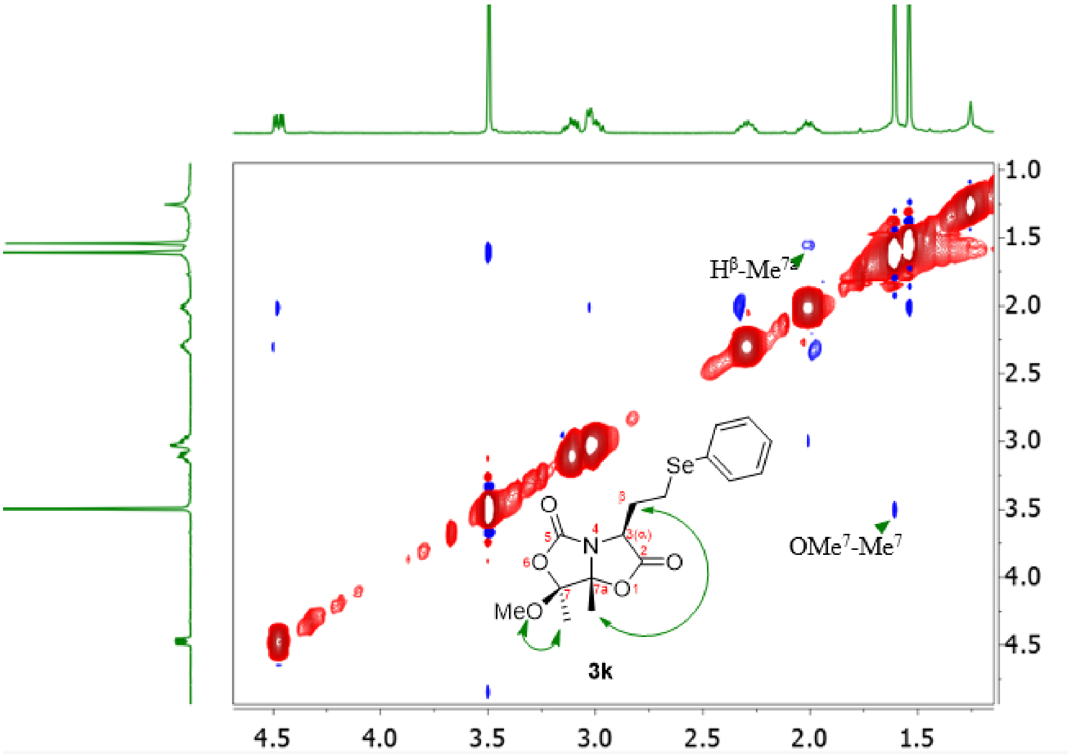

NOESY in $CDCl_3$ (400 MHz) **3l**

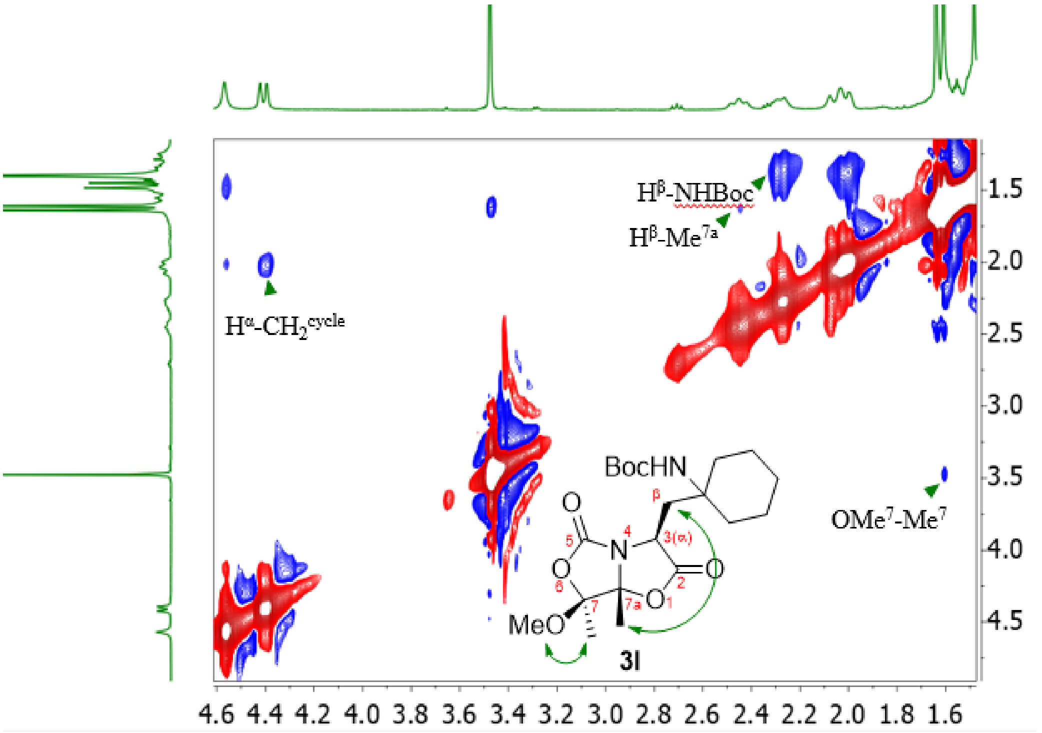

NOESY in $CDCl_3$ (400 MHz) **3m**

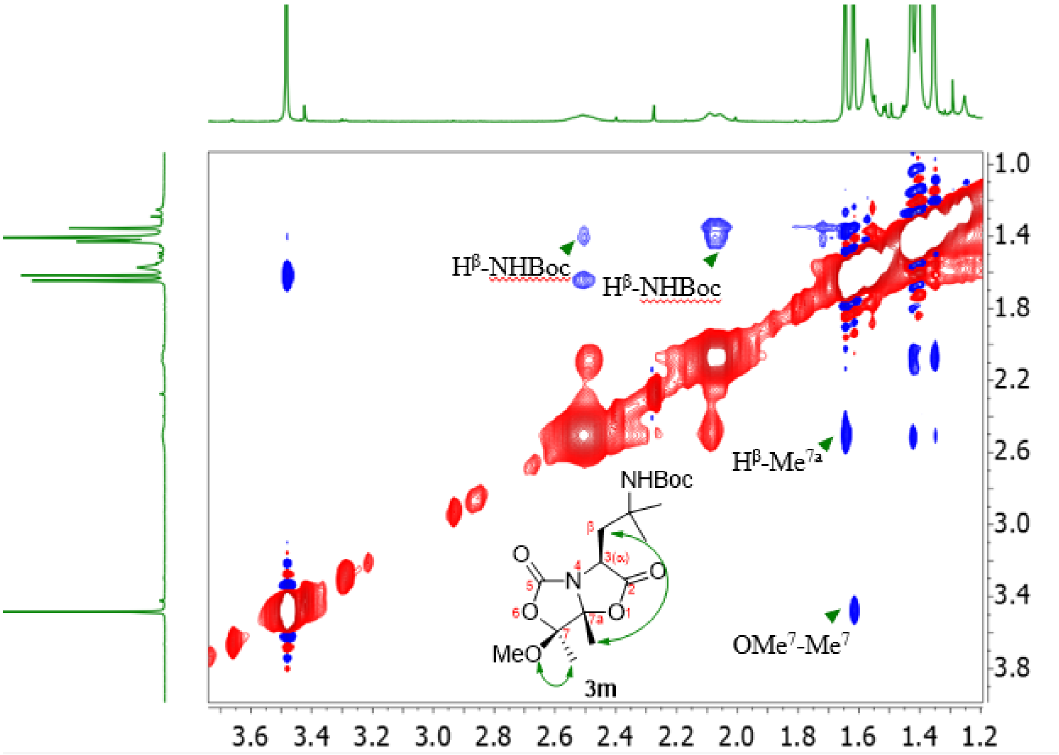

5. NMR spectra

^{1}H NMR in $CDCl_3$ (400 MHz) **2k**

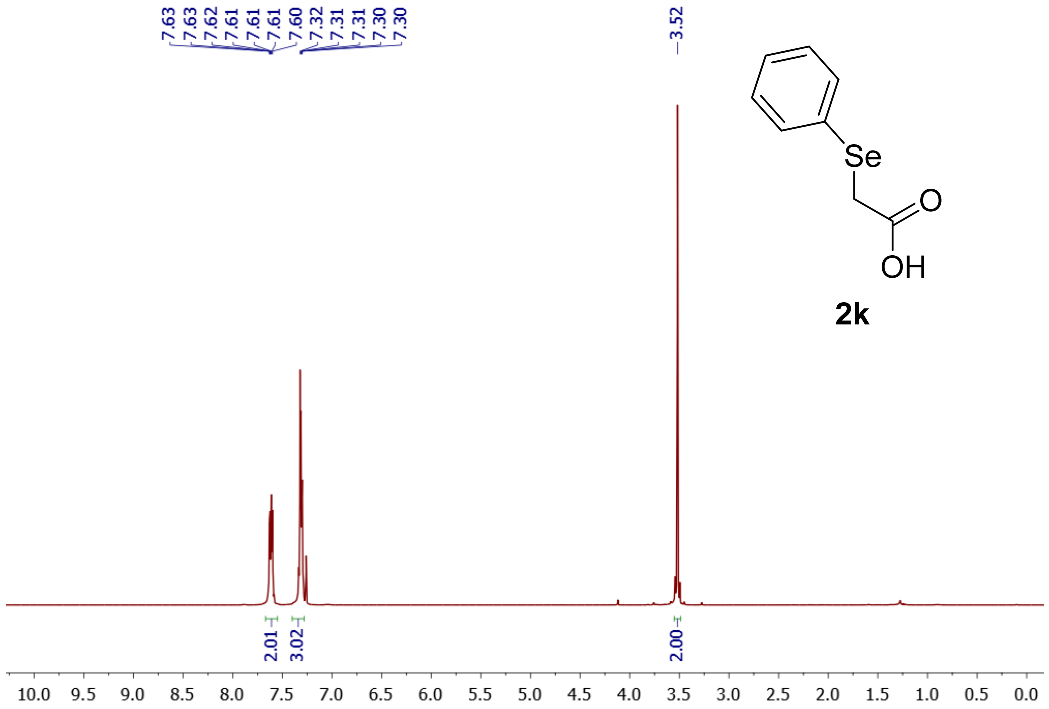

^{1}H NMR in $CDCl_3$ (400 MHz) **7**

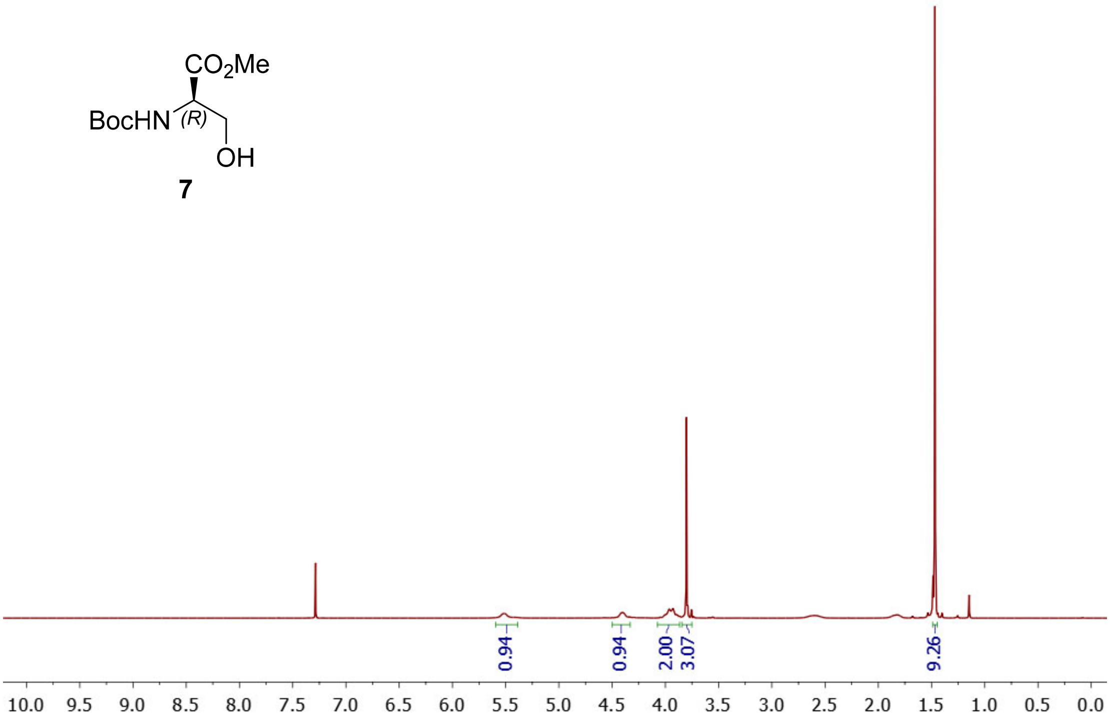

$^{13}C\{^{1}H\}$ NMR in $CDCl_3$ (100 MHz) **7**

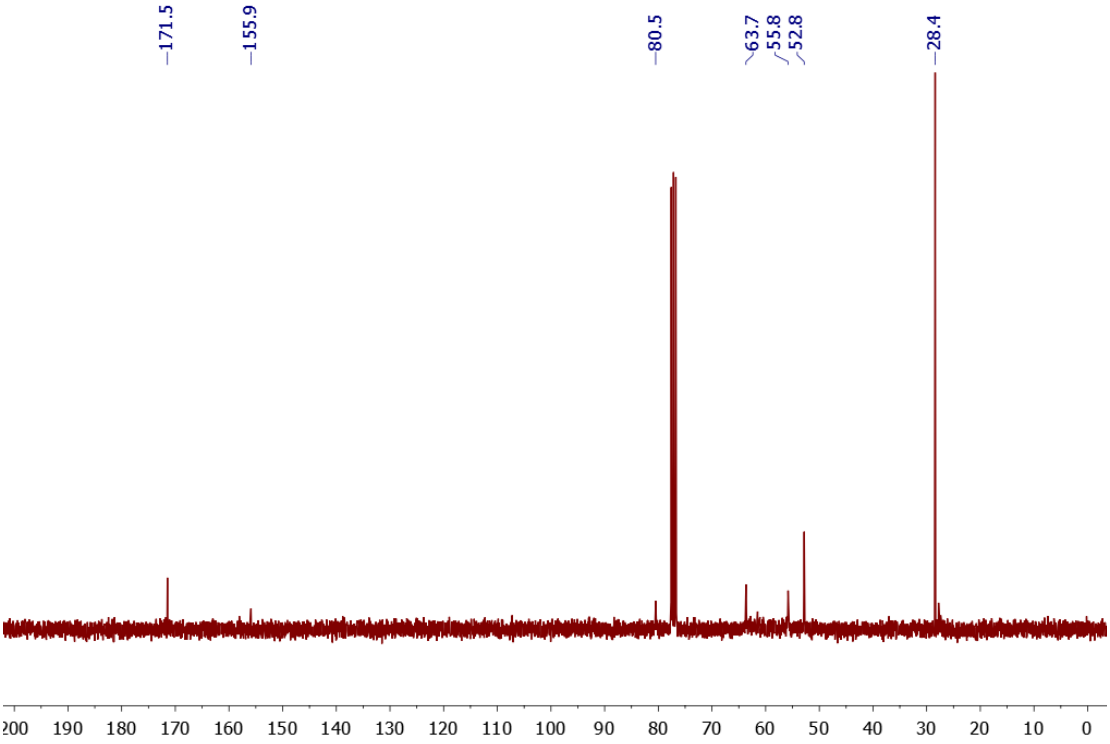

^{1}H NMR in $CDCl_3$ (400 MHz) **2b**

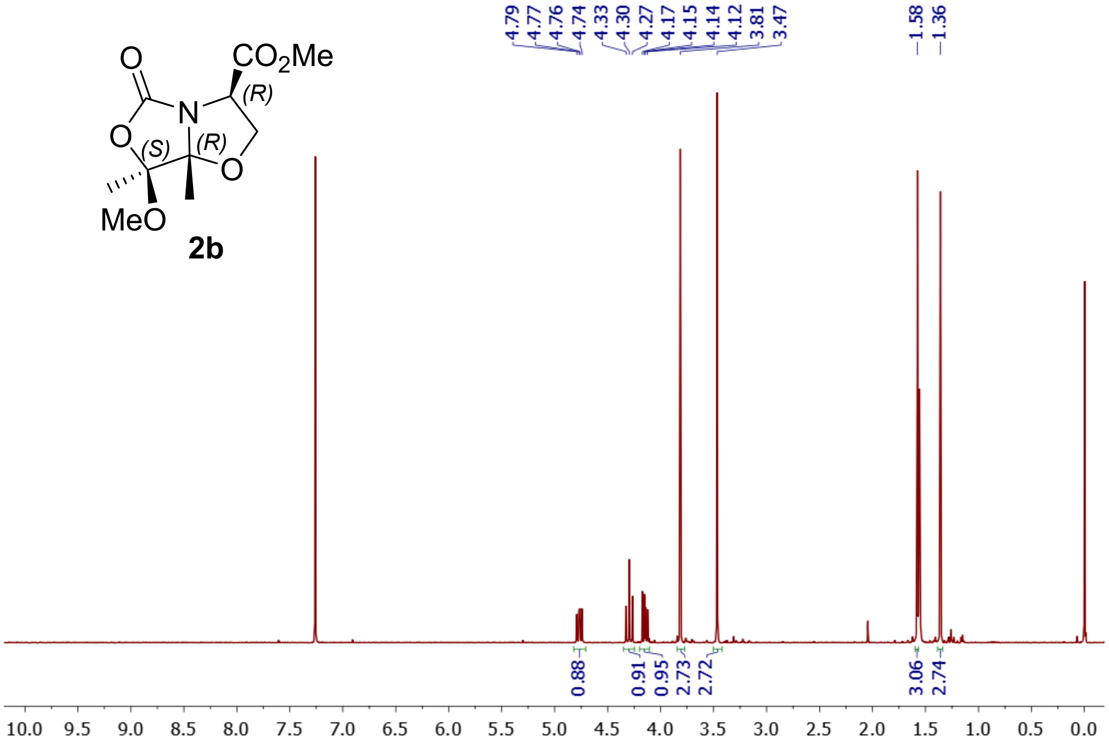

2b

$^{13}C\{^{1}H\}$ NMR in $CDCl_3$ (100 MHz) **2b**

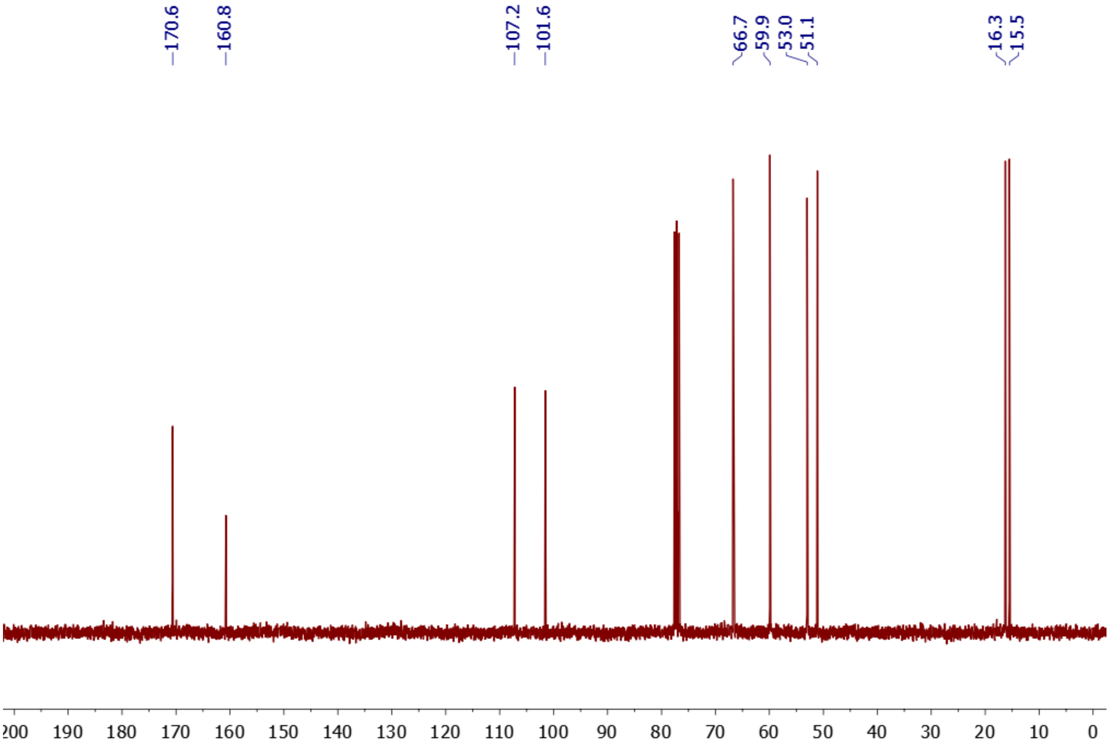

^{1}H NMR in $CDCl_3$ (400 MHz) **9**

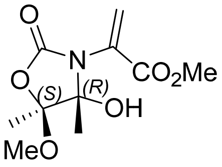

9

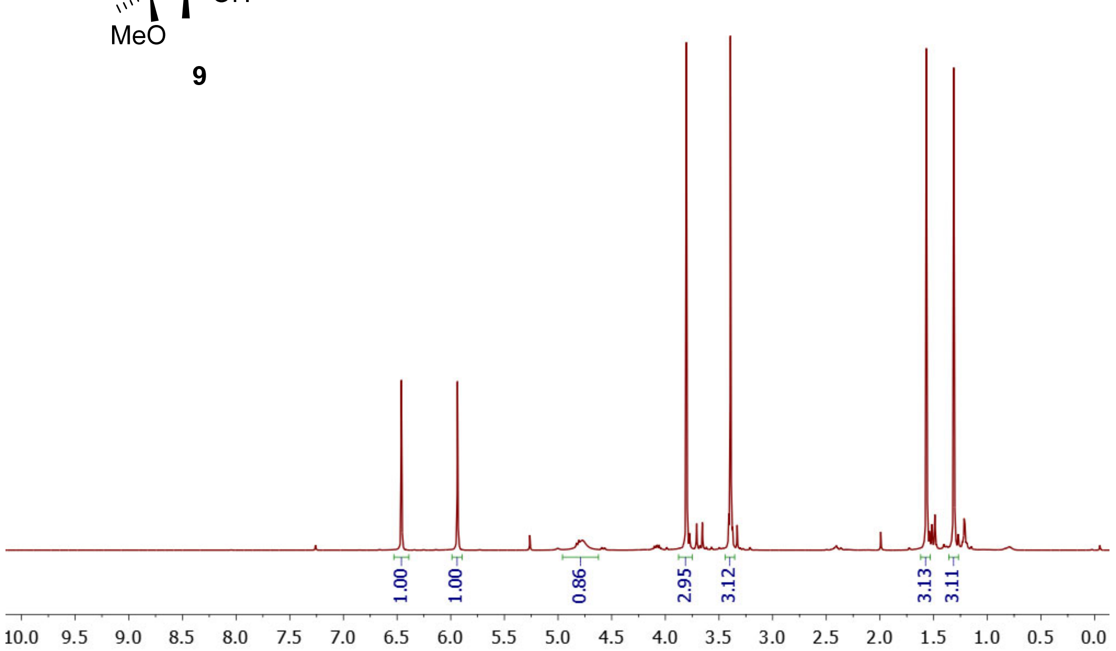

$^{13}C\{^{1}H\}$ NMR in $CDCl_3$ (100 MHz) **9**

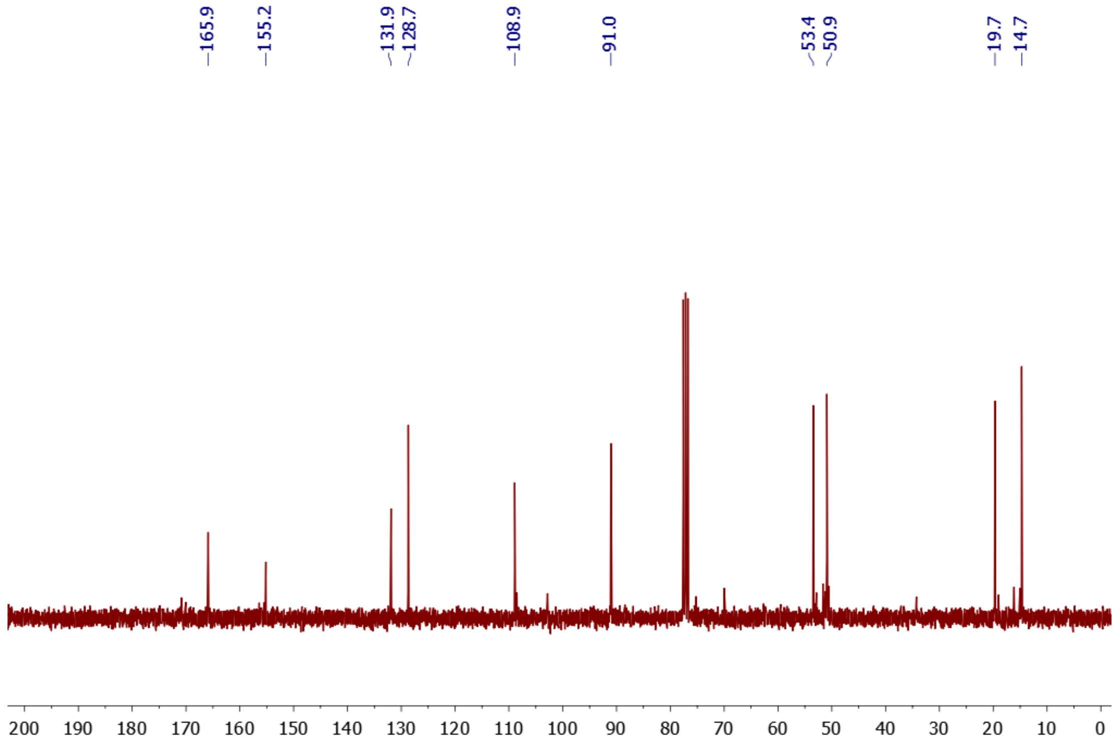

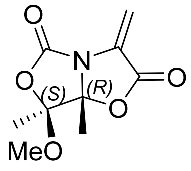

Dha **1**

^{1}H NMR in $CDCl_3$ (400 MHz) **1**

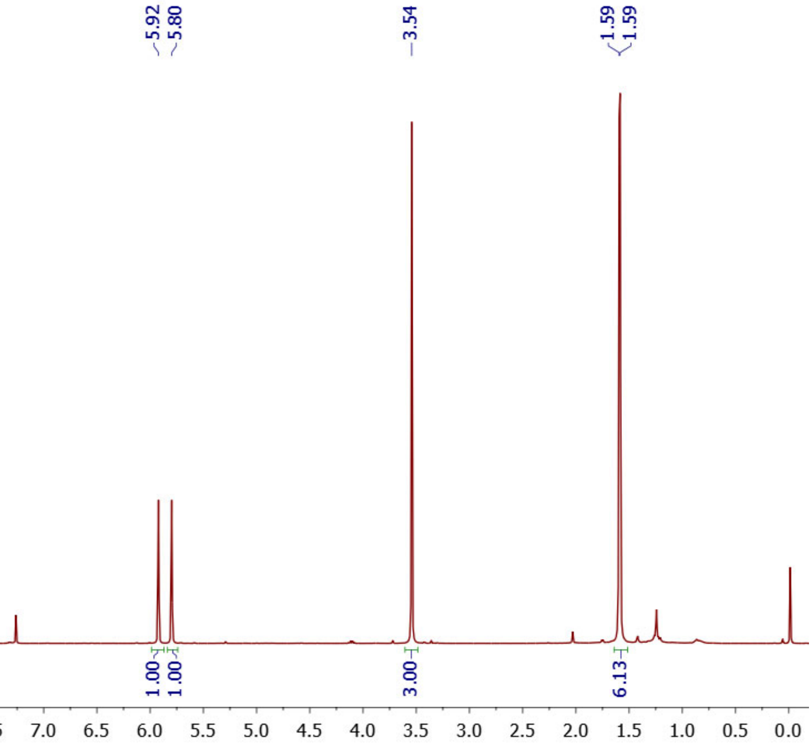

$^{13}C\{^{1}H\}$ NMR in $CDCl_3$ (100 MHz) **1**

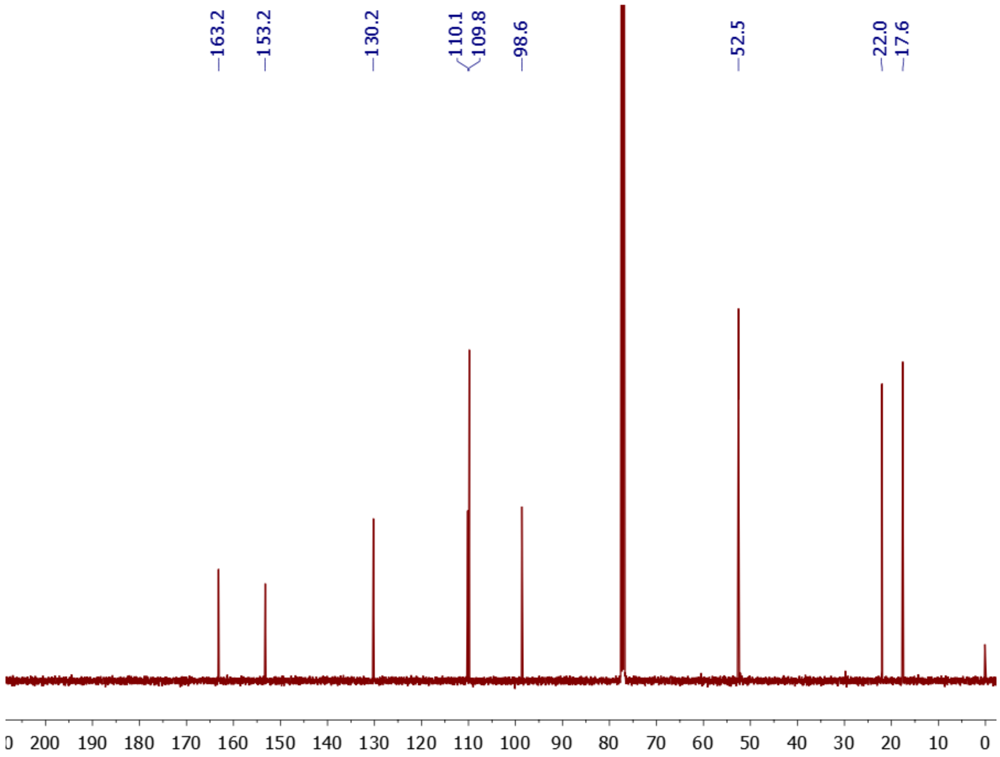

^{1}H NMR in $CDCl_3$ (400 MHz) **3a**

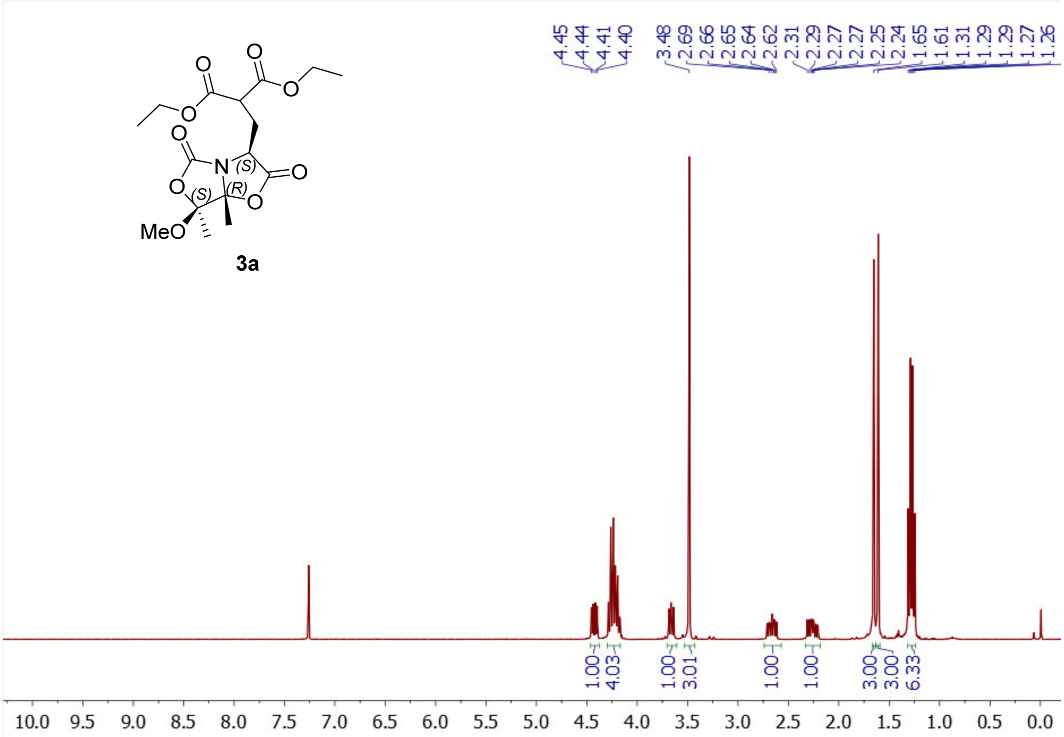

$^{13}C\{^{1}H\}$ NMR in $CDCl_3$ (100 MHz) **3a**

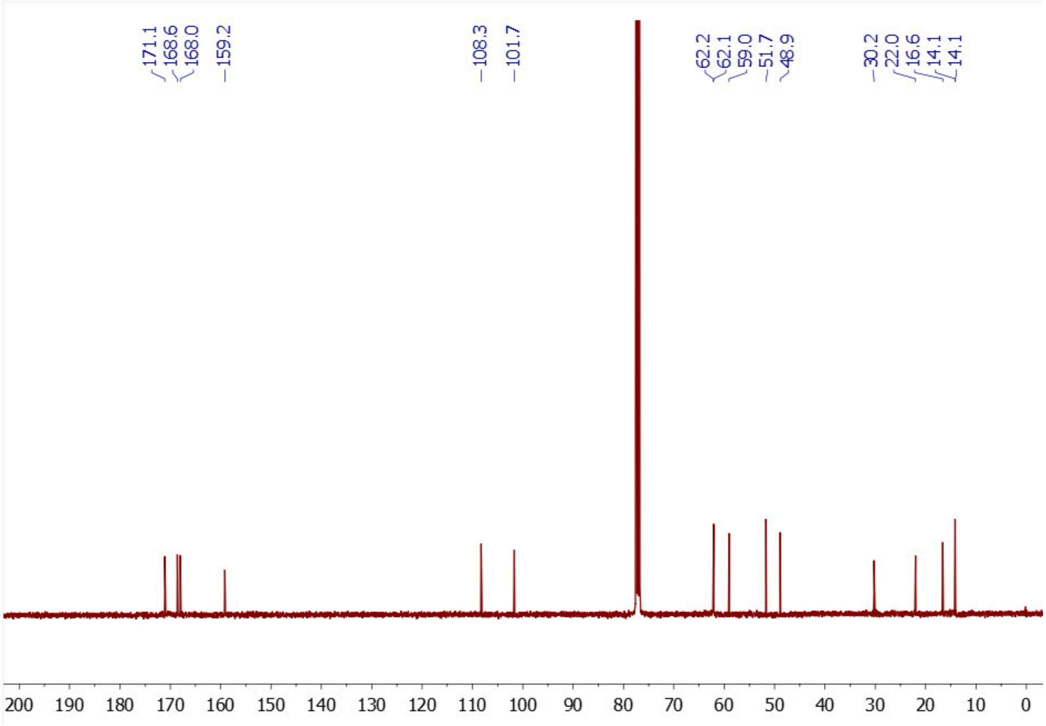

COSY in $CDCl_3$ **3a**

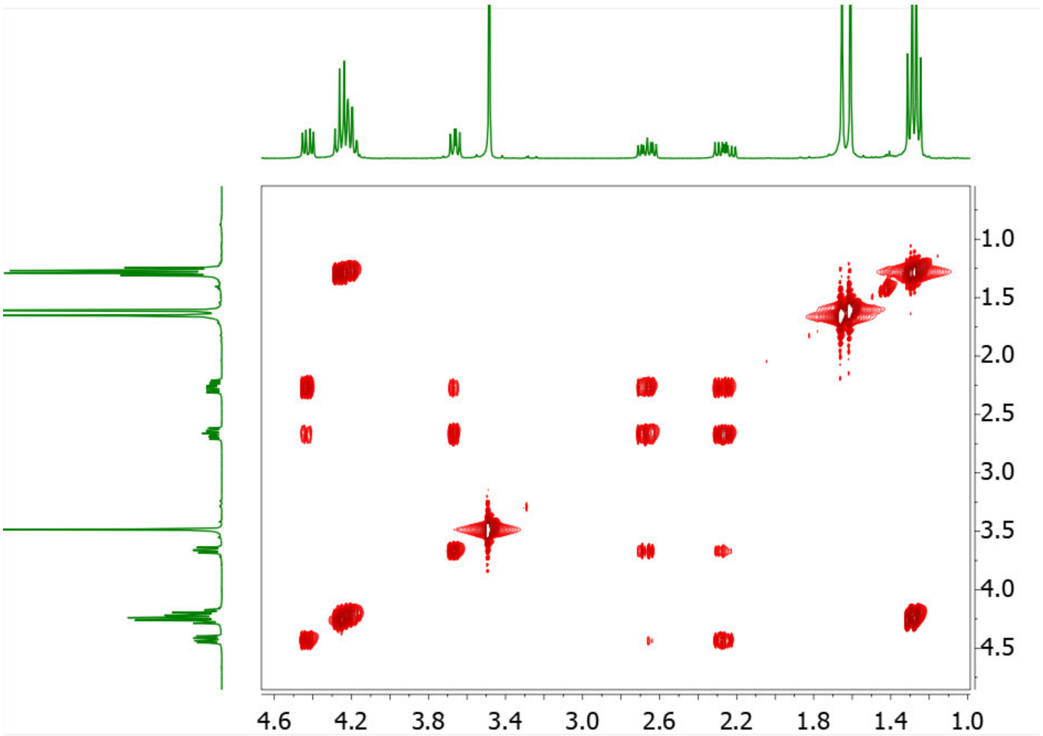

HSQC in $CDCl_3$ **3a**

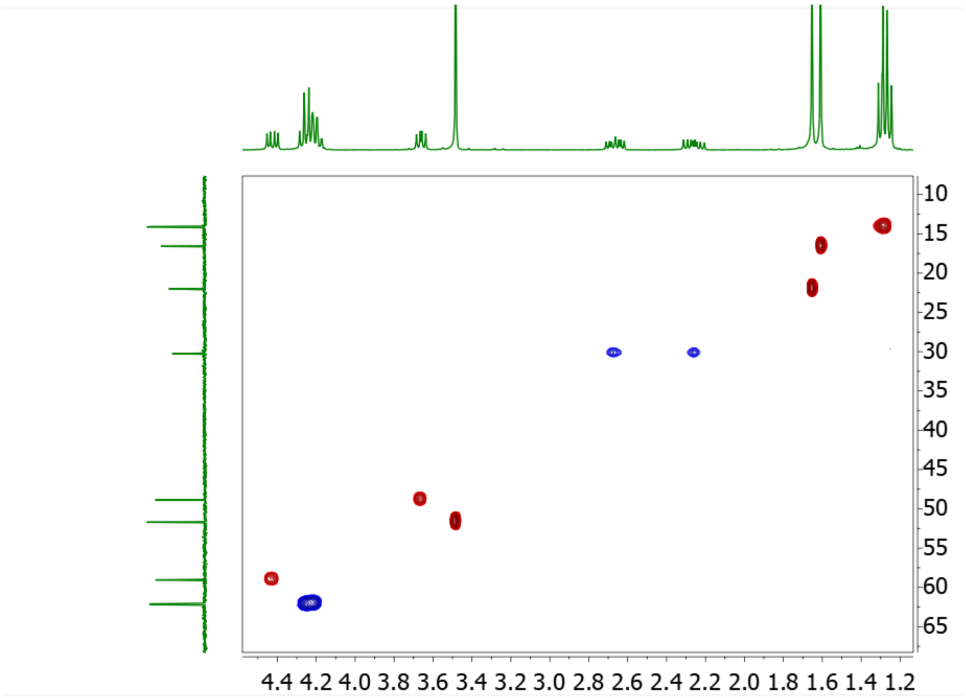

^{1}H NMR in $CDCl_3$ (400 MHz) **3b**

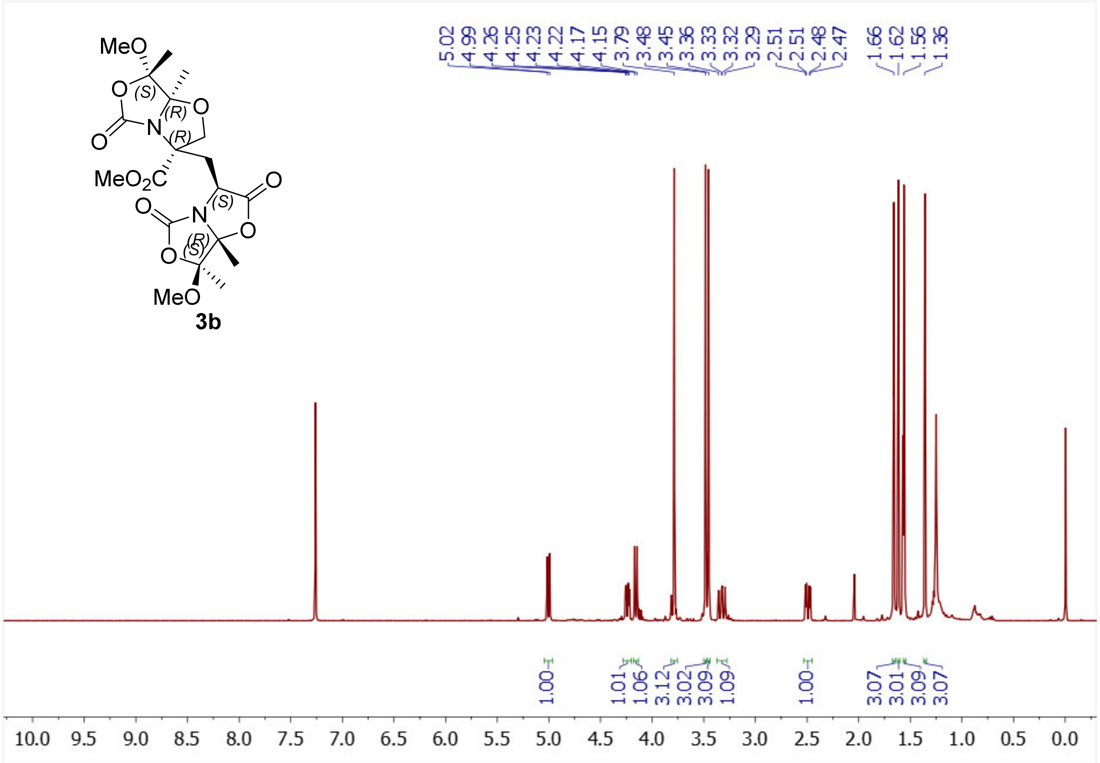

$^{13}C\{^{1}H\}$ NMR in $CDCl_3$ (100 MHz) **3b**

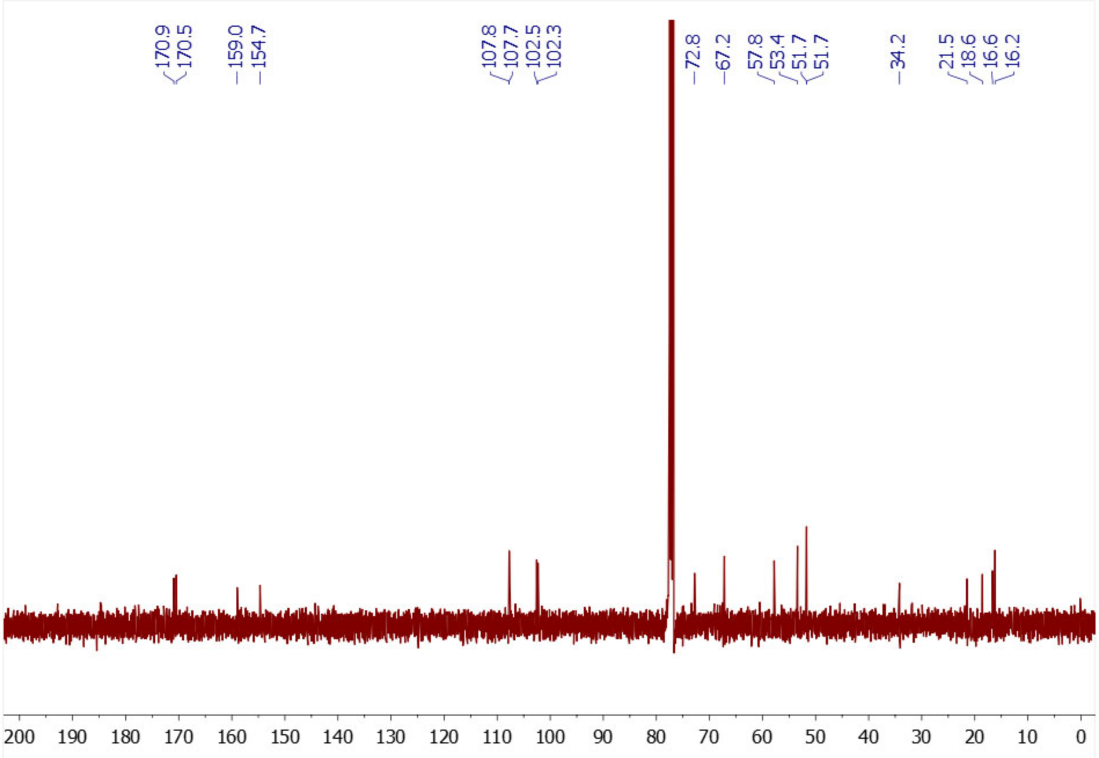

COSY in $CDCl_3$ **3b**

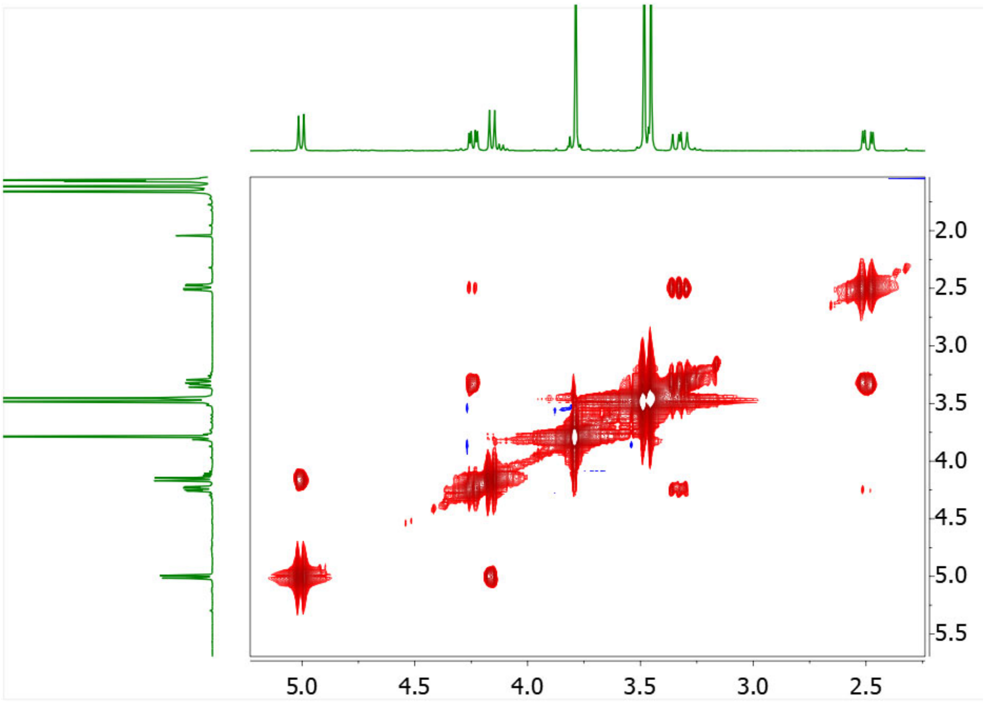

HSQC in $CDCl_3$ **3b**

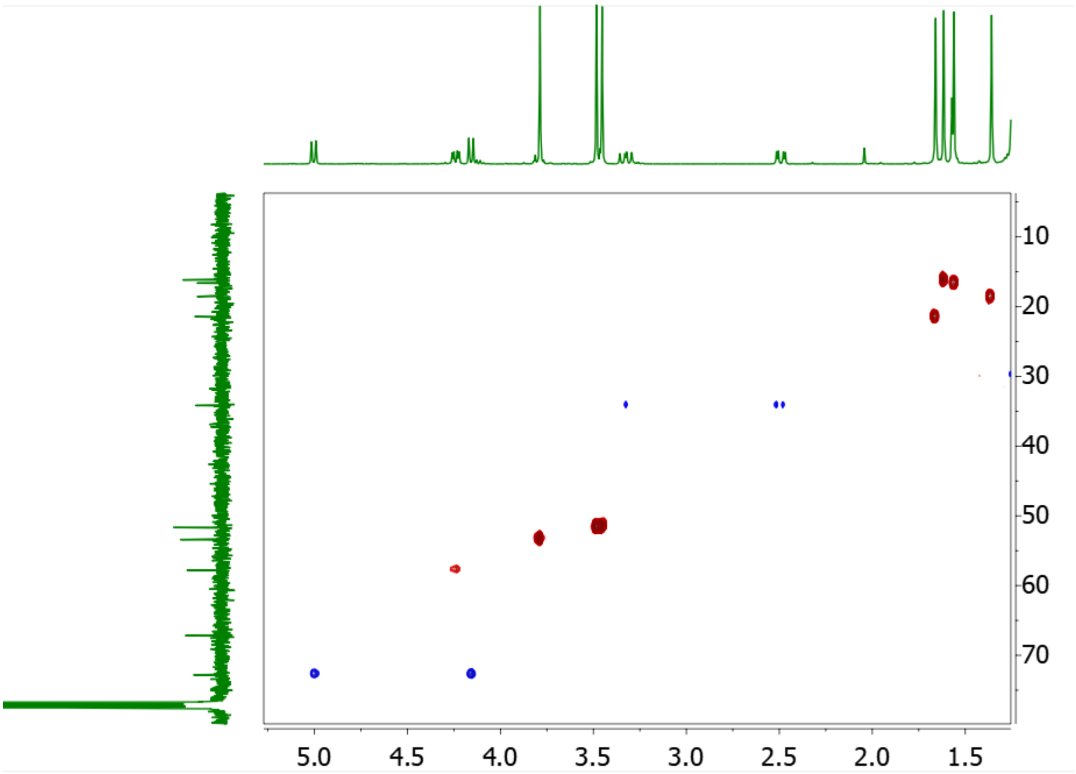

^{1}H NMR in $CDCl_3$ (400 MHz) **3a-D**

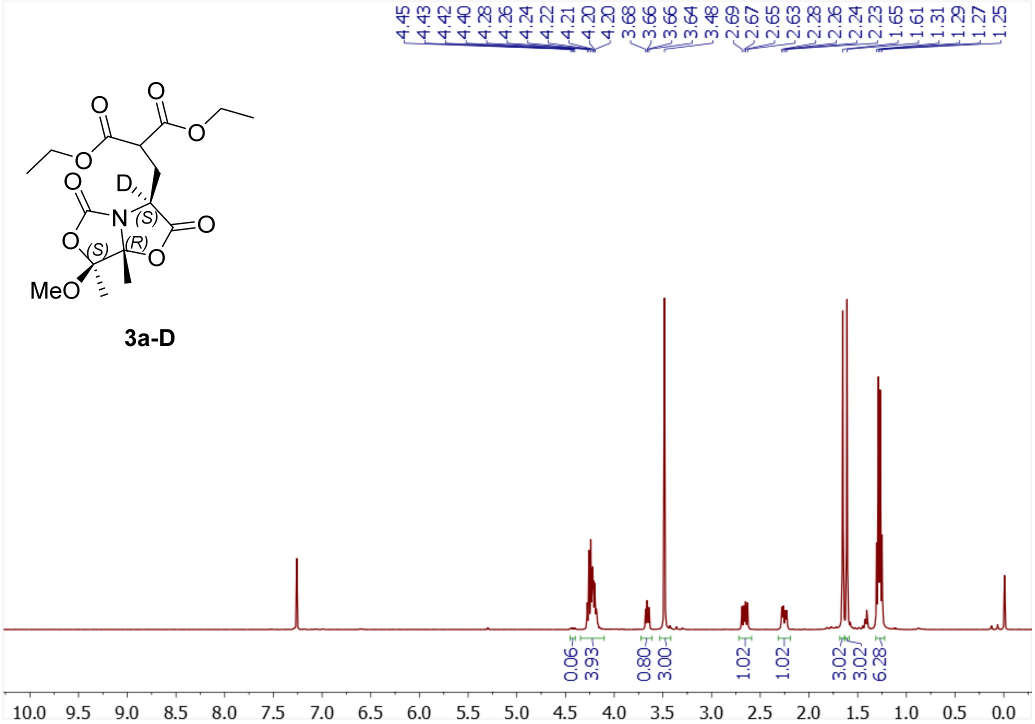

3a-D

$^{13}C\{^{1}H\}$ NMR in $CDCl_3$ (100 MHz) **3a-D**

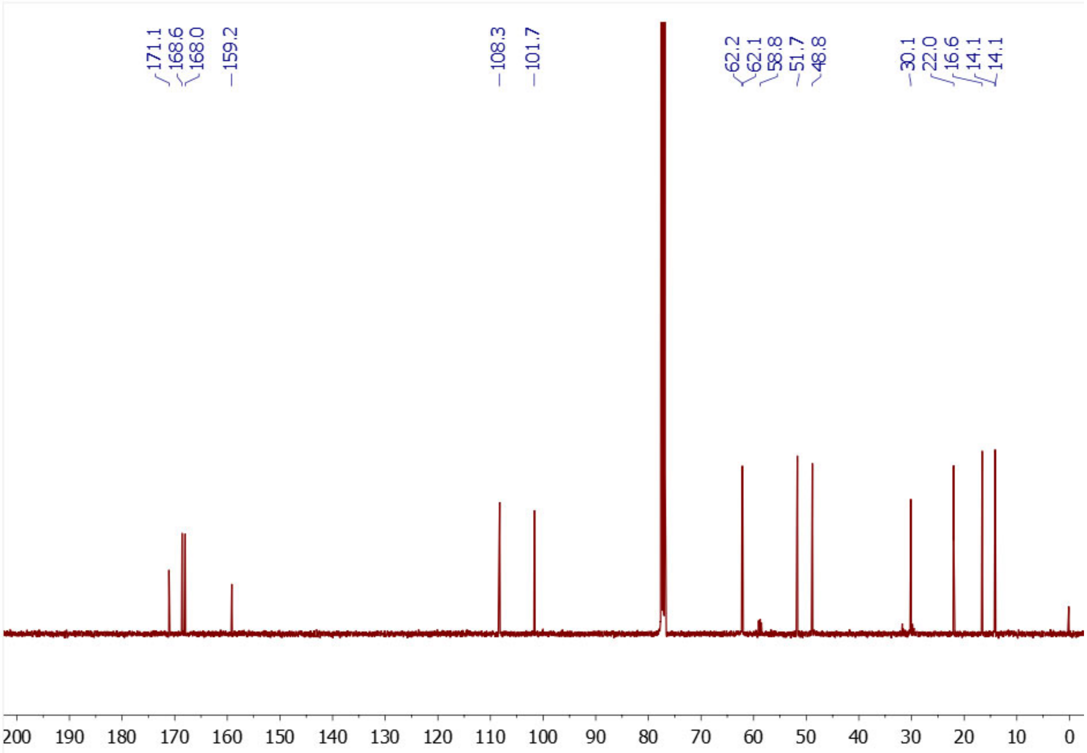

COSY in $CDCl_3$ **3a-D**

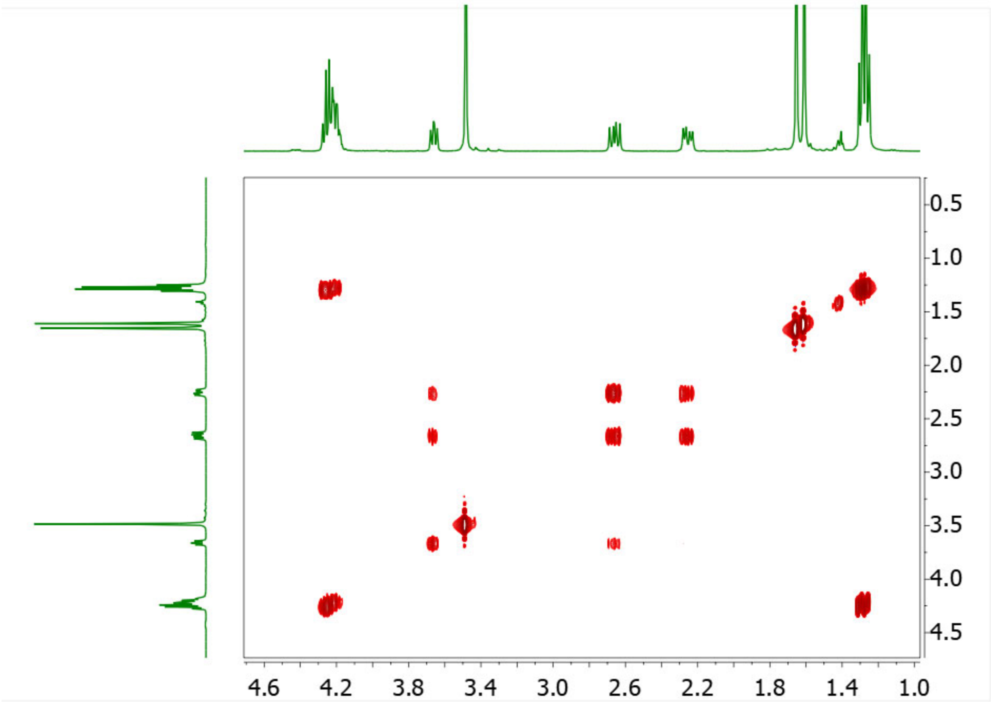

HSQC in $CDCl_3$ **3a-D**

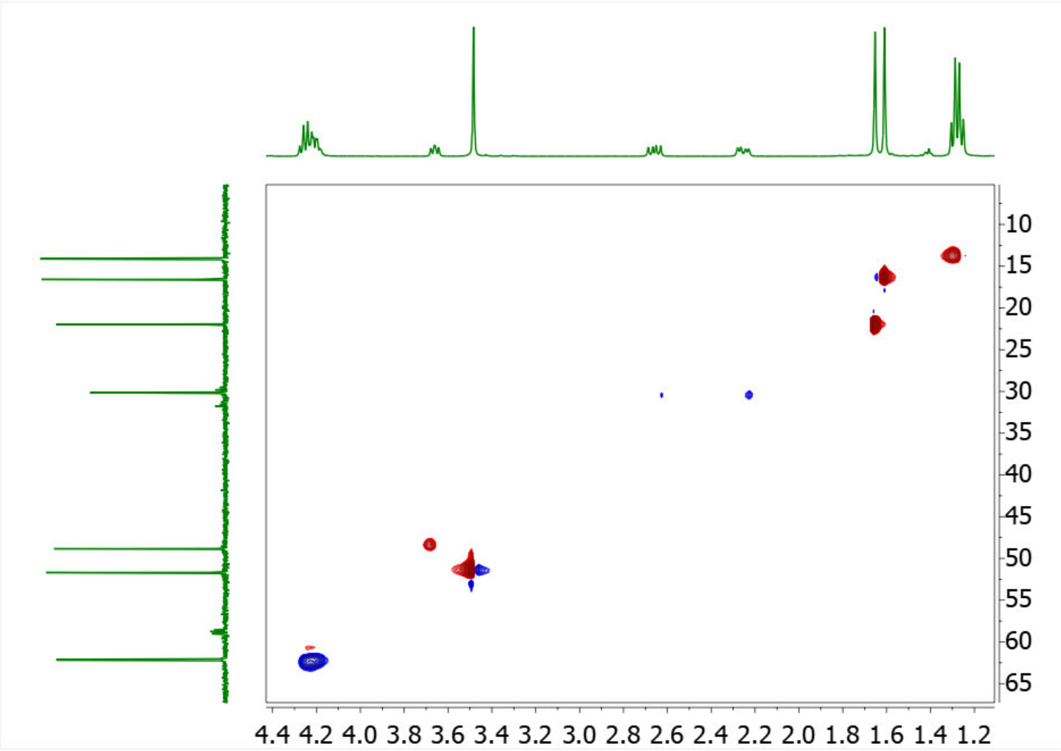

^{1}H NMR in D_2O (400 MHz) **4a**

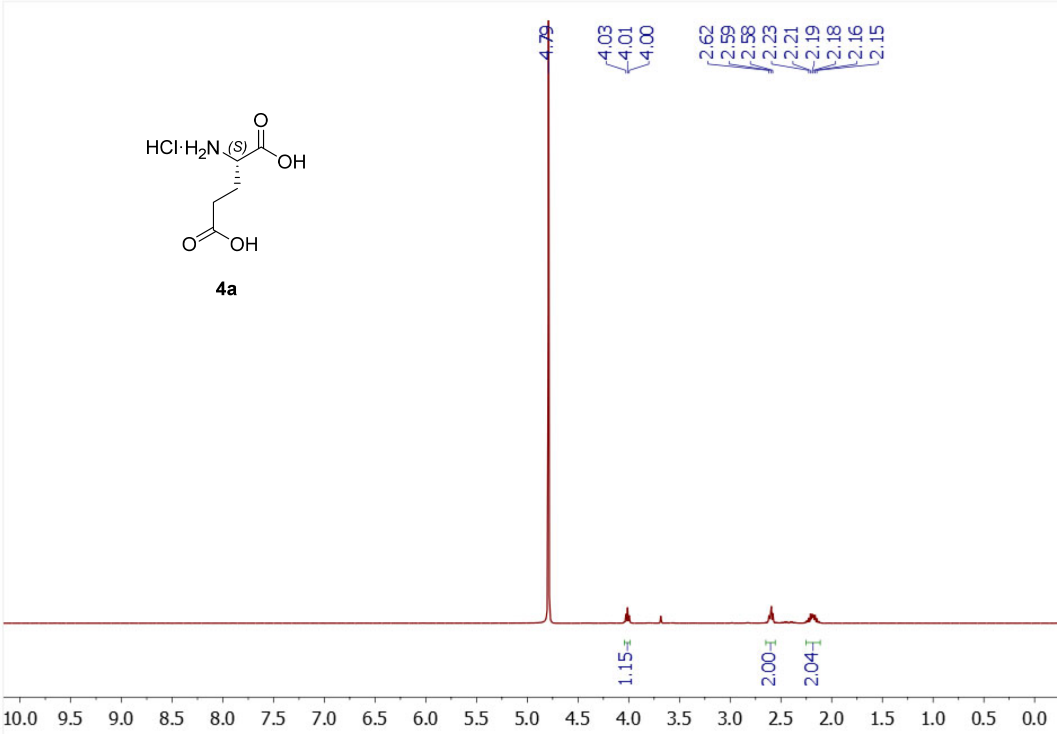

^{13}C{^{1}H} NMR in D_2O (100 MHz) **4a**

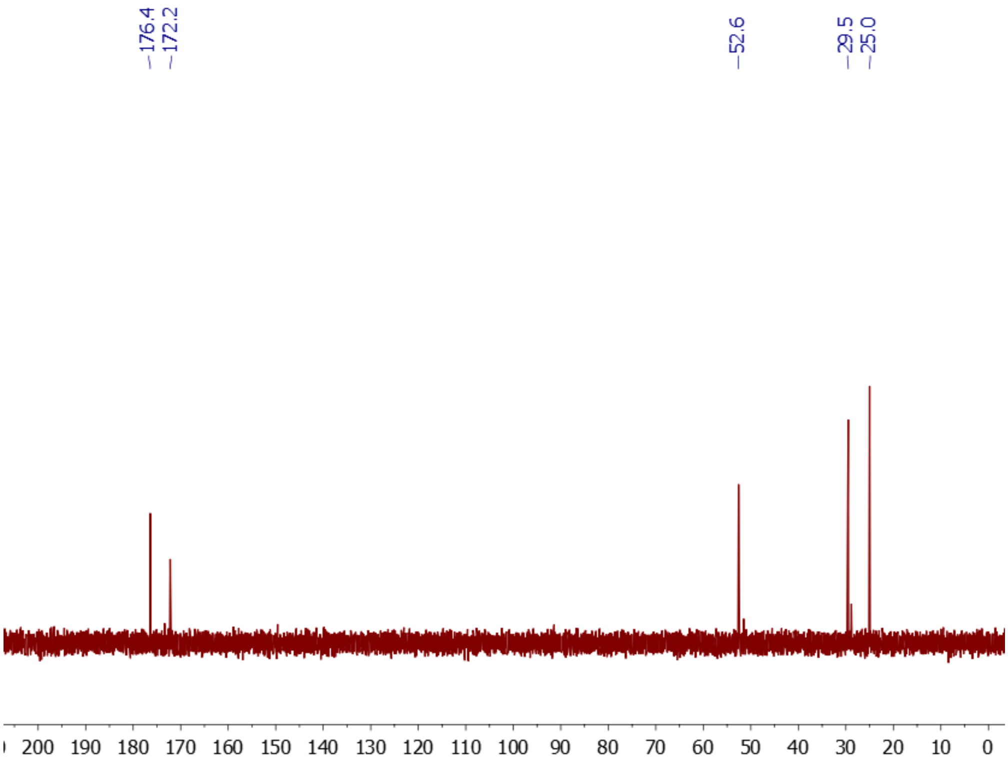

COSY in D_2O **4a**

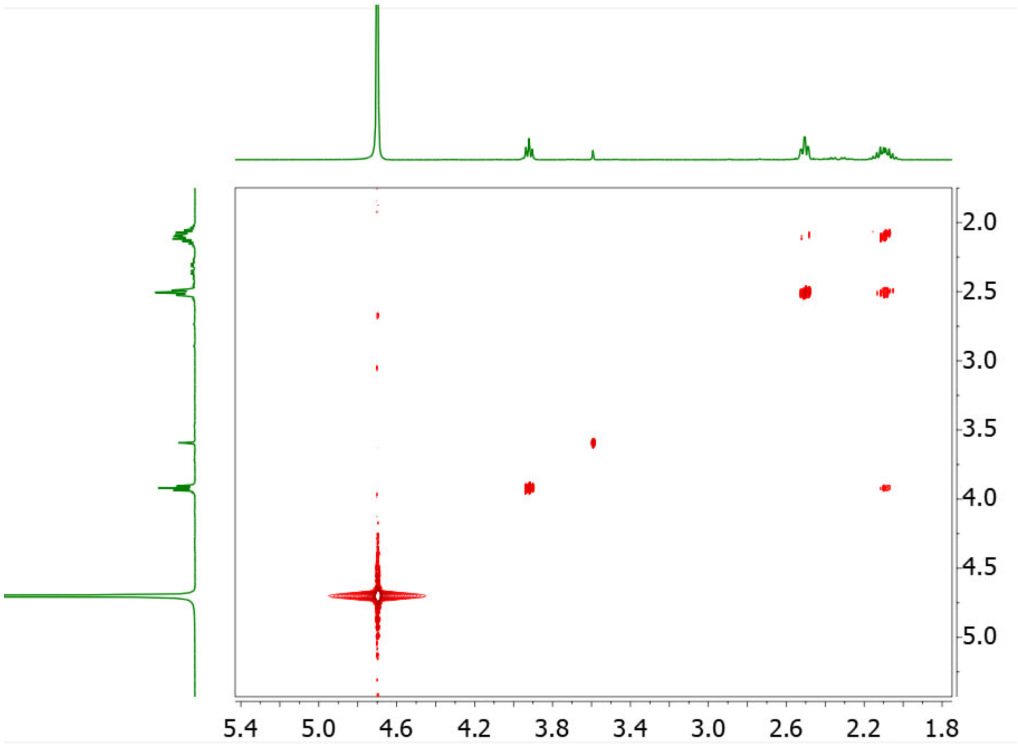

HSQC in D_2O **4a**

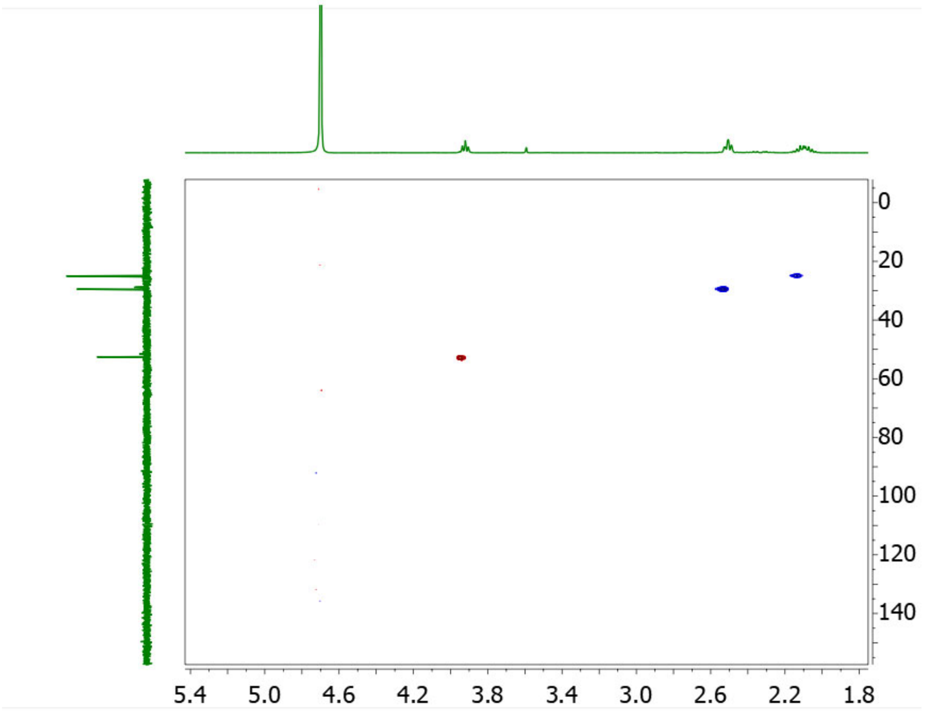

^{1}H NMR in D_2O (400 MHz) **4a-D**

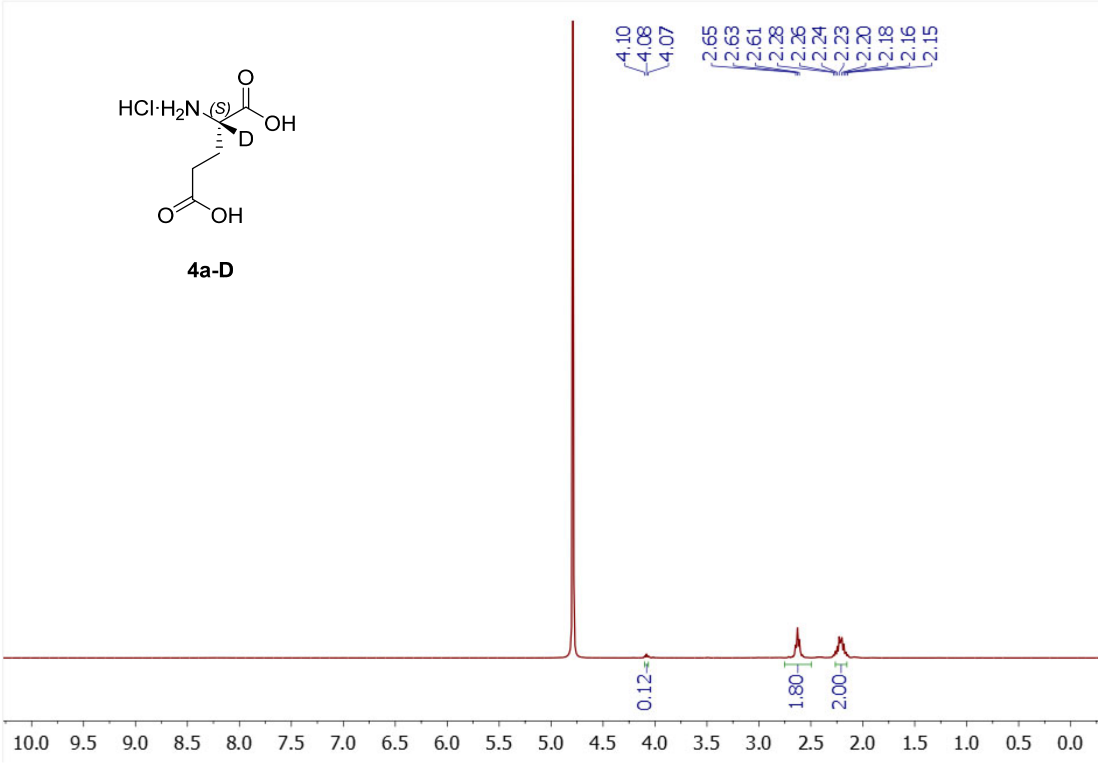

$^{13}C\{^{1}H\}$ NMR in D_2O (100 MHz) **4a-D**

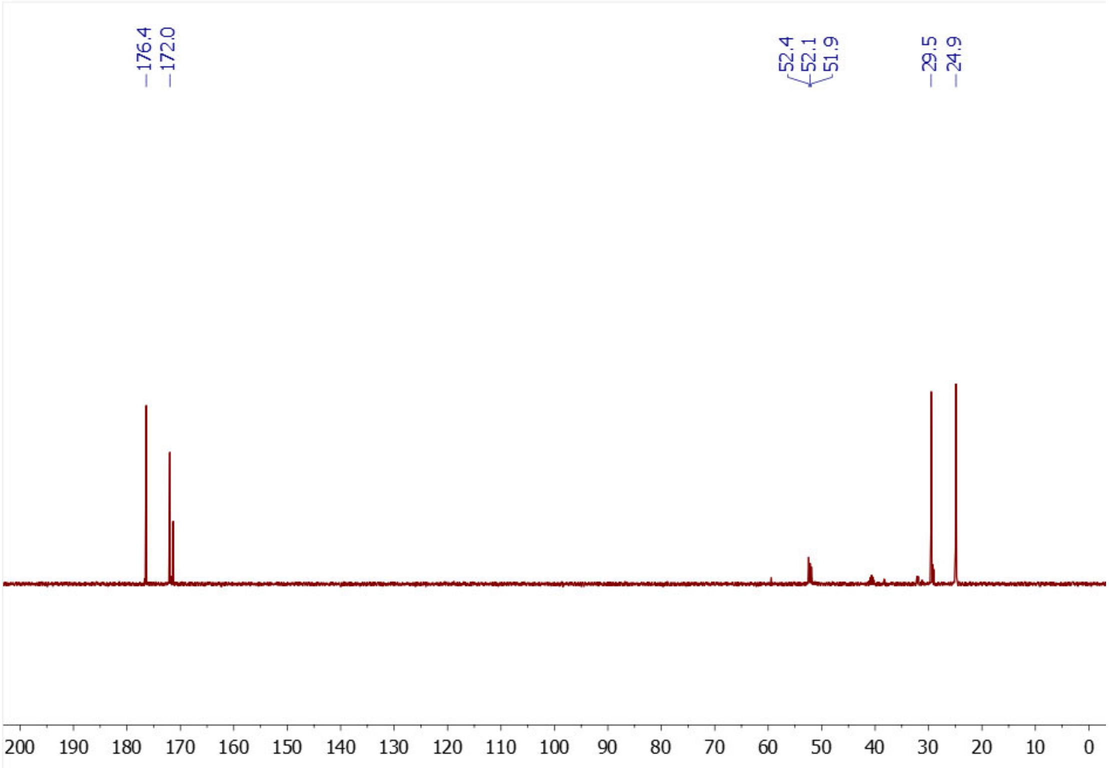

COSY in D_2O **4a-D**

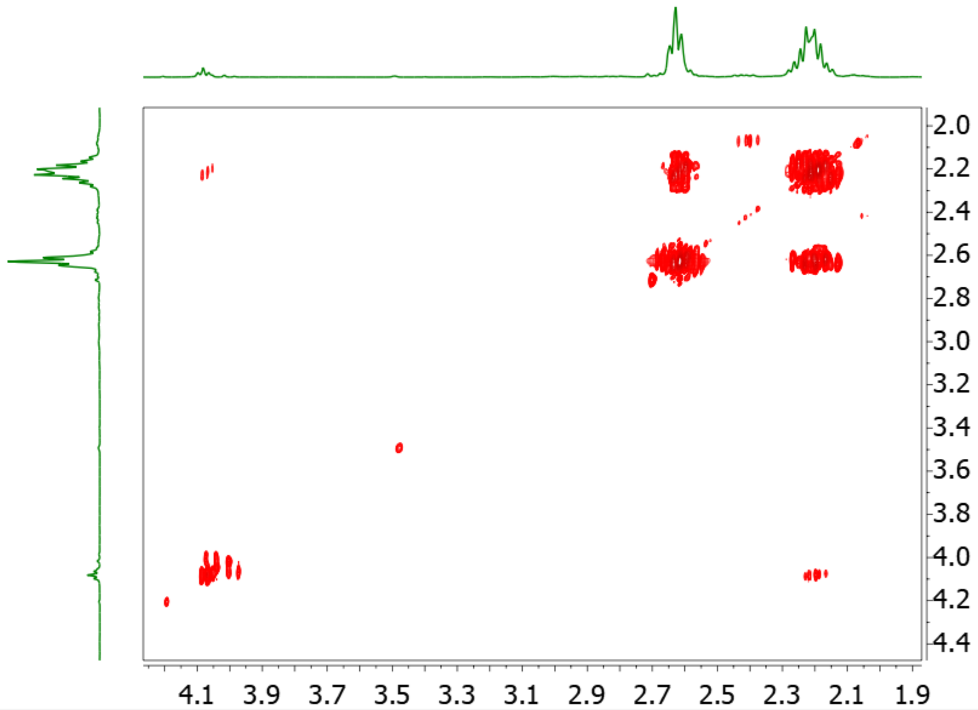

HSQC in D_2O **4a-D**

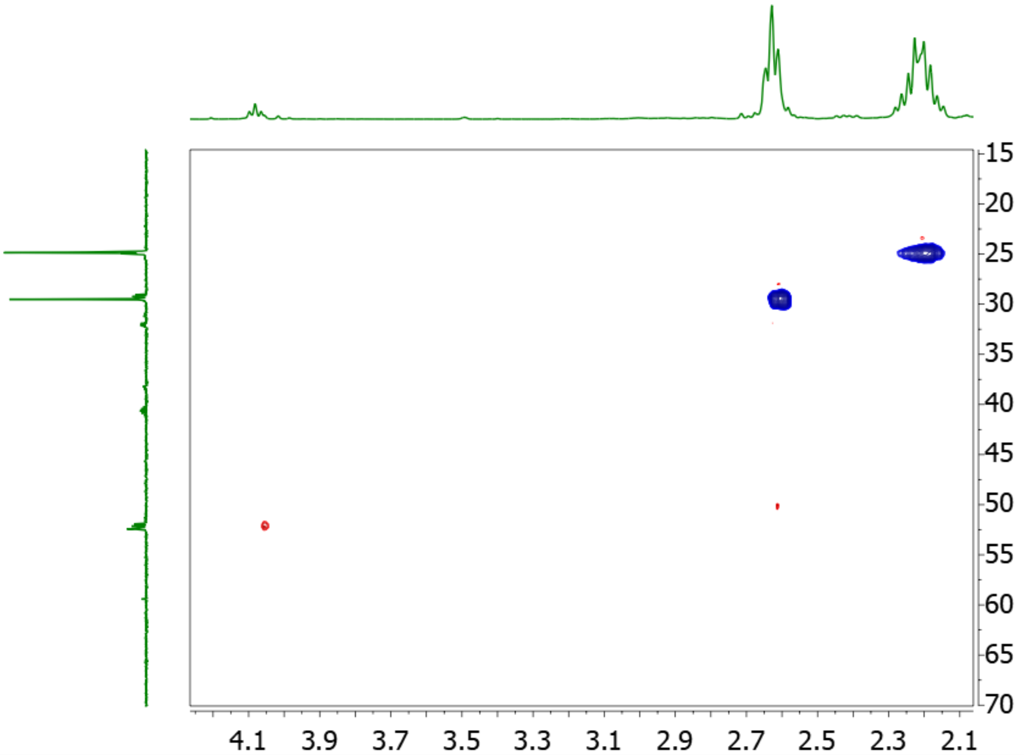

^{1}H NMR in D_2O (400 MHz) **4b**

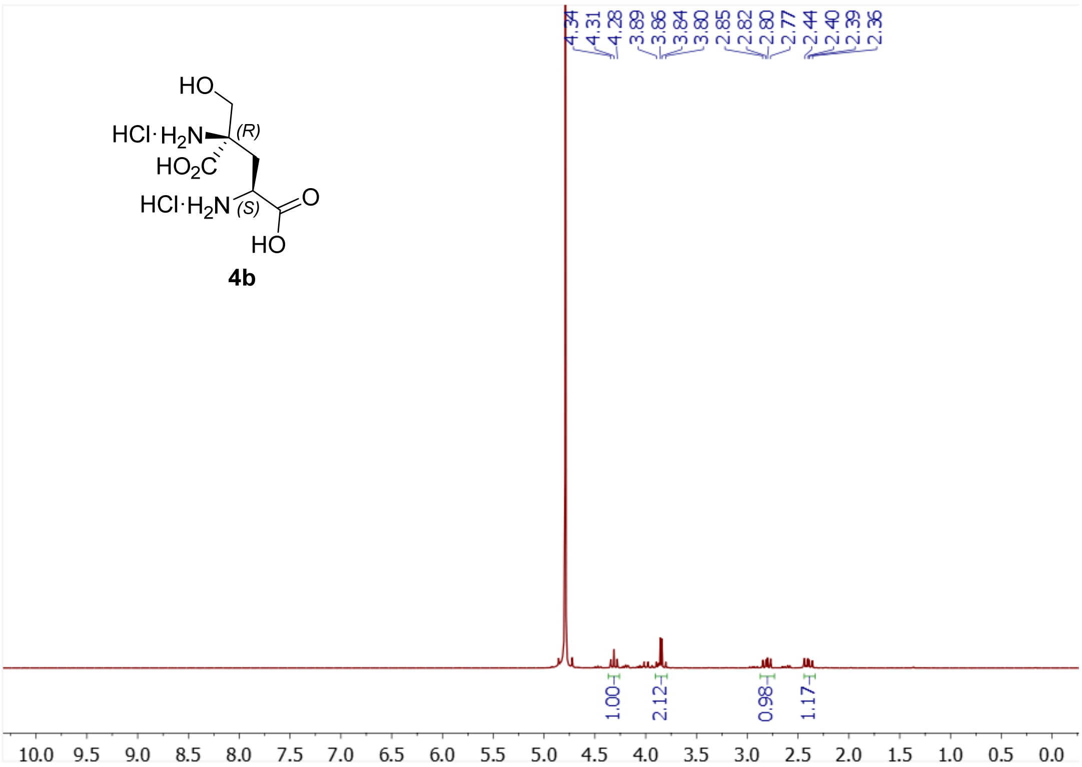

$^{13}C\{^{1}H\}$ NMR in D_2O (100 MHz) **4b**

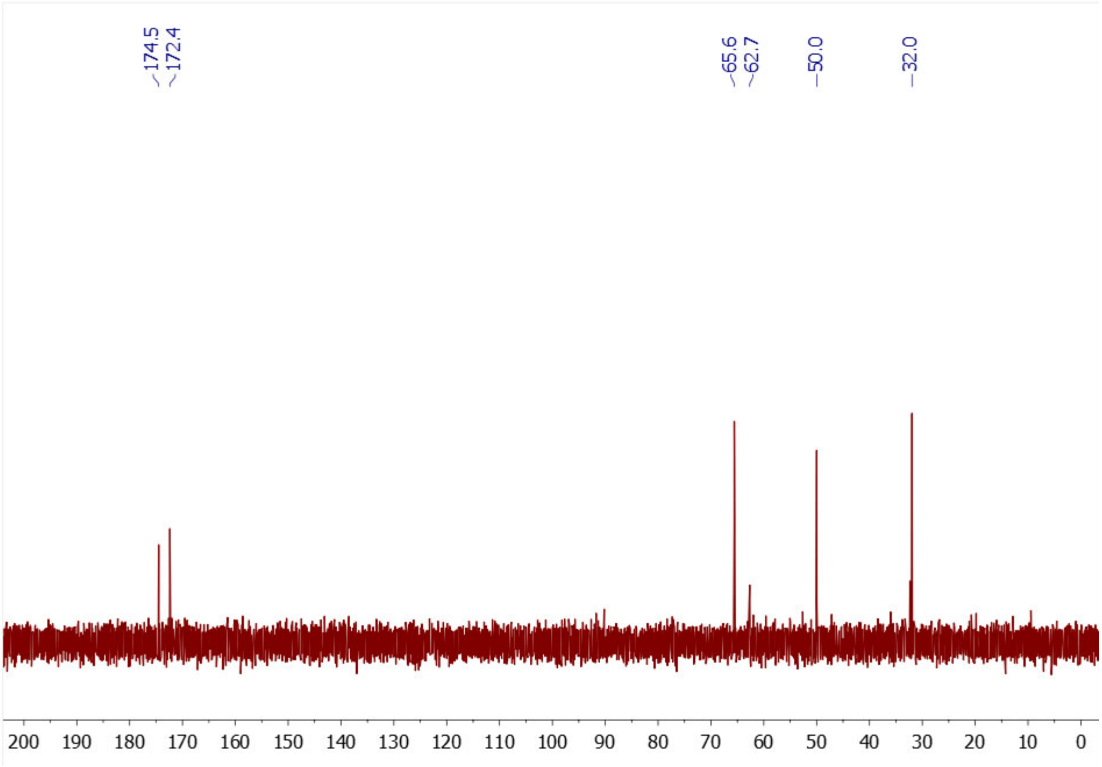

COSY in D_2O **4b**

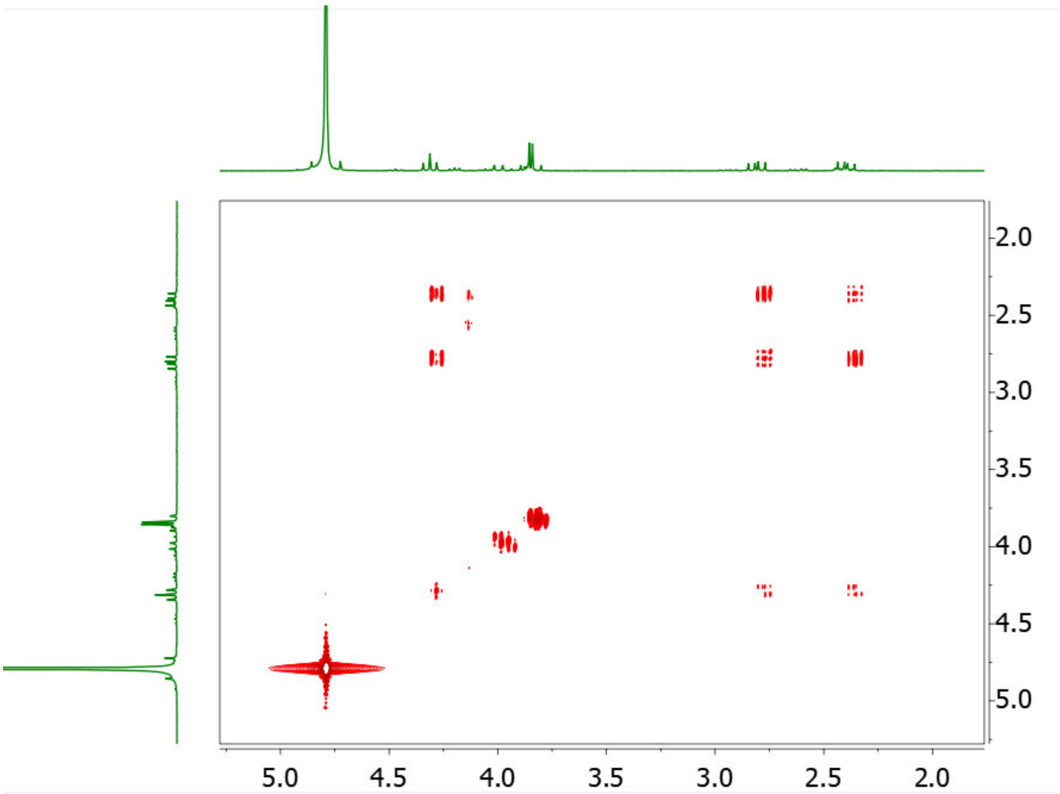

HSQC in D_2O **4b**

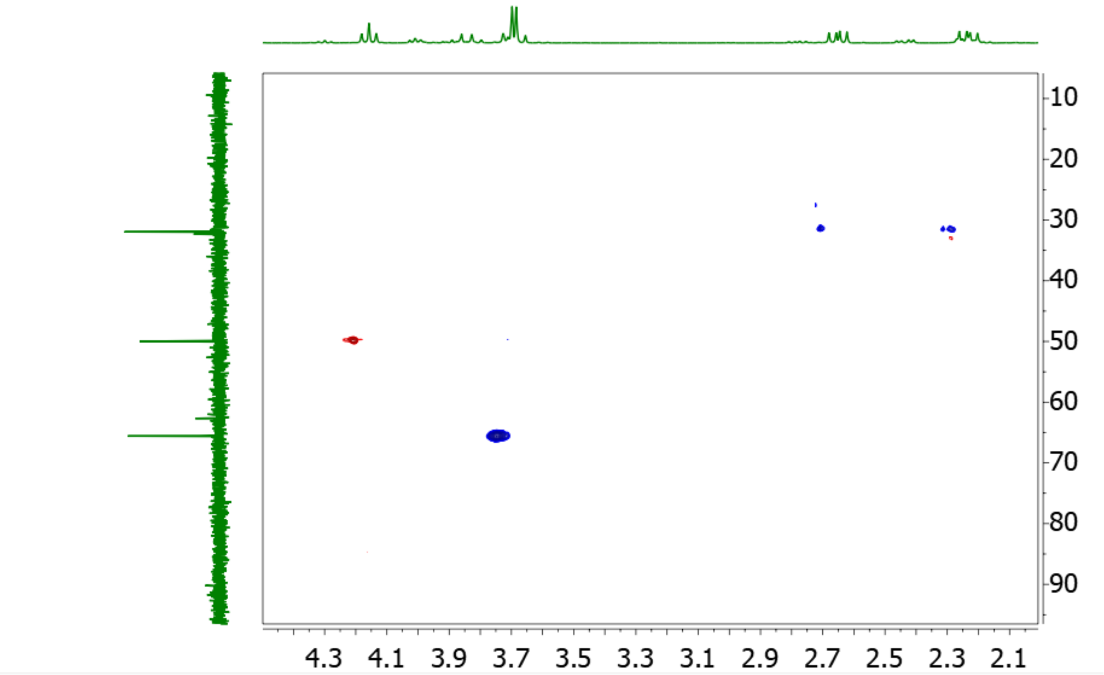

^{1}H NMR in $CDCl_3$ (400 MHz) **3c**

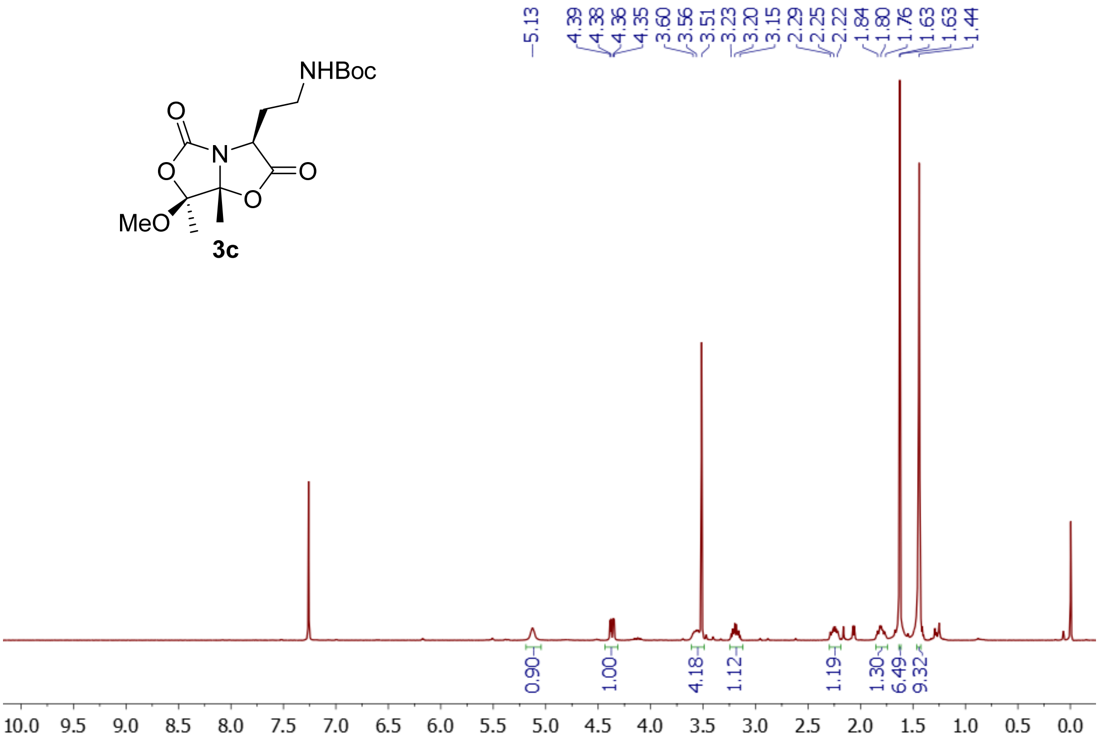

3c

$^{13}C\{^{1}H\}$ NMR in $CDCl_3$ (100 MHz) **3c**

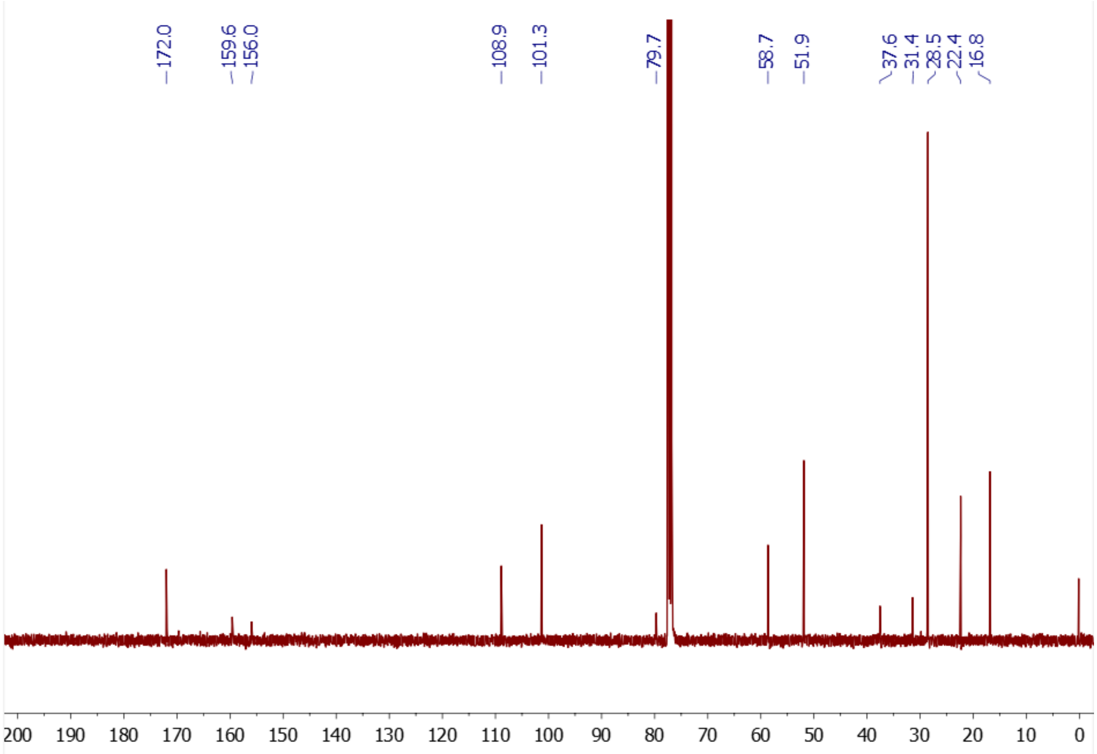

COSY in $CDCl_3$ **3c**

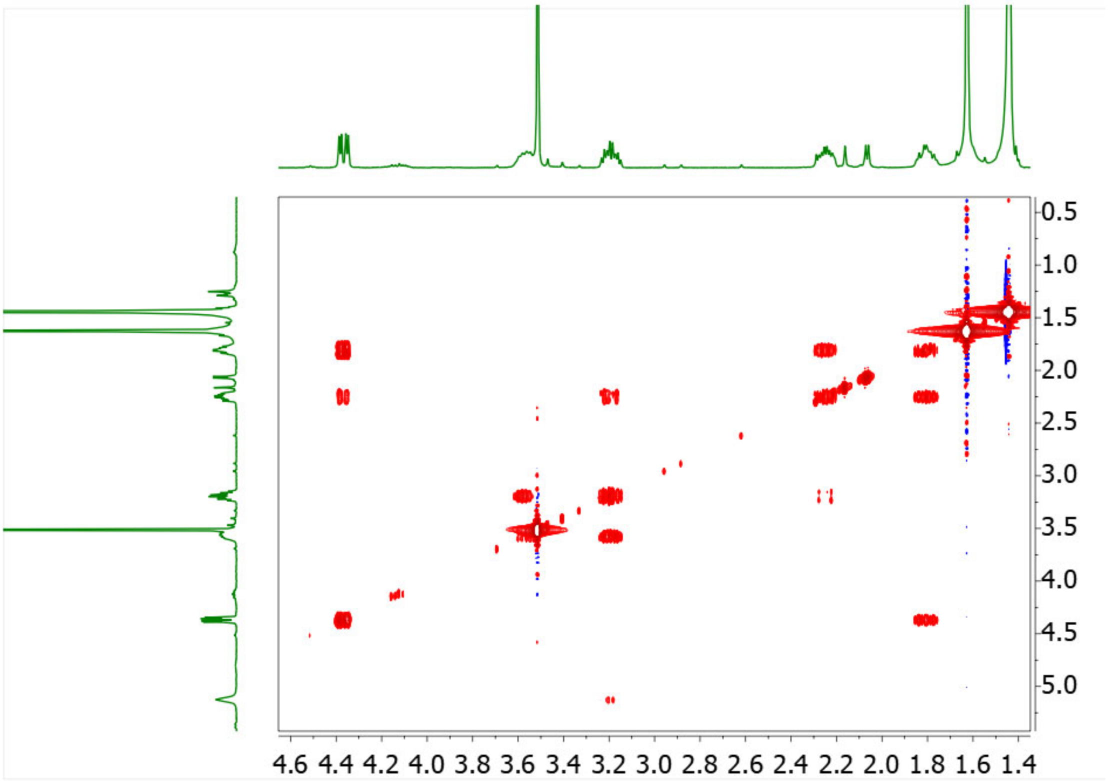

HSQC in $CDCl_3$ **3c**

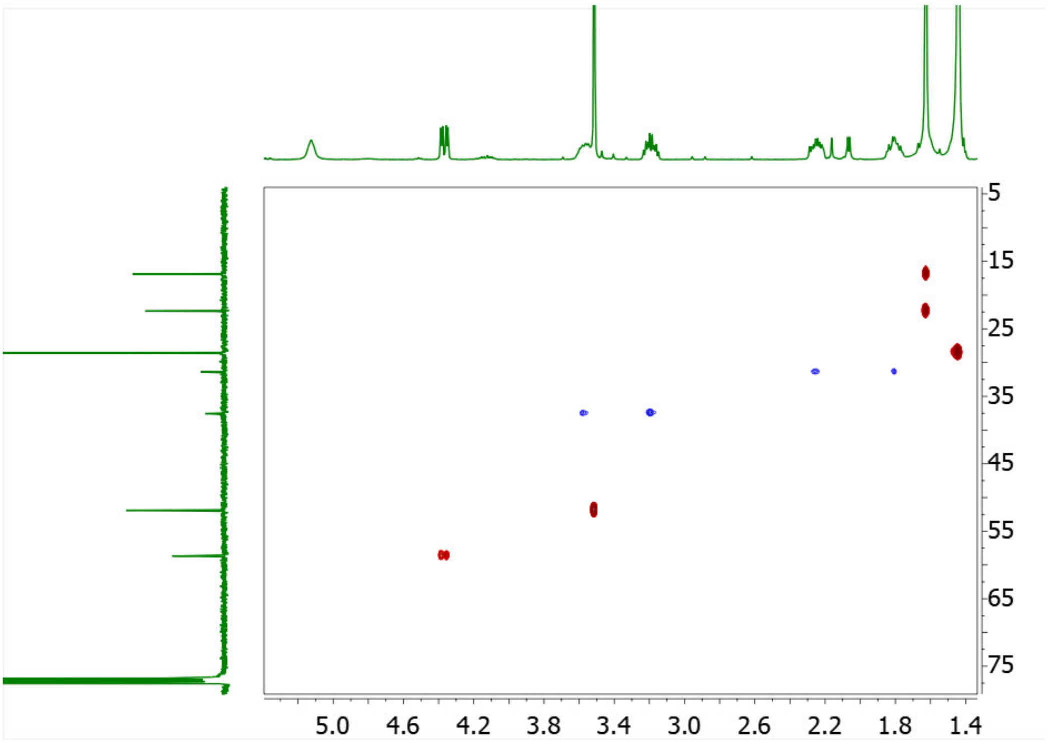

^{1}H NMR in $CDCl_3$ (400 MHz) **3c-D**

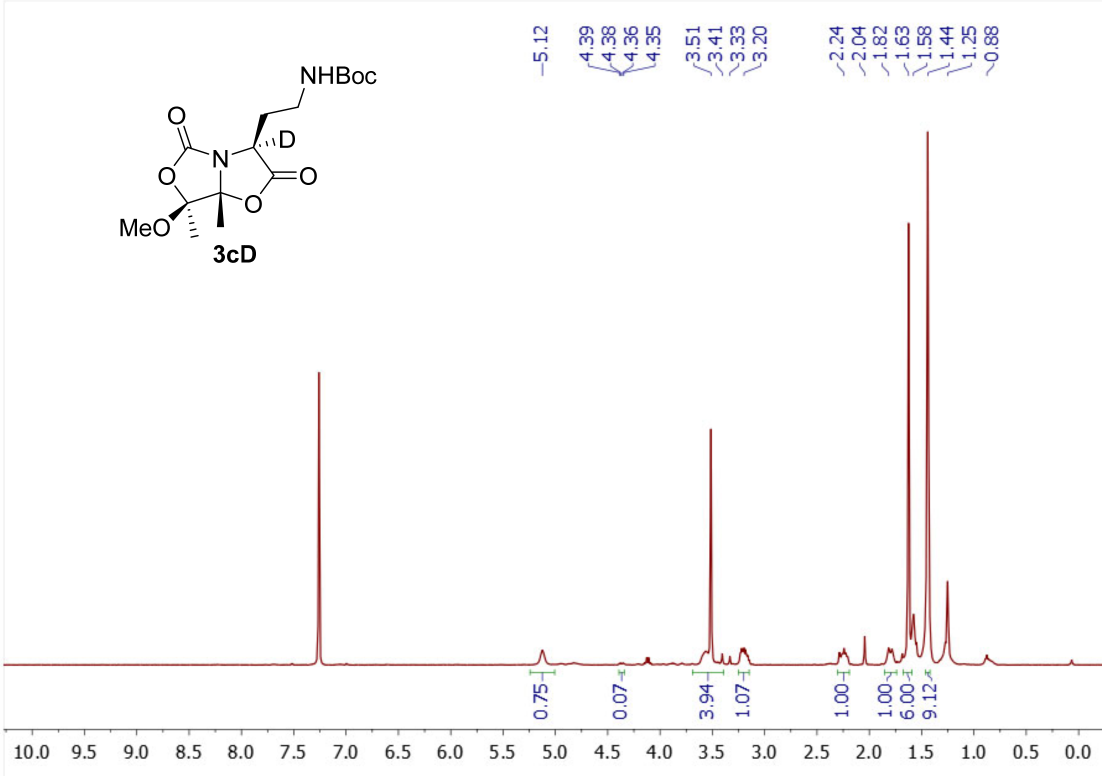

^{13}C{^{1}H} NMR in $CDCl_3$ (100 MHz) **3c-D**

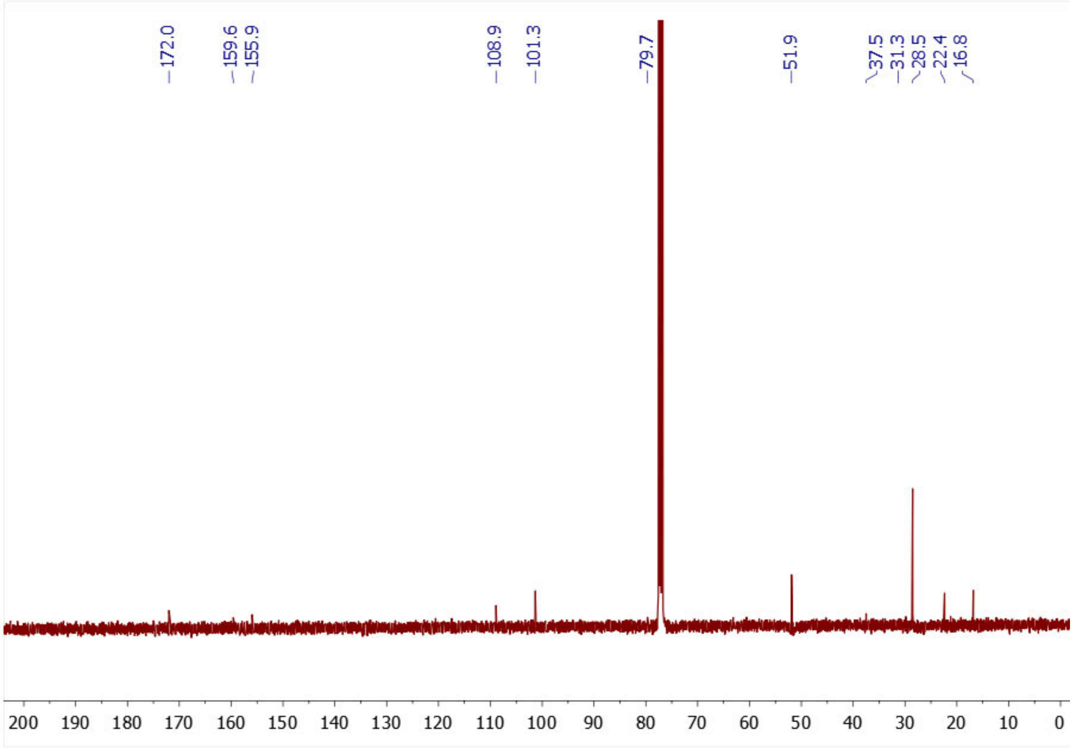

COSY in $CDCl_3$ **3c-D**

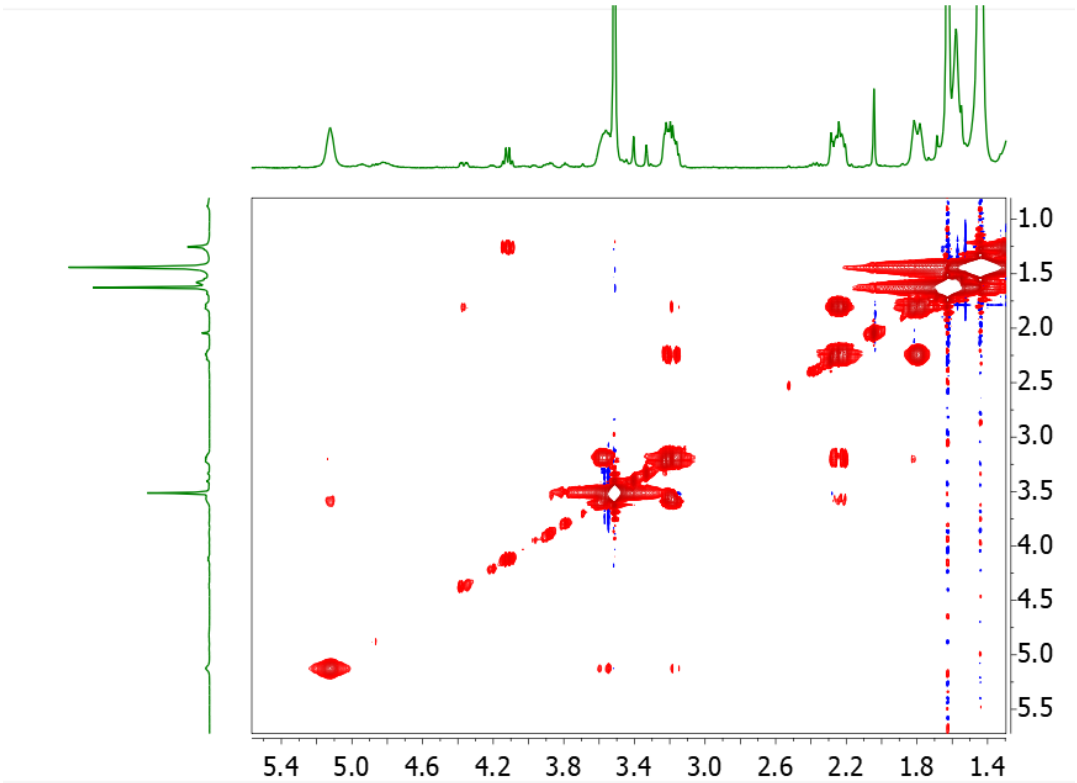

HSQC in $CDCl_3$ **3c-D**

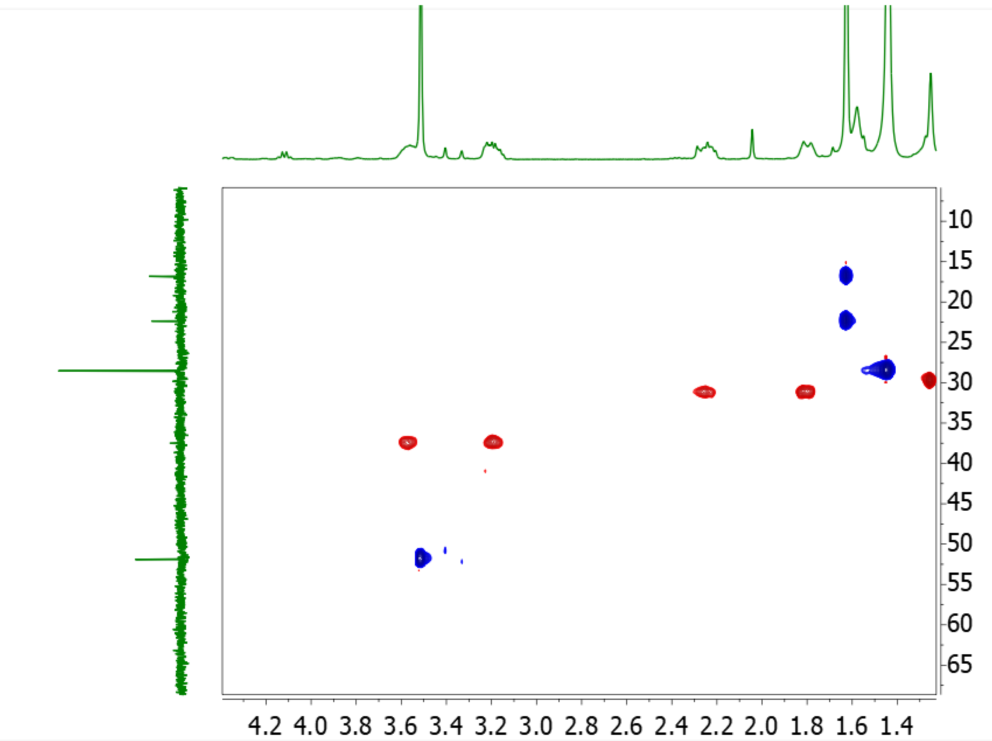

^{1}H NMR in $CDCl_3$ (400 MHz) **3d**

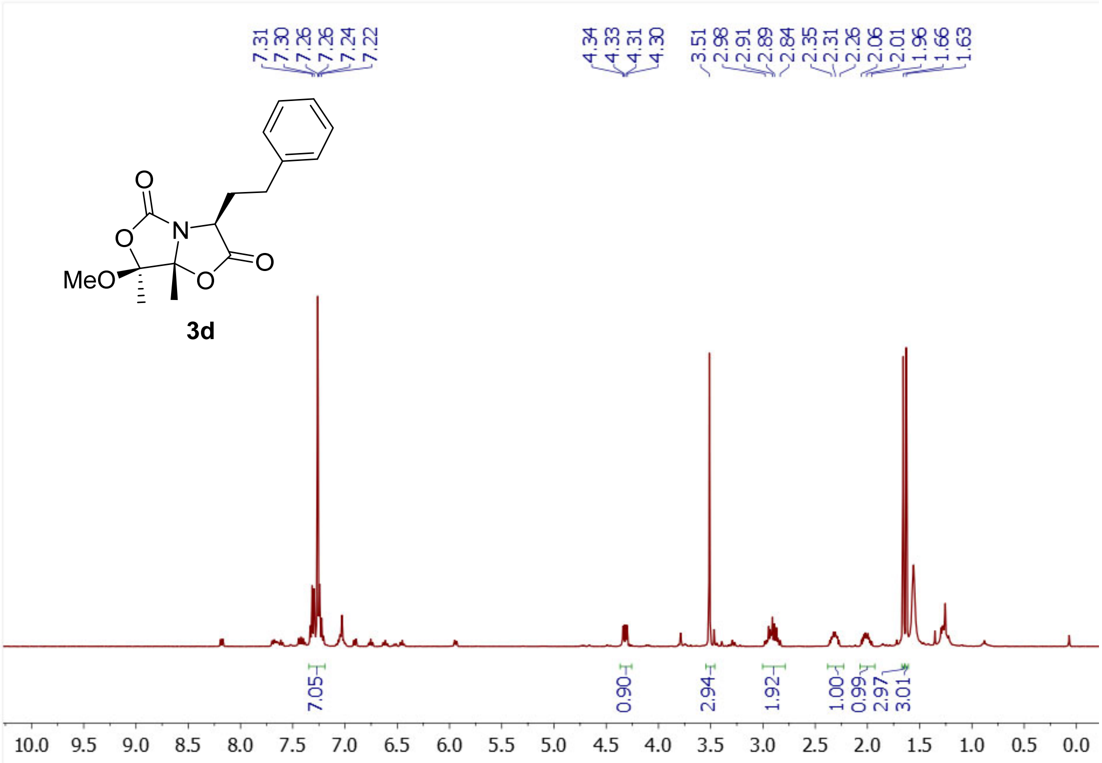

$^{13}C\{^{1}H\}$ NMR in $CDCl_3$ (100 MHz) **3d**

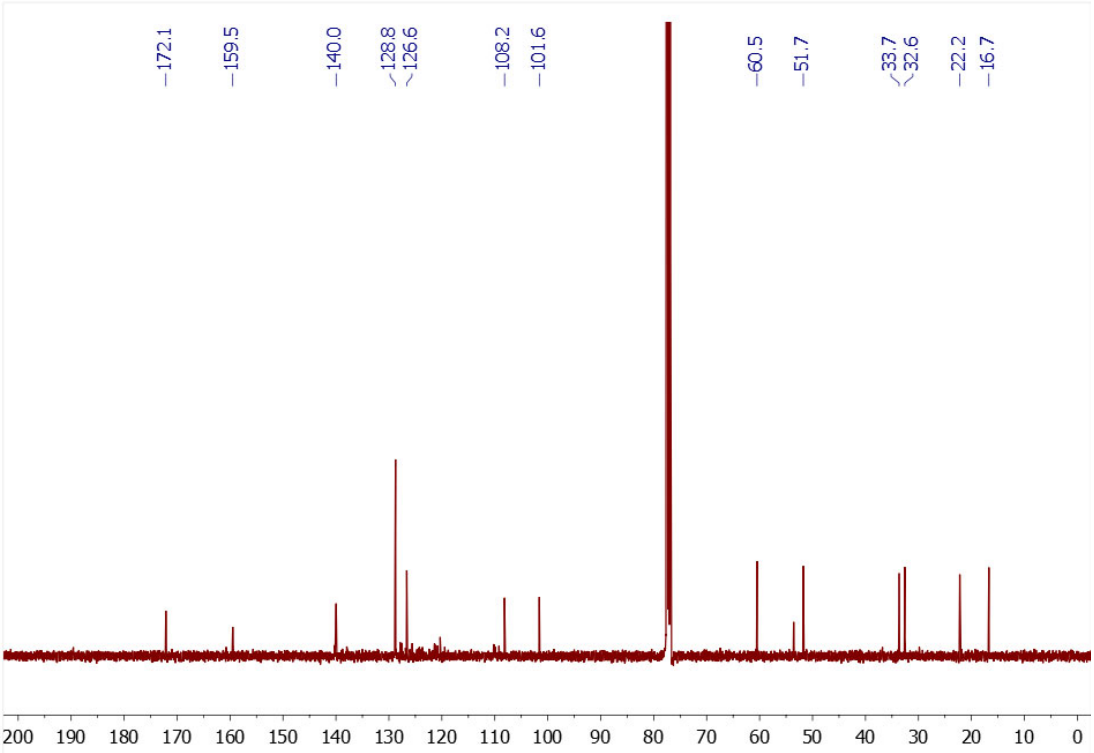

COSY in $CDCl_3$ **3d**

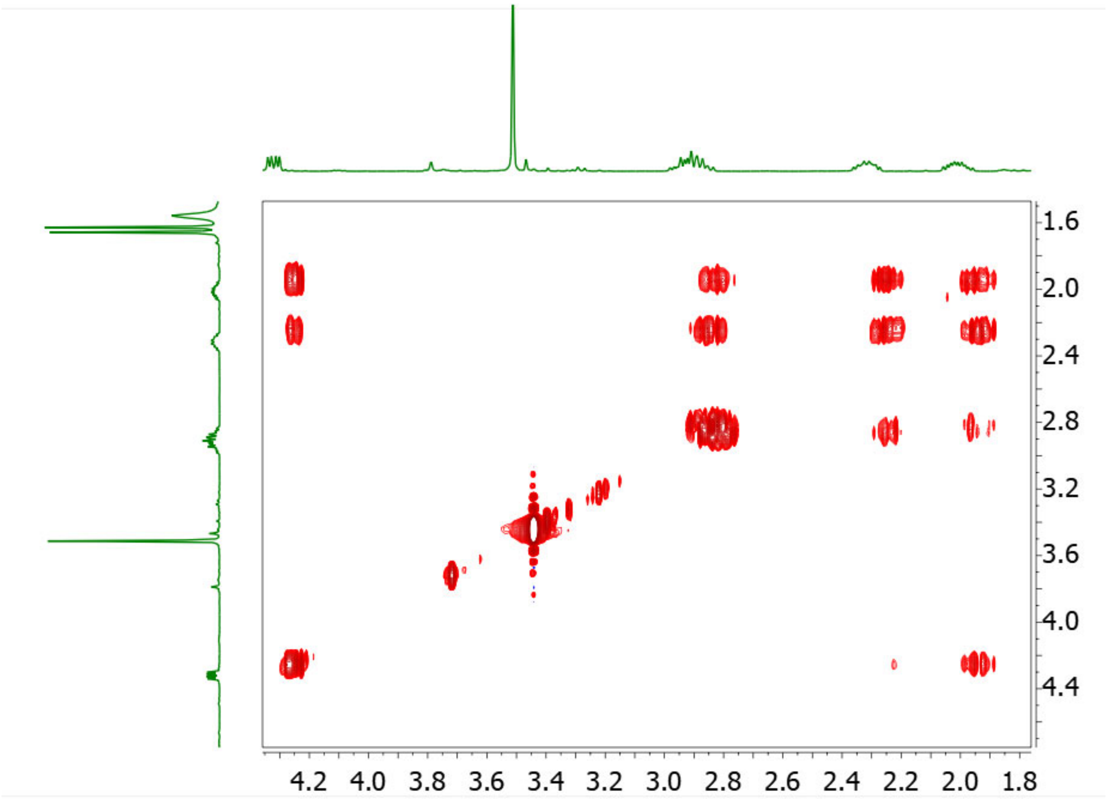

HSQC in $CDCl_3$ **3d**

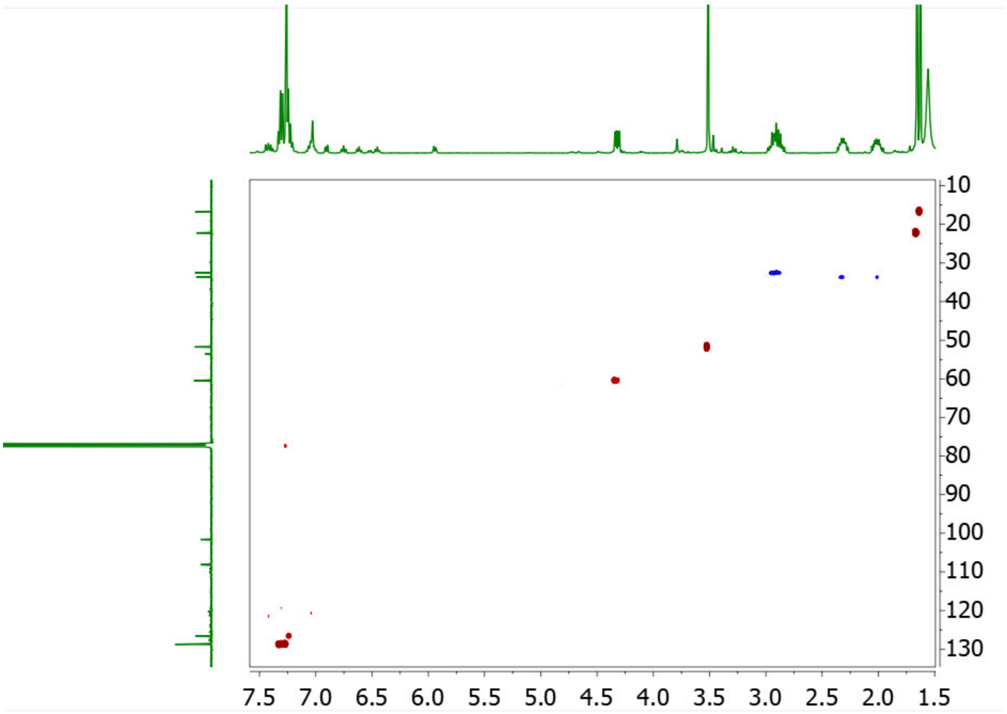

^{1}H NMR in $CDCl_3$ (400 MHz) **3e**

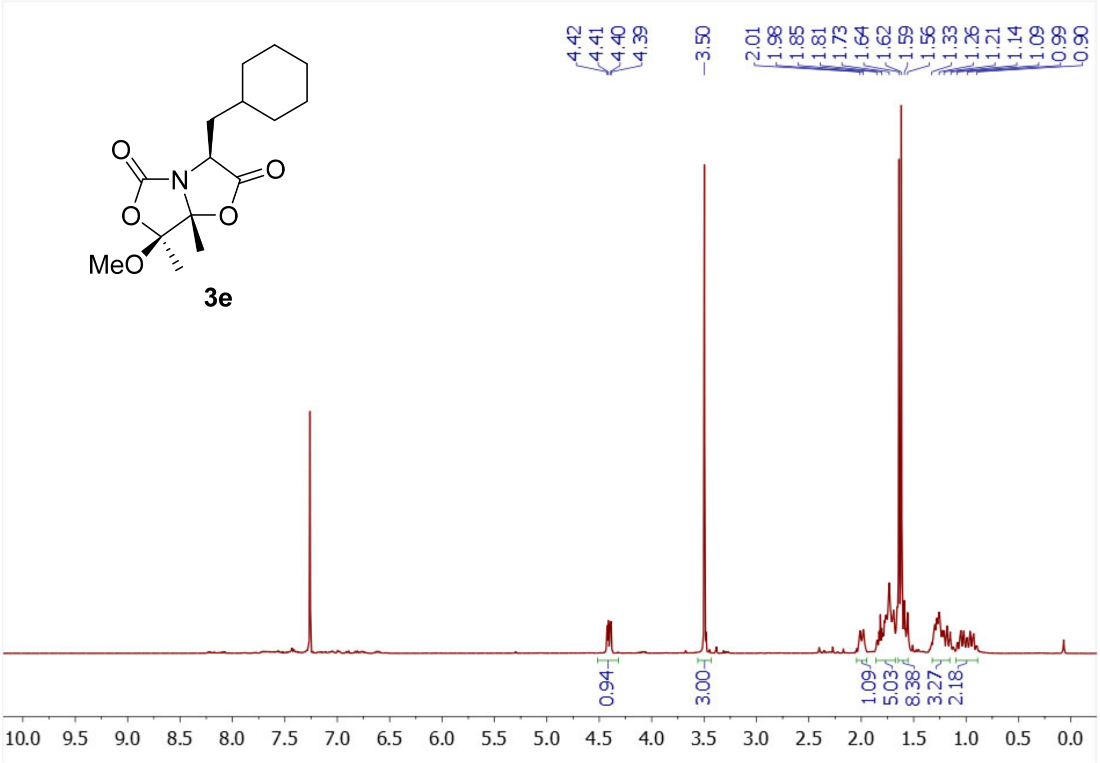

$^{13}C\{^{1}H\}$ NMR in $CDCl_3$ (100 MHz) **3e**

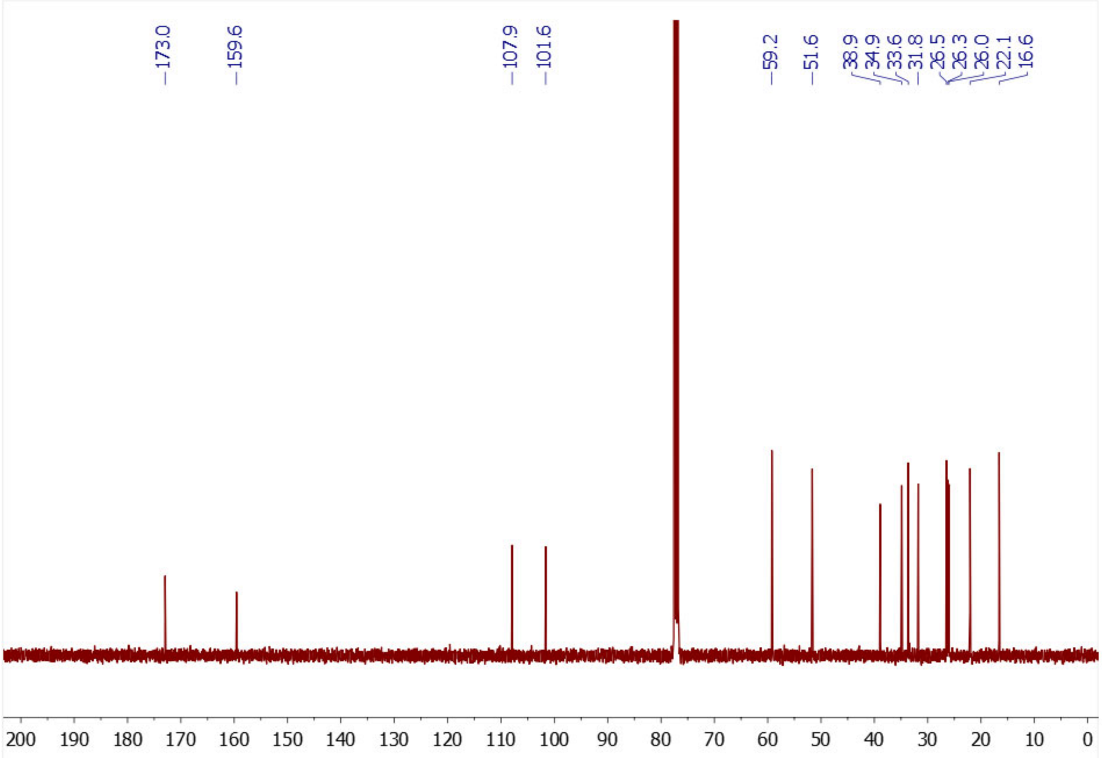

COSY in $CDCl_3$ **3e**

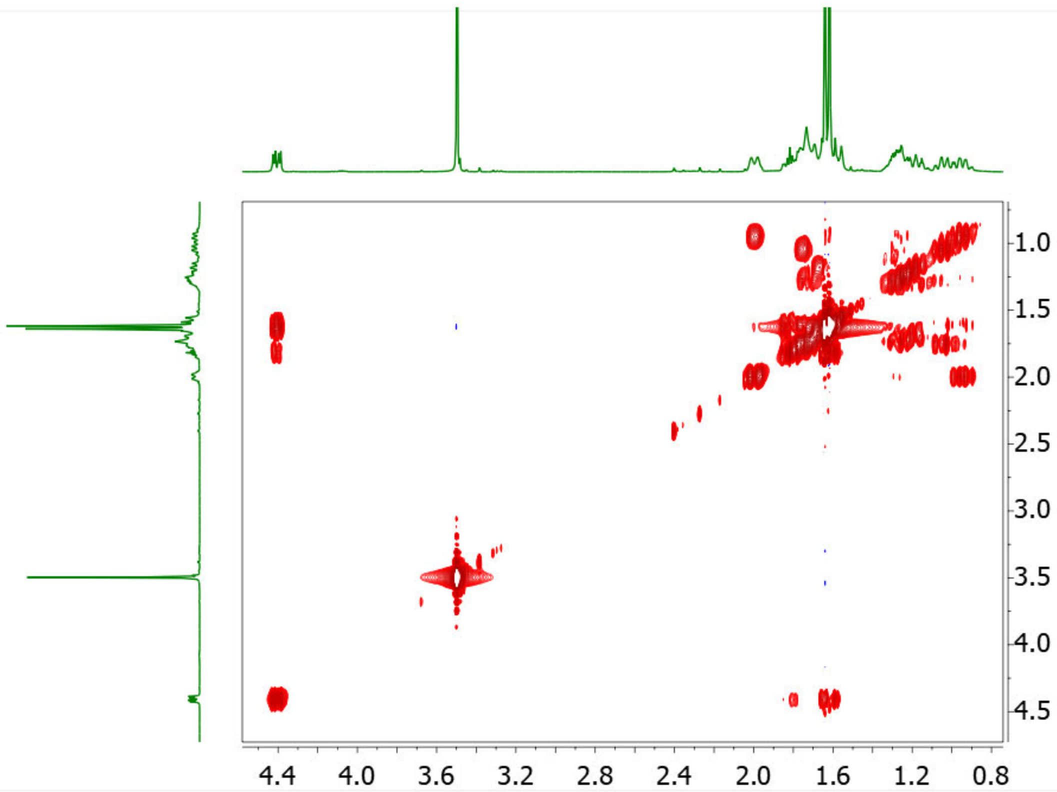

HSQC in $CDCl_3$ **3e**

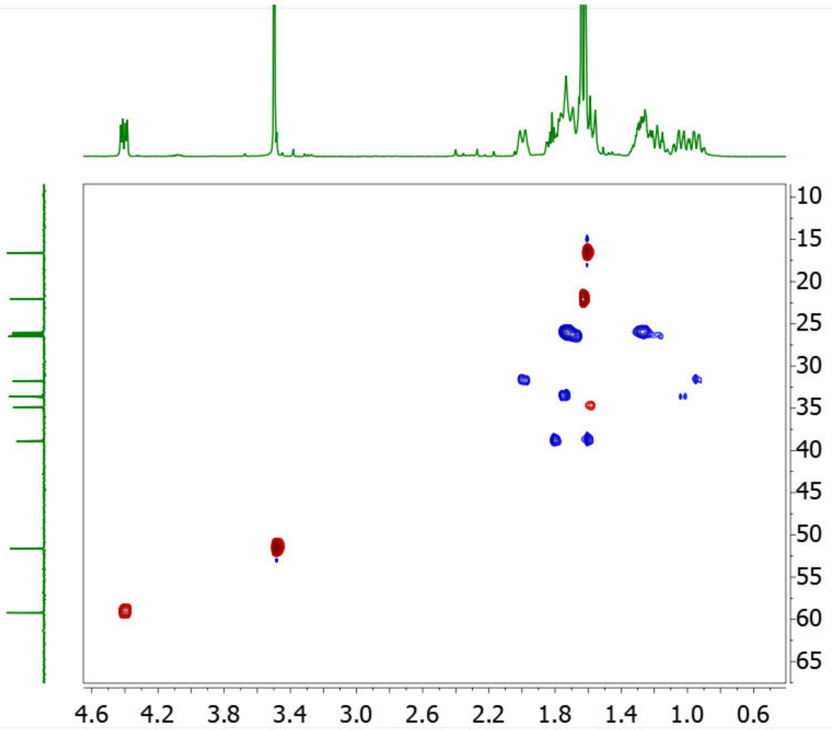

^{1}H NMR in $CDCl_3$ (400 MHz) **3f**

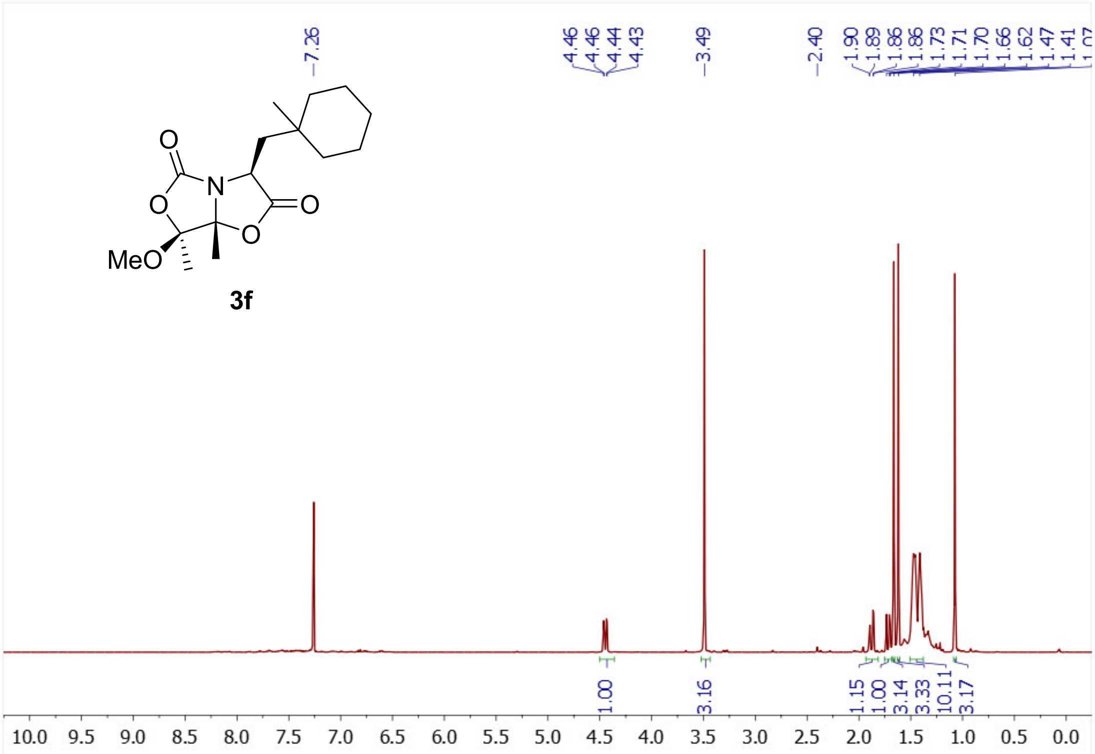

$^{13}C\{^{1}H\}$ NMR in $CDCl_3$ (100 MHz) **3f**

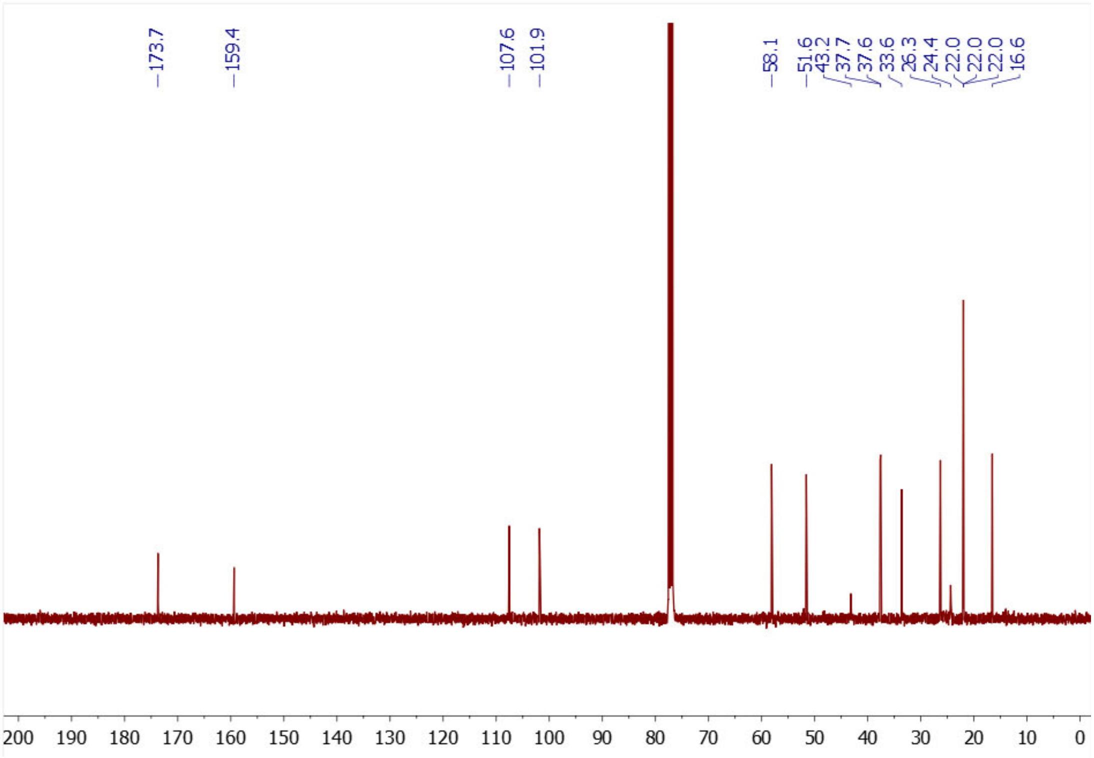

COSY in $CDCl_3$ **3f**

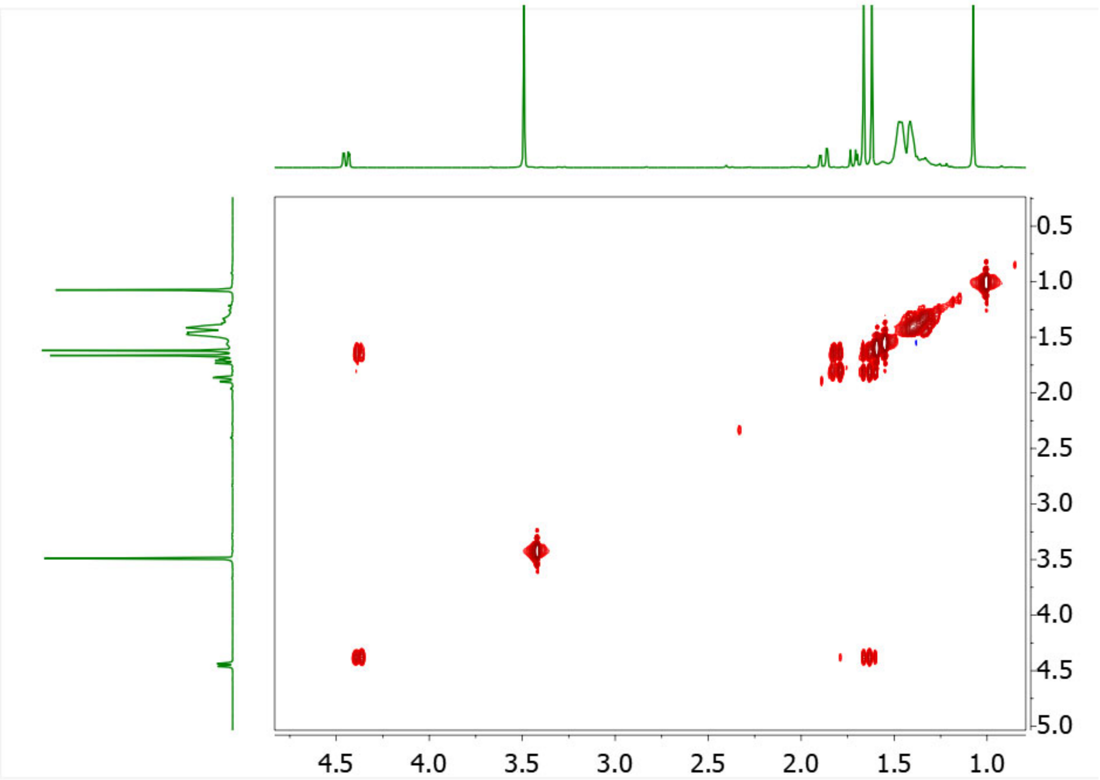

HSQC in $CDCl_3$ **3f**

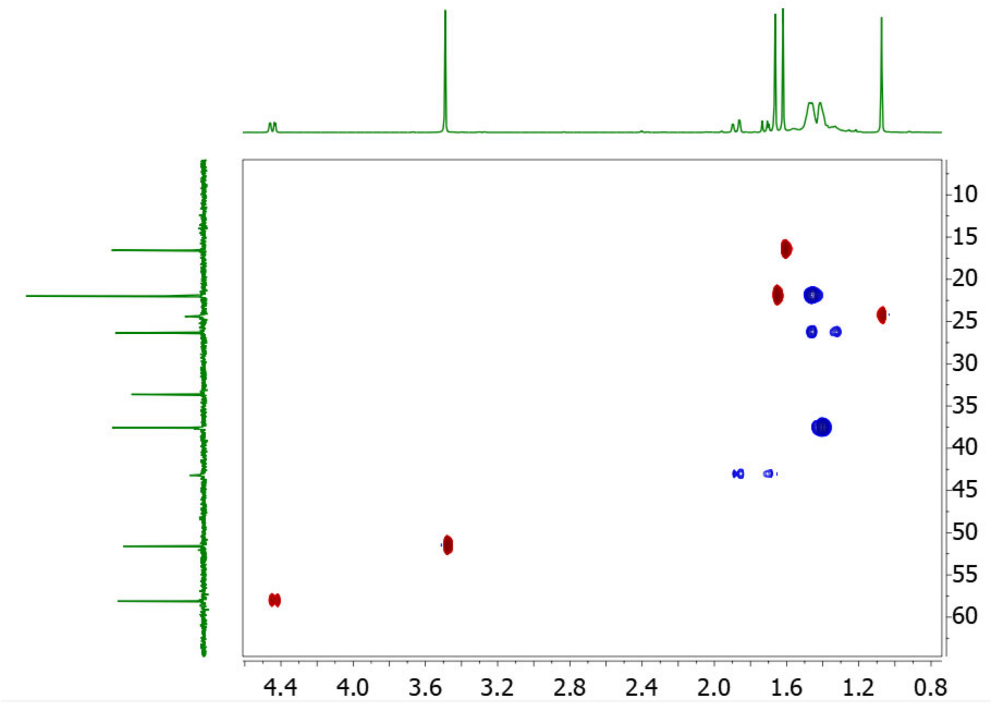

^{1}H NMR in $CDCl_3$ (400 MHz) **3g**

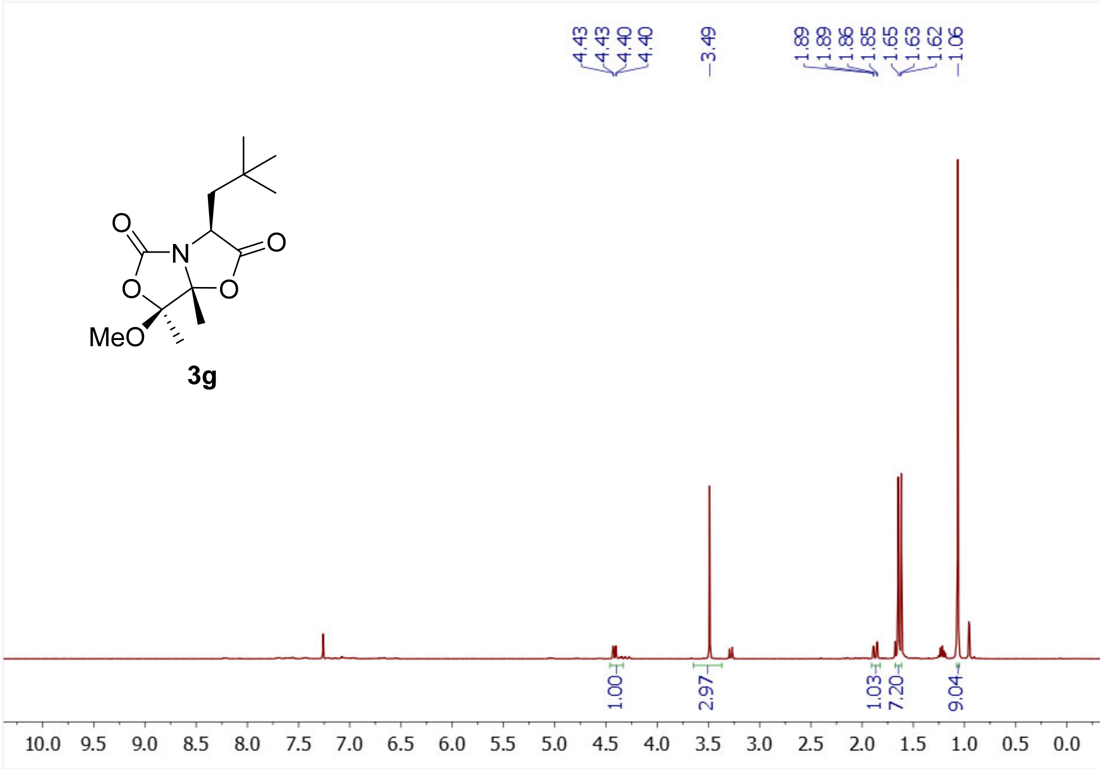

$^{13}C\{^{1}H\}$ NMR in $CDCl_3$ (100 MHz) **3g**

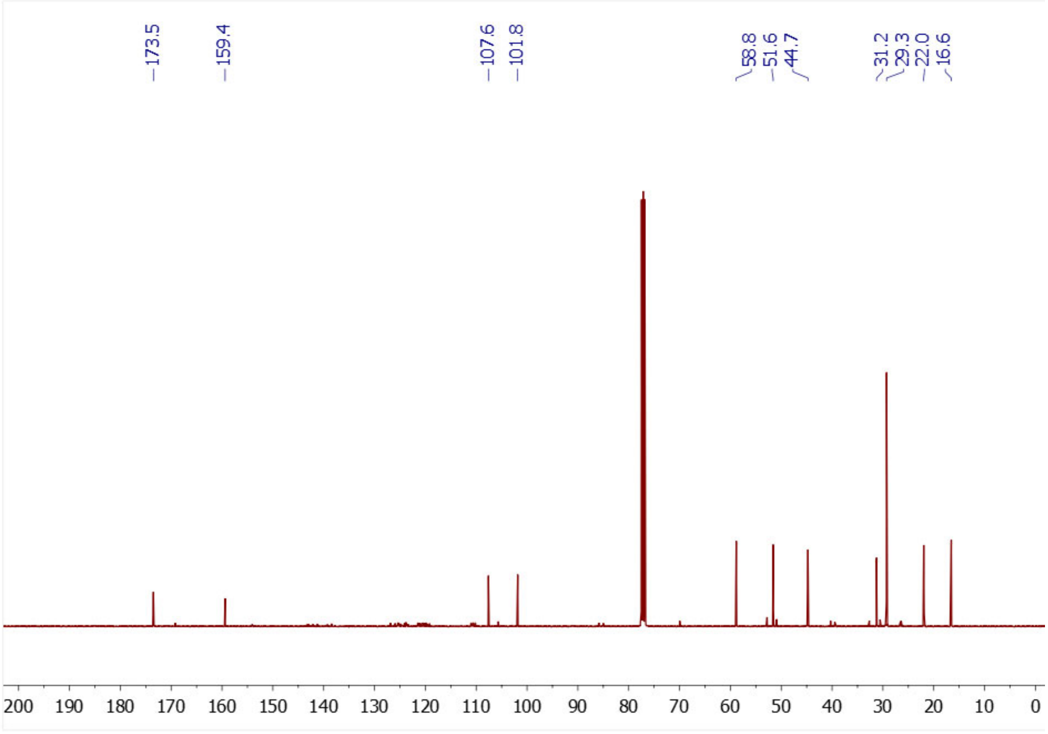

COSY in $CDCl_3$ **3g**

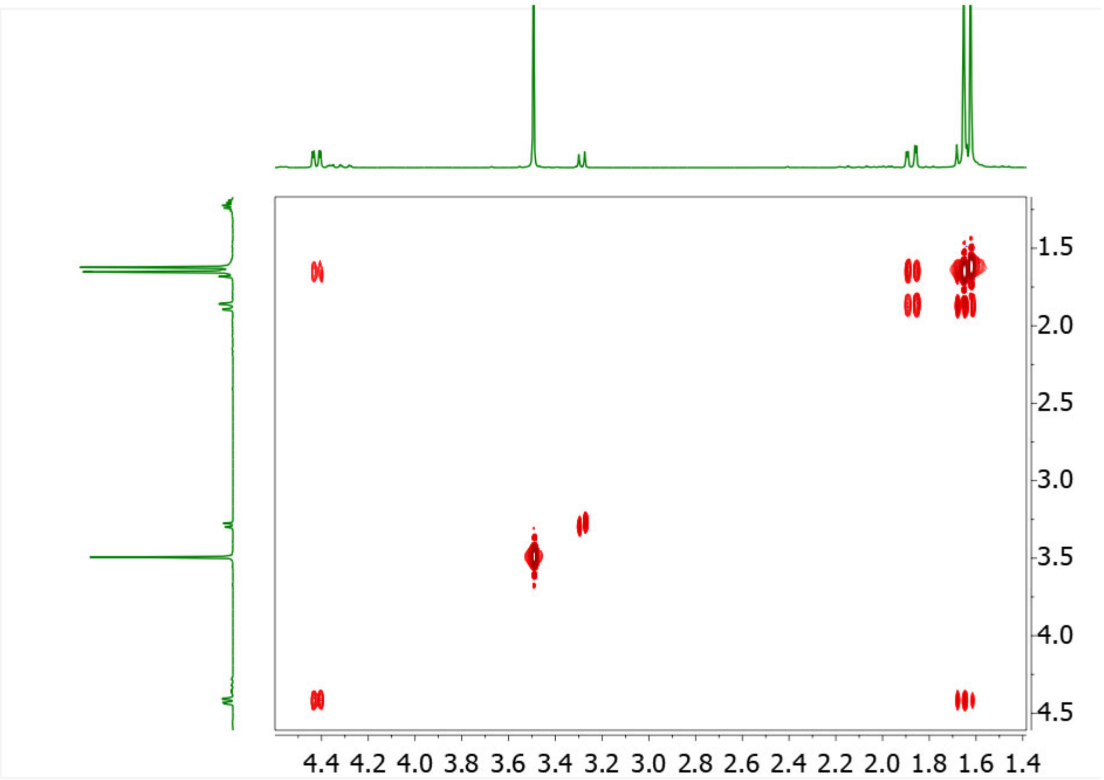

HSQC in $CDCl_3$ **3g**

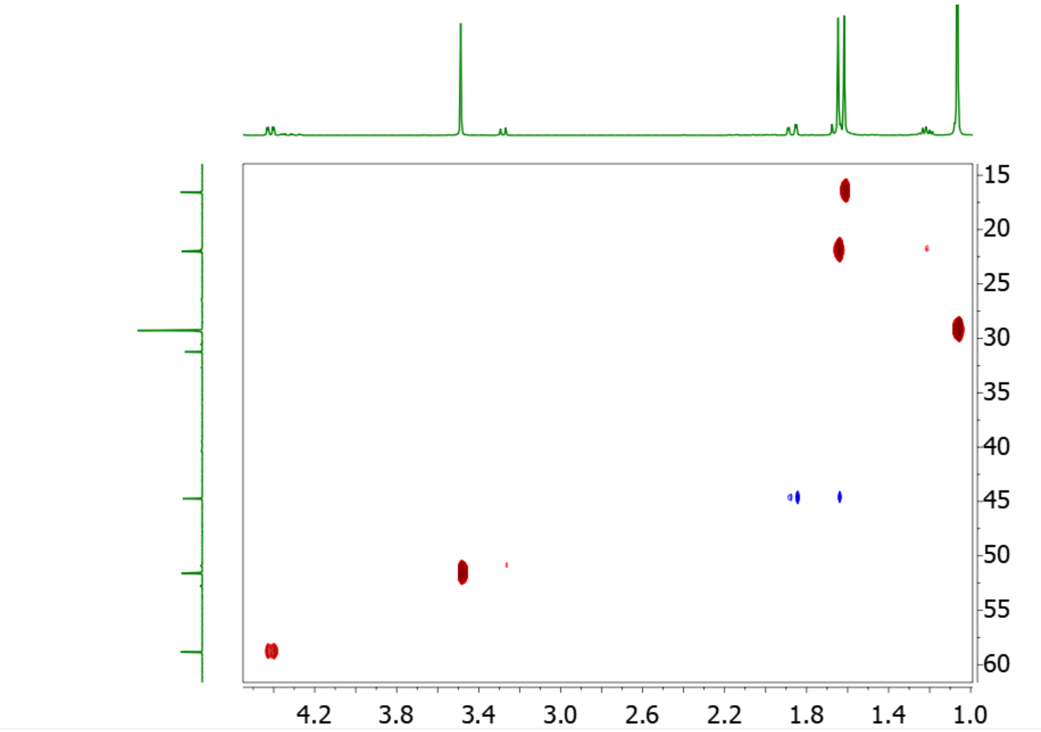

^{1}H NMR in $CDCl_3$ (400 MHz) **3h**

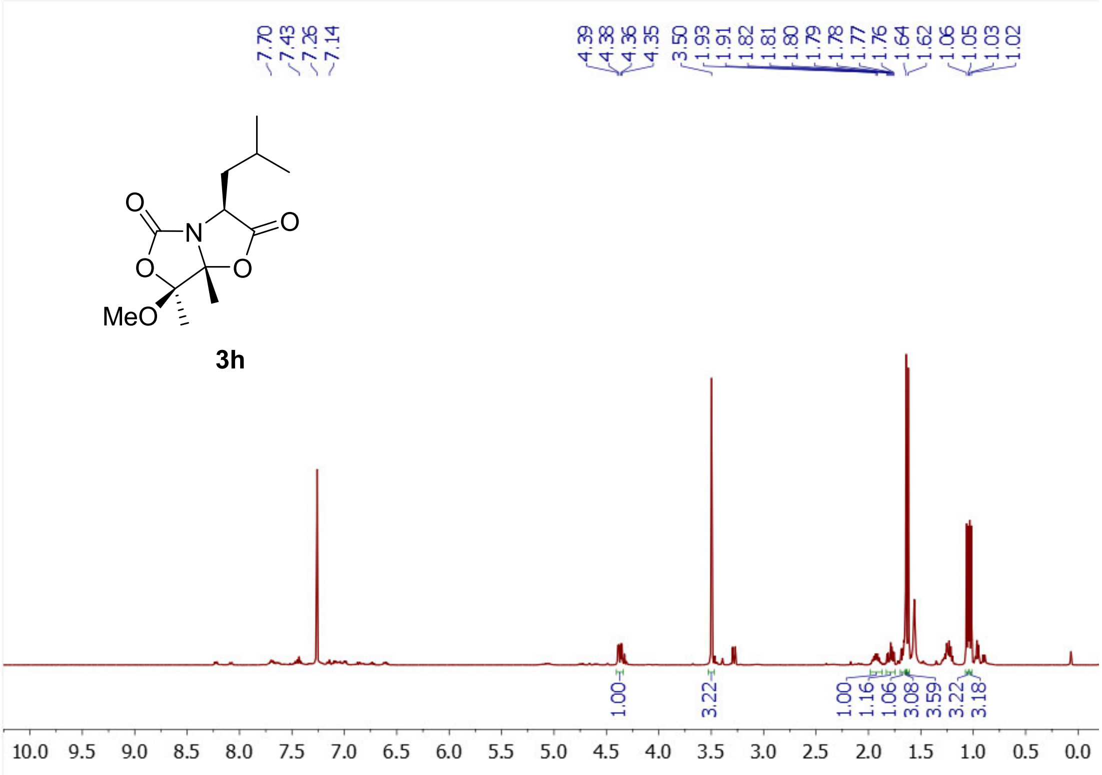

$^{13}C\{^{1}H\}$ NMR in $CDCl_3$ (100 MHz) **3h**

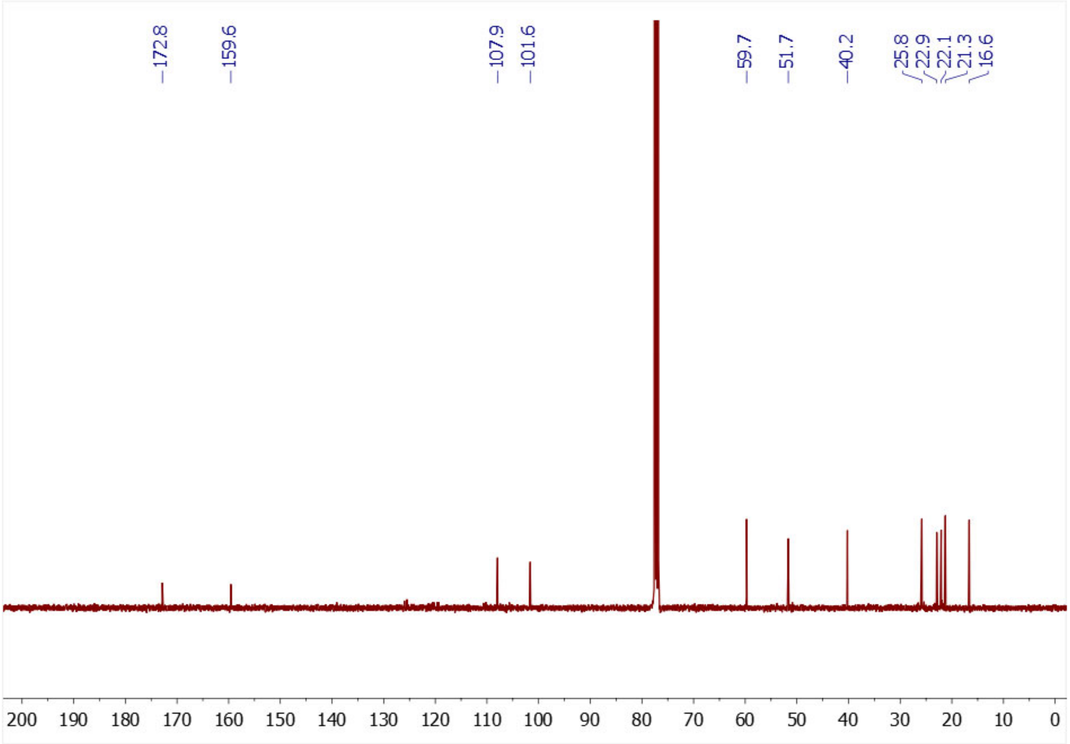

COSY in $CDCl_3$ **3h**

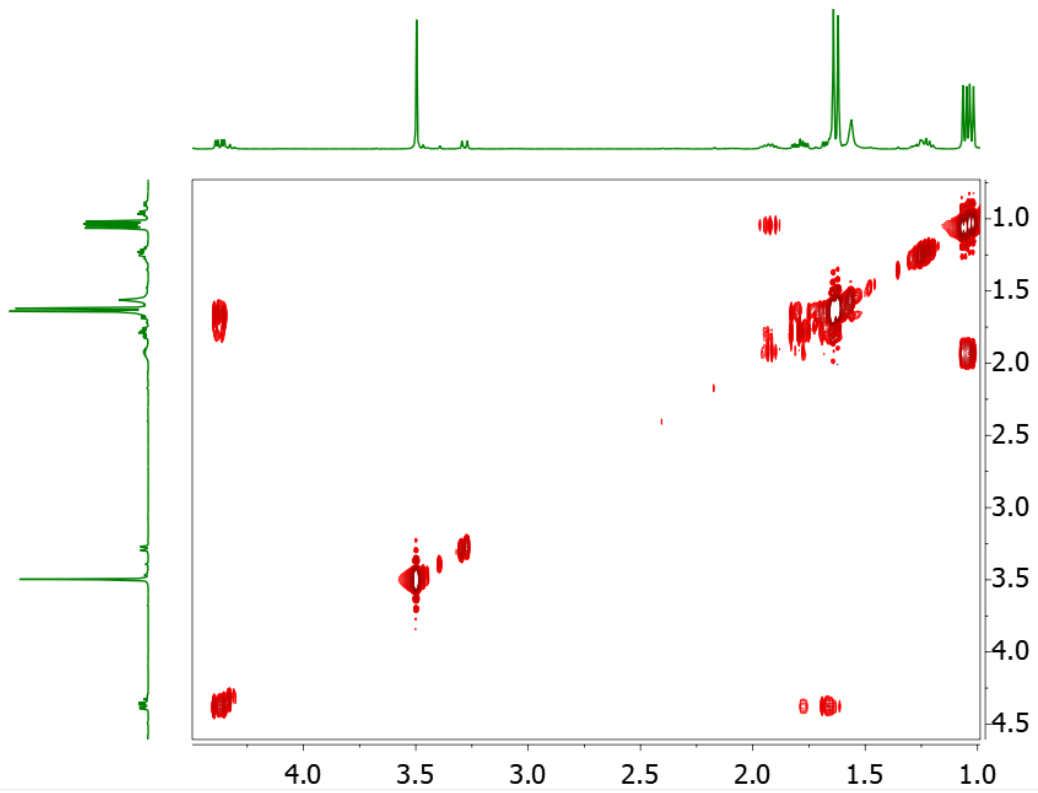

HSQC in $CDCl_3$ **3h**

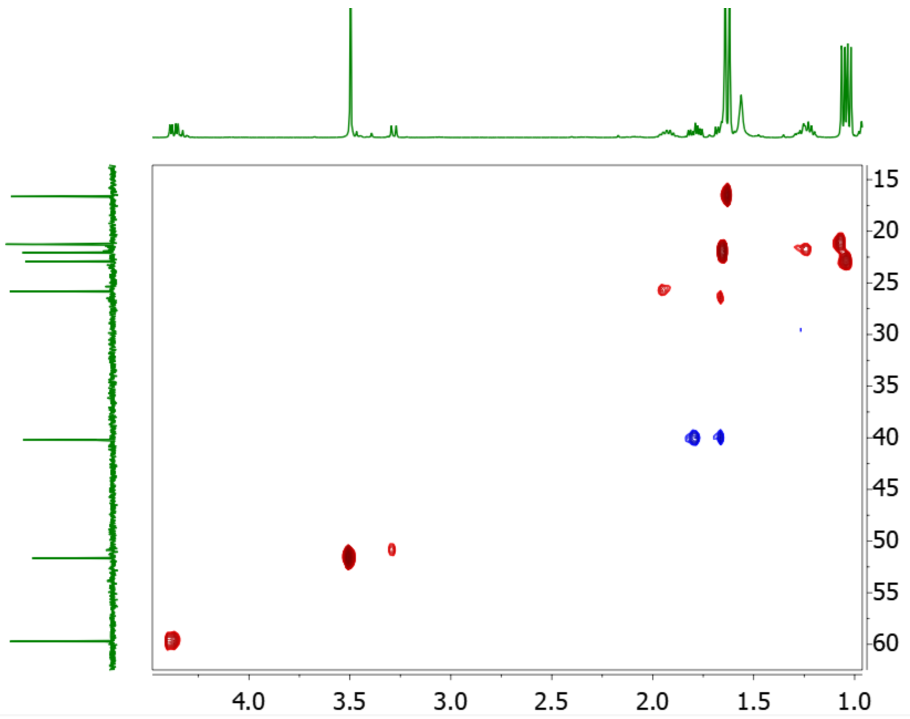

^{1}H NMR in $CDCl_3$ (400 MHz) **3i**

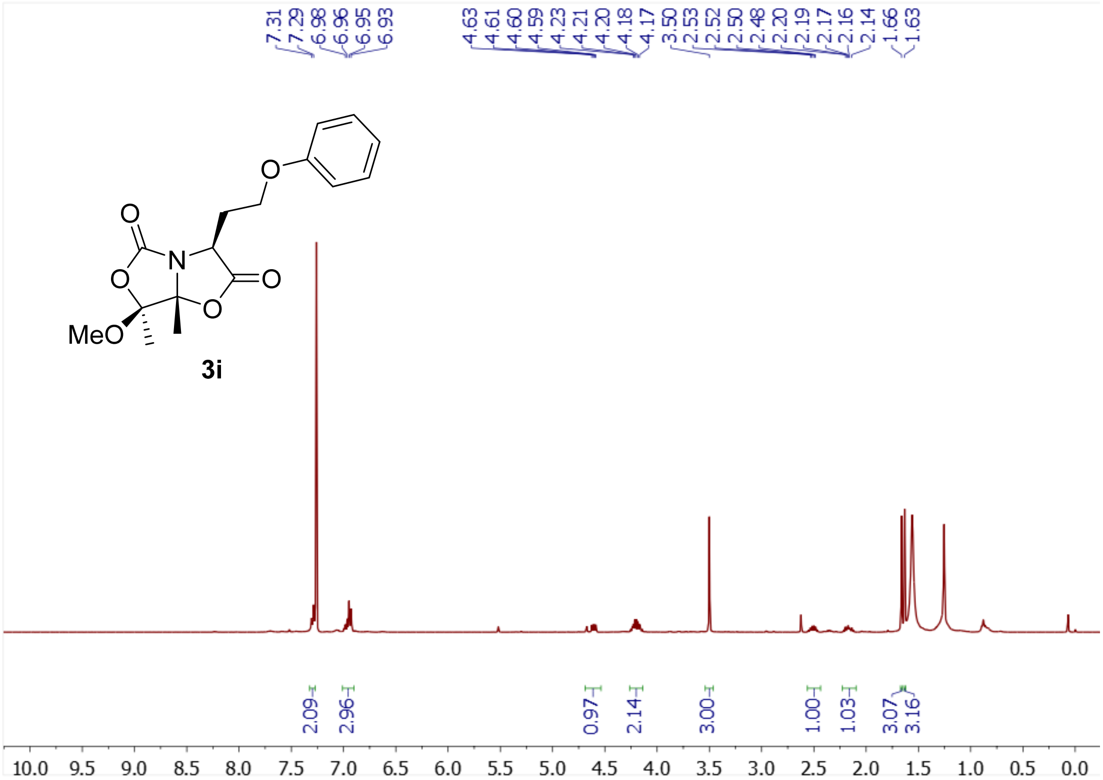

^{13}C{^{1}H} NMR in $CDCl_3$ (100 MHz) **3i**

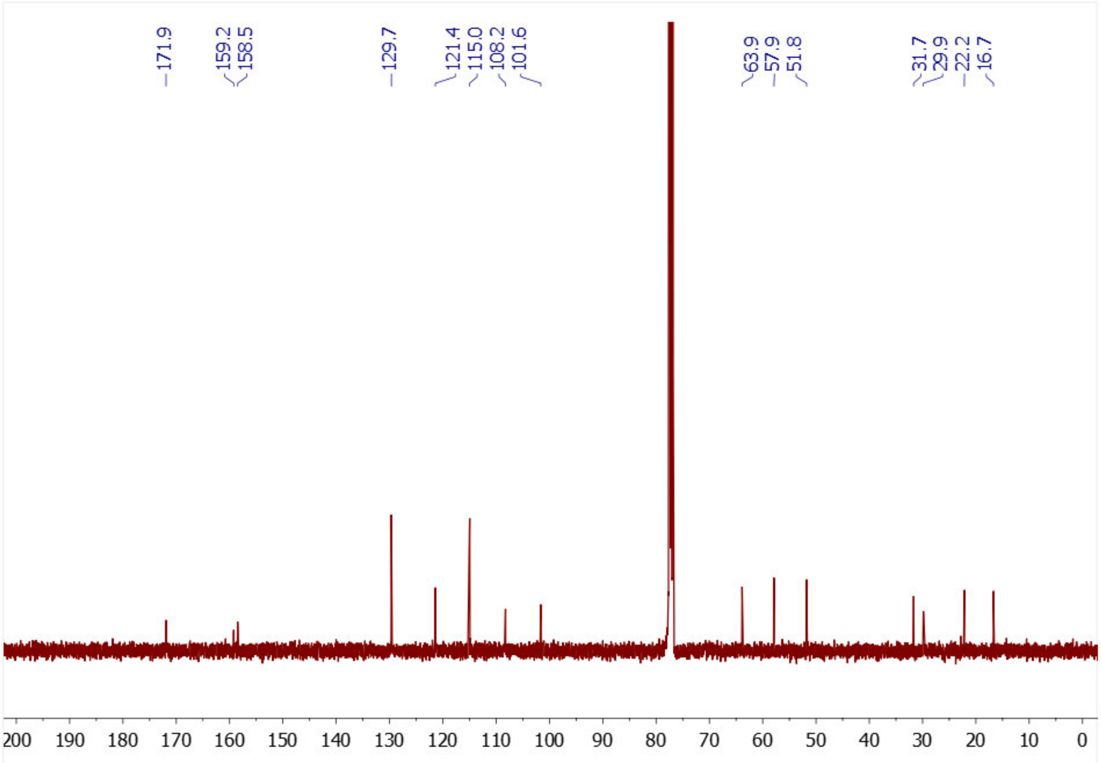

COSY in $CDCl_3$ **3i**

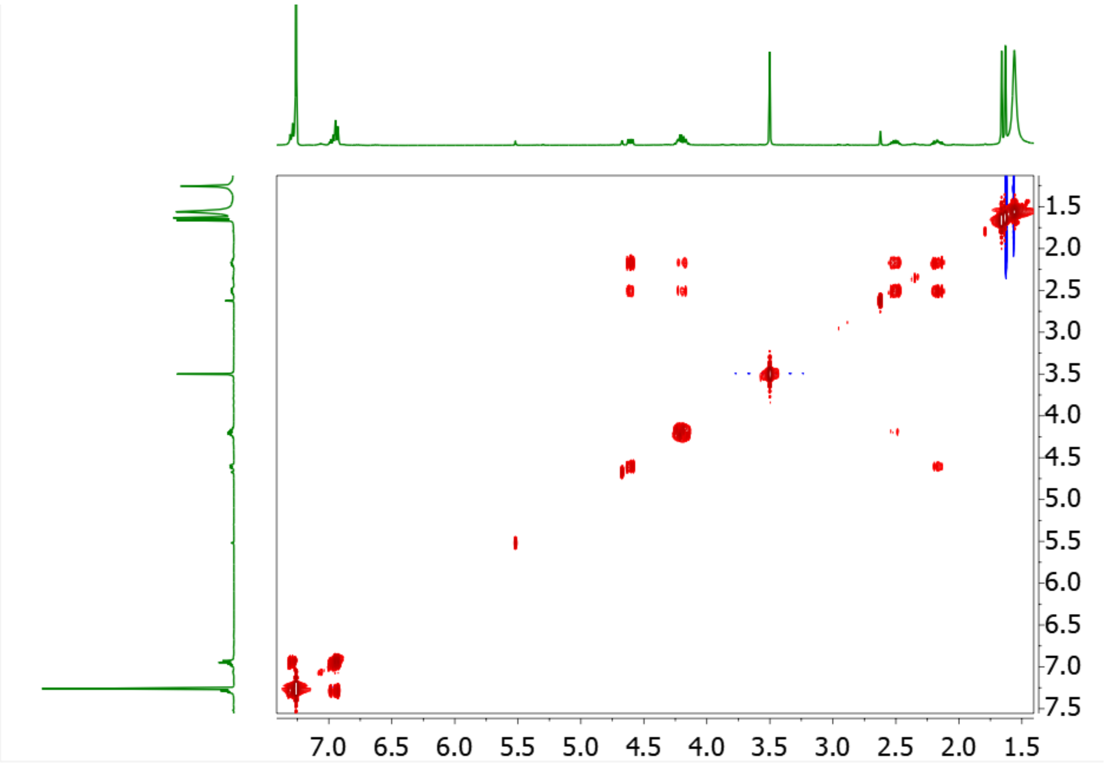

HSQC in $CDCl_3$ **3i**

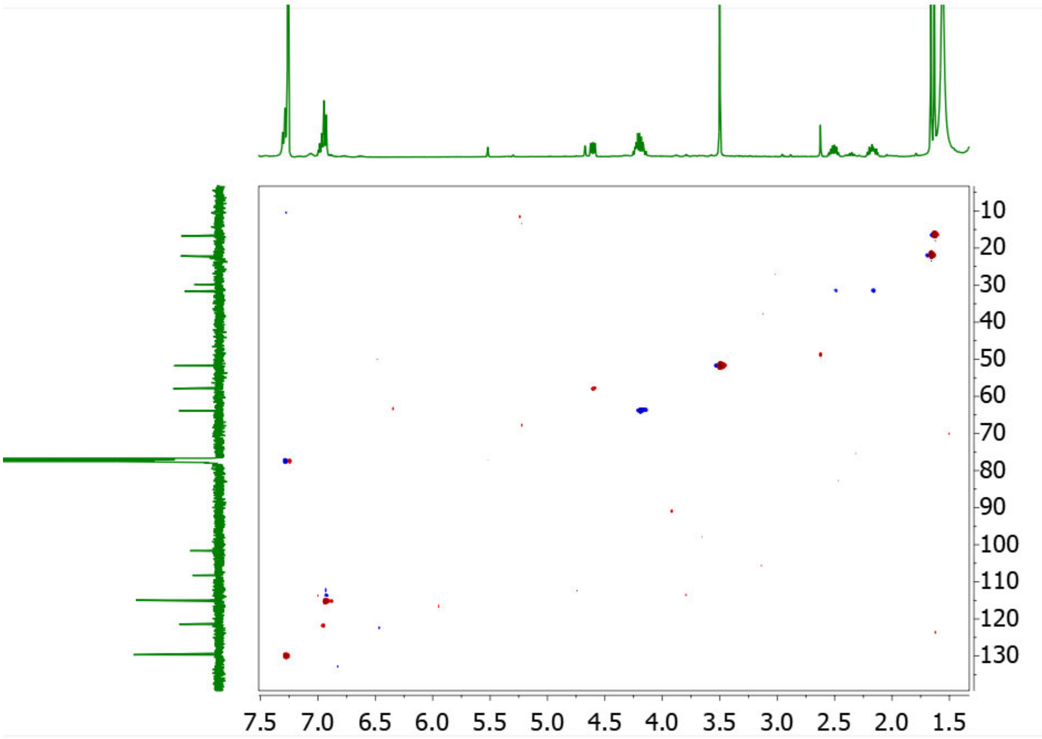

^{1}H NMR in $CDCl_3$ (400 MHz) **3j**

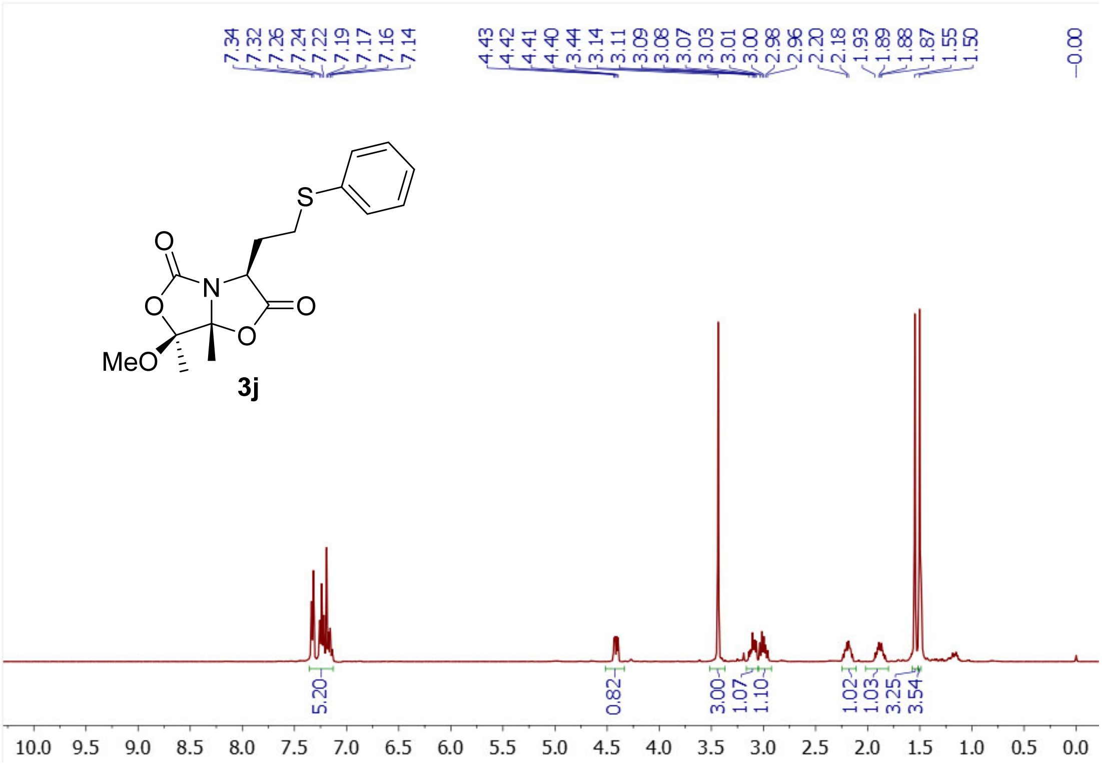

$^{13}C\{^{1}H\}$ NMR in $CDCl_3$ (100 MHz) **3j**

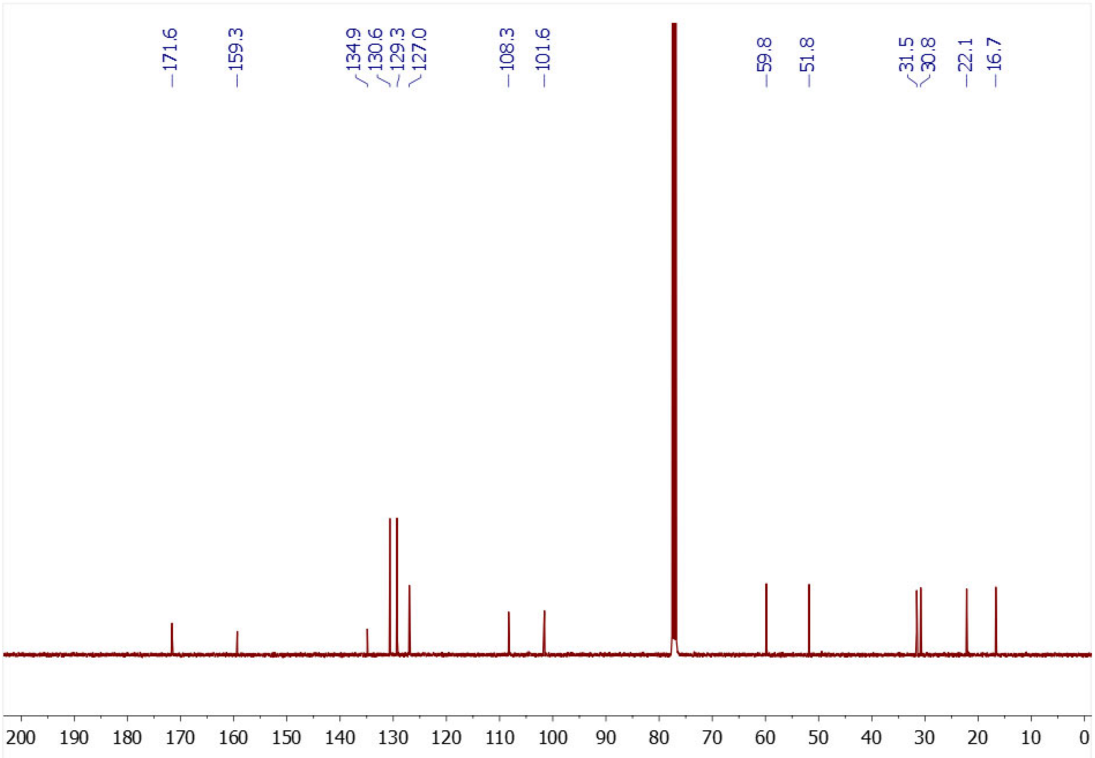

COSY in $CDCl_3$ **3j**

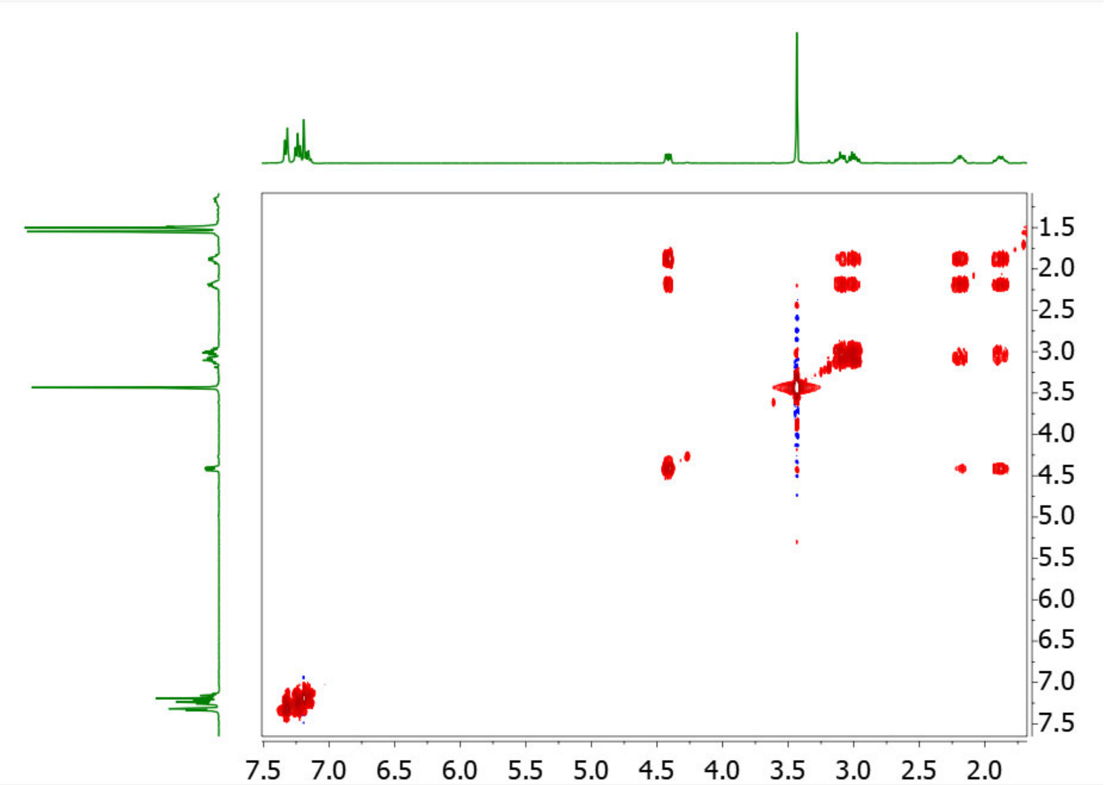

HSQC in $CDCl_3$ **3j**

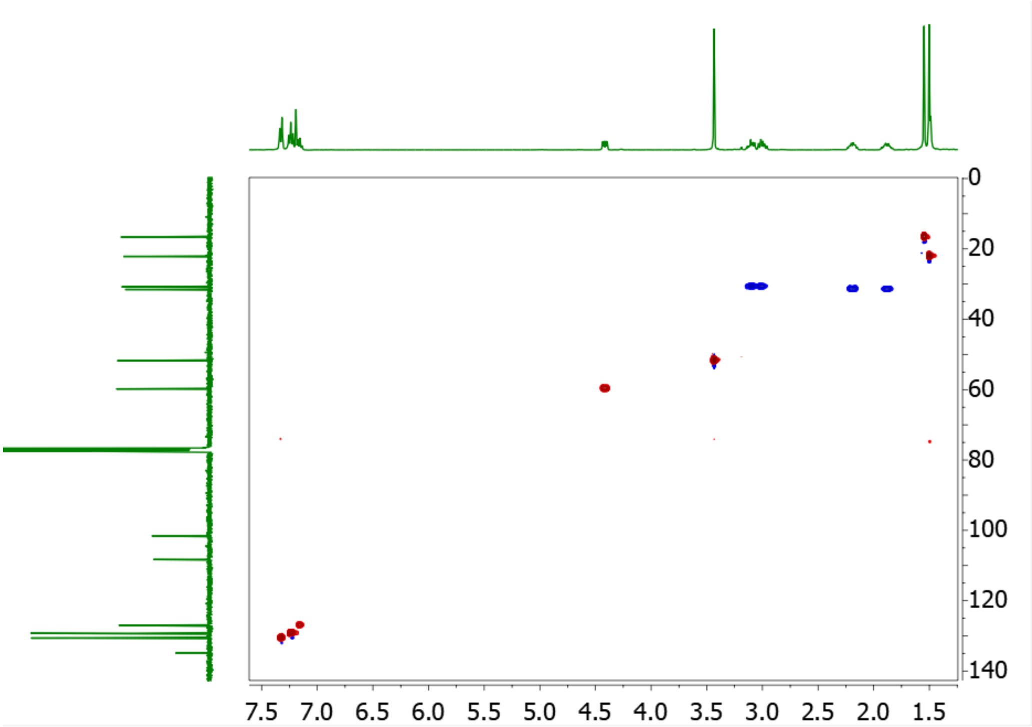

^{1}H NMR in $CDCl_3$ (400 MHz) **3k**

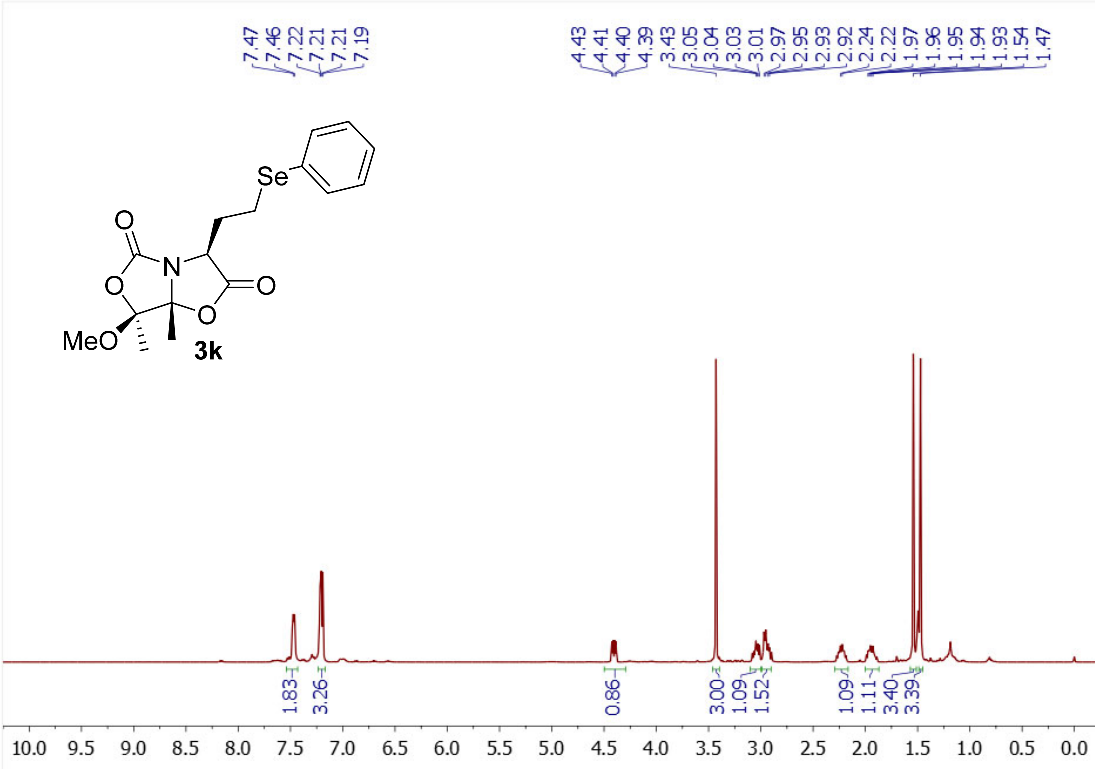

$^{13}C\{^1H\}$ NMR in $CDCl_3$ (100 MHz) **3k**

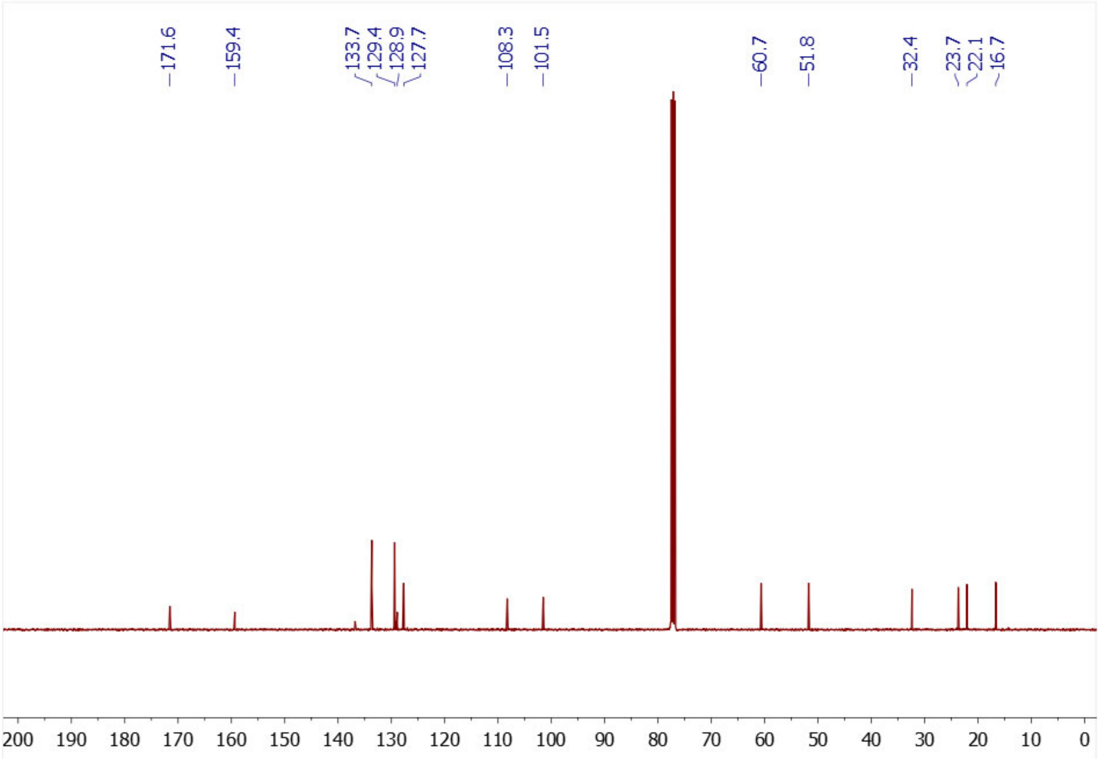

COSY in $CDCl_3$ **3k**

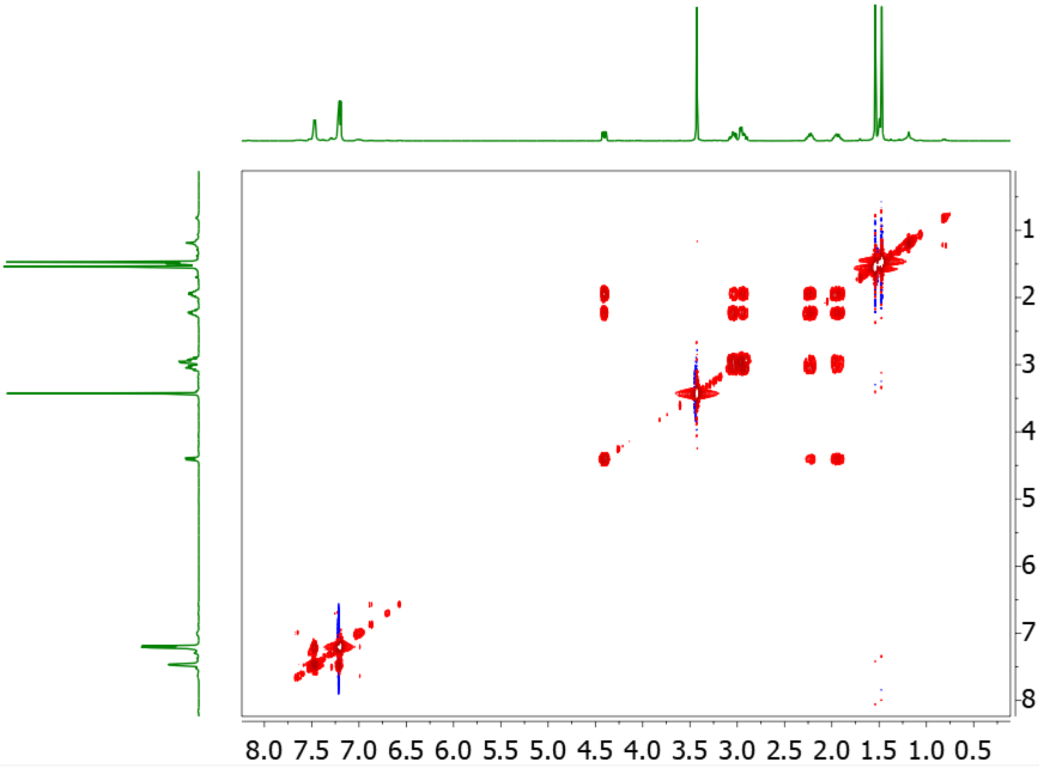

HSQC in $CDCl_3$ **3k**

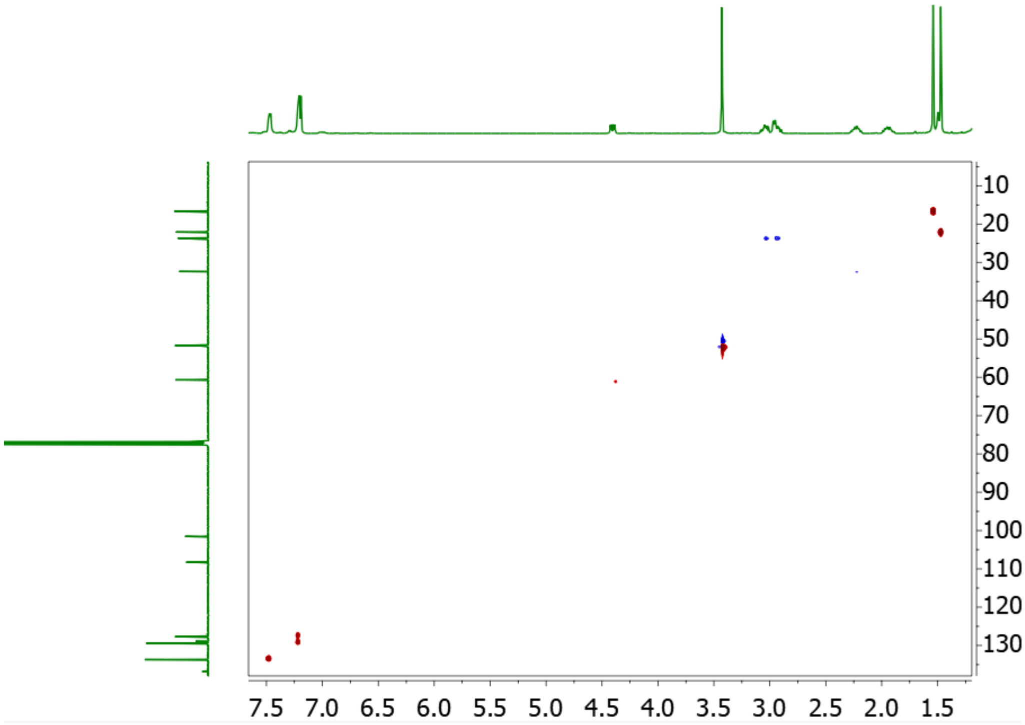

^{1}H NMR in $CDCl_3$ (400 MHz) **3l**

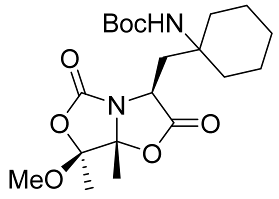

3l

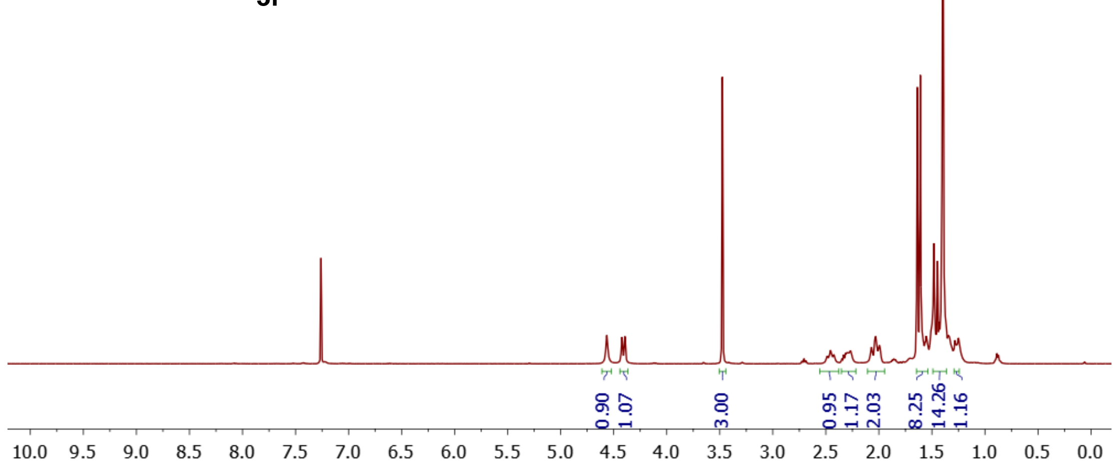

$^{13}C\{^{1}H\}$ NMR in $CDCl_3$ (100 MHz) **3l**

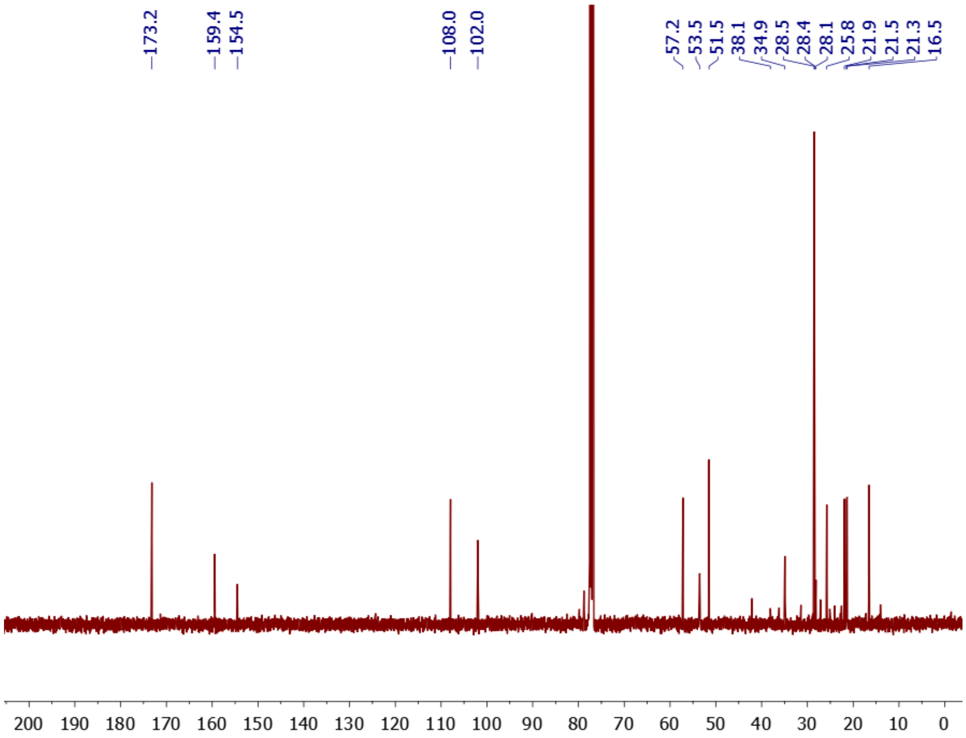

COSY in $CDCl_3$ **3l**

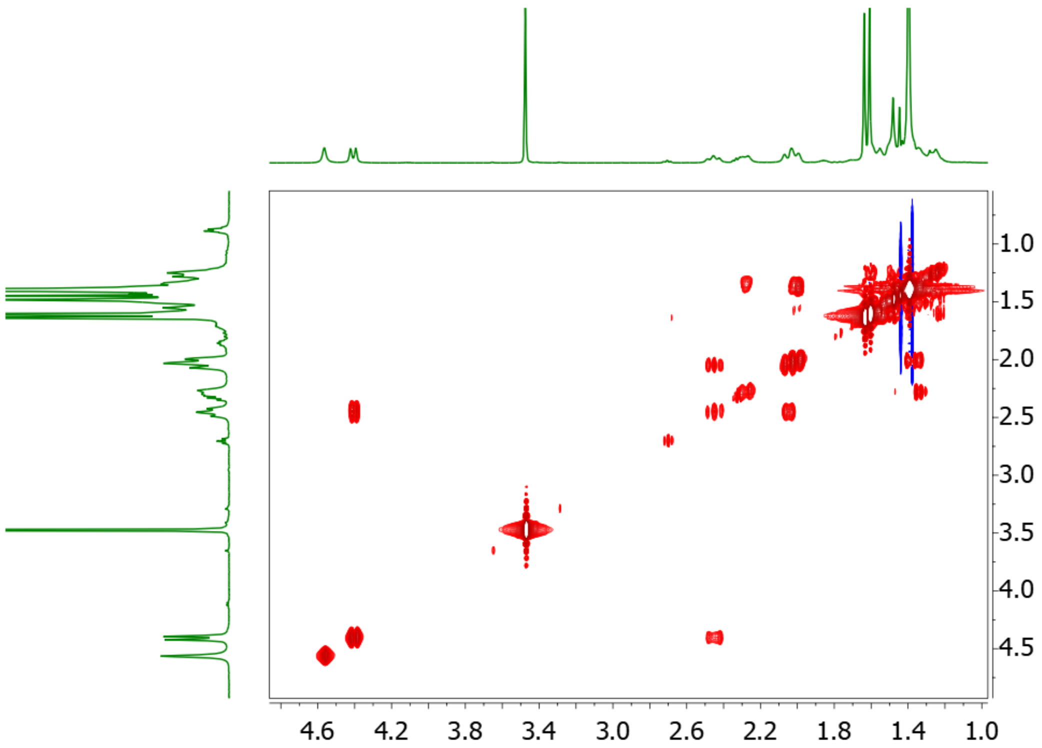

HSQC in $CDCl_3$ **3l**

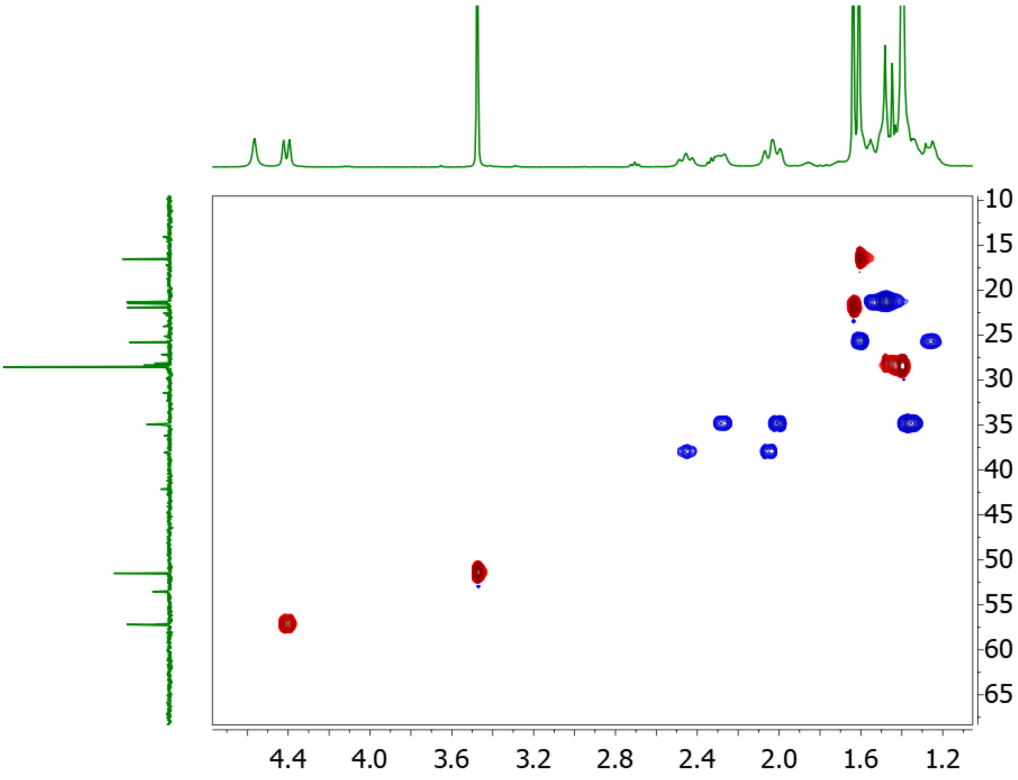

^{1}H NMR in $CDCl_3$ (400 MHz) **3m**

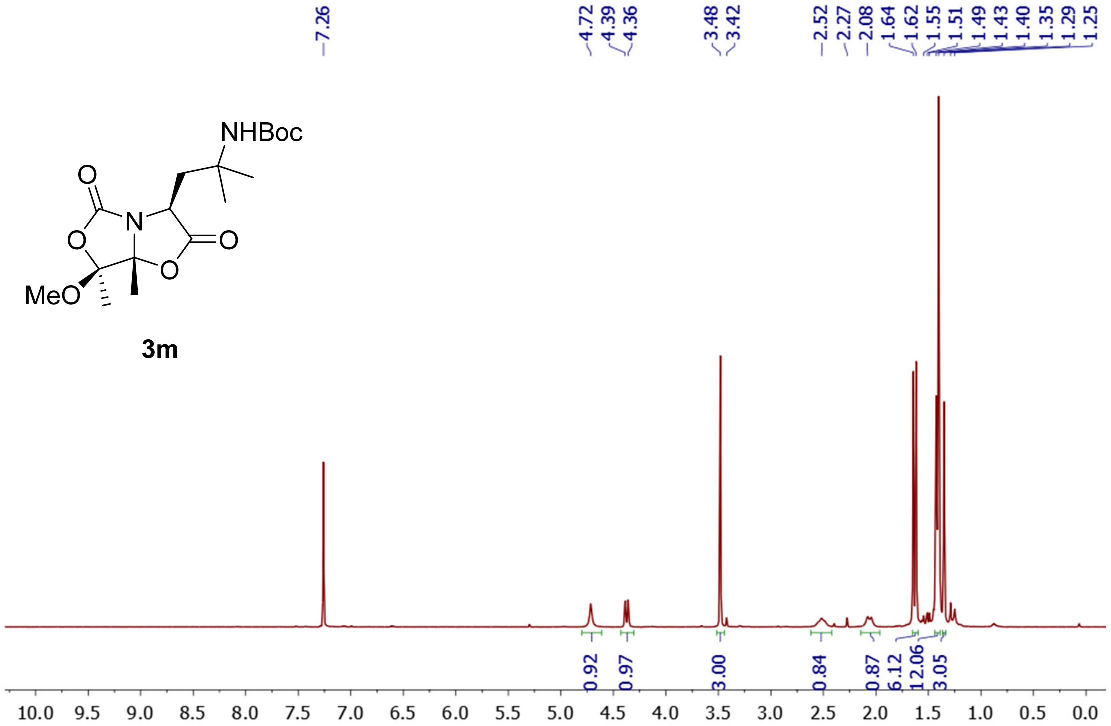

$^{13}C\{^1H\}$ NMR in $CDCl_3$ (100 MHz) **3m**

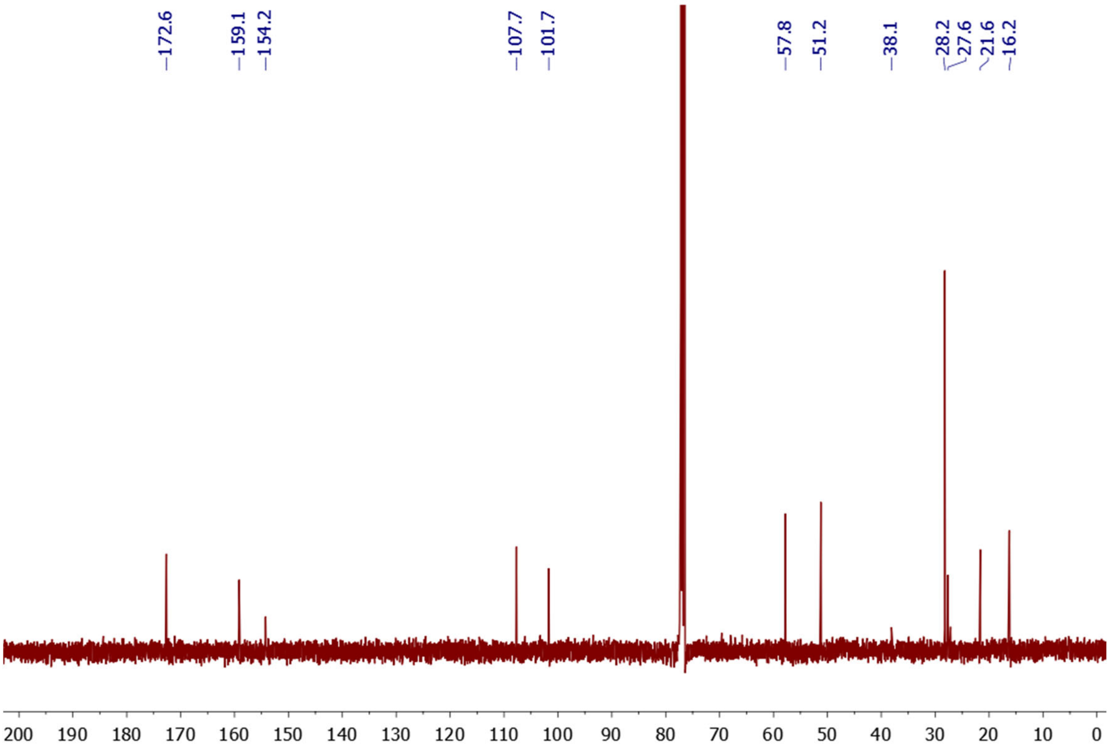

COSY in $CDCl_3$ **3m**

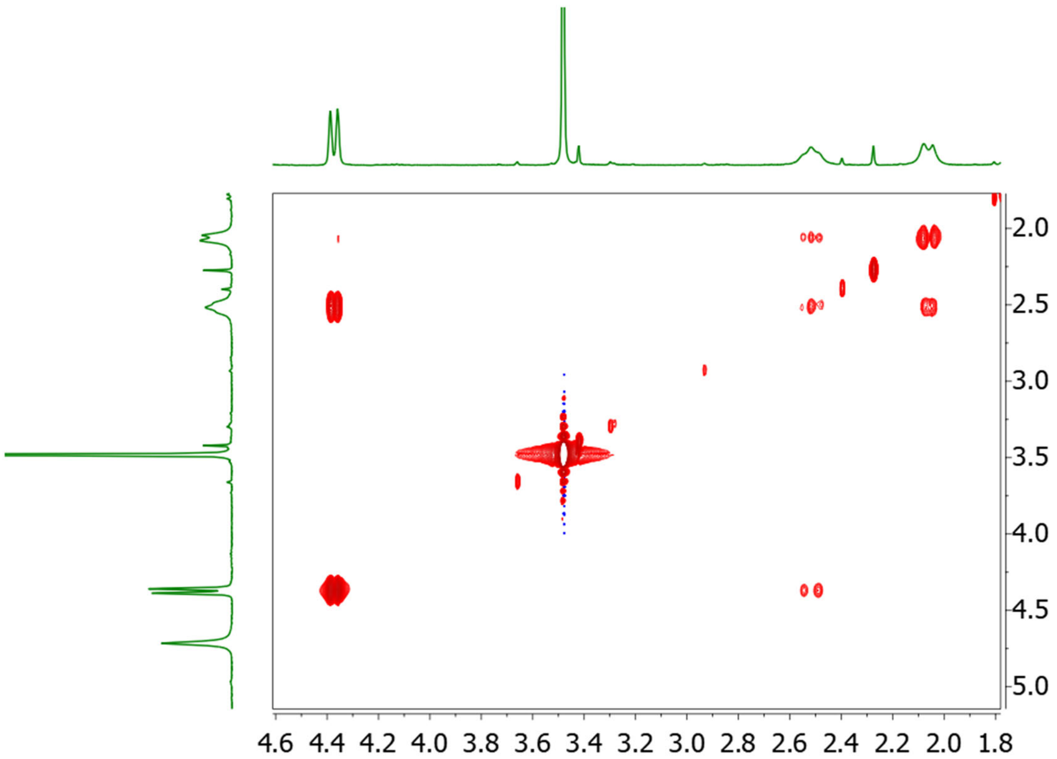

HSQC in $CDCl_3$ **3m**

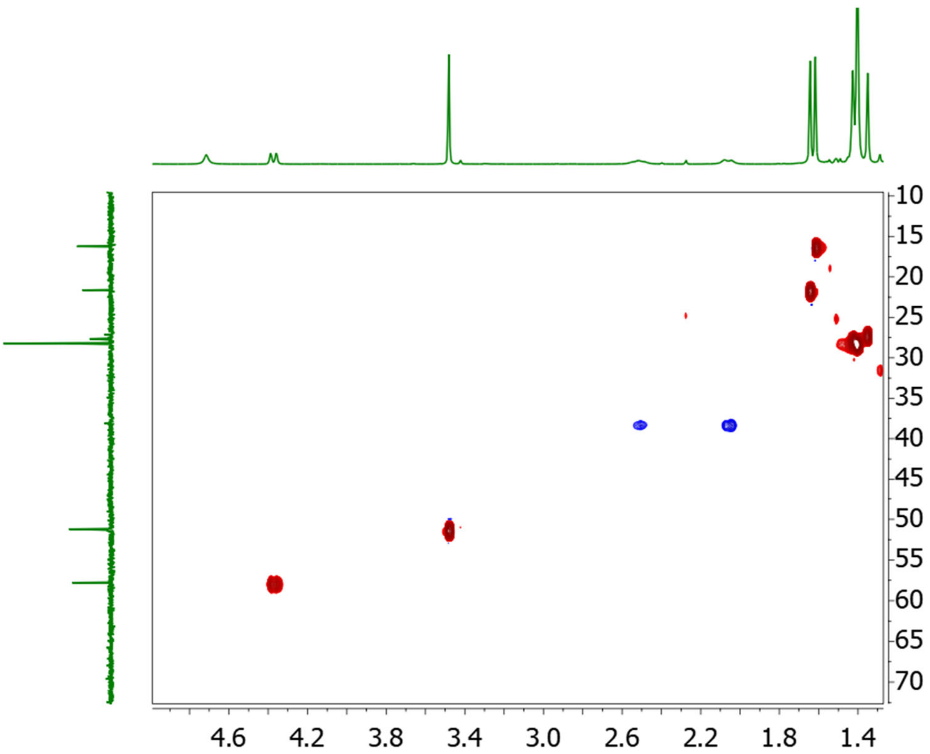

^{1}H NMR in $CDCl_3$ (400 MHz) **3n**

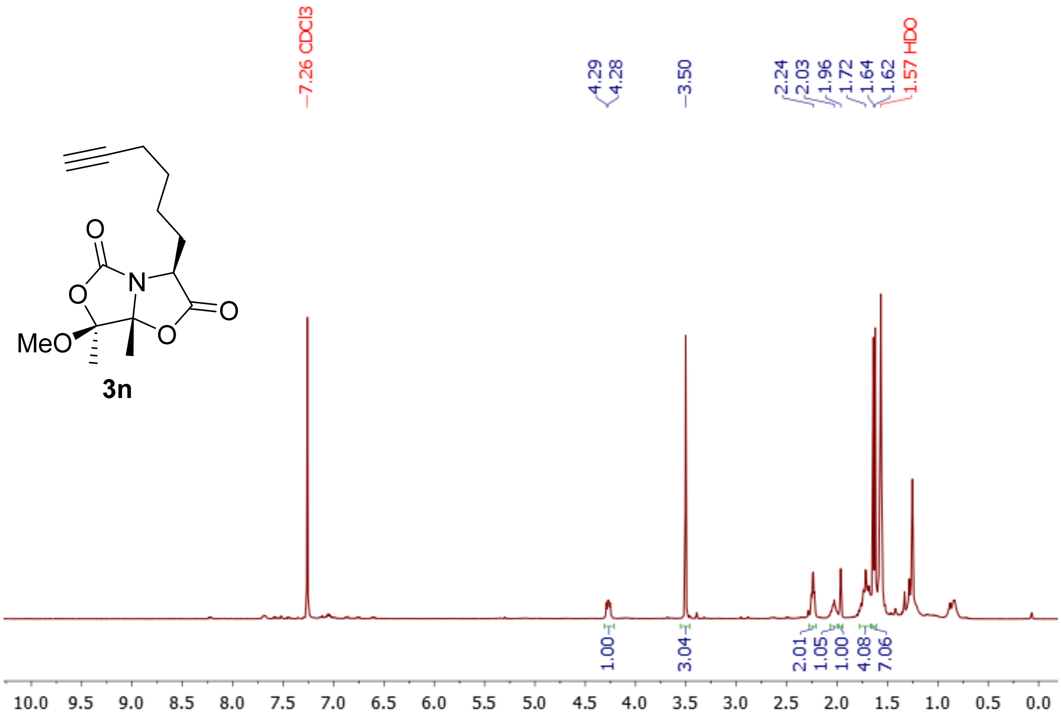

$^{13}C\{^{1}H\}$ NMR in $CDCl_3$ (100 MHz) **3n**

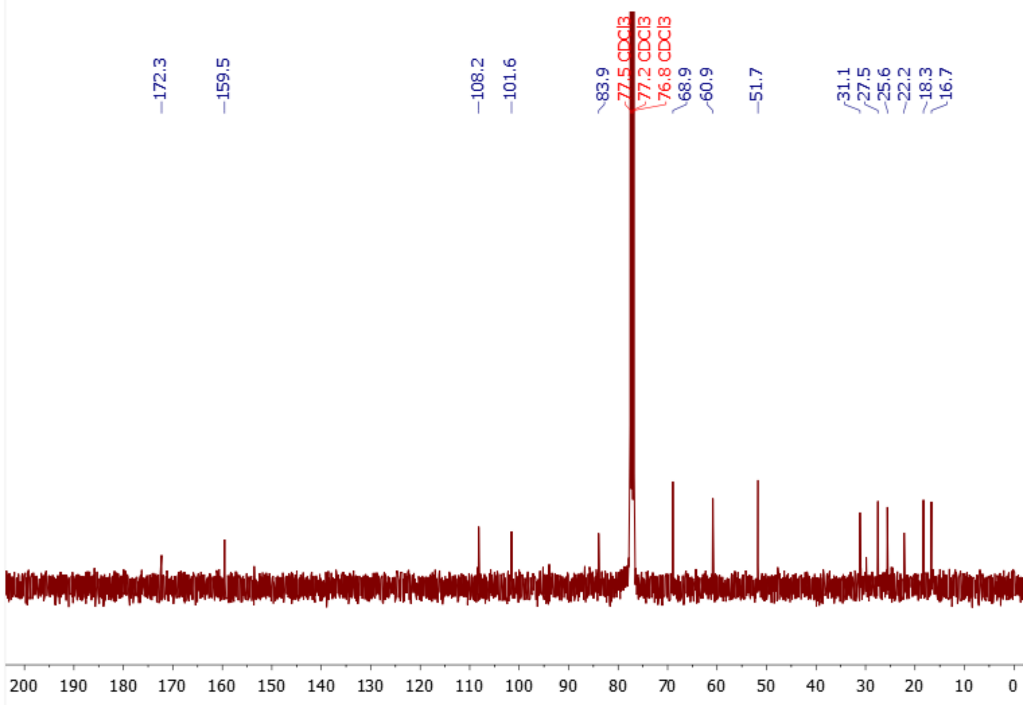

COSY in $CDCl_3$ **3n**

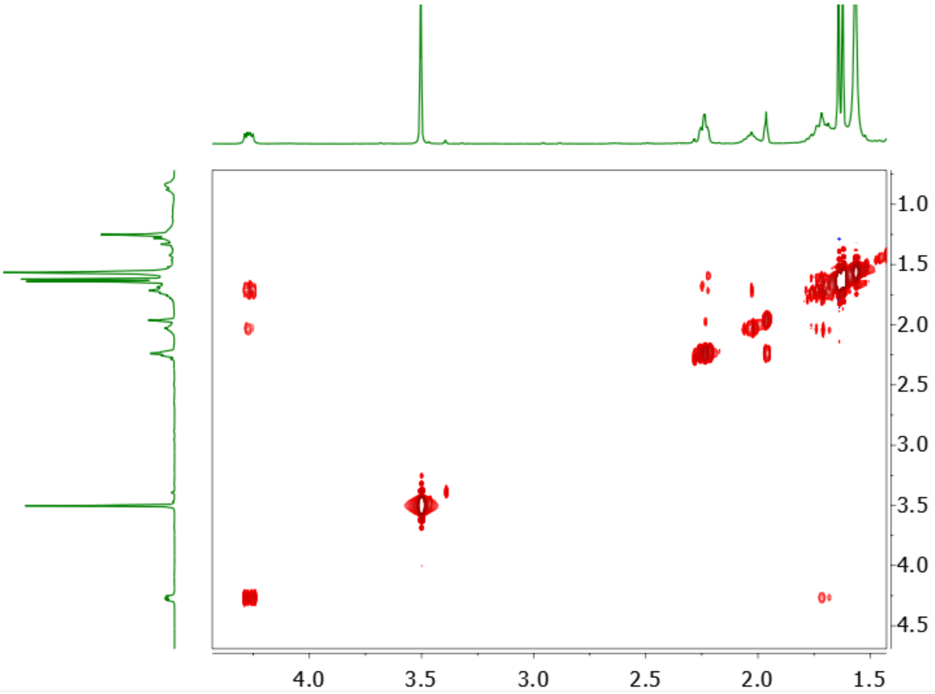

HSQC in $CDCl_3$ **3n**

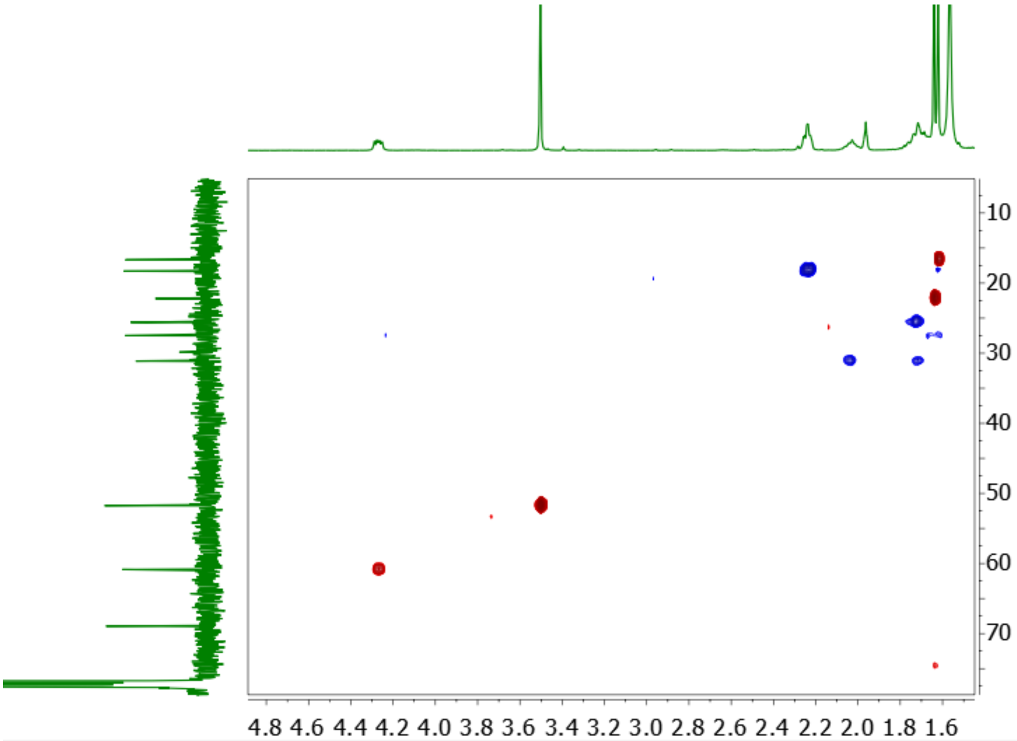

^{1}H NMR in D_2O (400 MHz) **4c**

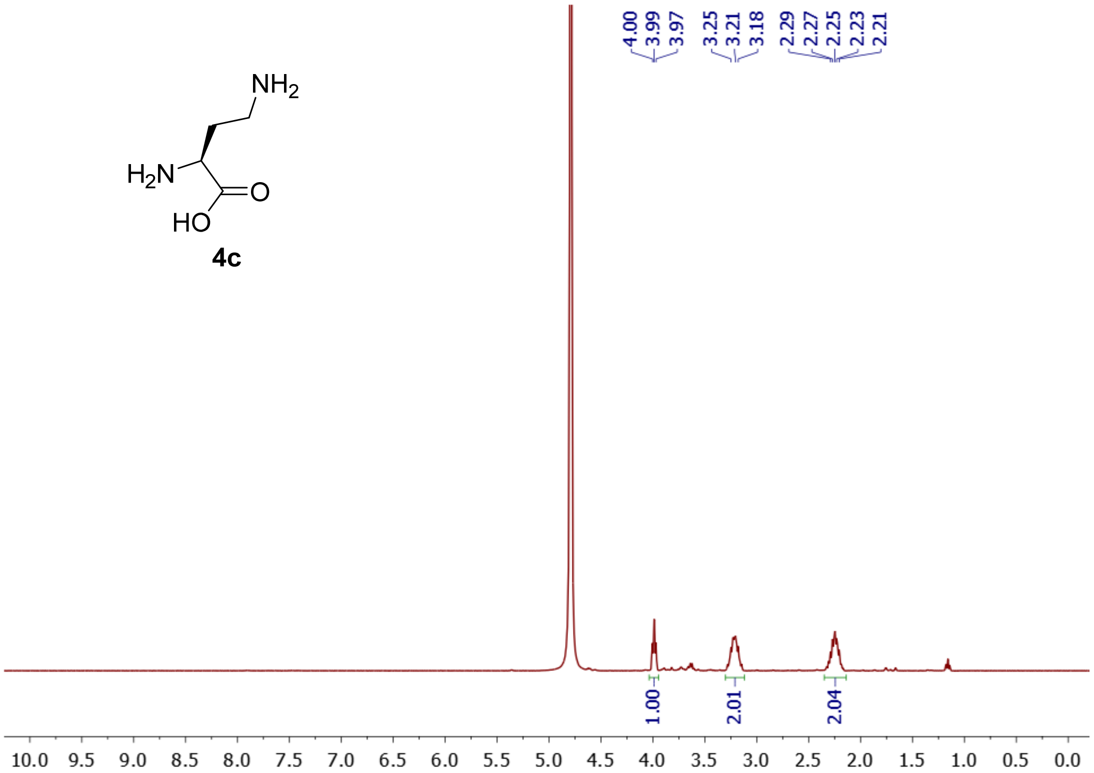

^{13}C{^{1}H} NMR in D_2O (100 MHz) **4c**

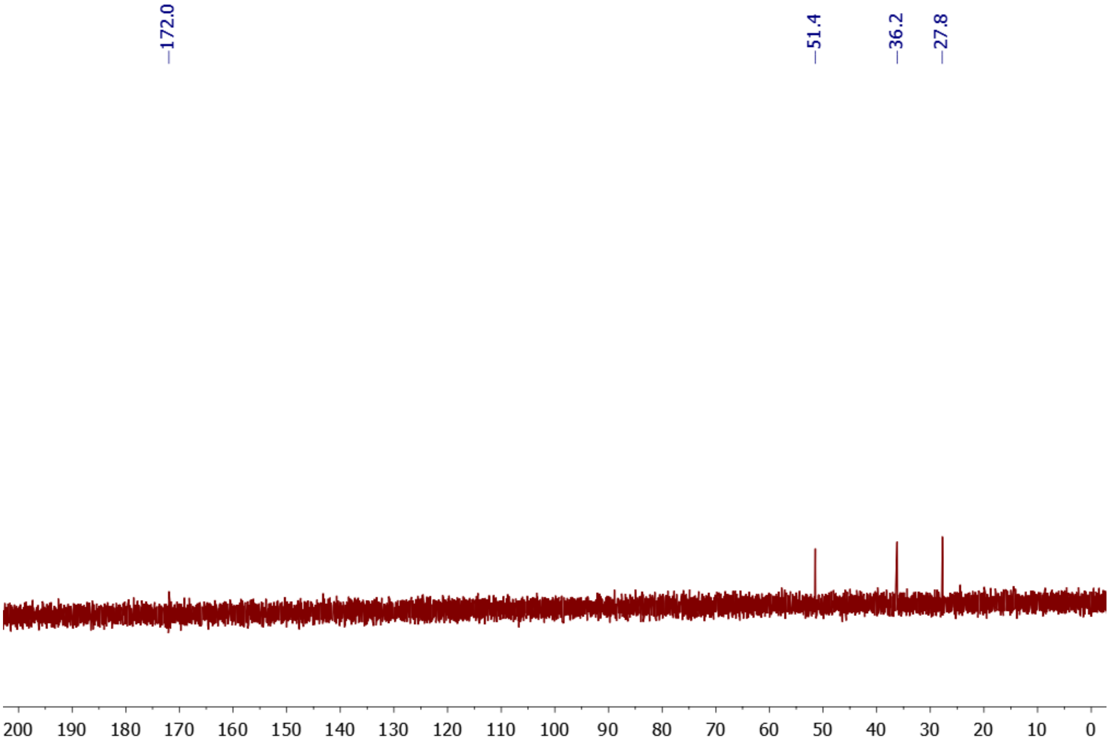

COSY in D_2O **4c**

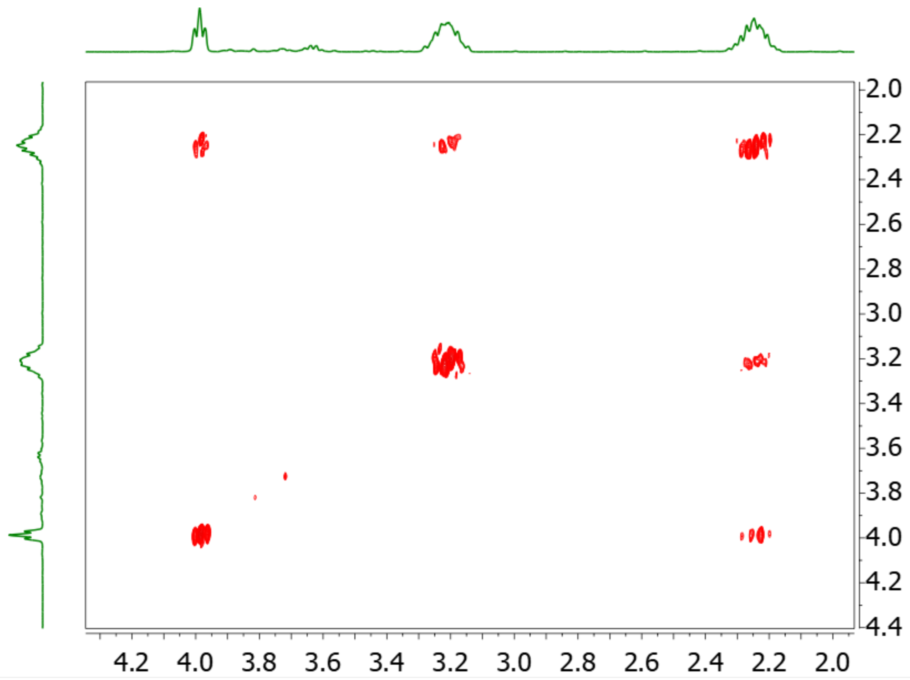

HSQC in D_2O **4c**

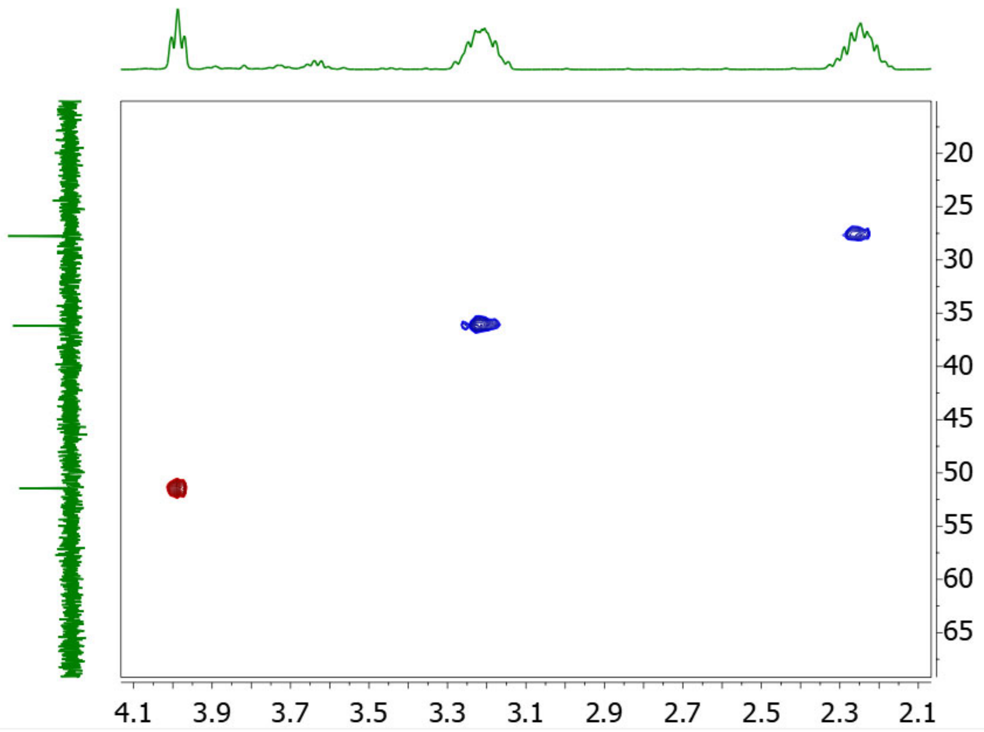

^{1}H NMR in D_2O (400 MHz) **4c-D**

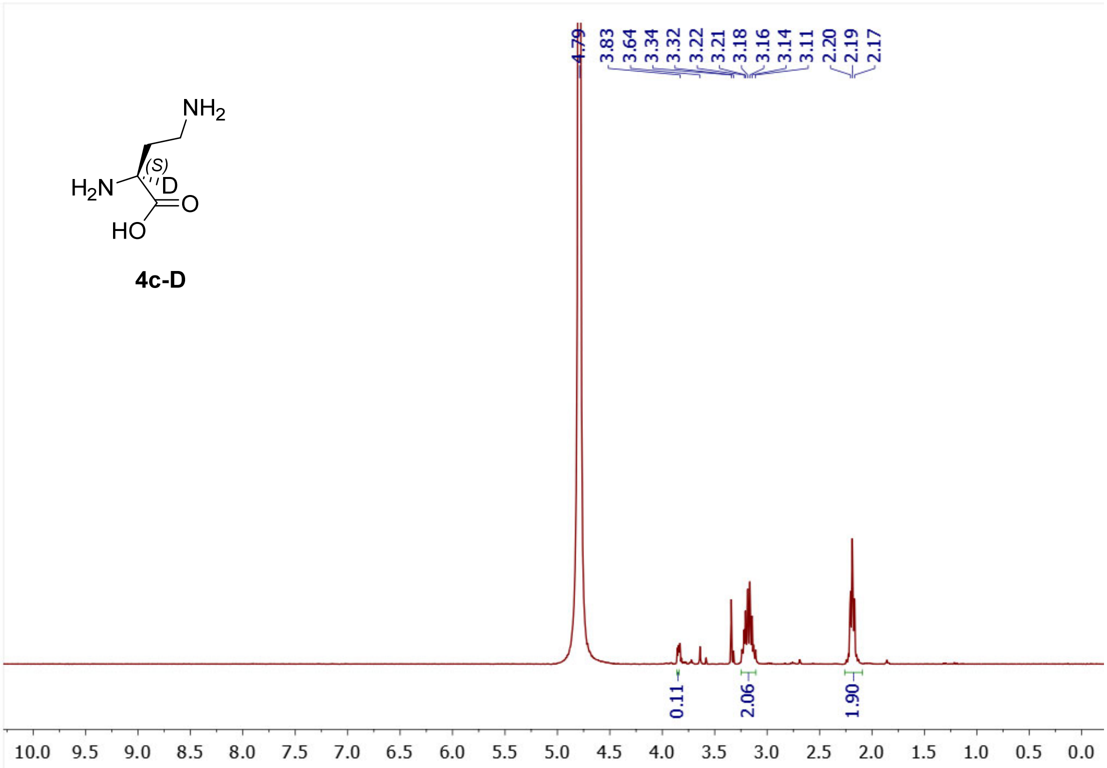

$^{13}C\{^{1}H\}$ NMR in D_2O (100 MHz) **4c-D**

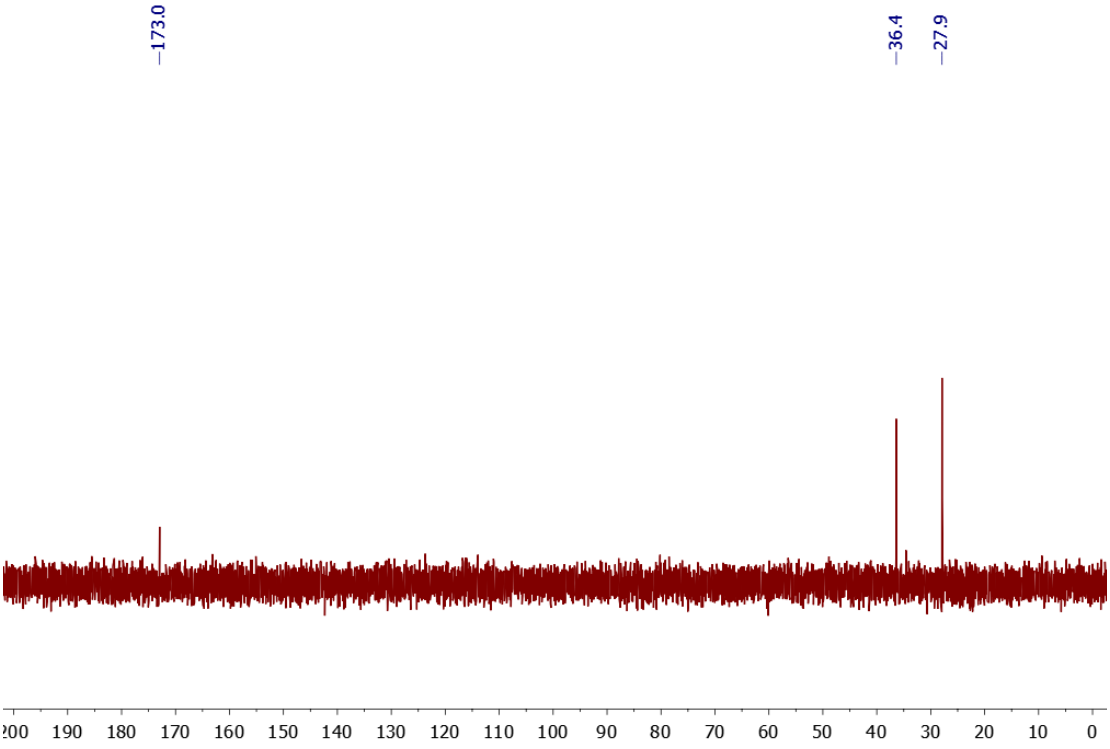

COSY in D_2O **4c-D**

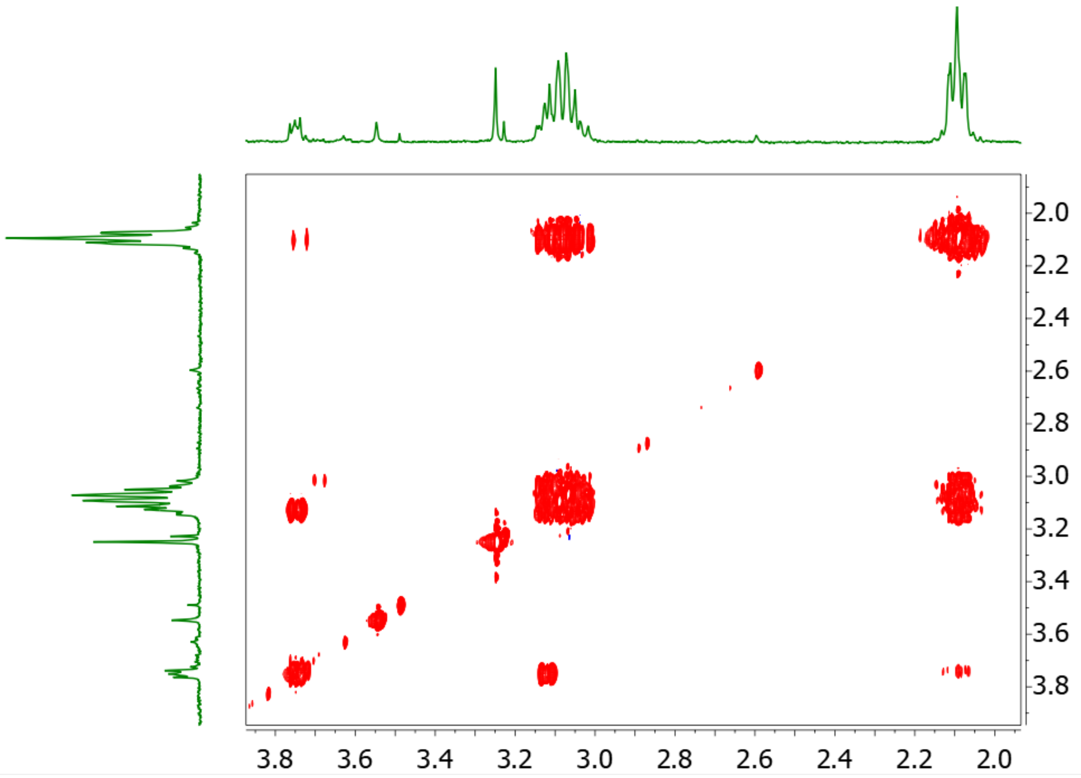

HSQC in D_2O **4c-D**

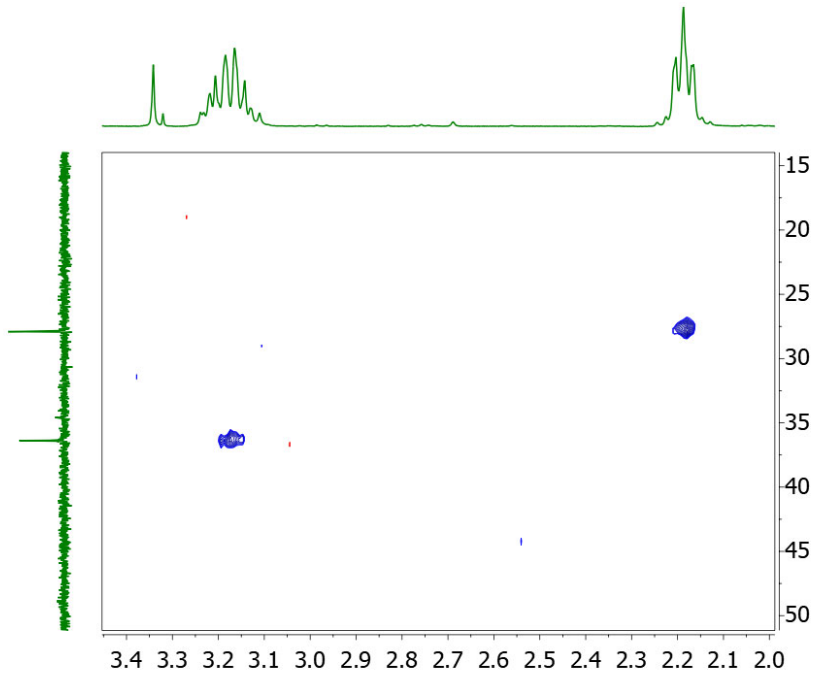

^{1}H NMR in D_2O (400 MHz) **4k**

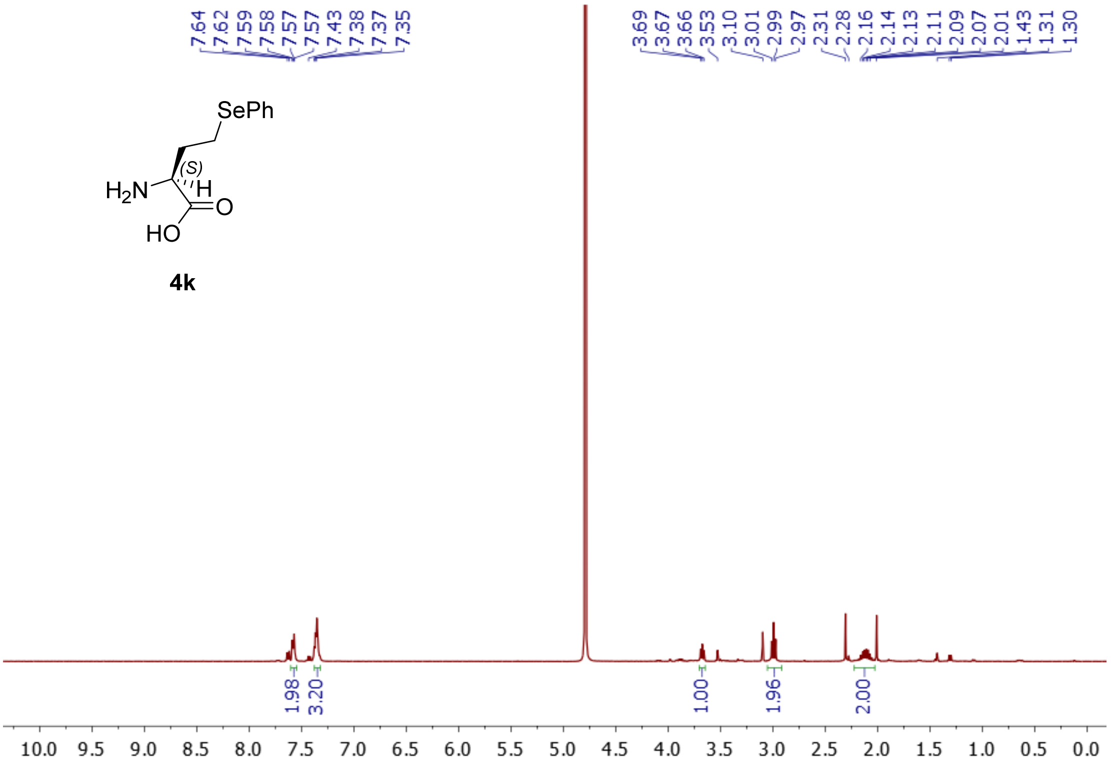

$^{13}C\{^{1}H\}$ NMR in D_2O (100 MHz) **4k**

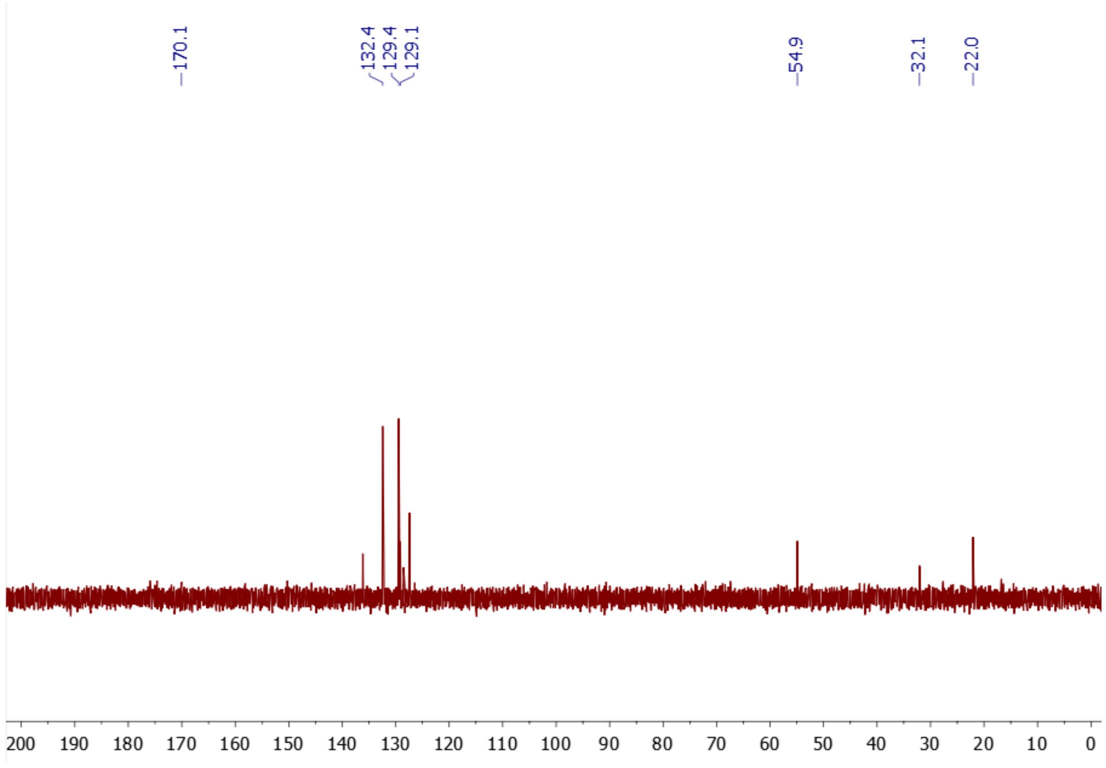

COSY in D_2O **4k**

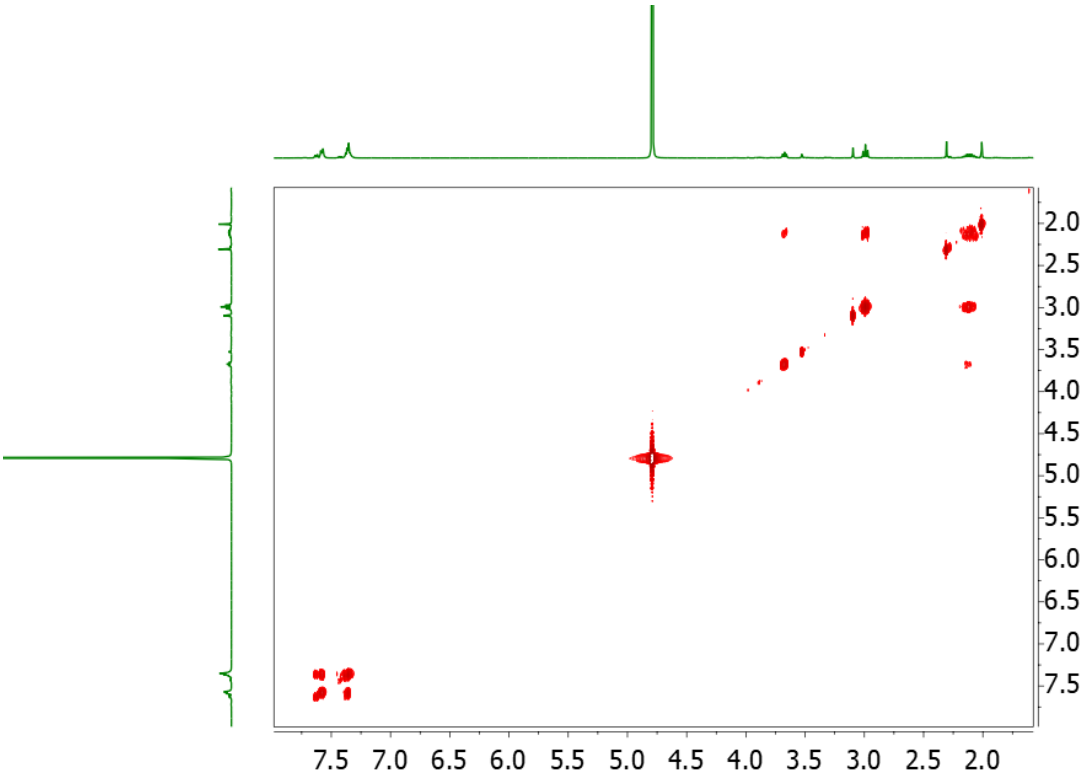

HSQC in D_2O **4k**

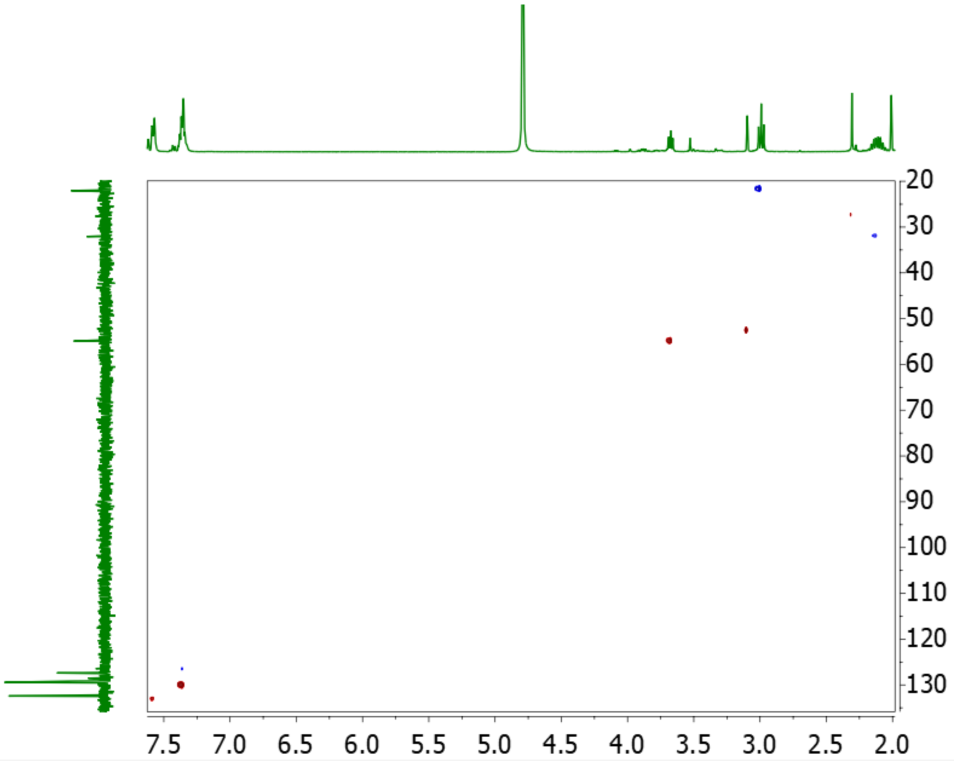

^{1}H NMR in $CDCl_3$ (400 MHz) **5k**

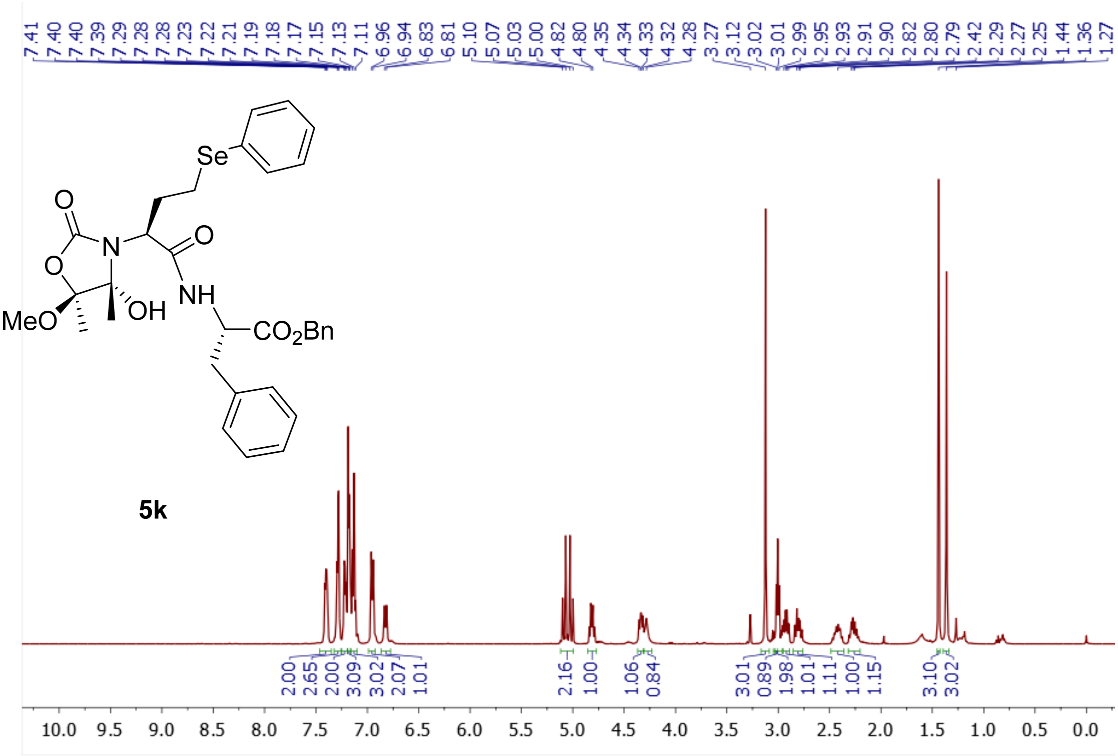

^{13}C{^{1}H} NMR in $CDCl_3$ (100 MHz) **5k**

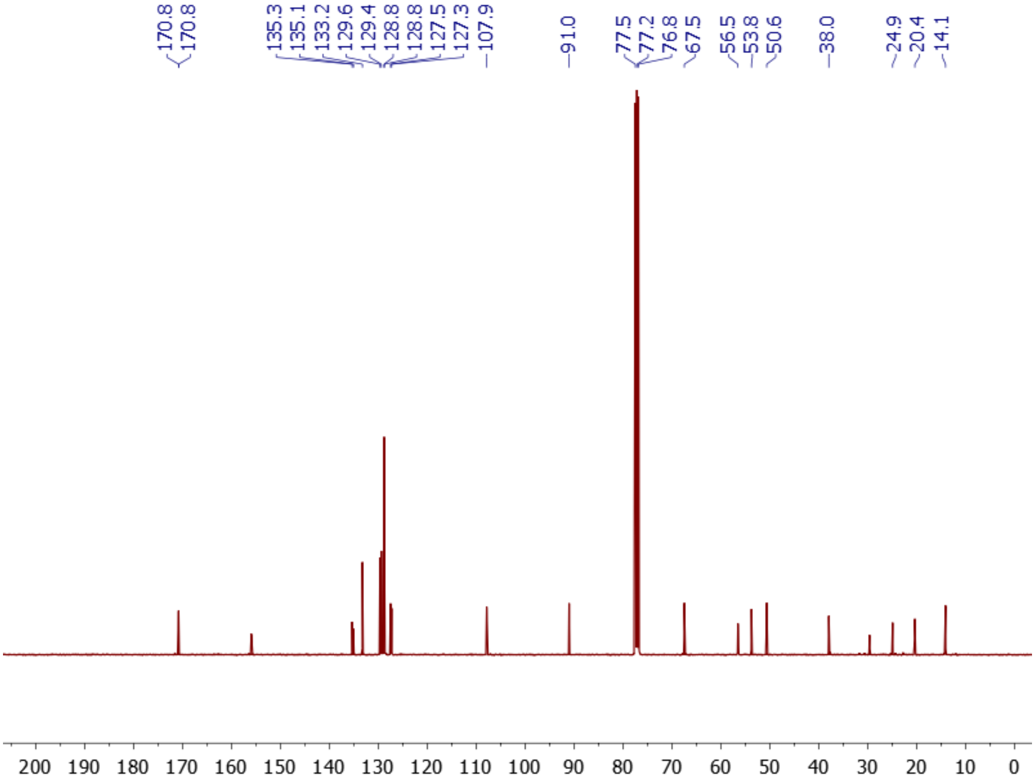

COSY in $CDCl_3$ **5k**

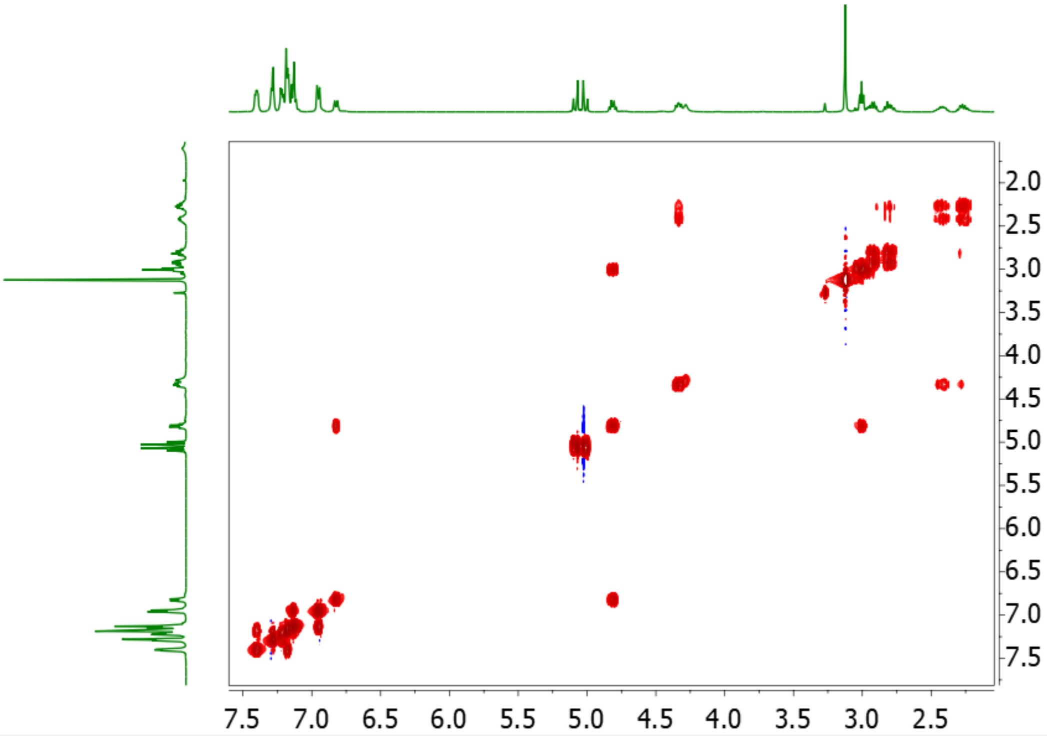

HSQC in $CDCl_3$ **5k**

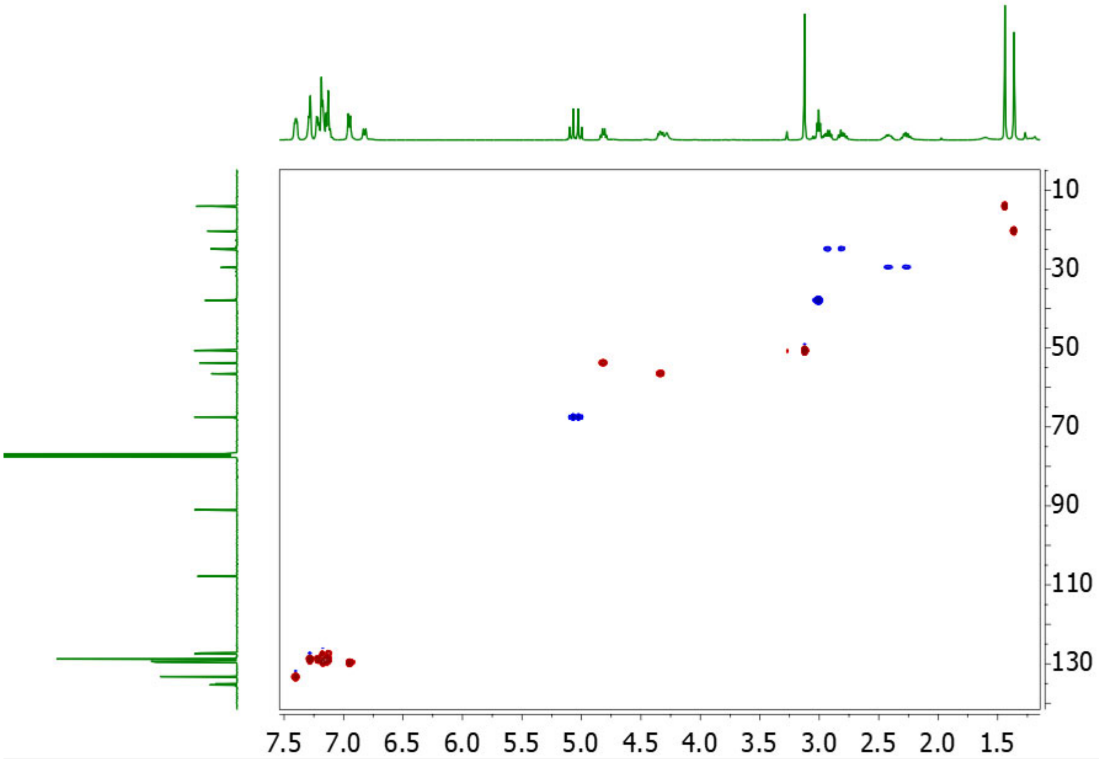

^{1}H NMR in D_2O (400 MHz) **6k**

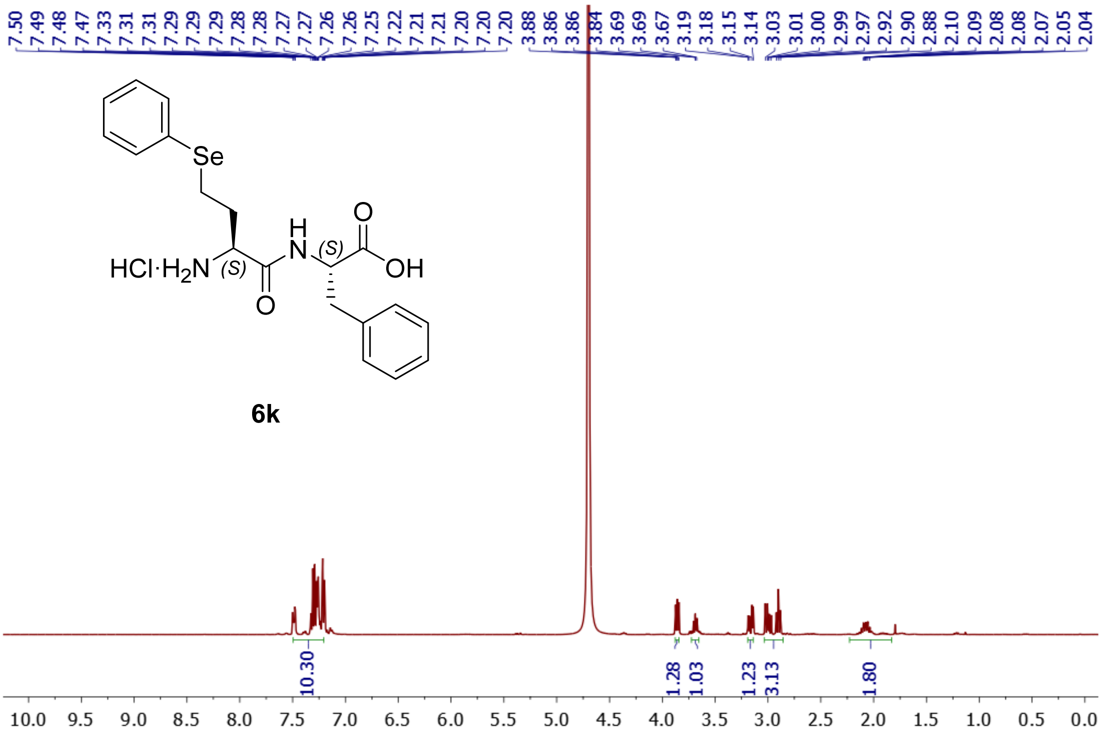

$^{13}C\{^{1}H\}$ NMR in D_2O (100 MHz) **6k**

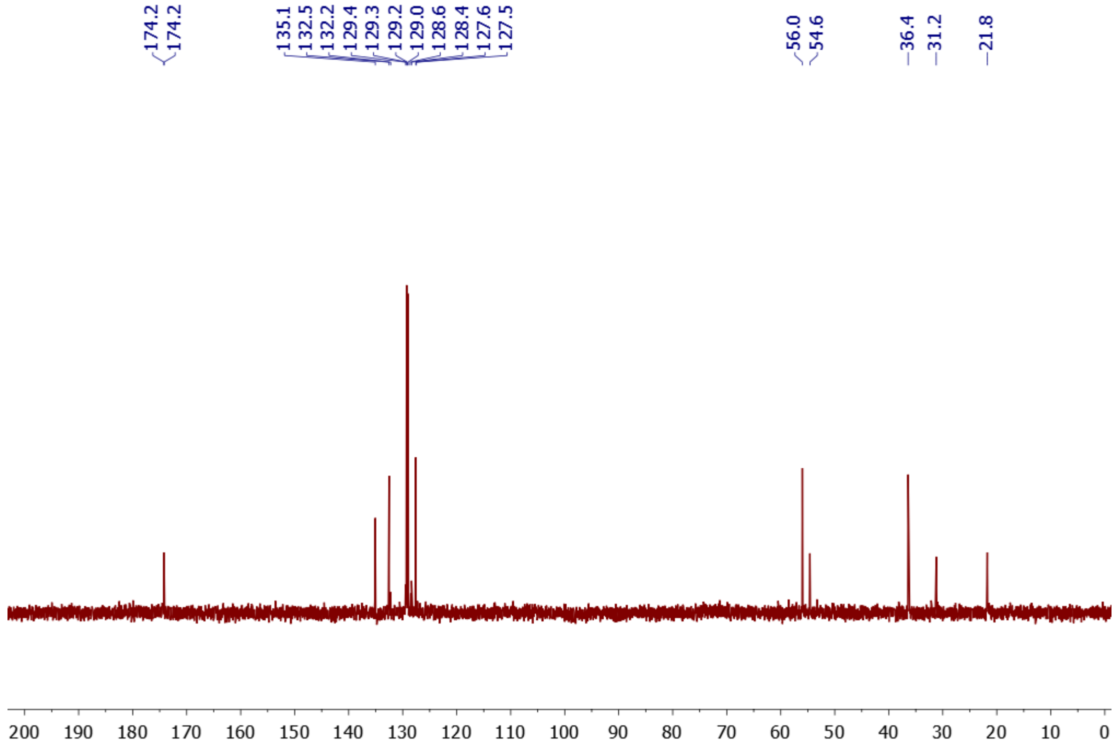

COSY in D_2O **6k**

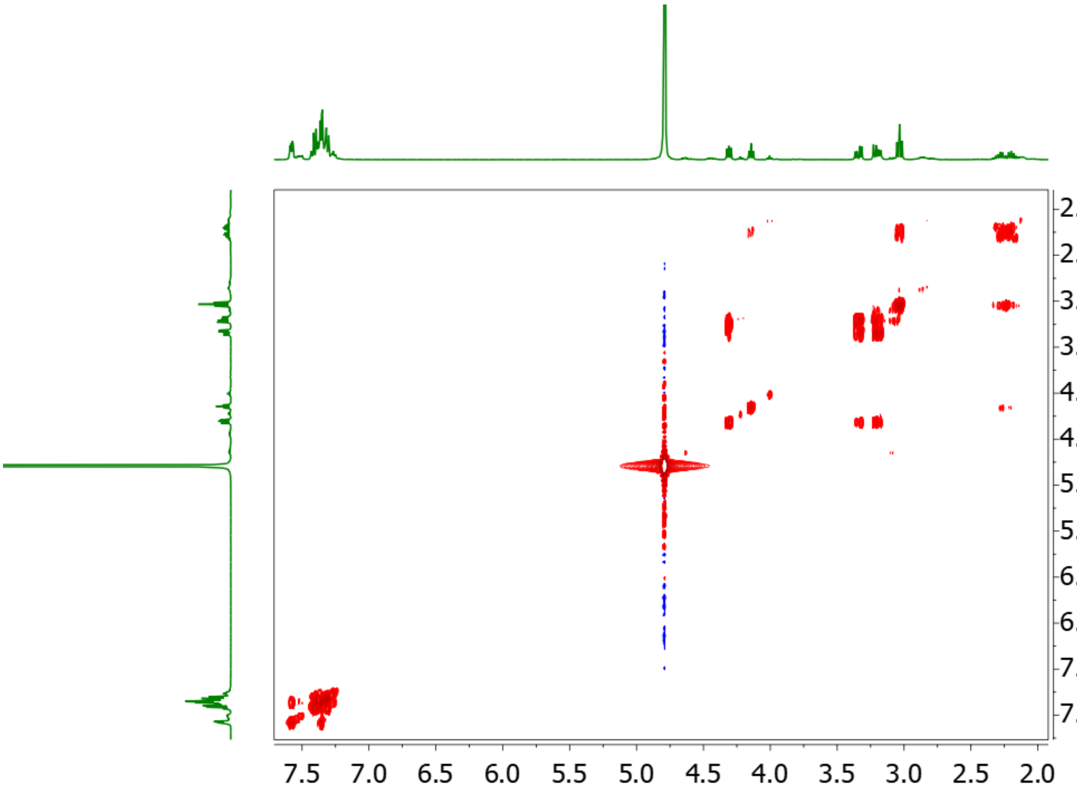

HSQC in D_2O **6k**

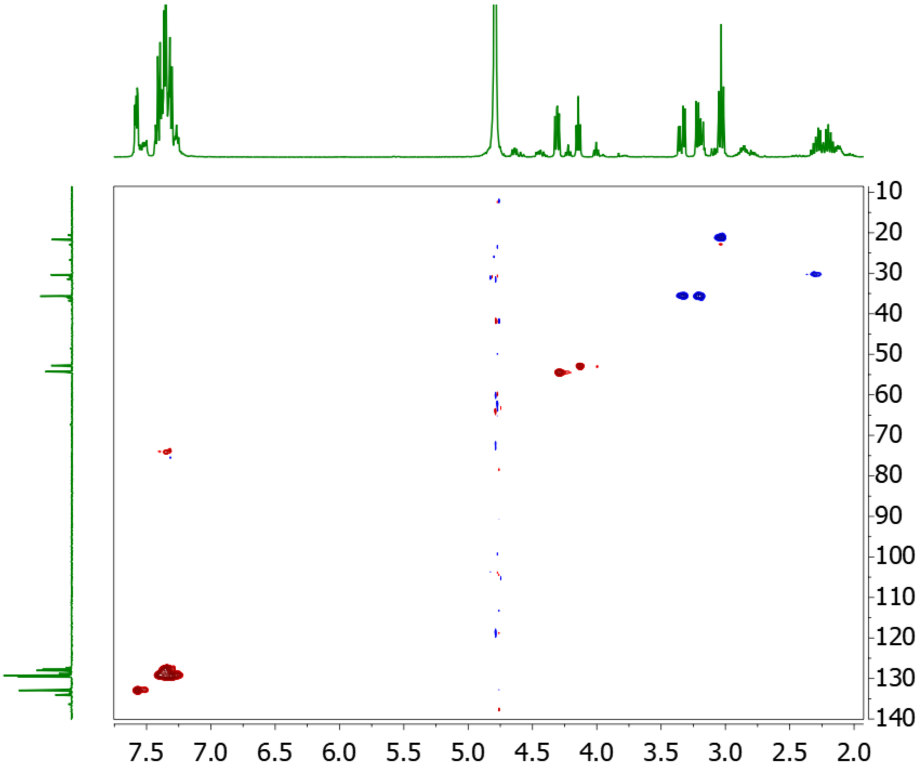

6. Quantum Mechanical calculations

Full geometry optimizations and transition structure (TS) searches were carried out with Gaussian 16[S6] using the M06-2X hybrid functional,[S7] 6-31+G(d,p) basis set with ultrafine integration grids. Bulk solvent effects in either *N*,*N*-dimethylformamide (DMF) or tetrahydrofuran (THF) were considered implicitly through the IEF-PCM polarizable continuum model.[S8] The possibility of different conformations was considered for all structures. All stationary points were characterized by a frequency analysis performed at the same level used in the geometry optimizations from which thermal corrections were obtained at 298.15 K. The quasiharmonic approximation reported by Truhlar et al. was used to replace the harmonic oscillator approximation for the calculation of the vibrational contribution to entropy.[S9] Scaled frequencies were not considered. Mass-weighted intrinsic reaction coordinate (IRC) calculations were carried out by using the Hratchian and Schlegel algorithm[S10] to ensure that the TSs indeed connected the appropriate reactants and products. Gibbs free energies (ΔG) were used for the discussion on the relative stabilities of the considered structures. The lowest energy conformer for each calculated stationary point was considered in the discussion; all the computed structures can be obtained from authors upon request. Electronic energies, entropies, enthalpies, Gibbs free energies, and lowest frequencies of the calculated structures are summarized in Table S4. Cartesian coordinates of the lowest energy structures calculated with PCM(DMF or THF)/M06-2X/6-31+G(d,p) are shown in Tables S5 and S6.

[S6] Gaussian 16, Revision C.01, Frisch, M. J.; Trucks, G. W.; Schlegel, H. B.; Scuseria, G. E.; Robb, M. A.; Cheeseman, J. R.; Scalmani, G.; Barone, V.; Petersson, G. A.; Nakatsuji, H.; Li, X.; Caricato, M.; Marenich, A. V.; Bloino, J.; Janesko, B. G.; Gomperts, R.; Mennucci, B.; Hratchian, H. P.; Ortiz, J. V.; Izmaylov, A. F.; Sonnenberg, J. L.; Williams-Young, D.; Ding, F.; Lipparini, F.; Egidi, F.; Goings, J.; Peng, B.; Petrone, A.; Henderson, T.; Ranasinghe, D.; Zakrzewski, V. G.; Gao, J.; Rega, N.; Zheng, G.; Liang, W.; Hada, M.; Ehara, M.; Toyota, K.; Fukuda, R.; Hasegawa, J.; Ishida, M.; Nakajima, T.; Honda, Y.; Kitao, O.; Nakai, H.; Vreven, T.; Throssell, K.; Montgomery Jr., J. A.; Peralta, J. E.; Ogliaro, F.; Bearpark, M. J.; Heyd, J. J.; Brothers, E. N.; Kudin, K. N.; Staroverov, V. N.; Keith, T. A.; Kobayashi, R.; Normand, J.; Raghavachari, K.; Rendell, A. P.; Burant, J. C.; Iyengar, S. S.; Tomasi, J.; Cossi, M.; Millam, J. M.; Klene, M.; Adamo, C.; Cammi, R.; Ochterski, J. W.; Martin, R. L.; Morokuma, K.; Farkas, O.; Foresman, J. B.; Fox, D. J. Gaussian, Inc., Wallingford CT, 2016.

[S7] Zhao, Y.; Truhlar, D. G. The M06 suite of density functionals for main group thermochemistry, thermochemical kinetics, noncovalent interactions, excited states, and transition elements: two new functionals and systematic testing of four M06-class functionals and 12 other functionals. *Theor. Chem. Acc.* **2008**, *120*, 215–241. DOI: 10.1007/s00214-007-0310-x

[S8] Scalmani, G.; Frisch, M. J. Continuous surface charge polarizable continuum models of solvation. I. General formalism. *J. Chem. Phys.* **2010**, *132*, 114110. DOI: 10.1063/1.3359469

[S9] Ribeiro, R. F.; Marenich, A. V.; Cramer, C. J.; Truhlar, D. G. Use of Solution-Phase Vibrational Frequencies in Continuum Models for the Free Energy of Solvation. *J. Phys. Chem. B* **2011**, *115*, 14556–14562. DOI: 10.1021/jp205508z

[S10] Hratchian, H. P.; Schlegel, H. B. Following Reaction Pathways Using a Damped Classical Trajectory Algorithm. *J. Phys. Chem. A* **2002**, *106*, 165–169. DOI: 10.1063/1.1724823

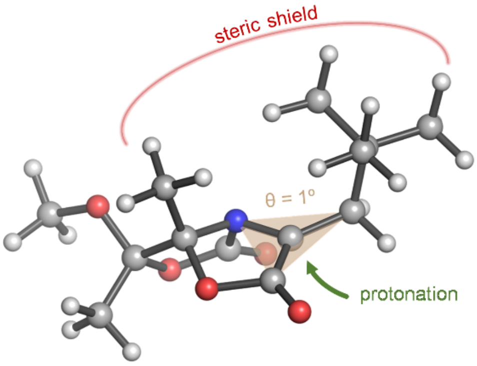

3g$^-$

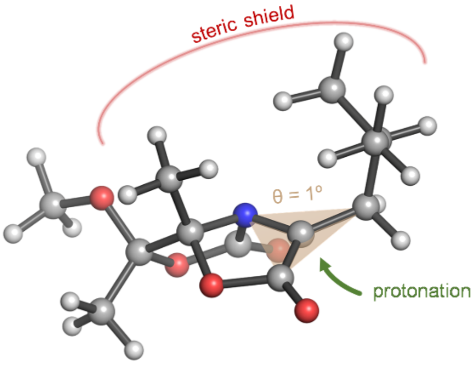

3h$^-$

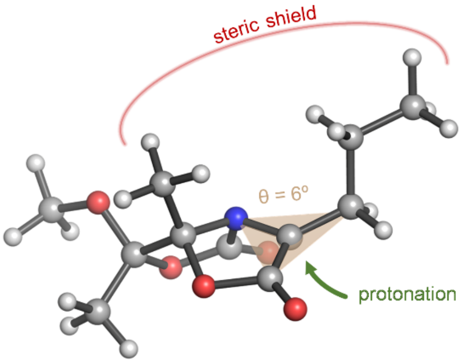

3Et$^-$

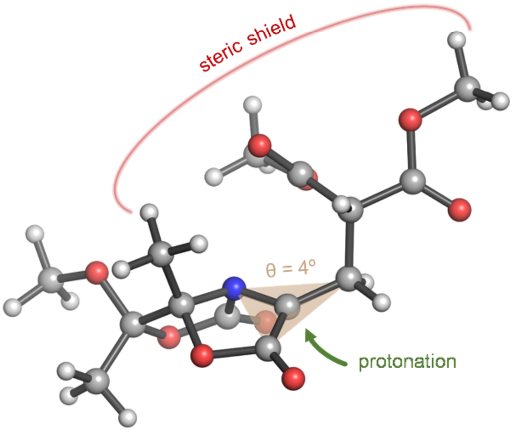

3a$^-$

Figure S4. Lowest energy structure for enolate intermediates **3**$^-$ calculated with PCM(DMF)/M06-2X/6-31+G(d,p). θ represents the out-of-plane (pyramidalization) angle of Cβ with respect the plane defined by C2, C3 and N4. Angles close to 0° correspond to planarity (i.e. negligible pyramidalization at Cα.). Of note. Pyramidalization at Cα increases slightly when the substituent at Cβ is smaller.

Rotation along the Cα–Cβ bond in enolates **3**$^-$ reduces the steric hindrance at the convex face of the bicyclic scaffold (**Figure S5**), which might facilitate protonation by such face. However, this rotation comes with an energetic penalty associated with either steric repulsion between the alkyl substituent at Cβ and the carbamate group (**Figure S5**, green inset) and/or with torsional strain on the bicyclic structure (**Figure S5**, blue inset). Additionally, all enolates but **3a**$^-$_*conf2* display higher pyramidalization angles at Cα than their respective minimum energy conformers, suggesting that despite the convex face is less hindered, the electron density is still more accessible by the concave face.

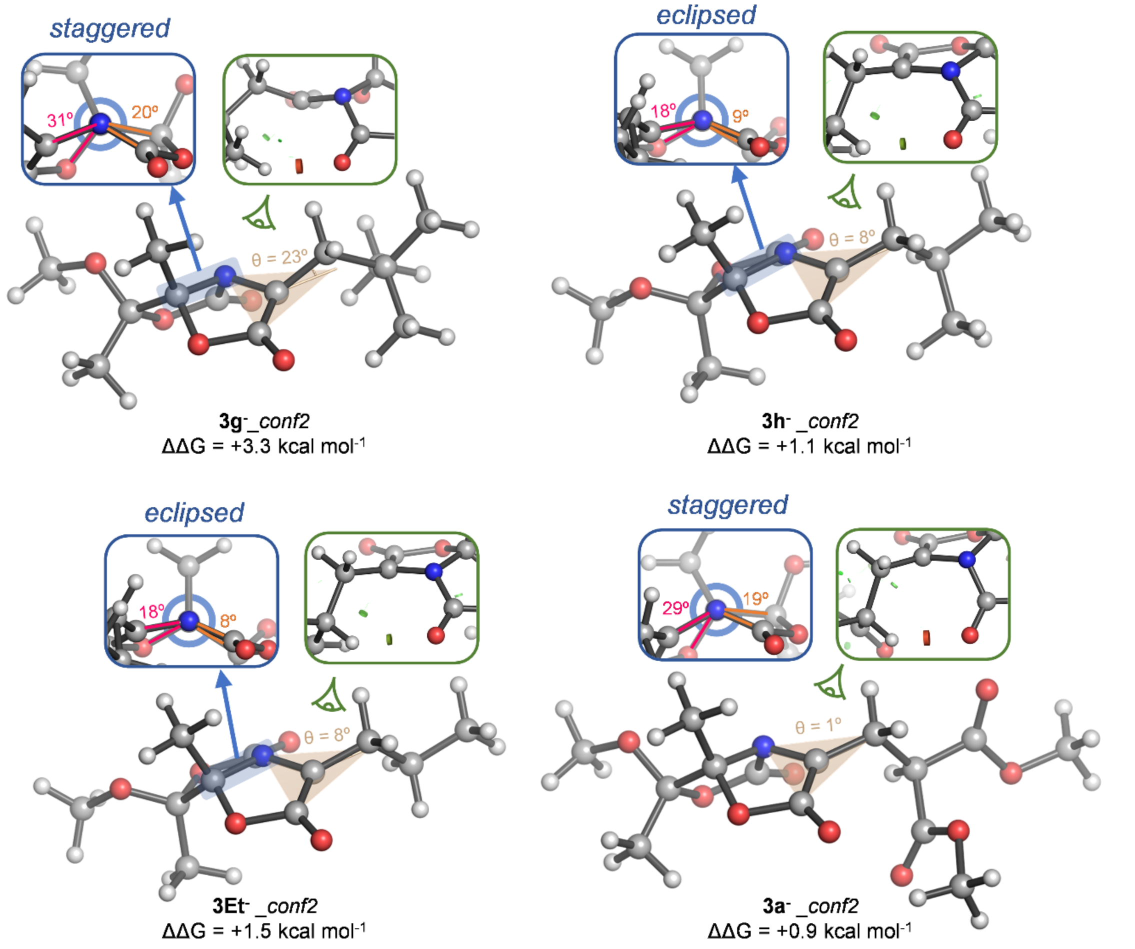

Figure S5. Lowest energy structure for the rotated conformer (*conf2*) of enolate intermediates **3**$^-$ calculated with PCM(DMF)/M06-2X/6-31+G(d,p). Gibbs free energies (ΔΔG) are relative to those of the lowest energy conformers shown in Figure S4. θ represents the out-of-plane (pyramidalization) angle of Cβ with respect the plane defined by C2, C3 and N4. Angles close to 0° correspond to planarity (i.e. negligible pyramidalization at Cα). Blue inset: Newman projections from atoms N4 to C7a. Torsional strain is represented through the dihedral angles highlighted in magenta and orange. Dihedral angles closer to 60° correspond to more staggered conformations. Green inset: Qualitative representation of repulsive interactions (pairwise overlap of atomic van der Waals radii) within the molecule. Large red and small green disks indicate significant and slight overlap of atomic van der Waals radii, respectively.

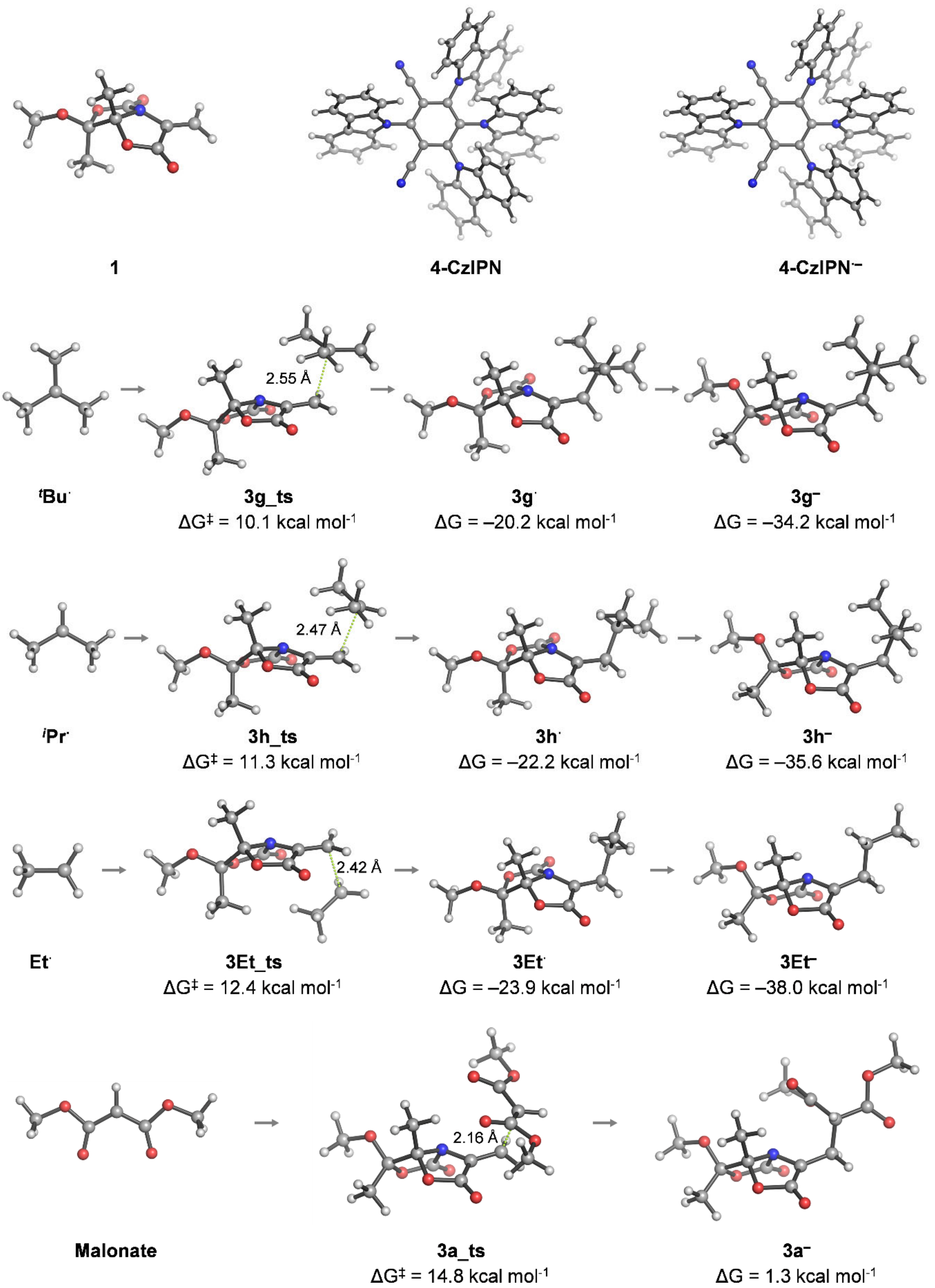

Figure S6. Geometries for the reactants, transition structures, radical intermediates and enolates for the Michael addition reaction calculated with PCM(DMF)/M06-2X/6-31+G(d,p).

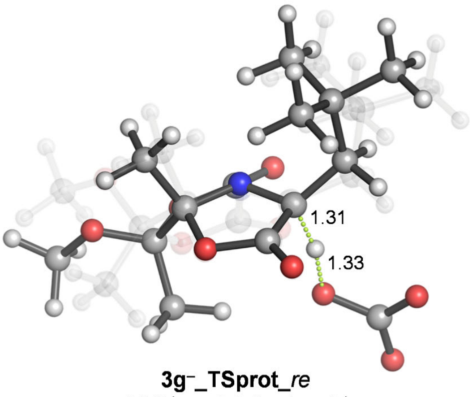

3g⁻_TSprot_*re*
ΔΔG‡ = +0.0 kcal mol-1

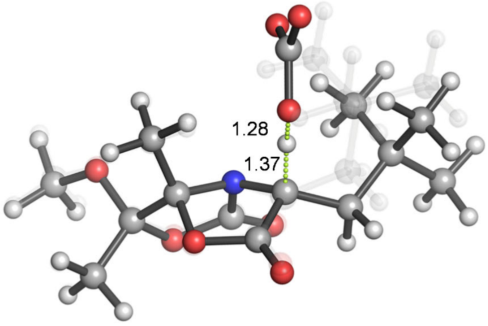

3g⁻_TSprot_*si*
ΔΔG‡ = +2.6 kcal mol-1

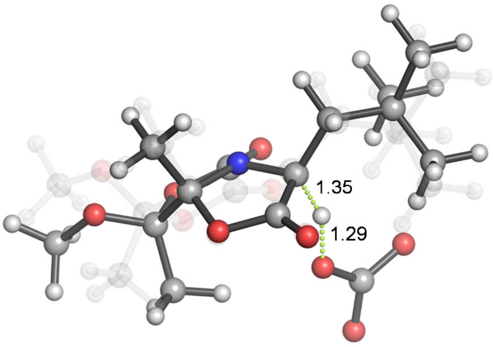

3g⁻_*conf2*_TSprot_*re*
ΔΔG‡ = +3.2 kcal mol-1

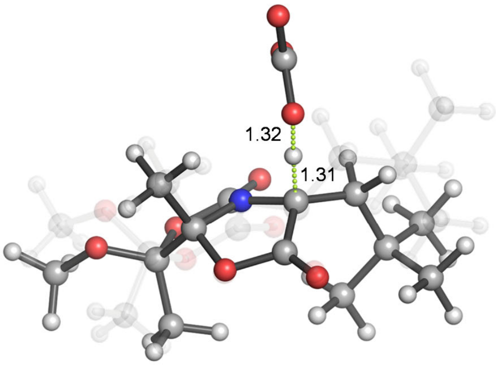

3g⁻_*conf2*_TSprot_*si*
ΔΔG‡ = +3.8 kcal mol-1

Figure S7. Lowest energy transition structures for the protonation of enolate **3g⁻** (**3g⁻_TSprot**) and its rotamer **3g⁻**_*conf2* (**3g⁻**_*conf2*_**TSprot**) by the *re* and *si* faces with hydrogencarbonate anion (**HCO_3^-**) as the proton source calculated with PCM(DMF)/M062-2X/6-31+G(d,p). Gibbs free energies of activation (ΔΔG‡) are relative to that calculated for **3g⁻_TSprot**_*re*. The lowest energy geometry for enolate **3g⁻** (top) and its rotamer **3g⁻**_*conf2* (bottom) are shown with transparency as a reference of the conformational changes ocurring upon protonation.

Table S4. Energies, entropies, and lowest frequencies of the lowest energy calculated structures.[a]

Structure	E_{elec} (Hartree)	E_{elec}+ZPE (Hartree)	H (Hartree)	S (cal mol^{-1} K^{-1})	G (Hartree)	Lowest freq. (cm^{-1})	# imag. freq.
**1**	–780.199349	–779.996437	–779.981910	114.1	–780.035296	45.8	0
**1**_*THF*	–780.197195	–779.994157	–779.979642	114.0	–780.033008	46.0	0
**4-CzIPN**	–2480.973992	–2480.243810	–2480.197825	263.7	–2480.310843	16.5	0
**4-CzIPN$^{•–}$**	–2481.090180	–2480.363775	–2480.317385	268.3	–2480.431704	15.8	0
**^{t}Bu$^•$**	–157.712799	–157.595485	–157.588268	76.6	–157.624684	146.8	0
**3g_ts**	–937.918810	–937.597373	–937.575827	150.3	–937.643964	–113.9	1
**3g$^•$**	–937.974036	–937.647153	–937.626763	142.5	–937.692105	38.8	0
**3g$^–$**	–938.116837	–937.791130	–937.770870	140.0	–937.835294	44.3	0
**3g$^–$**_*conf2*	–938.111265	–937.785735	–937.765477	139.6	–937.830021	38.0	0
**^{i}Pr$^•$**	–118.411388	–118.322831	–118.316761	69.7	–118.349880	121.4	0
**3h_ts**	–898.615421	–898.322010	–898.301992	143.1	–898.367121	–225.6	1
**3h$^•$**	–898.674804	–898.376099	–898.356837	138.7	–898.420314	36.4	0
**3h$^–$**	–898.817017	–898.519410	–898.500272	137.1	–898.562729	20.6	0
**3h$^–$**_*conf2*	–898.814960	–898.517617	–898.498403	137.3	–898.560958	25.9	0
**Et$^•$**	–79.111065	–79.051423	–79.046519	61.0	–79.075493	137.0	0
**3Et_ts**	–859.312742	–859.047435	–859.028992	134.6	–859.091027	–314.8	1
**3Et$^•$**	–859.376589	–859.105847	–859.087996	131.9	–859.148893	40.9	0
**3Et$^–$**	–859.518952	–859.249743	–859.231712	132.7	–859.292212	30.7	0
**3Et$^–$**_*conf2*	–859.516405	–859.247374	–859.229308	132.9	–859.289868	25.4	0
**Malonate** [b]	–495.596892	–495.475581	–495.464910	97.4	–495.510534	63.4	0
**3a_ts** [b]	–1275.796044	–1275.469892	–1275.445017	165.5	–1275.519891	–342.1	1
**3a$^–$** [b]	–1275.820478	–1275.491542	–1275.466590	166.2	–1275.541484	29.0	0
**3a$^–$**_*conf2* [b]	–1275.819005	–1275.489969	–1275.465078	166.1	–1275.539786	23.6	0
**$HCO_3^–$**	–264.473326	–264.446619	–264.442184	63.5	–264.472357	511.7	0
**3g$^–$_TSprot**_*re*	–1202.578413	–1202.228561	–1202.203903	163.0	–1202.277921	–1255.1	1
**3g$^–$**_*conf2*_**TSprot**_*re*	–1202.573363	–1202.223585	–1202.199006	162.8	–1202.272795	–1394.0	1
**3g$^–$_TSprot**_*si*	–1202.575668	–1202.224905	–1202.200731	158.6	–1202.273722	–1412.1	1
**3g$^–$_conf2_TSprot**_*si*	–1202.572544	–1202.222530	–1202.197814	166.7	–1202.271815	–1275.3	1

[a]Energy values calculated at the PCM(DMF)/M06-2X/6-31+G(d,p) level unless otherwise stated. 1 Hartree = 627.51 kcal mol^{-1}. Thermal corrections at 298.15 K.
[b]Energy values calculated at the PCM(THF)/M06-2X/6-31+G(d,p) level.

Table S5. Cartesian coordinates of the lowest energy structures calculated with PCM(DMF)/M06-2X/6-31+G(d,p)

Structure **1**

C	1.973975	-1.129703	-0.298211
C	-0.078780	-0.575584	0.573614
C	-1.330585	-0.129682	-0.239589
C	0.018753	1.720981	0.080593
O	-2.456012	-0.562208	0.411789
C	-3.706979	-0.271526	-0.216093
H	-3.769718	0.786993	-0.483083
H	-4.469924	-0.506612	0.523926
H	-3.854016	-0.895072	-1.101602
C	-1.258125	-0.478104	-1.713313
H	-2.039300	0.054133	-2.257924
H	-1.378469	-1.555046	-1.846752
H	-0.291763	-0.178352	-2.126142
O	-1.254116	1.334438	-0.126820
O	0.434923	2.837393	-0.093002
N	0.745753	0.629382	0.530960
C	2.048429	0.305481	0.120334
C	3.127818	1.077253	0.033990
H	3.109036	2.115178	0.341627
H	4.038893	0.651917	-0.370413
O	2.838949	-1.826560	-0.751301
O	0.700954	-1.565352	-0.104617
C	-0.361734	-1.044724	1.983032
H	-0.928182	-1.976378	1.959082
H	-0.941342	-0.288327	2.514642
H	0.590299	-1.202155	2.495397

Structure **4-CzIPN**

C	0.179202	-1.165789	-0.358142
C	-0.533120	-0.000008	-0.000264
C	0.181193	1.164676	0.357232
C	1.588473	1.144848	0.372026
C	2.299499	-0.002112	-0.001178
C	1.586511	-1.148018	-0.373807
C	2.313030	2.275149	0.882547
N	2.906210	3.174208	1.305341
C	2.309159	-2.279485	-0.884456
N	2.900838	-3.179515	-1.307287
N	3.700505	-0.003275	-0.001708
C	4.517143	0.791956	-0.816446
C	4.516351	-0.797623	0.814719
C	4.150255	1.705959	-1.803141
C	5.864728	0.504511	-0.519774
C	5.864228	-0.509136	0.520335
C	4.148516	-1.711972	1.800737
C	5.176134	2.355014	-2.482883
H	3.109952	1.900029	-2.044363
C	6.878348	1.166693	-1.218822
C	6.877169	-1.170562	1.221069
H	3.107973	-1.907021	2.040117
C	5.173753	-2.360160	2.482286
C	6.525868	2.093315	-2.193902
H	4.924495	3.074709	-3.254926
H	7.921920	0.956508	-1.005241
C	6.523764	-2.097418	2.195595
H	7.920935	-0.959603	1.009210
H	4.921353	-3.080052	3.253896
H	7.299985	2.617288	-2.744279
H	7.297359	-2.620774	2.747291
N	-0.488677	-2.335242	-0.742061
C	-0.415871	-3.569067	-0.070602
C	-1.486511	-2.425105	-1.724928
C	0.384449	-3.940573	1.008537
C	-1.378566	-4.438564	-0.619463
C	-2.060116	-3.710060	-1.674698
C	-1.863125	-1.481906	-2.678434
C	0.215335	-5.223686	1.520393
H	1.105814	-3.260498	1.450513
C	-1.531003	-5.723166	-0.091146
C	-3.076579	-4.046971	-2.573401
H	-1.381936	-0.511799	-2.742116
C	-2.882087	-1.833554	-3.557787
C	-0.727897	-6.110113	0.976102
H	0.826638	-5.540492	2.358993
H	-2.271754	-6.402063	-0.502498
C	-3.490334	-3.098829	-3.502972
H	-3.529553	-5.033374	-2.548297
H	-3.205308	-1.114761	-4.304214
H	-0.833240	-7.103538	1.398799
H	-4.281499	-3.341458	-4.204444
N	-1.935548	0.001129	0.000013
C	-2.755394	-0.857884	0.743829
C	-2.754246	0.861540	-0.743455
C	-2.395012	-1.830676	1.674773
C	-4.103081	-0.548620	0.472260
C	-4.102338	0.554492	-0.471394
C	-2.392602	1.833762	-1.674501
C	-3.420930	-2.533537	2.298674
H	-1.357467	-2.031192	1.921345
C	-5.118911	-1.263588	1.113404
C	-5.117226	1.271126	-1.112165
H	-1.354826	2.032629	-1.921434
C	-3.417592	2.538313	-2.298021
C	-4.770159	-2.262255	2.016463
H	-3.167861	-3.302554	3.021822
H	-6.161743	-1.036569	0.913404
C	-4.767167	2.269229	-2.015344
H	-6.160354	1.045796	-0.911798
H	-3.163506	3.306941	-3.021226
H	-5.545795	-2.829562	2.519889
H	-5.542055	2.837797	-2.518498

N	-0.484785	2.335055	0.741635
C	-1.481823	2.426220	1.725187
C	-0.410729	3.568825	0.070228
C	-1.859164	1.483421	2.678794
C	-2.053640	3.711991	1.675505
C	-1.371804	4.439630	0.619859
C	0.389346	3.939290	-1.009452
C	-2.876996	1.836409	3.558916
H	-1.379357	0.512605	2.741993
C	-3.068998	4.050237	2.574966
C	-1.522813	5.724485	0.091747
H	1.109478	3.258238	-1.451934
C	0.221660	5.222672	-1.521097
C	-3.483452	3.102573	3.504709
H	-3.200727	1.117981	4.305473
H	-3.520590	5.037285	2.550293
C	-0.719941	6.110379	-0.976057
H	-2.262315	6.404388	0.503682
H	0.832809	5.538689	-2.360106
H	-4.273760	3.346254	4.206781
H	-0.824201	7.103978	-1.398613

Structure **4-CzIPN$^{\bullet-}$**

C	0.139752	-1.175485	-0.330861
C	-0.547728	0.000202	0.000157
C	0.141283	1.174977	0.330843
C	1.594674	1.154614	0.381133
C	2.281118	-0.001559	-0.000399
C	1.593125	-1.156817	-0.381717
C	2.314823	2.262814	0.891923
N	2.901896	3.179959	1.307966
C	2.311767	-2.266003	-0.892572
N	2.897531	-3.183975	-1.308603
N	3.694801	-0.002449	-0.000520
C	4.507071	0.760976	-0.836598
C	4.506196	-0.766278	0.836062
C	4.138131	1.650295	-1.847645
C	5.858930	0.485130	-0.536669
C	5.858371	-0.491270	0.536767
C	4.136241	-1.655269	1.847029
C	5.160881	2.275694	-2.552164
H	3.094391	1.843924	-2.074058
C	6.869775	1.126699	-1.260300
C	6.868483	-1.133391	1.260931
H	3.092283	-1.848261	2.072967
C	5.158280	-2.281201	2.552108
C	6.513610	2.021346	-2.263089
H	4.907047	2.974429	-3.342969
H	7.914675	0.925642	-1.042947
C	6.511299	-2.027716	2.263646
H	7.913606	-0.932985	1.044055
H	4.903641	-2.979674	3.342886
H	7.285277	2.528293	-2.832870
H	7.282386	-2.535058	2.833860
N	-0.536719	-2.362431	-0.685707
C	-0.475109	-3.565020	0.015978
C	-1.454611	-2.511266	-1.723113
C	0.292535	-3.884378	1.138530
C	-1.370474	-4.490435	-0.562948
C	-2.005496	-3.811303	-1.676136
C	-1.816475	-1.593681	-2.711051
C	0.148979	-5.160050	1.672645
H	0.969814	-3.159907	1.581179
C	-1.497720	-5.768498	-0.008787
C	-2.963227	-4.191311	-2.622958
H	-1.367552	-0.606799	-2.751939
C	-2.771609	-1.991600	-3.639443
C	-0.735132	-6.096846	1.106774
H	0.732293	-5.436913	2.545280
H	-2.185453	-6.489904	-0.440635
C	-3.347085	-3.274959	-3.595812
H	-3.396737	-5.186976	-2.598635
H	-3.076097	-1.295751	-4.415468
H	-0.821813	-7.083887	1.548943
H	-4.091792	-3.552097	-4.334749
N	-1.965095	0.001037	0.000560
C	-2.780423	-0.816623	0.780879
C	-2.780111	0.818964	-0.779786
C	-2.418726	-1.751613	1.752467
C	-4.132796	-0.524629	0.499439
C	-4.132594	0.527304	-0.498498
C	-2.418108	1.754253	-1.750978
C	-3.441293	-2.423080	2.412995
H	-1.377865	-1.943898	1.990070
C	-5.146010	-1.210065	1.178457
C	-5.145572	1.213248	-1.177346
H	-1.377202	1.946309	-1.988549
C	-3.440452	2.426260	-2.411311
C	-4.793815	-2.163764	2.126895
H	-3.185750	-3.159894	3.168597
H	-6.190155	-0.994841	0.970687
C	-4.793064	2.167191	-2.125428
H	-6.189788	0.998241	-0.969704
H	-3.184641	3.163305	-3.166596
H	-5.567023	-2.706855	2.660307
H	-5.566091	2.710702	-2.658673
N	-0.533515	2.362951	0.685380
C	-1.450986	2.513697	1.722859
C	-0.469678	3.565303	-0.016520
C	-1.814378	1.597004	2.711066
C	-1.999367	3.814793	1.675746
C	-1.363180	4.492524	0.562370
C	0.298211	3.882857	-1.139421
C	-2.768490	1.996968	3.639627
H	-1.367355	0.609268	2.752044
C	-2.956099	4.196843	2.622763
C	-1.488158	5.770678	0.007895
H	0.973916	3.156944	-1.582114

C	0.156956	5.158675	-1.673803
C	-3.341485	3.281433	3.595893
H	-3.074125	1.301870	4.415874
H	-3.387687	5.193341	2.598350
C	-0.725205	6.097298	-1.107925
H	-2.174466	6.493472	0.439688
H	0.740531	5.434199	-2.546688
H	-4.085456	3.560166	4.334970
H	-0.810112	7.084392	-1.550321

Structure **^{t}Bu$^{\bullet}$**

C	0.000007	0.000001	-0.194823
C	-1.147350	0.934623	0.019942
H	-2.073732	0.543109	-0.413289
H	-0.950946	1.921165	-0.412840
H	-1.339456	1.090804	1.096100
C	1.383095	0.526307	0.019951
H	1.507215	1.524319	-0.413278
H	2.139265	-0.137025	-0.412885
H	1.614372	0.614569	1.096121
C	-0.235744	-1.460936	0.019952
H	-0.275254	-1.705339	1.096101
H	0.566643	-2.067409	-0.413034
H	-1.188155	-1.784161	-0.413127

Structure **3g_ts**

O	0.748270	1.768370	-0.016380
O	2.174796	-1.400762	-0.376948
O	0.379970	-2.427903	-1.292196
N	0.188127	-0.445165	-0.113170
O	3.380158	-0.029301	1.036105
C	2.487046	-0.014710	-0.004996
C	0.971862	0.528044	2.026109
H	1.222003	-0.448380	2.444506
H	1.641833	1.279173	2.446406
H	-0.062601	0.777175	2.276453
C	-0.854034	0.269743	-0.739227
C	0.862712	-1.517251	-0.666448
C	1.110756	0.493486	0.519786
C	4.713258	-0.438237	0.723443
H	5.212252	-0.576958	1.681018
H	4.709939	-1.382696	0.172222
H	5.234551	0.332521	0.149804
C	2.961753	0.697523	-1.256594
H	3.271769	1.715191	-1.011042
H	3.795155	0.150724	-1.699978
H	2.154217	0.743753	-1.991366
C	-0.450966	1.691029	-0.668820
O	-1.009130	2.666961	-1.100084
C	-1.974601	-0.202743	-1.307419
H	-2.621062	0.491261	-1.833406
H	-2.151599	-1.267347	-1.399167
C	-3.749981	-0.361023	0.513197
C	-3.760444	1.066114	0.953212
H	-4.116755	1.733033	0.161520
H	-2.760938	1.394291	1.263011
H	-4.423719	1.202764	1.822813
C	-3.032564	-1.339930	1.386027
H	-2.043445	-0.964260	1.673319
H	-2.906165	-2.308852	0.891641
H	-3.593455	-1.518318	2.317992
C	-4.893325	-0.854545	-0.313331
H	-5.785707	-1.018540	0.313060
H	-4.661109	-1.810949	-0.793824
H	-5.173401	-0.131350	-1.086397

Structure **3g$^{\bullet}$**

O	0.807930	1.852124	0.016179
O	1.953010	-1.465488	-0.271869
O	-0.007561	-2.474809	-0.776294
N	0.095350	-0.307007	0.060978
O	3.451586	-0.030704	0.744128
C	2.400751	-0.064235	-0.131710
C	1.211129	0.640177	2.051189
H	1.447760	-0.344534	2.458655
H	1.977510	1.349894	2.364861
H	0.233915	0.963990	2.418465
C	-0.943963	0.438599	-0.449973
C	0.613541	-1.524246	-0.379032
C	1.158366	0.582004	0.538674
C	4.695465	-0.549748	0.266568
H	5.342437	-0.614981	1.139505
H	4.559209	-1.545481	-0.164218
H	5.142519	0.123475	-0.469195
C	2.686603	0.469381	-1.520823
H	3.096494	1.478845	-1.449988
H	3.393251	-0.184521	-2.033840
H	1.765137	0.505942	-2.107689
C	-0.489844	1.809184	-0.470963
O	-1.054199	2.809873	-0.858175
C	-2.236543	-0.087442	-0.941948
H	-2.648745	0.654499	-1.634525
H	-2.057502	-1.015585	-1.497965
C	-3.283915	-0.370245	0.174264
C	-3.605262	0.922836	0.929955
H	-3.965657	1.699092	0.245996
H	-2.723546	1.311009	1.452428
H	-4.382975	0.738704	1.678518
C	-2.752388	-1.427704	1.146953
H	-1.874798	-1.066134	1.693708
H	-2.468199	-2.342205	0.615253
H	-3.523617	-1.680701	1.882168
C	-4.549250	-0.893607	-0.513112
H	-4.344104	-1.820290	-1.059952
H	-5.325774	-1.102209	0.230153
H	-4.945041	-0.157458	-1.221188

Structure **3g**$^{-}$

O	0.740836	1.818367	-0.095560
O	2.193821	-0.730843	-1.123492
O	0.416923	-1.578013	-2.203063
N	0.155609	-0.373260	-0.228710
O	2.885237	-0.810363	1.068164
C	2.451503	0.050363	0.059206
C	0.802363	0.606557	1.970078
H	0.924687	-0.376236	2.424607
H	1.508973	1.305091	2.426350
H	-0.214602	0.969960	2.139588
C	-1.016627	0.433607	-0.572762
C	0.859643	-0.947598	-1.267341
C	1.041224	0.550998	0.472494
C	4.153013	-1.423815	0.858705
H	4.231202	-2.217232	1.601505
H	4.219020	-1.858128	-0.143413
H	4.967836	-0.709200	1.009700
C	3.444139	1.138212	-0.289990
H	3.701507	1.700301	0.611793
H	4.351249	0.703262	-0.713881
H	2.999064	1.815218	-1.018072
C	-0.634487	1.750030	-0.533912
O	-1.197663	2.842942	-0.774505
C	-2.349616	-0.157342	-0.877283
H	-2.911572	0.578863	-1.469393
H	-2.225559	-1.042066	-1.519216
C	-3.233324	-0.563713	0.330459
C	-3.587939	0.676527	1.154590
H	-4.164210	1.390671	0.554295
H	-2.678302	1.180886	1.498669
H	-4.188136	0.404597	2.030795
C	-2.491362	-1.570275	1.215424
H	-1.587833	-1.122842	1.641596
H	-2.188378	-2.451017	0.635983
H	-3.133476	-1.906888	2.038141
C	-4.517872	-1.206405	-0.203161
H	-4.291942	-2.109411	-0.782529
H	-5.185180	-1.490106	0.618902
H	-5.060790	-0.512508	-0.855841

Structure **3g**$^{-}$_*conf2*

O	0.835717	1.684823	-0.559718
O	1.982865	-1.175052	-0.826450
O	0.012327	-2.229069	-0.932428
N	0.273865	-0.307938	0.369621
O	3.276412	-0.471420	0.938558
C	2.554329	-0.030126	-0.172093
C	1.509782	1.383242	1.720430
H	1.800651	0.654852	2.476283
H	2.284266	2.150987	1.642161
H	0.568271	1.859538	2.007143
C	-0.971330	0.421113	0.097892
C	0.669780	-1.311440	-0.495847
C	1.313949	0.720547	0.370329
C	4.440130	-1.241534	0.656038
H	4.740528	-1.693346	1.601044
H	4.222867	-2.030948	-0.069759
H	5.252783	-0.608789	0.286304
C	3.385377	0.737009	-1.178340
H	3.876812	1.577686	-0.681262
H	4.143614	0.086509	-1.618584
H	2.739439	1.116979	-1.968945
C	-0.598975	1.630341	-0.457112
O	-1.226824	2.642071	-0.824630
C	-2.232099	0.201723	0.880713
H	-1.984647	-0.410504	1.761134
H	-2.569670	1.179207	1.265034
C	-3.461541	-0.437094	0.186713
C	-3.157151	-1.868547	-0.255876
H	-2.866228	-2.487491	0.602630
H	-2.330201	-1.888325	-0.969338
H	-4.041383	-2.323944	-0.718271
C	-3.862300	0.401192	-1.029918
H	-3.027743	0.474122	-1.734913
H	-4.136173	1.419275	-0.729236
H	-4.720701	-0.048208	-1.543627
C	-4.618359	-0.456025	1.192598
H	-4.865318	0.558266	1.528119
H	-5.518573	-0.892260	0.744365
H	-4.359183	-1.051264	2.076547

Structure ^{i}**Pr**$^{\bullet}$

C	0.000000	0.544660	-0.062183
C	-1.290235	-0.200655	0.004909
H	-1.296212	-1.043353	-0.697774
H	-2.146927	0.439766	-0.219916
H	-1.458461	-0.632206	1.005394
C	1.290235	-0.200655	0.004909
H	1.458461	-0.632206	1.005394
H	2.146927	0.439766	-0.219916
H	1.296212	-1.043354	-0.697774
H	-0.000000	1.611484	0.138777

Structure **3h_ts**

O	0.333102	1.742668	0.057895
O	1.964281	-1.309625	-0.426891
O	0.257384	-2.399142	-1.433845
N	-0.083505	-0.492846	-0.165525
O	3.049168	0.058101	1.084124
C	2.180654	0.071970	0.022409
C	0.589826	0.414394	2.039771
H	0.891029	-0.564997	2.415501
H	1.202259	1.182550	2.513701
H	-0.463476	0.587013	2.276058
C	-1.155507	0.187978	-0.778155
C	0.668586	-1.492414	-0.753479
C	0.763905	0.466817	0.537402

C	4.412130	-0.251142	0.786480
H	4.896998	-0.406251	1.748796
H	4.481221	-1.165307	0.190332
H	4.896562	0.578165	0.264575
C	2.637753	0.876021	-1.179144
H	2.879066	1.896377	-0.874655
H	3.512821	0.404700	-1.629020
H	1.845235	0.911927	-1.930675
C	-0.848202	1.623905	-0.622794
O	-1.459891	2.585272	-1.013976
C	-2.234903	-0.325895	-1.396548
H	-2.904012	0.353665	-1.912235
H	-2.325052	-1.390927	-1.569513
C	-3.940633	-0.683229	0.359183
C	-4.086496	0.701298	0.890917
H	-4.511610	1.384952	0.151539
H	-3.115251	1.098765	1.214271
H	-4.739914	0.716505	1.776553
C	-3.214608	-1.696389	1.179109
H	-2.239671	-1.308881	1.501777
H	-3.052033	-2.629500	0.632870
H	-3.777029	-1.939913	2.093511
H	-4.679980	-1.037008	-0.354071

Structure **3h**$^{\bullet}$

O	-0.685467	-1.854847	-0.084505
O	-1.668110	1.528104	-0.133120
O	0.355286	2.475384	-0.489522
N	0.123326	0.257690	0.169701
O	-3.270052	0.084754	0.696962
C	-2.182091	0.141722	-0.130282
C	-1.122201	-0.799519	2.028561
H	-1.333017	0.157182	2.510043
H	-1.934484	-1.493938	2.246235
H	-0.179929	-1.201934	2.407770
C	1.129361	-0.486640	-0.407898
C	-0.325600	1.530417	-0.189551
C	-0.998092	-0.616625	0.530717
C	-4.470289	0.695230	0.215599
H	-5.151202	0.716270	1.064576
H	-4.275919	1.716305	-0.123867
H	-4.911677	0.105070	-0.591405
C	-2.429849	-0.259261	-1.570538
H	-2.884900	-1.251153	-1.603040
H	-3.084899	0.467258	-2.053449
H	-1.486340	-0.288623	-2.122097
C	0.620509	-1.828087	-0.551034
O	1.148433	-2.818311	-1.011035
C	2.437944	0.051926	-0.838982
H	2.999990	-0.770188	-1.296342
H	2.277514	0.817151	-1.610646
C	3.248564	0.670647	0.319831
C	4.538810	1.276717	-0.229015
H	5.163466	0.499506	-0.684661
H	4.330344	2.035326	-0.989746
H	5.116677	1.746400	0.572295
C	3.542933	-0.368202	1.401343
H	4.116482	-1.203758	0.982621
H	4.130707	0.073926	2.211035
H	2.623631	-0.773951	1.836625
H	2.639557	1.474694	0.753410

Structure **3h**$^{-}$

O	-0.305887	-1.785903	0.040704
O	-2.006516	0.531826	-1.150347
O	-0.335039	1.437220	-2.344817
N	0.073116	0.430651	-0.286317
O	-2.661143	0.725224	1.045471
C	-2.168496	-0.171399	0.096806
C	-0.438613	-0.416415	2.001614
H	-0.641462	0.585748	2.378272
H	-1.069454	-1.135078	2.531694
H	0.610443	-0.671665	2.172612
C	1.307561	-0.293118	-0.595339
C	-0.700433	0.851629	-1.348380
C	-0.710353	-0.507453	0.511340
C	-3.983956	1.202214	0.821877
H	-4.119814	2.045014	1.499088
H	-4.110021	1.543033	-0.210171
H	-4.726200	0.432268	1.053508
C	-3.062496	-1.369471	-0.140169
H	-3.248686	-1.879139	0.809009
H	-4.014268	-1.054027	-0.571884
H	-2.570782	-2.059000	-0.825340
C	1.049819	-1.630446	-0.437657
O	1.706785	-2.684737	-0.602250
C	2.575400	0.392189	-0.978631
H	3.196006	-0.337964	-1.515040
H	2.358471	1.199656	-1.691926
C	3.410719	0.976176	0.182838
C	3.931922	-0.129757	1.099257
H	4.557476	-0.840005	0.547734
H	3.091068	-0.686294	1.529642
H	4.526587	0.283156	1.921250
C	2.615824	2.009158	0.982265
H	1.766593	1.530111	1.481802
H	2.217405	2.795782	0.331493
H	3.242310	2.481068	1.746941
H	4.275533	1.483897	-0.269482

Structure **3h**$^{-}$_*conf2*

O	-0.686881	1.788576	0.244303
O	-1.665999	-1.535990	0.029442
O	0.402192	-2.427684	-0.152980
N	-0.024301	-0.201402	-0.650687
O	-3.429059	-0.082898	-0.298259
C	-2.175887	-0.193005	0.274432
C	-1.673006	1.187179	-1.858028

H	-2.038349	0.351736	-2.458826
H	-2.480691	1.908532	-1.720100
H	-0.836459	1.669338	-2.371314
C	1.162117	0.571837	-0.362192
C	-0.329207	-1.458195	-0.241263
C	-1.170106	0.698744	-0.511307
C	-4.469219	-0.831203	0.325241
H	-5.317546	-0.794422	-0.357343
H	-4.166705	-1.872164	0.473689
H	-4.757111	-0.386517	1.282391
C	-2.143777	0.043592	1.773388
H	-2.597344	1.007807	2.011700
H	-2.675798	-0.757142	2.290481
H	-1.106341	0.054032	2.116457
C	0.753910	1.766149	0.178047
O	1.346331	2.790638	0.583490
C	2.528339	0.180696	-0.812531
H	2.459547	-0.300518	-1.800193
H	3.112906	1.103088	-0.952423
C	3.332598	-0.742646	0.125248
C	4.665702	-1.118179	-0.521959
H	4.514593	-1.619253	-1.484400
H	5.269523	-0.219616	-0.702953
H	5.247713	-1.786916	0.120849
C	3.555922	-0.079062	1.482580
H	4.084681	-0.748231	2.169870
H	4.157623	0.831857	1.366905
H	2.601956	0.205981	1.937560
H	2.740926	-1.654355	0.274559

Structure **Et•**

C	0.794724	-0.000001	-0.027650
C	-0.694641	-0.000002	-0.000264
H	-1.106446	-0.886951	-0.490729
H	-1.106440	0.886883	-0.490852
H	-1.084355	0.000073	1.029583
H	1.348364	0.927077	0.059744
H	1.348382	-0.927068	0.059744

Structure **3Et_ts**

O	-0.124029	1.715414	0.590695
O	-1.258178	-1.416855	-0.368104
O	0.670715	-2.158513	-1.279027
N	0.247660	0.099093	-0.981163
O	-3.010252	0.049173	-0.052435
C	-1.703553	-0.189401	0.298122
C	-1.510602	1.798815	-1.362744
H	-2.018267	1.195653	-2.116442
H	-2.244101	2.421400	-0.848696
H	-0.762058	2.432741	-1.844631
C	1.499246	0.677751	-0.678691
C	-0.035302	-1.253287	-0.909884
C	-0.806816	0.900478	-0.368944
C	-3.986256	-0.837989	0.496620
H	-4.911982	-0.624959	-0.035285
H	-3.700544	-1.880420	0.330410
H	-4.128920	-0.650732	1.564083
C	-1.459627	-0.360623	1.784582
H	-1.857181	0.499784	2.326972
H	-1.938625	-1.276000	2.135239
H	-0.388559	-0.434185	1.984929
C	1.224723	1.680581	0.367335
O	1.993310	2.381729	0.976752
C	2.719378	0.324949	-1.131518
H	2.830793	-0.354795	-1.966280
H	3.570635	0.911924	-0.807591
C	3.510742	-1.453020	0.299636
C	2.641718	-1.329659	1.502451
H	1.602358	-1.576404	1.263042
H	2.677491	-0.320638	1.927072
H	2.958035	-2.023610	2.293662
H	3.313210	-2.248787	-0.409185
H	4.529662	-1.083776	0.346000

Structure **3Et•**

O	-0.184296	-1.807307	-0.010621
O	-1.557314	1.430829	-0.197471
O	0.330965	2.584114	-0.669216
N	0.378150	0.394978	0.112828
O	-2.956686	-0.140996	0.756528
C	-1.904902	-0.003444	-0.107209
C	-0.684159	-0.698817	2.060909
H	-0.991715	0.251619	2.500946
H	-1.403024	-1.469832	2.340465
H	0.309161	-0.968540	2.427600
C	1.450869	-0.258220	-0.454795
C	-0.226285	1.585098	-0.297873
C	-0.623295	-0.582750	0.552529
C	-4.232708	0.297811	0.283070
H	-4.889454	0.284900	1.151027
H	-4.169508	1.314767	-0.113717
H	-4.621414	-0.382688	-0.478657
C	-2.141150	-0.508236	-1.516408
H	-2.476780	-1.546628	-1.483638
H	-2.889442	0.109921	-2.014375
H	-1.215310	-0.457633	-2.095687
C	1.098019	-1.654962	-0.516730
O	1.726348	-2.600596	-0.943131
C	2.685547	0.401329	-0.937747
H	3.328863	-0.378850	-1.356527
H	2.431627	1.096225	-1.748396
C	3.417194	1.165308	0.180929
C	3.849915	0.254112	1.326654
H	4.486885	-0.557665	0.960040
H	2.984340	-0.198039	1.822980
H	4.411143	0.812194	2.080324
H	4.290244	1.653404	-0.262935
H	2.760921	1.960277	0.552063

Structure **3Et⁻**

O	0.347235	1.800101	-0.068311
O	1.697130	-0.788908	-1.124520
O	-0.161748	-1.657740	-2.034290
N	-0.264488	-0.389954	-0.084758
O	2.548932	-0.817628	1.011110
C	2.050269	0.021032	0.013917
C	0.553335	0.651332	2.024983
H	0.713884	-0.316239	2.499204
H	1.288027	1.365863	2.405792
H	-0.450205	1.016968	2.257604
C	-1.447846	0.418722	-0.377464
C	0.356134	-1.002454	-1.156691
C	0.679932	0.548399	0.516827
C	3.789051	-1.456058	0.724586
H	3.911159	-2.231533	1.480311
H	3.774115	-1.916702	-0.267755
H	4.622617	-0.750782	0.795791
C	3.025198	1.086462	-0.438486
H	3.350452	1.672867	0.425046
H	3.896396	0.628592	-0.910823
H	2.536372	1.745663	-1.154813
C	-1.060511	1.733321	-0.391249
O	-1.638583	2.825417	-0.599910
C	-2.812032	-0.173449	-0.444297
H	-3.504452	0.627792	-0.732666
H	-2.877889	-0.937309	-1.234447
C	-3.285909	-0.801434	0.874667
H	-3.310201	-0.023056	1.647618
C	-4.657834	-1.461013	0.749294
H	-5.413697	-0.731136	0.438755
H	-4.638858	-2.258188	-0.001870
H	-4.984640	-1.900404	1.696332
H	-2.544118	-1.541814	1.200082

Structure **3Et⁻**_*conf2*

O	0.471416	1.779731	-0.327610
O	1.464236	-1.533781	-0.018213
O	-0.606490	-2.438600	0.043187
N	-0.225291	-0.206741	0.547253
O	3.191595	-0.062960	0.408835
C	1.979123	-0.188520	-0.242648
C	1.332906	1.211695	1.837693
H	1.670944	0.387579	2.469252
H	2.139098	1.941309	1.739955
H	0.461694	1.689715	2.294057
C	-1.398865	0.552707	0.184118
C	0.112803	-1.464234	0.168437
C	0.918437	0.702739	0.468597
C	4.276922	-0.805711	-0.139738
H	5.080236	-0.751216	0.594162
H	3.996075	-1.851811	-0.294557
H	4.619397	-0.368211	-1.082097
C	2.042602	0.033398	-1.742717
H	2.506609	0.997388	-1.961161
H	2.610568	-0.769654	-2.216120
H	1.029516	0.035144	-2.151969
C	-0.970254	1.746917	-0.340267
O	-1.548587	2.763943	-0.783889
C	-2.787741	0.152100	0.552053
H	-2.769052	-0.371705	1.519883
H	-3.375945	1.068065	0.706154
C	-3.528208	-0.727360	-0.467534
H	-3.576038	-0.188431	-1.422398
C	-4.936702	-1.090142	-0.000875
H	-4.902052	-1.647913	0.941739
H	-5.536813	-0.189247	0.169915
H	-5.461031	-1.707765	-0.735960
H	-2.939675	-1.634257	-0.642828

Structure HCO_3^-

C	0.000000	0.144821	-0.000000
O	-1.012017	0.870389	-0.000000
O	1.212674	0.455764	-0.000000
O	-0.273453	-1.225534	0.000000
H	0.582371	-1.673879	0.000000

Structure 3g⁻_TSprot_*re*

O	0.704518	-0.525921	1.755077
O	2.242783	-0.165129	-1.368995
O	0.427023	0.431366	-2.569725
N	0.232194	-0.554284	-0.480808
O	3.540813	-1.283487	0.176275
C	2.509053	-0.374725	0.051153
C	1.249462	-2.553279	0.592613
H	1.593099	-2.958378	-0.361894
H	1.940457	-2.862833	1.378470
H	0.252234	-2.941236	0.809823
C	-0.892087	0.129767	0.176694
C	0.899829	-0.031818	-1.554301
C	1.181820	-1.039803	0.518588
C	4.836670	-0.816628	-0.191245
H	5.474708	-1.698946	-0.224973
H	4.816341	-0.343963	-1.177735
H	5.228457	-0.115080	0.550702
C	2.764787	0.977315	0.686411
H	3.036572	0.849557	1.736785
H	3.572516	1.486241	0.156182
H	1.863421	1.594013	0.612065
C	-0.572351	-0.014813	1.595456
O	-1.203090	0.326774	2.574955
C	-2.311620	-0.130094	-0.291682
H	-2.945718	0.468300	0.375186
H	-2.425305	0.316290	-1.289159
C	-2.874156	-1.568456	-0.340172
C	-2.783914	-2.242763	1.032249
H	-3.309682	-1.655580	1.793171
H	-1.746082	-2.358067	1.361691
H	-3.231871	-3.242438	0.996937
C	-2.149598	-2.421608	-1.390038
H	-1.103064	-2.588190	-1.125029

H	-2.172360	-1.928981	-2.369582
H	-2.640703	-3.396952	-1.489148
C	-4.352837	-1.462602	-0.739528
H	-4.458978	-0.977775	-1.716829
H	-4.809189	-2.456945	-0.803238
H	-4.916836	-0.875247	-0.006101
C	-0.880399	3.596433	-0.039824
O	-2.095225	3.348544	0.266507
O	-0.032267	2.578936	-0.158542
H	-0.612110	1.399285	0.032589
O	-0.431955	4.774587	-0.239616

Structure 3g$^-$_*conf2***_TSprot_***re*

O	1.175485	-0.953387	1.653852
O	2.187096	0.387900	-1.379321
O	0.155252	0.694107	-2.297859
N	0.477749	-0.752645	-0.508574
O	3.899482	-0.711546	-0.296194
C	2.690695	-0.069746	-0.094511
C	2.036705	-2.570092	0.112902
H	2.378021	-2.723631	-0.912435
H	2.839789	-2.839208	0.801437
H	1.164135	-3.202130	0.302298
C	-0.741344	-0.678939	0.312785
C	0.842928	0.170136	-1.449855
C	1.623663	-1.124737	0.313470
C	4.994905	0.123012	-0.662671
H	5.794544	-0.549065	-0.972129
H	4.725697	0.777034	-1.497502
H	5.335255	0.723369	0.185978
C	2.748187	1.126497	0.835297
H	3.198659	0.838407	1.788129
H	3.338363	1.921183	0.373970
H	1.731933	1.495545	1.006018
C	-0.200821	-0.900653	1.664879
O	-0.795172	-0.995854	2.718817
C	-1.869611	-1.652076	-0.054263
H	-1.511603	-2.285023	-0.878274
H	-2.055110	-2.320572	0.799433
C	-3.225828	-1.043295	-0.455542
C	-3.078834	-0.196919	-1.721191
H	-2.681434	-0.798828	-2.548168
H	-2.406559	0.645861	-1.540275
H	-4.054919	0.200978	-2.026659
C	-3.760955	-0.169624	0.683448
H	-3.109050	0.696820	0.838271
H	-3.832143	-0.743900	1.615247
H	-4.762165	0.205092	0.436301
C	-4.200545	-2.197438	-0.718011
H	-4.334758	-2.812936	0.179717
H	-5.183312	-1.813780	-1.014953
H	-3.834151	-2.846609	-1.522487
C	-1.196360	2.950485	0.382800
O	-0.648632	4.068546	0.670955
O	-0.566710	1.844764	0.775797
H	-0.932574	0.650097	0.440605
O	-2.297996	2.846739	-0.245013

Structure 3g$^-$_TSprot_*si*

O	-1.249861	0.669977	1.727497
O	-2.614119	-1.322607	-0.198717
O	-0.852271	-2.647030	-0.573440
N	-0.573007	-0.354693	-0.194504
O	-3.282531	0.619094	-1.226193
C	-2.886064	0.072280	-0.006352
C	-1.252582	2.056935	-0.235094
H	-1.383402	2.066503	-1.316324
H	-1.974263	2.741665	0.219220
H	-0.236050	2.369077	0.014903
C	0.628150	-0.258121	0.698411
C	-1.275726	-1.538698	-0.333350
C	-1.486021	0.662195	0.305025
C	-4.529319	0.158053	-1.738275
H	-4.567800	0.485566	-2.776650
H	-4.590252	-0.933602	-1.700176
H	-5.367927	0.598766	-1.190986
C	-3.913072	0.221765	1.096078
H	-4.163738	1.278402	1.221319
H	-4.817717	-0.334176	0.843302
H	-3.510968	-0.165281	2.031450
C	0.005517	0.157458	1.964164
O	0.456943	0.175637	3.094593
C	1.536687	-1.473001	0.819022
H	2.065344	-1.346328	1.773114
H	0.935265	-2.384007	0.920837
C	2.593720	-1.700720	-0.288464
C	3.811766	-0.799174	-0.053239
H	4.284494	-1.026918	0.910387
H	3.518290	0.254088	-0.054469
H	4.558805	-0.954473	-0.841152
C	2.024065	-1.407234	-1.680644
H	1.780446	-0.342630	-1.769659
H	1.124390	-2.001610	-1.868759
H	2.770667	-1.654804	-2.446216
C	3.038272	-3.166373	-0.211073
H	2.202660	-3.838625	-0.437579
H	3.843138	-3.366927	-0.927754
H	3.409481	-3.411537	0.791823
C	2.454297	2.535399	-0.600566
O	3.261322	3.516900	-0.485601
O	1.995759	1.980019	0.520828
H	1.340599	0.882938	0.435930
O	2.071190	2.063282	-1.719576

Structure 3g$^-$_*conf2***_TSprot_***si*

O	-0.848816	0.030197	1.822521
O	-2.209564	-0.428096	-1.336170
O	-0.288182	-0.785575	-2.470937
N	-0.322541	0.200257	-0.370071
O	-3.652273	0.732317	0.041204
C	-2.591482	-0.154627	0.040134
C	-1.468345	2.081097	0.737581
H	-1.839267	2.511429	-0.194370
H	-2.166139	2.320054	1.542405
H	-0.478558	2.491168	0.959495
C	0.957885	0.079366	0.335875

C	-0.845457	-0.379538	-1.467880
C	-1.332737	0.576261	0.592813
C	-4.894311	0.225392	-0.440232
H	-5.541961	1.091808	-0.568744
H	-4.764099	-0.277488	-1.403046
H	-5.347943	-0.460000	0.281591
C	-2.886016	-1.477842	0.719928
H	-3.245041	-1.305179	1.736998
H	-3.636516	-2.028566	0.150065
H	-1.976878	-2.079413	0.764499
C	0.508835	-0.214240	1.702857
O	1.144003	-0.529011	2.689739
C	2.047366	-0.749941	-0.325384
H	2.013891	-0.472779	-1.383445
H	3.009697	-0.374959	0.046977
C	2.065971	-2.303447	-0.204656
C	2.699908	-2.859732	-1.486483
H	3.690078	-2.418644	-1.651682
H	2.074416	-2.629566	-2.356042
H	2.820000	-3.947464	-1.423495
C	0.669559	-2.912850	-0.038803
H	0.019085	-2.668796	-0.883965
H	0.194168	-2.573076	0.888298
H	0.746146	-4.004690	0.018893
C	2.929469	-2.725212	0.993962
H	2.531380	-2.315754	1.926127
H	2.963431	-3.818113	1.075747
H	3.957375	-2.363876	0.872755
C	2.184587	3.194943	-0.400383
O	2.610562	4.390086	-0.260081
O	2.021184	2.458904	0.695038
H	1.470621	1.282288	0.458790
O	1.909650	2.676432	-1.534230

Table S6. Cartesian coordinates of the lowest energy structures calculated with PCM(THF)/M06-2X/6-31+G(d,p)

Structure **1**_*THF*

C	1.975128	-1.129864	-0.295420
C	-0.078834	-0.577372	0.575265
C	-1.327571	-0.130375	-0.241094
C	0.015865	1.721303	0.087220
O	-2.455098	-0.572696	0.401176
C	-3.703274	-0.270394	-0.224573
H	-3.763333	0.792162	-0.475919
H	-4.468616	-0.514925	0.510025
H	-3.850299	-0.880633	-1.119619
C	-1.245462	-0.471324	-1.716508
H	-2.025382	0.061167	-2.262748
H	-1.359206	-1.548121	-1.856534
H	-0.278055	-0.164808	-2.122427
O	-1.257404	1.331344	-0.119732
O	0.430784	2.836999	-0.085078
N	0.744756	0.628215	0.535434
C	2.046101	0.306969	0.119812
C	3.122115	1.082501	0.024032
H	3.099996	2.122072	0.325869
H	4.031903	0.657565	-0.383691
O	2.840689	-1.824731	-0.748046
O	0.701588	-1.567342	-0.099708
C	-0.367854	-1.045285	1.984049
H	-0.934766	-1.976609	1.958718
H	-0.949526	-0.288182	2.512524
H	0.582241	-1.202737	2.500156

Structure **Malonate**

C	0.000000	0.555810	0.000019
H	0.000001	1.637367	0.000025
C	-1.251484	-0.102446	0.011746
O	-1.526142	-1.306323	0.041198
O	-2.292117	0.818331	-0.010707
C	1.251483	-0.102445	-0.011710
O	1.526139	-1.306324	-0.041196
O	2.292118	0.818331	0.010714
C	-3.598772	0.267990	-0.011569
H	-4.282028	1.117741	-0.037028
H	-3.762070	-0.365236	-0.888299
H	-3.780627	-0.327937	0.887367
C	3.598776	0.267988	0.011527
H	3.762104	-0.365235	0.888253
H	3.780595	-0.327938	-0.887416
H	4.282030	1.117742	0.036960

Structure **3a_ts**

O	-1.102926	-1.886566	0.119106
O	-3.086390	0.233631	-1.078225
O	-1.669330	1.060026	-2.613304
N	-0.894083	0.282893	-0.563115
O	-3.432896	0.624491	1.160499
C	-3.018695	-0.337879	0.244242
C	-0.991076	-0.243697	1.866395
H	-1.253577	0.775315	2.148302
H	-1.441123	-0.945935	2.573837
H	0.097864	-0.349698	1.868510
C	0.275810	-0.441465	-1.003096
C	-1.853135	0.572850	-1.524327
C	-1.495923	-0.547275	0.473405
C	-4.799517	1.014290	1.078685
H	-4.890850	1.917159	1.681693
H	-5.083319	1.237963	0.046016
H	-5.456538	0.240785	1.488197

C	-3.851119	-1.602638	0.258861
H	-3.850620	-2.027006	1.266328
H	-4.877037	-1.380529	-0.040453
H	-3.428921	-2.328677	-0.434777
C	0.073048	-1.811325	-0.617185
O	0.714046	-2.821138	-0.852684
C	1.405959	0.087545	-1.577661
H	2.081311	-0.614897	-2.057217
H	1.368413	1.086087	-2.000815
C	2.947247	0.658606	-0.176291
H	3.722899	0.858013	-0.905421
C	2.314572	1.806895	0.423533
O	1.599010	1.855642	1.413039
O	2.554109	2.932070	-0.322000
C	2.999827	-0.602495	0.509138
O	2.297233	-0.977955	1.438909
O	3.887803	-1.454102	-0.088008
C	1.893289	4.111729	0.124244
H	2.199593	4.368434	1.141238
H	2.189294	4.899273	-0.567782
H	0.808612	3.978421	0.103290
C	3.843269	-2.800671	0.379718
H	2.852510	-3.225881	0.197270
H	4.601790	-3.336789	-0.190370
H	4.071679	-2.847908	1.447343

Structure **3a**$^{-}$

O	-2.016630	1.966586	-0.485849
O	-2.709856	-0.459020	1.328272
O	-0.817045	-0.394311	2.534984
N	-0.820006	0.189589	0.280190
O	-3.228823	-1.403851	-0.701234
C	-3.120174	-0.186989	-0.022823
C	-1.614371	0.224334	-2.081737
H	-1.417251	-0.839320	-2.207918
H	-2.471060	0.516077	-2.695592
H	-0.737459	0.795517	-2.396506
C	0.033549	1.374992	0.322138
C	-1.377140	-0.227676	1.476163
C	-1.901153	0.557342	-0.630482
C	-4.254561	-2.276582	-0.244623
H	-4.057868	-3.243509	-0.707590
H	-4.224952	-2.383555	0.844057
H	-5.243046	-1.924877	-0.557229
C	-4.406072	0.610708	0.014550
H	-4.770205	0.762296	-1.005217
H	-5.165057	0.081689	0.594383
H	-4.217050	1.580706	0.472780
C	-0.706987	2.447562	-0.112452
O	-0.491547	3.667437	-0.251581
C	1.470827	1.298200	0.683385
H	1.865646	2.316541	0.740588
H	1.623120	0.834541	1.666005
C	2.314585	0.517005	-0.348256
H	2.310311	1.044721	-1.308063
C	3.763983	0.391371	0.088223
O	4.289864	1.062231	0.946473
O	4.416245	-0.538385	-0.621312
C	1.723211	-0.853759	-0.618972
O	1.347697	-1.249465	-1.699585
O	1.652780	-1.576857	0.502714
C	5.804001	-0.709624	-0.305588
H	5.916358	-1.006059	0.738650
H	6.161042	-1.494343	-0.968728
H	6.346106	0.220931	-0.482408
C	0.936689	-2.810040	0.401128
H	1.437267	-3.485779	-0.295215
H	0.925275	-3.229030	1.405152
H	-0.080944	-2.609652	0.054546

Structure **3a**$^{-}$_*conf2*

O	1.870403	1.653491	0.257041
O	2.639789	-1.032797	-1.147124
O	0.584301	-1.934551	-1.096743
N	1.314536	-0.529119	0.607408
O	4.353359	-0.924279	0.381177
C	3.438705	-0.151349	-0.337264
C	2.970991	0.587330	2.096465
H	3.330537	-0.360963	2.493941
H	3.792979	1.308061	2.083342
H	2.168097	0.976650	2.727198
C	0.126724	0.291833	0.820448
C	1.419205	-1.223378	-0.583300
C	2.423685	0.419287	0.691270
C	5.340732	-1.594276	-0.392822
H	5.810863	-2.315610	0.275384
H	4.887476	-2.124169	-1.236061
H	6.100368	-0.895330	-0.757291
C	4.083513	0.878108	-1.240413
H	4.753330	1.510754	-0.651661
H	4.653029	0.385406	-2.030979
H	3.310511	1.499695	-1.690262
C	0.460284	1.597158	0.550853
O	-0.169578	2.673269	0.535429
C	-1.191629	-0.259072	1.227683
H	-1.055340	-1.172403	1.816349
H	-1.714873	0.462770	1.866237
C	-2.131567	-0.600386	0.044201
H	-1.650037	-1.340374	-0.602382
C	-3.453877	-1.168248	0.518263
O	-3.712086	-1.490555	1.656404
O	-4.320689	-1.305266	-0.494916
C	-2.354727	0.638857	-0.798242
O	-1.887431	0.827819	-1.898718
O	-3.111259	1.531205	-0.150071
C	-5.601456	-1.849161	-0.154424
H	-6.098310	-1.209518	0.577183
H	-6.165267	-1.878785	-1.084178

H	-5.485682	-2.853222	0.257567	H	-3.865287	3.404353	-0.119265
C	-3.212421	2.820833	-0.765701	H	-2.215683	3.264213	-0.813647
H	-3.641903	2.730401	-1.765266				
